# SW1PerS: Sliding windows and 1-persistence scoring; discovering periodicity in gene expression time series data

**DOI:** 10.1186/s12859-015-0645-6

**Published:** 2015-08-16

**Authors:** Jose A. Perea, Anastasia Deckard, Steve B. Haase, John Harer

**Affiliations:** 1 Department of Mathematics, Duke University, Science Dr, Durham, 27708 NC USA; 2 Institute for Mathematics and its Applications (IMA), University of Minnesota, Minneapolis, MN USA; 3 Program in Computational Biology and Bioinformatics, Duke University, Durham, 27708 NC USA; 4 Center for Systems Biology, Institute for Genome Sciences & Policy, Duke University, Durham, 27708 NC USA; 5 Department of Biology, Duke University, Durham, 27708 NC USA; 6 Department of Computer Science and Department of Electrical and Computer Engineering, Duke University, Science Dr, Durham, 27708 NC USA

**Keywords:** Periodicity, Gene expression, Time series, Sliding windows, Persistent homology

## Abstract

**Background:**

Identifying periodically expressed genes across different processes (e.g. the cell and metabolic cycles, circadian rhythms, etc) is a central problem in computational biology. Biological time series may contain (multiple) unknown signal shapes of systemic relevance, imperfections like noise, damping, and trending, or limited sampling density. While there exist methods for detecting periodicity, their design biases (e.g. toward a specific signal shape) can limit their applicability in one or more of these situations.

**Methods:**

We present in this paper a novel method, **SW1PerS**, for quantifying periodicity in time series in a shape-agnostic manner and with resistance to damping. The measurement is performed directly, without presupposing a particular pattern, by evaluating the circularity of a high-dimensional representation of the signal. SW1PerS is compared to other algorithms using synthetic data and performance is quantified under varying noise models, noise levels, sampling densities, and signal shapes. Results on biological data are also analyzed and compared.

**Results:**

On the task of periodic/not-periodic classification, using synthetic data, SW1PerS outperforms all other algorithms in the low-noise regime. SW1PerS is shown to be the most shape-agnostic of the evaluated methods, and the only one to consistently classify damped signals as highly periodic. On biological data, and for several experiments, the lists of top 10% genes ranked with SW1PerS recover up to 67% of those generated with other popular algorithms. Moreover, the list of genes from data on the Yeast metabolic cycle which are highly-ranked only by SW1PerS, contains evidently non-cosine patterns (e.g. ECM33, CDC9, SAM1,2 and MSH6) with highly periodic expression profiles. In data from the Yeast cell cycle SW1PerS identifies genes not preferred by other algorithms, hence not previously reported as periodic, but found in other experiments such as the universal growth rate response of Slavov. These genes are BOP3, CDC10, YIL108W, YER034W, MLP1, PAC2 and RTT101.

**Conclusions:**

In biological systems with low noise, i.e. where periodic signals with interesting shapes are more likely to occur, SW1PerS can be used as a powerful tool in exploratory analyses. Indeed, by having an initial set of periodic genes with a rich variety of signal types, pattern/shape information can be included in the study of systems and the generation of hypotheses regarding the structure of gene regulatory networks.

**Electronic supplementary material:**

The online version of this article (doi:10.1186/s12859-015-0645-6) contains supplementary material, which is available to authorized users.

## Background

### Previous Work

Many methods are available for detecting periodicity in time series data [1, 2], and many have been successfully applied in the task of identifying periodic gene expression. Most of these algorithms can be classified into three broad classes, based on how/if they use reference patterns. In particular: approaches which use sinusoidal curves as a base for comparison, user-defined shape templates, and those that do not use a reference pattern. We provide a brief description below.

Methods in the first class determine the period and measure the strength of periodicity by comparing the input time series to sinusoidal curves with different periods. This includes algorithms which transform a time series into the frequency domain, as with the discrete Fourier transform, and those that fit sinusoidal curves to the target signal. The method introduced in [3] uses a Fourier-based approach and a measure of amplitude (as an indicator of regulation strength) to generate a score, as well as a permutation test to asses significance. COSOPT [4] compares a signal to cosine curves with different phases and periods to measure their correspondence, and then uses empirical resampling to compute significance. Lomb-Scargle [5, 6] uses a variation of the discrete Fourier transform to handle unevenly sampled data, and returns a significance score.

Other methods compare the signal to reference curves that are specified by the user. The method of Luan and Li [7], for example, generates a spline function to represent the pattern of known periodic genes, and then uses this shape model to score other signals. JTK_CYCLE [8] determines increasing or decreasing patterns of the observations in both a reference curve and the signal, and then measures the statistical significance of correlation between them.

Other methods, by way of contrast, do not use a set pattern to identify signals of interest, but instead attempt to discover patterns that exist in the data. Address Reduction [9] measures the algorithmic compressibility of the signal; a signal that is more compressible indicates there is a pattern and it might be of biological interest. It is worth noting that non-compressibility does not imply periodicity. An instance of Persistent Homology [10] pairs, in a subtle way, minima and maxima of a time series. This can be used to measure periodicity: if there is only one minimum and maximum pair, it is considered to be a perfect oscillation. Additional oscillations in the time series will create more minimum-maximum pairs, indicating a less perfect curve.

A comparative study of the Lomb-Scargle, Persistent Homology, JTK_CYCLE and de Lichtenberg methods was undertaken in [1]. One of their main conclusions is that curve shape has considerable impact on the scoring of biological signals; this is specially relevant in exploratory settings where the shapes of interest produced by a particular periodic process are not known.

### Our Contribution

SW1PerS, the algorithm introduced here, was designed to help overcome the limitations posed by: Signal-shape biases in the rankings of algorithms which use predetermined templates, the effects of damping in periodicity estimation, and the difficulty of interpreting scores derived from p-values. In a nutshell, SW1PerS transforms the input time series into a high-dimensional set of points (also referred to as a **point cloud**) and interprets periodicity of the original signal as “circularity" of this set. When constructing this point cloud one uses a local normalization process geared toward diminishing the effects of damping. A more in depth description will be presented in the Methods section.

We compare SW1PerS (SW) to existing algorithms, specifically: Lomb-Scargle (LS), de Lichtenberg (DL), JTK_CYCLE (JTK), and Persistent Homology (PH). The first test evaluates their performance on separating periodic from non-periodic signals in a synthetic data set. Their biases for different signal shapes is also analyzed. We then examine how the algorithms behave when applied to real data from different periodic processes and species: the cell cycle in yeast, the metabolic cycle in yeast, and circadian rhythms in mouse.

## Results

### Synthetic data description

**Fig. 1:**
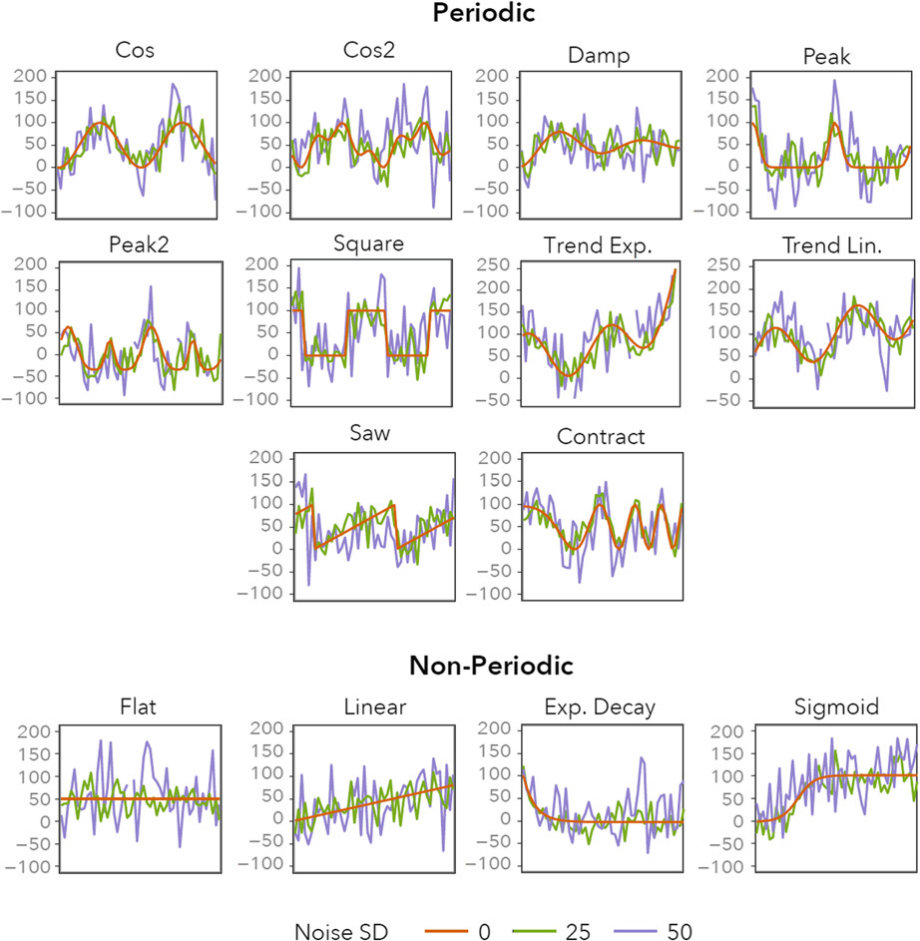
Periodic and Non-Periodic signals in the synthetic data. Signals are shown with additive Gaussian noise with SD = 0, 25, 50. Please refer to an electronic version for colors

The synthetic data used in this paper attempts to capture characteristics found in biological time series, but was generated with known parameters so that results across algorithms could be compared. The periodic shapes included can be seen in Fig. 1; please refer to the supplements (table S1) for the equations which generate these curves.

The periods and amplitudes were fixed, but the phase shifts were allowed to vary from 0 to the length of the period. The period length was 100 (time units) and the signals covered 200 units of time, so each signal spans two cycles. One thousand signals were generated for each signal shape.

Four noise models were applied to the set of signals, each at five different levels: Gaussian Additive with standard deviation *SD* equal to 0,12,25,37 and 50, Laplacian Additive with spread *b* at 0, 8.49, 17.68, 26.16, and 35.36, Gaussian Multiplicative with *SD* equal to 0, 0.12, 0.25, 0.37 and 0.5, and Laplacian Multiplicative with *b*={0,0.08,0.18,0.26,0.35}. The standard deviation *SD* for additive (resp. multiplicative) Gaussian noise and the spread *b* for additive (resp. multiplicative) Laplacian noise were matched ($SD = \sqrt {2}b$) so the distributions would have the same variance. Given the shapes of the distributions, this results in the Laplacian noise model producing signals with more accentuated outliers, as compared to the less extreme behavior of the Gaussian noise. The additive and multiplicative variances were not matched to each other.

### Synthetic Data Analysis

In what follows we present our results on the synthetic data. The first analysis of performance is how well an algorithm can distinguish between periodic and non-periodic signals for several noise models, levels of noise and temporal sampling density. The second explores signal shape bias for each method. For this study JTK, LS, DL, PH and SW1PerS were set to scan for periodicity at a period-length equal to the true period.

**Fig. 2:**
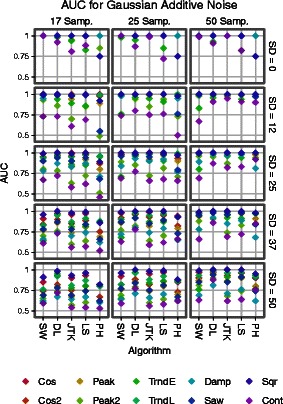
AUC’s showing the algorithms’ performance on identifying periodic signals for different signal shapes, additive Gaussian noise levels (standard deviation = {0,12,25,37, 50}), and number of samples (= {50, 25, 17}). Please refer to an electronic version for colors

**Fig. 3:**
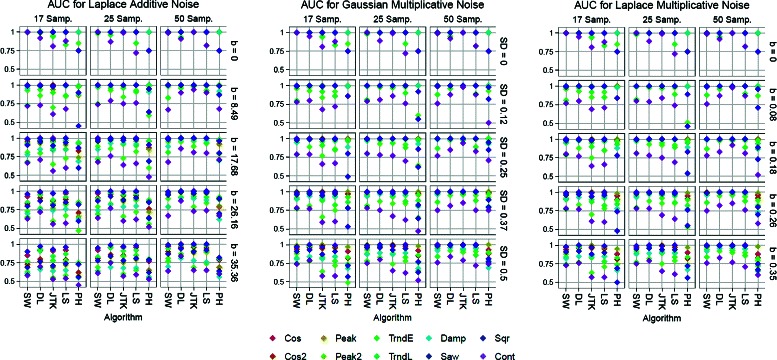
AUC’s showing the algorithms’ performance on identifying periodic signals for different signal shapes, noise models, noise levels (SD and b) and number of samples. Please refer to an electronic version for colors

Receiver Operating Characteristic (ROC) curves provide a succinct visualization of the classification accuracy furnished by a scoring scheme. In a nutshell, each point (*F*,*T*) in the ROC curve records the proportion of signals which have been correctly (*T*) and incorrectly (*F*) classified as periodic for a particular choice of score cut-off. The ROC curve is formed as this choice is varied. It follows that the area under curve (AUC) is an explicit numerical summary for the classification accuracy of a scoring scheme: a value of 1 for the AUC implies a perfect classifier, while a value of 0.5 corresponds to random classification. We report in Figs. 2 and 3 the AUCs obtained on the synthetic data for all algorithms under consideration. The ROC curves for each number of samples, noise model, noise level, and shape can be found in the supplements (Figures S3-S14).

The first thing to notice (see Figs. 2 and 3) is that at the low sampling (17 time points) and low noise regime (SD = 0 to 12 in the additive Gaussian model, SD = 0 to 0.12 in the multiplicative, *b*=0 to 8.49 in the additive Laplacian and *b*=0 to 0.08 in the multiplicative), SW has the best performance among the evaluated algorithms in the task of identifying periodic and non-periodic signals. Moreover, as the number of samples increases and the noise level is kept constant (SD = 0), SW continues to be at the top even as the other algorithms improve their scores. This is due to signals like the contracting cosine and the exponential trend, for Fourier-based methods; e.g. Lomb-Scargle and de Lichtenberg. Indeed, for these types of signals the spectral density will not be as concentrated at a single frequency. This, even when there is a clear repeating pattern, which methods like SW and JTK correctly identify.

Classification results deteriorate across the board as noise increases, with DL being the most resilient – specially in high-sampling conditions, and SW performing on par with the others. It is worth noting the similarity in spacing and ordering (with respect to signal shape) of the AUC scores between algorithms. This can be interpreted as follows: for all the evaluated methods classification is more accurate for simpler signals (e.g. cosines and square waves) but as shape patterns become more intricate (e.g. contracting cosine and double peaked) correct classification in the presence of noise is more difficult. Indeed, periodicity (interpreted as the repetition of patterns) is more severely affected in complicated signal shapes when random additive noise increases.

If we now turn our attention to Fig. 3, we see a very similar picture to what we have described so far. That is, even with Laplacian noise, which tends to add more accentuated outliers, the relative performance of the algorithms tends to be similar. This can be interpreted as follows: the algorithms presented here are stable, for the most part, for the noise models under consideration. The exception is PH, as can be seen from the figures.

In summary: For the noise models considered here, SW1PerS is the best performer in the no-noise/all-samplings and small-noise/low-sampling regimes. de Lichtenberg is the most successful in the medium to high noise regime. What we will show next is that SW1PerS has better ranking properties, in that it has a greater richness of signal types at the top of its score distributions.

**Fig. 4:**
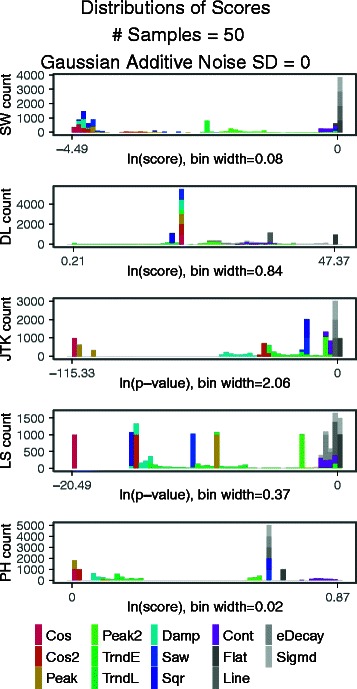
Biases for curve shapes for each algorithm (rows). Distributions of scores are by shape with no noise (Gaussian noise SD=0). The x-axis shows the log of the scores, ranging from the lowest (best score) to the highest (worst score) returned by the algorithm. The y-axis shows the number of signals receiving the score. Please refer to an electronic version for colors

In our second analysis, we examined how biased each algorithm was toward each signal shape. This can be visualized by plotting the distribution, as a histogram, of periodicity scores for all instances of all signal shapes in the synthetic data (Fig. 4). When one shape consistently receives better scores than all others, the algorithm is biased towards this shape. For JTK and LS, we can see a strong bias for cosine signals, which receive the best scores (Fig. 4). DL groups most exemplars at an intermediate level, except for peak2 and contracting signals which receive worse scores, and the trended signals which are distributed across a wide range. For SW1PerS, there is a mixture of cosine, cosine 2, cosine damped, and square signals near the top of the rankings. These are followed closely by peaked and sawtooth signals. The plots of score distributions for each algorithm, number of samples, noise level, and shape can be seen in the supplement (Figures S15-S34). As the noise level increases, these divisions by shape become further blurred. In summary, SW1PerS is the method with the most shape variation for signals scored as highly periodic, and the only one to include damped shapes at the top of its rankings.

Methods such as JTK and LS base their score on p-values. This has a subtle drawback: increasing the number of samples on a periodic curve causes the p-values to become more significant, muddling comparisons across experiments with different numbers of time points. Since SW1PerS ignores the number of samples in its measure of periodicity, it is more amenable to inter-experiment queries.

### Significance Analysis

Using synthetic data we have shown that SW1PerS is a powerful method for quantifying periodicity in time series data. And though the score it produces does not have the subtle drawbacks of methods based on p-values, it is still important to assess its statistical significance.

In what follows we will present a permutation analysis of the SW1PerS score, in order to quantify the probability that observed good scores are due to chance alone. In particular, we compute the empirical probability that a permuted version of a signal gets a better score than the original one. The setup is described below.

For permutation testing we use signals with 25 time points and Gaussian additive noise of 12. One signal was selected for each shape. This set of one signal per shape was then subjected to permutation testing. For permutation testing, each original signal was permuted using python’s **~random.shuffle~** method to create a sample, of size *N*, of permuted versions. This process was repeated *R* times. Each one of the permuted signals, along with the original ones, were then run through SW1PerS. For each sample of size *N*, the p-value was computed as the proportion of permuted signals with SW1PerS score better than or equal to that of the original version.

The number *N* of permuted signals was tested at increasing orders of magnitude: 1000, 10,000, 100,000. The number of repetitions *R* was set to 5. The convergence of the p-values for 5 (=*R*) repetitions and 100,000 (=*N*) permutations was sufficient for analysis. In particular, the standard deviation of the computed p-values for 5 repetitions, across all shapes, was less than 0.0023.

We report in Table 1 the mean p-values, across the 5 repetitions, along with their computed standard deviations for all signal shapes. The low p-values, save for the most challenging signal types, suggests that assigning a good score with SW1PerS by chance alone is highly unlikely. Figure S35 (supplements) depicts histograms of the distributions of scores for the permuted signals.

**Table 1:** Computed mean p-values and standard deviations, across 5 repetitions, for each signal type

Type	Shape	Mean p-value	Std
Periodic	Cos	0.00005	0.000012
	Cos 2	0.003354	0.000313
	Peak	0.010792	0.000363
	Trend Lin	0.009752	0.00035
	Trend Exp	0.161562	0.001052
	Damp	0.006814	0.000177
	Saw	0.00027	0.000035
	Square	0.00001	0.00001
	Contract	0.262642	0.002222
Non-periodic	Flat	0.54663	0.002278
	Line	0.935736	0.001094
	Exp Decay	0.897834	0.000586
	Sigmoid	1	0

### Biological Data Sets

We examined the results of the algorithms on data sets from three microarray experiments (Additional file 2). These experiments were designed to measure periodic gene expression of different processes in different organisms which, as we will show, feature signal shapes which deviate from the usual cosine-like curves.

The wild-type data (WT) from [11] shows periodic gene expression during the cell division cycle (CDC) in budding yeast, *S. cerevisiae*. A population of wild-type cells were synchronized and samples were taken at 16 minute intervals. The period for the cell cycle in this experiment is estimated to be approximately 95 minutes, and the data sets cover a recovery period and roughly two cell cycles. This data set contains 15 samples, but only the last 13 were used in order to omit a stress response. There are two replicates, WT1 and WT2.

The yeast metabolic cycle (YMC) data of [12] are from *S. cerevisiae* that were grown to a high density, briefly starved and then given low concentrations of glucose. Samples were taken at variable intervals of 23-25 minutes. We evened the sample intervals by changing the times to every 24 minutes. The yeast metabolic cycle is estimated to be approximately 300 minutes; this data set covers approximately three cycles and contains 36 samples.

The mammal circadian rhythm data from [13] is from wild-type mice that were synchronized by entraining them to an environment with 12 h light and 12 h dark for one week. They were then placed into total darkness. Samples were taken from the liver every hour. The period of the circadian rhythm is approximately 24 hours, and this data set covers two circadian cycles and contains 48 samples.

For the yeast cell cycle, the data has a low sampling density of 13 samples for two periods (6.5 samples per cycle). Additionally, the data is damped. The yeast metabolic cycle data has a higher sampling density of 36 samples for three periods (12 samples per cycle). For the circadian rhythm, the data has a higher sampling density of 48 samples for two periods (24 samples per cycle) and the data appear noisier than the yeast cell cycle data.

### Biological Data Analysis

Each data set was run through the LS, JTK, DL, and SW algorithms (Parameters in Table S3). We omitted PH from further analysis, as it did not perform as well as the others on the synthetic data. Comparing these algorithms is challenging; unlike in the synthetic data there is no ground truth; the algorithms return p-values or scores that can be difficult to compare directly, and their score distributions are difficult to interpret (Figures S39-S41). We evaluate the performance of SW on biological data, relative to the other algorithms, based on its ability to: find periodic shapes which the other algorithms also identify; find uncommon signals that have nonstandard periodic shapes; and to recover signals of genes that are believed to be part of a given periodic process. In addition, we report sets of genes from overlapping periodic processes found with SW1PerS. We present next the results of these analyses.

#### Finding common periodic signals.

One of the goals in developing SW was that it would be more shape agnostic, and therefore able to detect a larger range of periodic shapes in the data. SW should, however, recover results from the top of the other algorithm’s lists, which have been shown to detect periodic signals.

First, we take the top 10 % and 20 % of results by rank from each algorithm and compare their overlaps (Table 2).

**Table 2:** Percentage of overlap from the top 10 % and 20 % of probes as ranked by the algorithms

Data	Cell Cycle		Met. Cycle		Circ. Rhy.	
Top #	590	1180	933	1866	4510	9020
Top %	10 %	20 %	10 %	20 %	10 %	20 %
SW ∩DL	51 %	59 %	36 %	56 %	64 %	68 %
SW ∩LS	52 %	60 %	67 %	78 %	67 %	59 %
SW ∩JTK	51 %	59 %	60 %	73 %	67 %	66 %
All	26 %	42 %	23 %	42 %	53 %	55 %

Complete Venn diagrams (Figures S42-S44) and tables of percent overlap (Tables S4-S6) can be found in the supplements.

We also quantify the ability of each algorithm to identify genes in sets of *consensus*. For this analysis, a set of consensus is a list of probes which appear in the top 10 % of at least 3 different algorithms. We report in Table 3 the number, and percentage, of consensus genes that each algorithm is able to discover in its top 10 % of rankings. We do this for each one of the biological data sets.

**Table 3:** Number and percentage of probes in the top 10 % of rankings from each algorithm that are in a consensus set. That is, those which appear in the top 10 % of rankings for at least three algorithms

Data Set	Alg	#Consensus	%Consensus
Yeast Cell Cycle	sw	316	0.90
Consensus: 353	dl	289	0.82
	ls	298	0.84
	jtk	311	0.88
Yeast Met. Cycle	sw	553	0.93
Consensus: 596	dl	345	0.58
	ls	563	0.94
	jtk	541	0.91
Mammal Circadian	sw	3090	0.82
Consensus: 3767	dl	3330	0.88
	ls	3640	0.97
	jtk	3636	0.97

As shown, SW has the highest percentage of probes (90 %) in the consensus for the yeast cell cycle. SW has second highest percentage (93 % compared to 94 % for LS) in the yeast metabolic cycle. In the mammal circadian set, SW has 82 % in the consensus set, while the other algorithms have higher percentages (88-97 %). These analyses suggest that SW1PerS is able to identify a large portion of genes labeled as highly periodic, even when the labelling process has been done with very different algorithms.

#### Finding uncommon periodic signals.

**Fig. 5:**
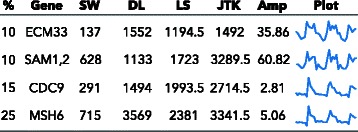
Some example shapes in the yeast metabolic cycle data found in the top 10 % of SW results, but not in the top % listed (first column) of any of the other algorithms. The columns are: gene symbol; rank from SW, DL, LS, and JTK; amplitude; and the plot of the time series. Ties in the rank are averaged. The amplitude is the maximum minimum. The plots are normalized from the minimum to the maximum of the signal. For a full listing of genes in the top 10 % of one algorithm but not in the top 10 % the others, see supplemental files “top_genes”

To determine if SW finds unusual periodic shapes that other algorithms might overlook, we next examine signals ranked highly only by SW. To this end, we study the sets of signals that are in the top 10 % of SW’s rankings, but not in the top 10 % for any other algorithm (Additional file 3). These sets include 151 probes of a total 5900 yeast cell cycle probes on the microarray, 179 of 9335 yeast metabolic probes, 1029 of 45101 mammal circadian probes. The yeast metabolic cycle data, with higher sampling and three cycles, shows interesting examples (Fig. 5).

All signals in this figure are listed in the 3,656 probes (39 % of all probes on the array) identified as periodic in [12]. They use an autocorrelation function with a period determined by Lomb-Scargle. These signals are ranked very highly by SW, are not necessarily highly periodic according to the other algorithms under consideration, and have shapes which are very unusual. Notice that a repetition across three periods makes it highly unlikely for these shapes to be artifacts.

#### Finding signals that are part of a periodic process.

To determine if the algorithms recover genes associated with periodic processes, we examine their rankings of genes associated with the yeast cell cycle and the circadian rhythm. The lists of genes were created from previous studies that locate the binding sites of genes known to be part of the given periodic process.

For the yeast cell cycle, the ChIP-chip data of [14] includes nine known cell cycle transcription factors: Mbp1, Swi4, Swi6, Mcm1, Fkh1, Fkh2, Ndd1, Swi5, and Ace2. From this data set, we selected a list of 141 genes as targets of these transcription factors. For the mouse circadian rhythm, the Chip-Seq data of [15] includes seven known circadian transcription factors: BMAL1, CLOCK, NPAS2, PER1, PER2, CRY1, and CRY2. From this data set, we selected 361 genes as targets of these transcription factors. See Methods for our inclusion criteria.

**Fig. 6:**
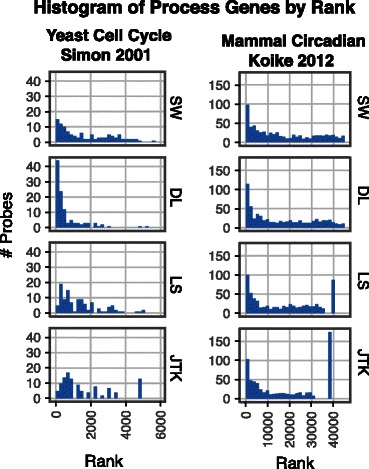
Distribution of genes identified as targets of transcription factors involved in periodic processes. The yeast cell cycle list comes from ChIP-chip experiments and the mammal circadian rhythm data comes from ChIP-Seq experiments

Promoter binding does not guarantee functional regulation and therefore some targets may not be periodically expressed in response to binding by a cell cycle or circadian rhythm transcription factor. However, for including genes in our periodic process list, we are willing to accept the cost of including false positives in exchange for the benefit of not using other periodicity detection methods to determine inclusion. The rankings of these genes are visualized with a histogram to show how periodic the algorithms consider them (Fig. 6).

For the yeast cell cycle, SW and DL pick the highest number of binding targets at the top of their rankings, with DL selecting the most. Within the top 10 % of the rankings, DL finds 53 % of the genes, SW 24 %, LS 22 %, and JTK 19 %. For the mammal circadian data, the distributions for the top rankings are more similar; SW and the other algorithms find 50-55 % of the gene list within the top 10 % of their rankings. We find that these results are relatively stable across different cutoffs, as shown in Tables 4 and 5.

**Table 4:** Overlap between algorithm rankings and binding data for Yeast Cell Cycle data. The percent of probes in the top X % of rankings for each algorithm that are in the set of bindings targets that we compiled from the ChIP-chip data of Simon, et al, 2001

Alg	5 %	10 %	15 %	20 %
SW rank	14	24	31	35
DL rank	39	53	56	59
LS rank	9	22	35	38
JTK rank	10	19	31	40

**Table 5:** Overlap between algorithm rankings and binding data for Mammal Circadian data. The percent of probes in the top X % of rankings for each algorithm that are in the set of bindings targets that we compiled from the ChIP-Seq data of Koike, et al, 2012. Note that the array for the circadian data set has multiple probes for some genes and duplicates were not removed

Alg	5 %	10 %	15 %	20 %
SW rank	32	50	63	74
DL rank	41	54	69	70
LS rank	37	53	65	71
JTK rank	35	55	70	78

In contrast with SW, LS and JTK have pushed a larger portions of these genes to the lowest rank. See supplement for a comparison of rankings for yeast cell cycle (Figure S45) and mammal circadian rhythm (Figure S46) for a selected set of known genes.

#### Discovery of signals from multiple processes.

To determine if SW finds genes involved in multiple processes in budding yeast, we compared cell cycle genes preferred by SW with results from other experiments in yeast. To create the list of genes that SW prefers, we selected the top 10 % of ranked results from SW that were not in the top 10 % of ranked results for the yeast cell cycle (WT1) on either DL, JTK or LS. This results in 151 probes with 148 unique systematic names. To filter out probes that are potentially more noise than signal, we compared the replicates WT1 and WT2 using a combined score from SW and JTK (see Supplements, section 8). A cutoff of 0.05 yielded a list of 77 probes.

We compared this set of signals to lists of genes from other experiments: the gene list of the yeast metabolic cycle (YMC) from [12] and the gene lists of the positive and negative universal growth rate response (GRR) from [16]. The genes with universal growth rate response are a subset of the periodically expressed genes in the yeast metabolic cycle [16]. The percent overlap between our list of 77 probes and these data sets are shown in Table 6. (Supplemental figures S47-S49.)

**Table 6:** Number of probes from the top 10 % of SW, not in the top 10 % of other algorithms, filtered for noise using the replicates, that overlap with other data sets. We also show the numbers of these probes not identified in Orlando 2008, Spellman 1998, and not in either of these data sets

Dataset	Overlap	¬Orlando	¬Spellman	¬Either
YMC	36 (47 %)	21 (27 %)	30 (39 %)	18 (23 %)
GRR Pos	3 (4 %)	2 (3 %)	3 (4 %)	2 (3 %)
GRR Neg	13 (17 %)	8 (10 %)	12 (16 %)	8 (10 %)

**Fig. 7:**
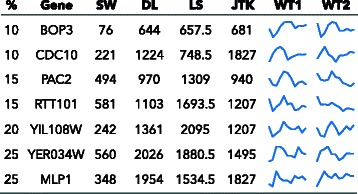
Examples of genes from the yeast cell cycle data in the top 10 % of SW, not in the top % listed (first column) of other algorithms, filtered for noise, that overlap with other data sets and have not been identified in [11] and/or [18]. Columns are gene symbols; the rank out of 5,900 probes for each method; and the plot of the wild-type replicates 1 and 2. Ties in rank are averaged. The plots are normalized from the minimum to the maximum. For a full listing of genes in the top 10 % of one algorithm but not in the top 10 % the others, see supplemental files “top_genes”

In the overlap with the yeast metabolic data, but not previously identified as periodic in [11], was CDC10, a component of the septin ring [17]. Not identified in [18] or [11] were BOP3, a potential target of Cdk1 [19]; and YIL108W and YER034W, which are involved in the response to DNA replication stress [20]. In the overlap with the negative universal growth rate response, but not previously identified as periodic in [11] or [18], were MLP1, which has a role in controlling the length of telomeres [21]; PAC2, which is involved in microtubule functioning and chromosome segregation [22]; and RTT101, which is involved in the progression of anaphase [23]. The algorithm rankings and time series for these genes are shown in Fig. 7.

## Discussion

The results from the synthetic data show that SW is comparable to other popular algorithms for most signal shapes, noise levels, and sampling densities. Additionally, SW outperforms DL, LS, JTK, and PH on the low noise regime and across all sampling densities. This analysis has shown that SW1PerS performs well on data that has shapes which occur in biological systems from different organisms, and that it is well behaved under sampling densities and noise levels found in microarray data sets. SW1PerS shows less bias against damped signals, which occur frequently for instance in the yeast cell cycle data.

The analysis of the biological data shows that SW1PerS is able to recover many of the signals other algorithms find, and can additionally discover non-cosine shapes that other algorithms might exclude. We believe that finding signals with a greater diversity of shapes well outweighs the cost of giving higher ranks to signals that might appear to be noise. SW also appears to detect different types of biological processes than the other algorithms based on GO enrichment (see Supplemental table S7-S14).

Each algorithm tested here has strengths and weaknesses that vary by signal shapes, noise levels, and sampling rates. For SW1PerS, in particular, we have observed the following relative **strengths:**

- SW1PerS, in the low noise range, has been shown to be the most shape-agnostic algorithm out of the methods studied here.
- SW1PerS is able to effectively estimate periodicity even as the period length changes from oscillation to oscillation. We saw this, for instance, with the contracting cosine in the synthetic data.
- The score that SW1PerS returns has a geometric interpretation, and can be compared across different data sets.
- SW1PerS can be used on data with low temporal resolution and uneven time spacing.
- While the algorithm requires the selection of certain parameters (e.g. window size, embedding dimension, etc), the theory behind the method suggests reasonable values.
- Even though the inner-workings of the SW1PerS algorithm are quite different from the other methods studied here, it is able to recover – to a large extent – what other algorithms find.

There are also **weaknesses** which are worth keeping in mind:

- The implementation we have of SW1PerS has been clocked at between 0.5sec and 1.0sec per signal, on a laptop computer. Hence, running-time can be an issue. We expect that as better algorithms for computing 1-persistent homology and more computational resources become available, this problem can be mitigated.
- The probability distribution for the SW1PerS score, even for the additive Gaussian noise model, has not been described as of yet. Hence, we lack a principled way of producing *p*-values. And though studying this distribution is out of the scope of the present article, we have used synthetic data – where the ground truth is known – to assess the performance of SW1PerS relative to other algorithms. In addition, permutation tests were also performed to evaluate significance and positive results were obtained as shown in Table 1.
- As we have observed with the synthetic data, SW1PerS tends to degrade as noise increases, and it does so at a faster rate than some of the other methods studied here. Signal processing, however, is a rich field with highly successful denoising algorithms that can be brought to bear in this problem.
- SW1PerS does not recover the phase or period length.

Keeping all this in mind, the analyses presented here have shown the benefits of applying SW1PerS, especially in exploratory situations where signal shapes might not be known and a broad set of candidates is desirable.

## Conclusions

We have presented in this paper a new algorithm, SW1PerS, for quantifying periodicity in time series data. The algorithm has been extensively tested and compared to other popular methods in the literature, using both synthetic and biological data. Specifically, with a vast synthetic data set spanning 14 different signal types (10 periodic and 4 non-periodic), 4 noise models, 5 noise levels and 3 sampling densities, it was shown that SW1PerS outperforms the other algorithms presented here in the low-noise and low-sampling regimes. Moreover, it exhibits at the top of its rankings the most variety in signal types, making it the most shape-agnostic and the only one to identify damped signals as highly periodic. In the biological data SW1PerS recovers, to a large extent, what other algorithms have identified in previous work. Moreover, it was also able to discover signals with interesting shapes, which were overlooked by the other methods.

By using SW1PerS along with other algorithms that complement its strengths and lessen its weaknesses, it can be used as a powerful tool in exploratory analyses. Indeed, in biological systems with low noise, i.e. where periodic signals with interesting shapes are more likely to occur, SW1PerS can be used to identify an initial set of periodic genes with a rich variety of signal types. Patterns and shape information can then be included in the study of systems, as well as in the generation of hypotheses regarding the structure of gene regulatory networks.

## Methods

### The SW1PerS Algorithm

The way SW1PerS recognizes periodicity is simple: It measures the existence of a distinctive pattern in the graph of the signal, and quantifies the extent to which it repeats. The quantification step, in contrast with other methods, does not involve the usual measures of correlation. Instead we use tools from **topological data analysis** [24], a new set of techniques that probe/quantify the shape of data, to measure the circularity of a point cloud derived from the time series.

**Fig. 8:**
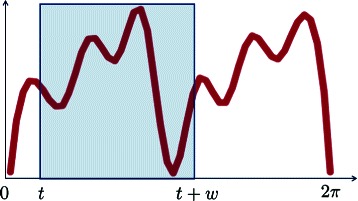
*g* along with a window of length *w* starting at *t*∈[0,2*π*−*w*]

More specifically, given a time series *g*_0_,*g*_1_,…,*g*_*S*_ (e.g. of gene expression data) measured at times *t*_0_,*t*_1_,…,*t*_*S*_, we map the interval [*t*_0_,*t*_*S*_] linearly onto [0,2*π*] and apply cubic splining to obtain a continuous function $g:[0,2\pi ] \longrightarrow \mathbb {R}$ so that *g*(0)=*g*_0_ and *g*(2*π*)=*g*_*S*_. For a fixed 0<*w*<2*π*, referred to as the **window size**, and each time *t*∈[0,2*π*−*w*] we consider the graph of *g* restricted to the interval [*t*,*t*+*w*]. Let us use Fig. 8, where we depict a prototypical function *g* with a window of length *w*, as a running example.

**Fig. 9:**
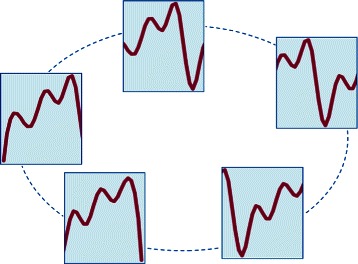
Arrangement of snippets according to similarity

Sliding this window (the first two words in the acronym SW1PerS) corresponds to letting *t* vary from 0 to 2*π*−*w*, and it follows that each *t*∈[0,2*π*−*w*] yields a snapshot, or snippet, from *g*. If these snippets were arranged according to their degree of similarity, similar snippets being closer, then the emerging picture would be analogous to that in Fig. 9.

The repetition of a pattern in the graph of *g* is thus associated with the circular arrangement of the snippets, while its distinctiveness corresponds to the size of the “hole” in the middle of the arrangement. Notice that the term “pattern” applies to any type of snippet; this is what gives SW1PerS its shape-agnostic nature.

We formalize this construction as follows: let *M* be a positive integer (usually larger than twice the number of time points) and set $\tau = \frac {w}{M}$ for some window size *w*∈(0,2*π*). The theory behind SW1PerS [25] implies that a good window size should be close to $\frac {2\pi M}{L(M+1)}$, where *L* is the number of expected periods.

The discretized sliding window starting at *t* is given by the vector

$${SW}_{M,\tau} g(t) =\left[ \begin{array}{c} g(t)\\ g(t + \tau)\\ \vdots\\ g(t+M\tau) \end{array} \right] $$

As we let *t* take values in a (sufficiently dense) finite set^1^ *T*⊂[0,2*π*−*w*], the result is a collection of points $X\subset \mathbb {R}^{M+1}$ which we refer to as a **sliding window point cloud**. In this cloud, viewed as a subset of $\mathbb {R}^{M+1}$, two points are close if and only if the corresponding snippets they discretize are similar. It follows that the extent to which *X* can be thought of as sampled from a closed curve without self-intersections (a topological circle), is in direct correspondence with the periodicity of *g* as measured with windows of size *w*. The resulting point cloud is then pointwise mean-centered and normalized; that is, we replace *X* by

$$\bar{X} =\left\{ \begin{array}{lll} \frac{\mathbf{x} - mean(\mathbf{x})}{\|\mathbf{x} - mean(\mathbf{x})\|} & : & \mathbf{x}\in X \end{array} \right\} $$

This ameliorates the effects of damping and trending in the original time series, and also makes SW1PerS amplitude blind.

We input $\bar {X}$ into the **1-Persistent Homology** algorithm [26] using a fast implementation tailored specifically for sliding window point-clouds (see supplements, Section 2). From the 1-Persistent Homology computation one can extract two numbers: $0 \leq b \leq d \leq \sqrt {3}$, where *b* measures the maximum distance from a point $\bar {\mathbf {x}}\in \bar {X}$ to its nearest neighbor in $\bar {X}$, and *d* measures both how circular and wide $\bar {X}$ is. This is what we alluded to as measuring the shape of data and the size of the hole in Fig. 9. The pair (*b*,*d*) yields a score

$$s(n,m)= 1 - \frac{d^{n} - b^{m}}{3^{n/2}} $$

between 0 (periodic) and 1 (not periodic) for each choice of integers *n*≥*m*. For this paper we use *n*=*m*=2, as this pair yielded the best results on the synthetic data. We refer the reader to [25] and Section 1 of the supplements for a more detailed discussion on the choice of the parameters *M*,*w*, the set *T*⊂[0,2*π*−*w*], and the mathematical underpinnings of the method.

### Dealing with Noise

We present two denoising paradigms included in the SW1PerS pipeline; the first operates on time series, and the second focuses on noise at the point-cloud level.

#### Simple Moving Average

Can be interpreted as a discrete version of convolution with a step function. The input for this method is an odd integer 2*k*+1, much smaller than the number of observations in the time series *g*_0_,…,*g*_*S*_. The result, a locally averaged time series $\tilde {g}_{0},\ldots, \tilde {g}_{S}$, is obtained as follows: for each *s*=0,…,*S* we let *ℓ*= min{*s*, *S*−*s*, *k*} and define

$$\tilde{g}_{s} := \frac{g_{s-\ell} + \cdots + g_{s} + \cdots + g_{s+\ell}}{2\ell + 1} $$

Simple Moving Average often yields satisfactory results given its local nature, and that it can be applied to time series with low time resolution (*S*≥13). A limitation, however, is that it can remove fine features and peak-like behavior. Thus, we restrict *k* to values so that *g*_*s*−*k*_,…,*g*_*s*_,…,*g*_*s*+*k*_ does not span more than a third of the window size *w*.

#### Mean-Shift

Has appeared numerous times in the statistics literature, and more recently in the work of [27]. It can be seen as a point-cloud-level version of moving average, in which each point of the cloud is replaced by the average of those close to it. Intuitively, this has a tightening effect. Closeness to a point can be defined as being among its *q*-th nearest neighbors for some integer *q*, or by being no farther than *ε* away for some constant *ε*>0. It is the second option we use in this paper. Since in SW1PerS the sliding window point cloud has been pointwise mean-centered and normalized, it follows that it lies on the surface of the unit sphere in $\mathbb {R}^{M+1}$. Hence we measure distance between two such points **x**,**y** via the angle between them and deem them to be close^2^ if $\measuredangle (\mathbf {x},\mathbf {y}) < \frac {\pi }{16}$. Once each point has been replaced by the average of those no more than $\frac {\pi }{16}$ away, we proceed to pointwise normalizing the resulting cloud.

## Availability and Supporting Data

An implementation of SW1PerS can be found at http://cms.math.duke.edu/harer/?q=downloads.

## Endnotes

^1^ In practice we use $T= \{\frac {j(2\pi -w)}{200} | j = 0, 1, \ldots,$

^2^ This constant was set experimentally based on performance on the synthetic data.

## Additional files

Additional file 1
**Supplements.** The supplements file contains detailed information on several points discussed in this paper. In particular: 1. The mathematics behind the SW1PerS algorithm, 2. A detailed description of the fast 1-Persistent Homology algorithm, 3. Generating functions for the synthetic data, 4. All ROC plots from the synthetic data analysis, 5. All score distributions from the synthetic data analysis, 6. Histograms of score distributions for permutation test,7. Details regarding the availability and processing of the biological data, 8. Gene lists from ChIP-chip and ChIP-seq data, 9. The method used for filtering noise using replicates and 10. GO Enrichment analysis.

Additional file 2
**Rankings.** These three files contain the biological data sets used in this paper, as well as the scores and rankings from all the algorithms presented here. In addition, they include filters so that users can re-order the probes by the score of a particular algorithm.

Additional file 3
**Top genes.** This zip file contains three pdf files, associated to each one of the 3 biological data sets studied in this paper. Each file shows the full ordered list, sparkLines included, of genes in the top 10 % of rankings according to SW1PerS and that are not present in the top 10 % of the other algorithms.
