# Supplementary material for: SW1PerS: Sliding windows and 1-persistence scoring; discovering periodicity in gene expression time series data
Source: BMC Bioinformatics. 2015 Aug 16;16:257. doi: 10.1186/s12859-015-0645-6 (PMC4537550; doi:10.1186/s12859-015-0645-6)
Supplement: Additional file 1 — Supplements. The supplements file contains detailed information on several points discussed in this paper. In particular: 1. The mathematics behind the SW1PerS algorithm, 2. A detailed description of the fast 1-Persistent Homology algorithm, 3. Generating functions for the synthetic data, 4. All ROC plots from the synthetic data analysis, 5. All score distributions from the synthetic data analysis, 6. Histograms of score distributions for permutation test,7. Details regarding the availability and processing of the biological data, 8. Gene lists from ChIP-chip and ChIP-seq data, 9. The method used for filtering noise using replicates and 10. GO Enrichment analysis. [file 12859_2015_645_MOESM1_ESM.pdf]

SUPPLEMENTARY INFORMATION
SW1PerS: Sliding Windows and 1-Persistence Scoring; Discovering Periodicity in Gene Expression Time Series Data

Jose A. Perea[1,2]*, Anastasia Deckard[3]*,

Steven B. Haase[4], John Harer[1,3]

[1]Department of Mathematics, [2] Institute for Mathematics and its Applications, University of Minnesota, Minneapolis, MN, USA [3]Program in Computational Biology and Bioinformatics, [4]Department of Biology, Duke University, Durham, NC, USA

Abstract

Background Identifying periodically expressed genes across different processes (e.g. the cell and metabolic cycles, circadian rhythms, etc) is a central problem in computational biology. Biological time series may contain (multiple) unknown signal shapes of systemic relevance, imperfections like noise, damping, and trending, or limited sampling density. While there exist methods for detecting periodicity, their design biases (e.g. toward a specific signal shape) can limit their applicability in one or more of these situations.

Methods We present in this paper a novel method, **SW1PerS**, for quantifying periodicity in time series in a shape-agnostic manner and with resistance to damping. The measurement is performed directly, without presupposing a particular pattern, by evaluating the circularity of a high-dimensional representation of the signal. SW1PerS is compared to other algorithms using synthetic data and performance is quantified under varying noise levels, sampling densities, and signal shapes. Results on biological data are also analyzed and compared.

Results On the task of periodic/not-periodic classification, using synthetic data, SW1PerS outperforms all other algorithms in the low-noise regime. SW1PerS is shown to be the most shape-agnostic of the evaluated methods, and the only one to consistently classify damped signals as highly periodic. On biological data, and for several experiments, the lists of top 10% genes ranked with SW1PerS recover up to 67% of those generated with other popular algorithms. Moreover, the list of genes from data on the Yeast metabolic cycle which are highly-ranked only by SW1PerS, contains evidently non-cosine patterns (e.g. ECM33, CDC9, SAM1,2 and MSH6) with highly periodic expression profiles. In data from the Yeast cell cycle SW1PerS identifies genes not preferred by other algorithms, hence not previously reported as periodic, but found in other experiments such as the universal growth rate response of Slavov. These genes are BOP3, CDC10, YIL108W, YER034W, MLP1, PAC2 and RTT101.

Conclusions In biological systems with low noise, i.e. where periodic signals with interesting shapes are more likely to occur, SW1PerS can be used as a powerful tool in exploratory analyses. Indeed, by having an initial set of periodic genes with a rich variety of signal types, pattern/shape information can be included in the study of systems and generation of gene regulatory networks.

Availability http://cms.math.duke.edu/harer/?q=downloads

Contact Jose A. Perea joperea@math.duke.edu, Anastasia Deckard anastasia.deckard@duke.edu

*To whom correspondence should be addressed

Contents

1 Persistent Homology of Sliding Window Point-Clouds 2
1.1 The SW1PerS Pipeline . . . 4

2 Computing 1-Persistent Homology 5
2.1 Algorithm . . . 5
2.2 Running Time . . . 6

3 Synthetic Data: Generating Functions 7

4 Synthetic Data: ROC Plots 9

5 Synthetic Data: Score Distributions 22

6 Biological Data 44

7 Gene lists from ChIP-chip and ChIP-seq Data 58

8 Filtering Noise using Replicates 60

9 GO Enrichment Analysis 64
9.1 SW1PerS . . . 64
9.2 DL . . . 65
9.3 JTK . . . 67
9.4 LS . . . 68

1 Persistent Homology of Sliding Window Point-Clouds

The problem of estimating topological properties from a geometric object $\mathbb{X}$ given a finite sample X, has received wide attention in the computational topology literature. One of the most successful strategies emerging from these studies is the application of persistent homology to point clouds.

Intuitively, homology is an algebraic way of measuring shape invariants of continuous spaces; these invariants include number of connected components, holes, voids, and their higher dimensional analogs. **Persistent homology**, in turn, is an adaptation of these continuous invariants to discrete sets of points. Let us illustrate how it works. If we consider, for instance, the sample in figure S1 then it is apparent it evokes an ellipsoidal shape.

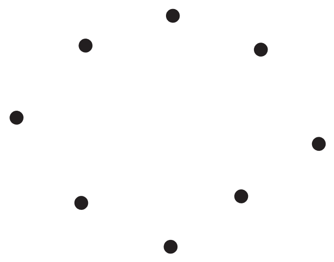

Figure S1: Eight points on the plane, sampled with noise from an ellipse.

The way we capture this pattern is by fattening the points in order to blur the gaps between them. Formally, we fix a real number $\epsilon > 0$ and consider the region covered by disks[1] of radius ϵ centered at the sample points. The resulting regions for two choices of ϵ can be seen in figure S2. The one pictured on the right has what is called a nontrivial 1-homological feature: for the larger radius it is possible to draw a closed curve lying entirely inside the disks, so that one cannot continuously deform (shrink) it to a point without either tearing the curve or leaving

[1]For point clouds in $\mathbb{R}^n$ one uses closed balls $\overline{B}_\epsilon(\mathbf{x}) = \{\mathbf{y} \in \mathbb{R}^n : \|\mathbf{x} - \mathbf{y}\| \leq \epsilon\}$.

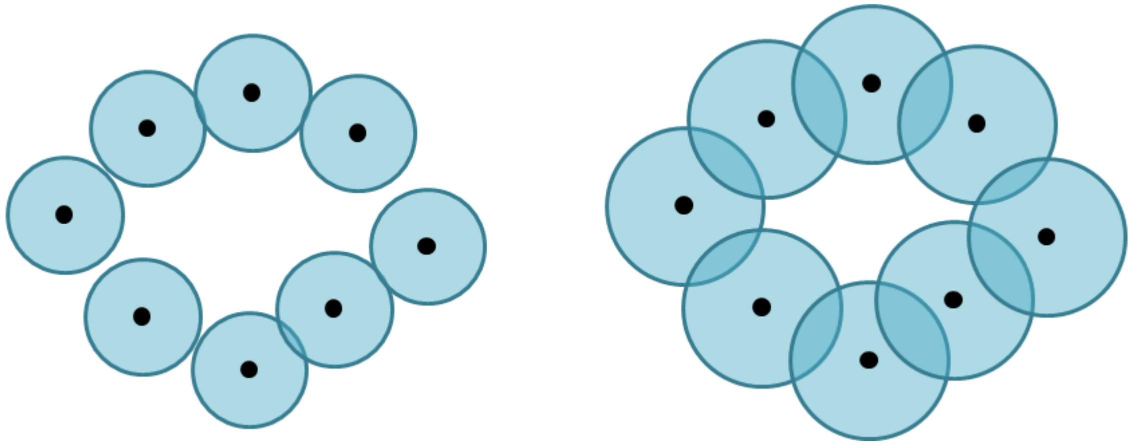

Figure S2: Coverings with disks of radius ϵ (left) and ϵ' (right), $\epsilon < \epsilon'$, centered at the sample points.

the region. The covering on the left, on the other hand, does not have this property. Hence there exists a choice of radius, between ϵ and ϵ', where the nontrivial 1-homological feature appears for the first time. We denote this value by b and call it the feature's **birth time**. Moreover, if we continue to increase the radius, then eventually the union of disks will cover the "hole" in the middle making the 1-homology feature trivial. That is, it will be possible to continuously shrink the aforementioned curve to a point while staying inside the covered region. Let d be the radius where this first occurs, and let us refer to it as the feature's **death time**. The **persistence** of a homological feature, defined as $d - b$, measures both its prominence and the level of confidence on whether it captures a topological property of the underlying continuous space. For a general sliding window point cloud $X \subset \mathbb{R}^{M+1}$ there will be several 1-homological features, corresponding to the different holes, being born and dying at different times. We compute their persistence and use the largest as a measure of circularity of X, and hence of periodicity of f.

One characteristic of 1-dimensional persistent homology, as described here, is that it measures not only the circularity of a point cloud but also its roundness. For instance, dominant 1-homological features from (planar) ellipsoidal shapes have smaller persistence than that of a circular pattern, when the length of the underlying curve is kept constant. This is the case because for ellipses largest persistence is associated with minor axes, while for circles it is a measure of diameter. As described in (Perea and Harer, 2014), the choice of window size w provides a way of accounting for this bias: when w approaches the length of the period, the sliding window point cloud is as round as it can be. In particular, if f satisfies the identity $f\left(t + \frac{2\pi}{L}\right) = f(t)$ for some $L \in \mathbb{N}$ and all t, i.e. f is L-**periodic**, then its L-periodicity is best captured by its sliding window point cloud when

$$w = \left(\frac{M}{M+1}\right)\frac{2\pi}{L} \tag{1}$$

The embedding dimension $M+1$, on the other hand, determines the accuracy with which the snippets from f are captured by their discretizations. Indeed, complicated patterns require larger embedding dimensions in order to be faithfully represented. The Shannon-Nyquist sampling theorem (Shannon, 1949) provides a guide for making this choice. Let us assume we have a time series $f(t_1), \ldots, f(t_S)$ from evaluating f at the evenly spaced time points $0 \leq t_1 < t_2 < \cdots < t_S \leq 2\pi$. It follows that if f is L-periodic and the window size is chosen as in equation 1, then within any window of such length there should be around $\frac{S}{L}$ observations. The Shannon-Nyquist sampling theorem implies that with this number of time points one can be sure to capture at most $\frac{1}{2}\left(\frac{S}{L} - 1\right)$ harmonics from the windowed snippet. Combining this with the Structure Theorems from (Perea and Harer, 2014), we get that choosing

$$M + 1 \geq \frac{S}{L} \tag{2}$$

guarantees that there is no loss of information in going from the Whittaker-Shannon reconstruction of f, to the sliding window point cloud. In practice we use this number only as a guide and combine it with knowledge on how intricate the patterns might be. Indeed, cosine-like patterns only require three points per period in order

to be accurately described, even if the signal has been sampled at a higher temporal resolution. For the results obtained in this paper we use $M = 14$ to study the periodicity of times series from signals with various shapes, $L = 2, 3$ and sample size $S = 13, 17, 25, 42, 50$.

Let us now examine the incidence of the finite set $T \subset [0, 2\pi - w]$ used to populate the sliding window point cloud. The persistence $(= death - birth)$ of a homological feature is prominent when one has both a large death time, and a prompt birth. Death times are mostly about the diameter of the homological feature, while births are intimately related to how densely the point cloud has been populated. We say that T is δ-dense in $[0, 2\pi - w]$, $\delta > 0$, if for each $x \in [0, 2\pi - w]$ there exists $t_x \in T$ so that $|x - t_x| < \delta$. The main point is that, with some work, one can show that the birth time of the prominent 1-homological feature of a sliding window point cloud from an L-periodic function, $L \geq 2$, populated with a δ-dense set T, is no larger than δM. For the results presented in this paper we let T be the set consisting of 200 points evenly spaced between 0 and $2\pi - w$, for w as in equation 1. That is, for $M = 14$ and $L \geq 2$ we expect the birth time to be smaller than or equal to 0.1173.

1.1 The SW1PerS Pipeline

Input:

- Observations $f_1, f_2, \ldots, f_S$ sampled at times $0 = t_1 < t_2 < \cdots < t_S = 2\pi$. The sampling is not required to be at equally spaced time points.
- Targeted L-periodicity supplied as a number $L \geq 2$; SW1PerS will score how L-periodic the time series is. L should be the number of periods in the time series, but it does not need to be an integer. This is specially useful when the periods have different durations; in this case one should choose L so that the window size (equation 1) is that of the shortest.

Output:

- L-periodicity score. This is a number between 0 and 1, with 0 being perfectly L-periodic.

The Pipeline

1. *Preprocess/Denoise time series data.* This is an optional step in which we apply Simple Moving Average.
2. *Populate sliding window point cloud.* We recover a continuous sampling function $f : [0, 2\pi] \longrightarrow \mathbb{R}$ by cubic splining the (denoised) time series. This allows us to deal with uneven sampling, and low temporal resolution. Given the finite set $T \subset [0, 2\pi - w]$ we populate the sliding point cloud X by evaluating $SW_{M,\tau} f$ at each $t \in T$.
3. *Postprocess/denoise point cloud.* Here we pointwise mean-center and normalize the sliding window point cloud $X \subset \mathbb{R}^{M+1}$. That is, for each $\mathbf{x} \in X$ we let
$$\overline{\mathbf{x}} = \frac{\mathbf{x} - mean(\mathbf{x})}{\|\mathbf{x} - mean(\mathbf{x})\|}$$
where $mean(\mathbf{x})$ is the constant $(M + 1)$-tuple having entries equal to the average of the ones in $\mathbf{x}$. Mean-centering each snippet has the effect of making the L-periodicity score less sensitive to signal **trending**. Normalizing each window, on the other hand, makes SW1PerS amplitude blind and provides a way of dealing with signal **dampening**. We let $\overline{X}$ denote the resulting pointwise mean-centered and normalized sliding window point cloud. In the event that the periodic pattern presents noticeable variations across the signal, e.g. due to noise, it is sometimes helpful to apply cloud-level denoising in the form of Mean-Shift (Comaniciu and Meer, 2002). This has the effect of making the circular pattern close up properly for $L = 2$, and of preventing the point cloud from spiraling in/out when $L \geq 3$.
4. *Calculate score.* Let $mp(\overline{X})$ denote the persistence of the dominant 1-homological feature underlying $\overline{X}$, i.e. the one with largest persistence, and let (b, d) be a pair attaining it. For this we use the fast implementation described in the next section. We let the L-periodicity score be
$$score = 1 - \frac{d^n - b^m}{3^{n/2}} \tag{3}$$

for some choice of positive integers $n \geq m$ (in this paper we let $n = m = 2$). Notice that each choice yields a way of measuring periodicity, and that for $n = m$ and a planar circle of radius 1, as the sampling density goes to infinity mp is exactly[2] $\sqrt{3}$.

2 Computing 1-Persistent Homology

2.1 Algorithm

The method described in this paper requires the computation of the 1-dimensional persistence pairing of the filtered Rips complex of a point cloud. The standard method for doing this can be slow if the point cloud is large, so we implemented an improvement that reduces the time significantly. We learned the basics of this method from Vidit Nanda, who has also implemented a version in his software package *Perseus* (Mischaikow and Nanda, 2013). Our version is different in that we never build the full Rips complex, instead we reduce the complex as we go along, resulting in less storage and, we believe, a faster process. In any case, the key idea is a combinatorial version of Morse Theory due to Forman (1998).

Start with a point cloud $X \subset \mathbb{R}^n$, and a maximum distance $dMax > 0$. In the SW1PerS method, the point cloud is given by first filtering a time series, then using the sliding window to create a point cloud, and then possibly doing a second filtering of the cloud itself to obtain X. The first filtering is typically based on the kind of data that one has. We use mean-shift-iteration (Comaniciu and Meer, 2002) for the second filtering, as it tightens up the circular shape of the point cloud when it is there without creating one when it is not. The maximum distance $dMax$ limits the length of edges that we consider in order to keep the analysis manageable. The rest of our description concerns only the computation of 1-dimensional persistence.

Data Structures: We maintain $\mathcal{E}$, a vector of edge classes and $\mathcal{L}$, a vector of lists of the vertices in the lower link of each vertex. (The lower link is the portion of the link of each vertex that has been constructed up to the current stage of the algorithm.) The lists in $\mathcal{L}$ are kept sorted. Each edge instance from $\mathcal{E}$ maintains a label as positive or negative (all are initially labeled positive). We also maintain Γ, a directed acyclic graph (DAG) whose nodes are the positive edges and whose edges will be described below. The DAG Γ is used to construct the persistence matrix $M_{1,2}$ (as a vector of lists) which gets reduced at the end to compute persistence.

Step 1 - Set Up We read in the point cloud X, and compute the pairwise distance matrix $\mathcal{M}$. Each pair of vertices gives an edge whose length is the corresponding entry of $\mathcal{M}$. To manage the data, we only store those edges whose length is at most $dMax$. Finally, we sort these edges by their length.

Step 2 - Union Find We make a first pass through the edges of $\mathcal{E}$ applying the union-find algorithm of Tarjan (1975) to determine if each edge reduces the number of components of the growing complex or not. This continues until all edges are considered or the number of components reduces to 1. We change an edge label to negative if it reduces the number of components in this process. At the end all edges are correctly labeled negative or positive, where negative edges are the ones that reduce the number of components, and positive ones create a loop or 1-*cycle*.

Main Step The main part of the algorithm makes a second pass through the edges of $\mathcal{E}$ and builds the matrix $M_{1,2}$.

Consider the next edge e from $\mathcal{E}$. Let the vertices of e be v_0 and v_1.
If the edge e is positive, do the following steps:

- Compute the intersection I of the local lists $\mathcal{L}[v_0]$ and $\mathcal{L}[v_1]$; I is the set of vertices that lie in the lower link of e. Since the lists of $\mathcal{L}$ are kept sorted, this is done in linear time with a mod 2 merge.
- If I is empty, add a row to the matrix $M_{1,2}$ and add a new *leaf vertex* to Γ. We then skip the next steps in the bulleted list.

[2] This is the answer when using the Rips complex, which is how SW1PerS is implemented.

- If I is non-empty, compute the edges already added between the vertices in I. Do Union-Find on I using these edges, and retain a representative of each component. This gives a list $\{u_0, \ldots, u_k\}$ of representatives for the components of the lower link of e.
  - Process the vertex u_0, which we think of as paired with e. Let $e_0 = v_0 * u_0$ and $e_1 = v_1 * u_0$ be the other two edges of the triangle spanned by e and u_0.
    - If e_0 and e_1 are both negative, then we switch e to negative and DO NOT add a new vertex to Γ.
    - If either e_0, e_1, or both are positive, we add a vertex (not a leaf vertex) to Γ and a edge to Γ from e to e_i, if e_i is positive, $i = 0, 1$. Note that the number of outgoing edges from this new vertex is either 1 or 2.
  - Next we process the other vertices of I. Each u_j with $0 < j \leq k$ creates a triangle $e * u_j$ and will give a new column to $M_{1,2}$ corresponding to its union with the triangle $e * u_0$. The edges of $e * u_j$ are e, $v_0 * u_j$ and $v_1 * u_j$. For each of these which is positive we search Γ down to its leaf vertices to obtain its base list. Each path to a leaf adds that vertex, but counting is modulo 2 so an even number of paths means the leaf is not included and an odd number means it is. The resulting list gives the new column of $M_{1,2}$. (An alternative to using the DAG Γ is to maintain a list of "below" edges with each positive edge, and to pass these lists up in the u_0 processing step, with the usual mod 2 merge.)

Now, whether e is negative or positive, we add v_0 to $\mathcal{L}[v_1]$ and v_0 to $\mathcal{L}[v_1]$. These are inserted in their correct place so that the lists of $\mathcal{L}$ remain sorted, which takes linear time.

Reduction Step The process ends by reducing the matrix $M_{1,2}$ to obtain a matrix $\hat{M}_{1,2}$, (reduction is described in Edelsbrunner and Harer (2010)). The 0-D persistence diagram has a point for each *negative* edge e with coordinates $(0, l(e))$, where l denotes length. The 1-D diagram has a point with coordinates (b, d) for each column c of the matrix $\hat{M}_{1,2}$ that consists only of 0's. Here the birth time b is the length of the edge e associated to c. To find d, look at the row corresponding to e and find it's first non-zero entry. The death time d is then the length of the edge associated to the column that contains that entry. If no such row exists, we set $d = \infty$.

2.2 Running Time

Let n be the number of points in X. It takes $\mathcal{O}(n^2)$ time to compute the pairwise distances. The number of edges is nk, where k is the expected number of edges at each vertex when lengths are limited to $dMax$, and the amount of time it takes to sort is thus $\mathcal{O}(nklog(nk)) = \mathcal{O}(nklog(n))$ since $k < n$.

The union find algorithm runs in time $\mathcal{O}(nk + n\alpha(n))$, where α is a very slow growing function that is essentially less than 4. Thus the time is dominated by the fact that we may have to consider every edge to get connectivity, which is the nk term.

Inserting a new entry into $\mathcal{L}$ takes linear time in it's length, so the total insertion time is $\mathcal{O}(n^2k)$.

Let a_0 be the expected number of positive edges whose lower link in empty, and more generally let a_k be the expected number of positive edges whose lower link has k components. Set $m = \sum_k (k-1)a_k$. Since every triangle gets added with it's longest edge, m is the number of triangles in $R_{dMax}(X)$. The matrix $M_{1,2}$ has a_0 rows and m columns. Note that $a_0 + a_1 + \ldots = nk$, the number of edges in $\mathcal{E}$ and m

Searching Γ takes time that i If we store the information given by Γ as local lists and pass these among levels, the storage required is $\mathcal{O}()$.

Finally, reducing the matrix $M_{1,2}$ is $\mathcal{O}(m^2a_0)$. Note that in the traditional reduction, the number of columns is the number of 2-cells which is nk^2 and the number of rows is the number of 1-cells nk so the time it takes is $\mathcal{O}(n^3k^5)$.

3 Synthetic Data: Generating Functions

Shapes	Function f for signal
Periodic	
cos	f(t) = amp * cos(2*pi/per * t - pshift*(2*pi/per)) + amp
cos 2	per2 = per * 0.3333<br>amp2 = amp * 0.50<br>pshift2 = (pshift + (per2 * 0.25)) % per<br>f(t) = amp * cos(2*pi/per * (t - pshift))<br>+ amp2 * cos(2*pi/per2 * (t - pshift2)) + amp<br>The signal height is then scaled to match the original amplitude.
peak	peak = 20<br>f(t) = amp * (-1 + 2 * fabs(cos(pi/per * t - pshift*(pi/per)))**peak) + amp
peak2	peak1 = 10, peak2 = 40, amp2 = amp * 0.70,<br>pshift2 = (pshift1 + (per* 0.5)) % per<br>f(t) =<br>amp1 * (-1 + 2 * abs(cos(pi/per * (t - pshift1)))**peak1) +<br>amp2 * (-1 + 2 * abs(cos(pi/per * (t - pshift2)))**peak2)<br>The signal is then adjusted to have minimum zero.
trend exp	trende = 0.027<br>f(t) = amp * cos(2*pi/per * (t - pshift)) + amp<br>+ exp(trende * t)
trend linear	trendl = 0.5<br>f(t) = amp * cos(2*pi/per * t - pshift*(2*pi/per))<br>+ (trendl * t) + amp
damp	damp = 0.01<br>f(t) = amp * cos(2*pi/per * t - pshift*(2*pi/per)) * exp(-damp*t) + amp
saw	f(t) = 2 * amp * (((t - pshift) % per) / (per-1))
square	f(t) = 2 * amp * round(((t - pshift) % per) / (per-1))
contract	f(t) = amp * cos(2*pi/per * (t ** 2 / per - pshift)) + amp
Non-Periodic	
flat	height = [0, 2 * amp]<br>f(t) = height
linear	m = [-0.5, 0.5]<br>f(t) = (m * t)<br>The signal is then adjusted to have minimum zero.
exp decay	k = [0.023, 0.092], so y < 1 between t = 50 and t = 200<br>f(t) = 2 * amp * exp(-k * t)
sigmoid	growth = -0.1, pshift = [50, 150]<br>U = 0, L = amp * 2; U and L swapped with 0.5 probability<br>f(t) = L + ((U-L) / (1 + exp(growth * (t - pshift))))

Table S1: Functions of time (t) used to generate profiles. The value for amplitude (amp) is 100. The period (per) is 100. Phase shift (pshift) is selected from a uniform distribution within 0 to the period length.

**Noise Model**	**Function**	**Noise Levels**
Gaussian Additive	normal(0, sd) + x	sd = {0, 12, 25, 37, 50}
Laplacian Additive	laplace(0, b) + x	b = {0, 8.49, 17.68, 26.16, 35.36}
Gaussian Multiplicative	normal(1, sd) * x	sd = {0, 0.12, 0.25, 0.37, 0.5}
Laplacian Multiplicative	laplace(1, b) * x	b = {0, 0.08, 0.18, 0.26, 0.35}

Table S2: Noise models applied to the signals.

4 Synthetic Data: ROC Plots

ROC plots of Performance on identifying periodic signals for different signal shapes and noise levels. These plots show the degradation in performance for classifying periodic from non-periodic signals. Different shapes of periodic signals included were cosine, two cosine signals with different amplitudes, cosine peaked, two peaked signals with different amplitudes, cosine with a linear trend, cosine with an exponential trend, cosine damped, sawtooth, square waves, and a contracting cosine signal. Non-periodic shapes included were linear, exponential, flat and sigmoidal. Four noise models were applied to the set of signals, each at five different levels: Gaussian Additive with standard deviation SD equal to $0, 12, 25, 37$ and 50, Laplacian Additive with spread b at 0, 8.49, 17.68, 26.16, and 35.36, Gaussian Multiplicative with SD equal to 0, 0.12, 0.25, 0.37 and 0.5, and Laplacian Multiplicative with $b = \{0, 0.08, 0.18, 0.26, 0.35\}$. The standard deviation SD for additive (resp. multiplicative) Gaussian noise and the spread b for additive (resp. multiplicative) Laplacian noise were matched $(SD = \sqrt{2}b)$ so the distributions would have the same variance. The additive and multiplicative variances, however, were not matched to each other.

Shown for all algorithms: SW1perS (SW) (Perea and Harer, 2014), JTK_CYCLE (JTK) (Hughes *et al.*, 2010), Lomb-Scargle (LS) (Glynn *et al.*, 2006), de Lichtenberg (DL) (de Lichtenberg *et al.*, 2005), Persistent Homology (PH) (Cohen-Steiner *et al.*, 2010).

The R package ROCR was used to compute ROC and AUC (Sing *et al.*, 2005). The results from synthetic data were plotted in R using the ggplot2 package (Wickham, 2009).

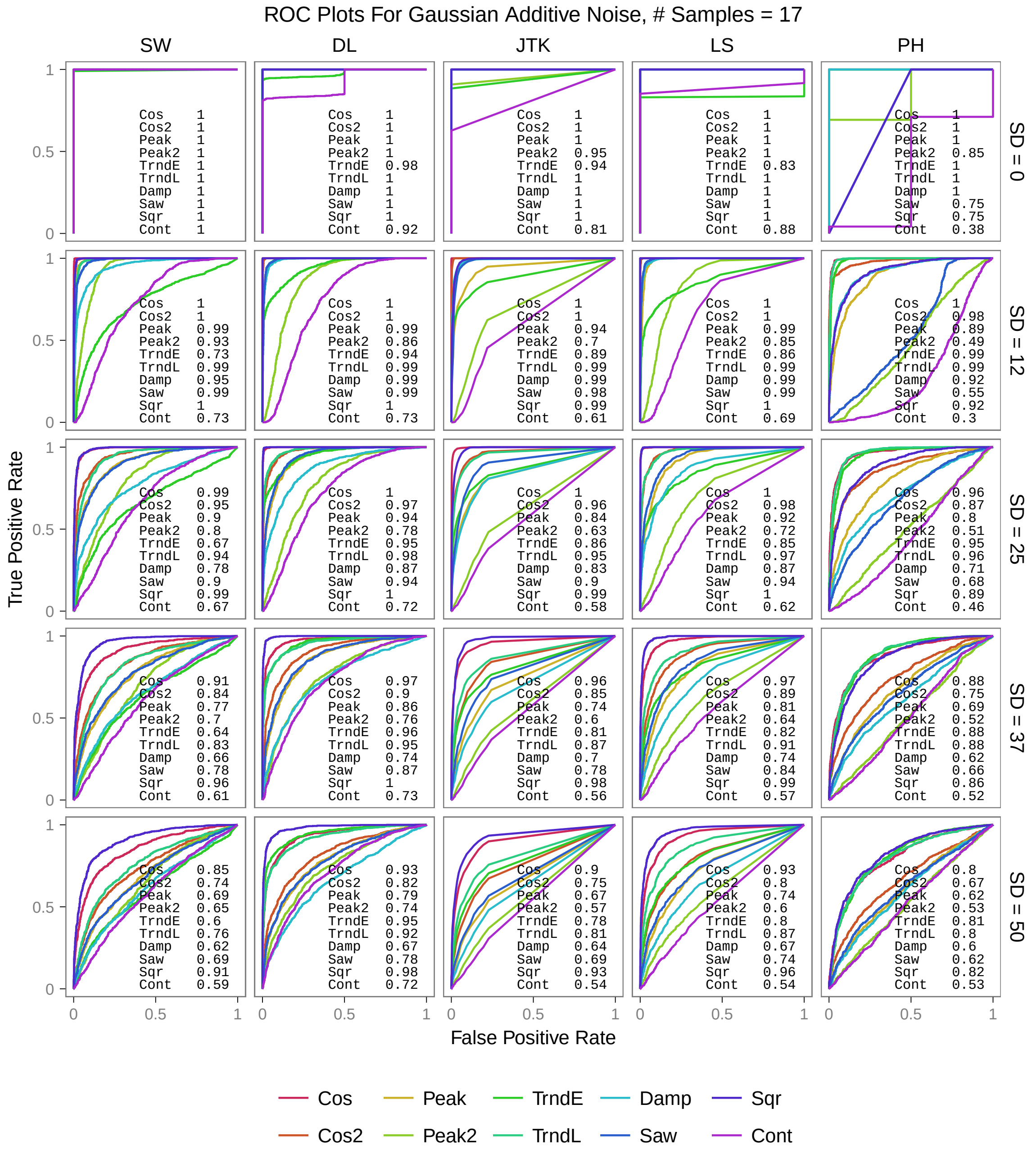

Figure S3: ROC plots of Performance on identifying periodic signals for different signal shapes and additive Gaussian noise levels on 17 samples.

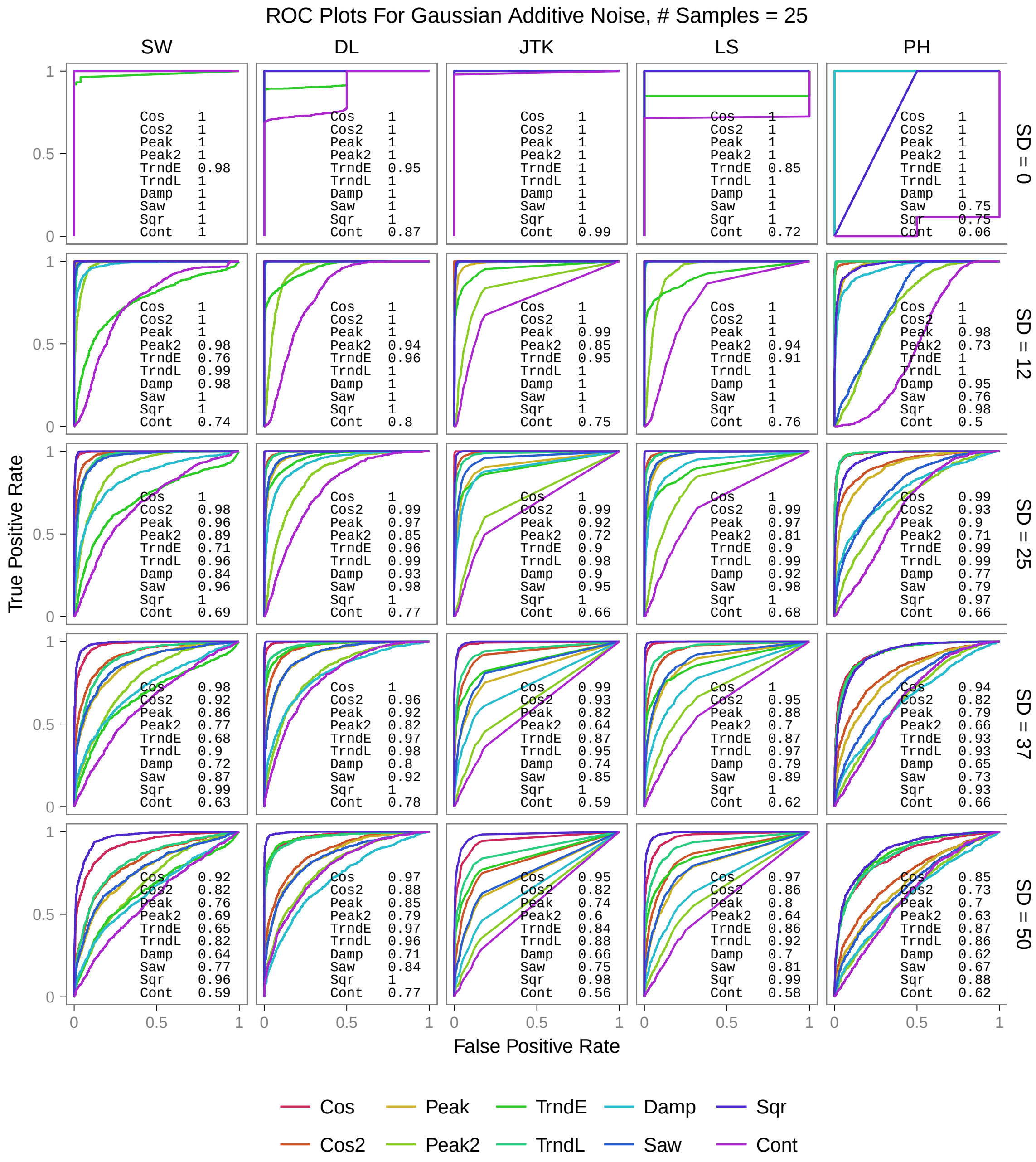

Figure S4: ROC plots of Performance on identifying periodic signals for different signal shapes and additive Gaussian noise levels on 25 samples.

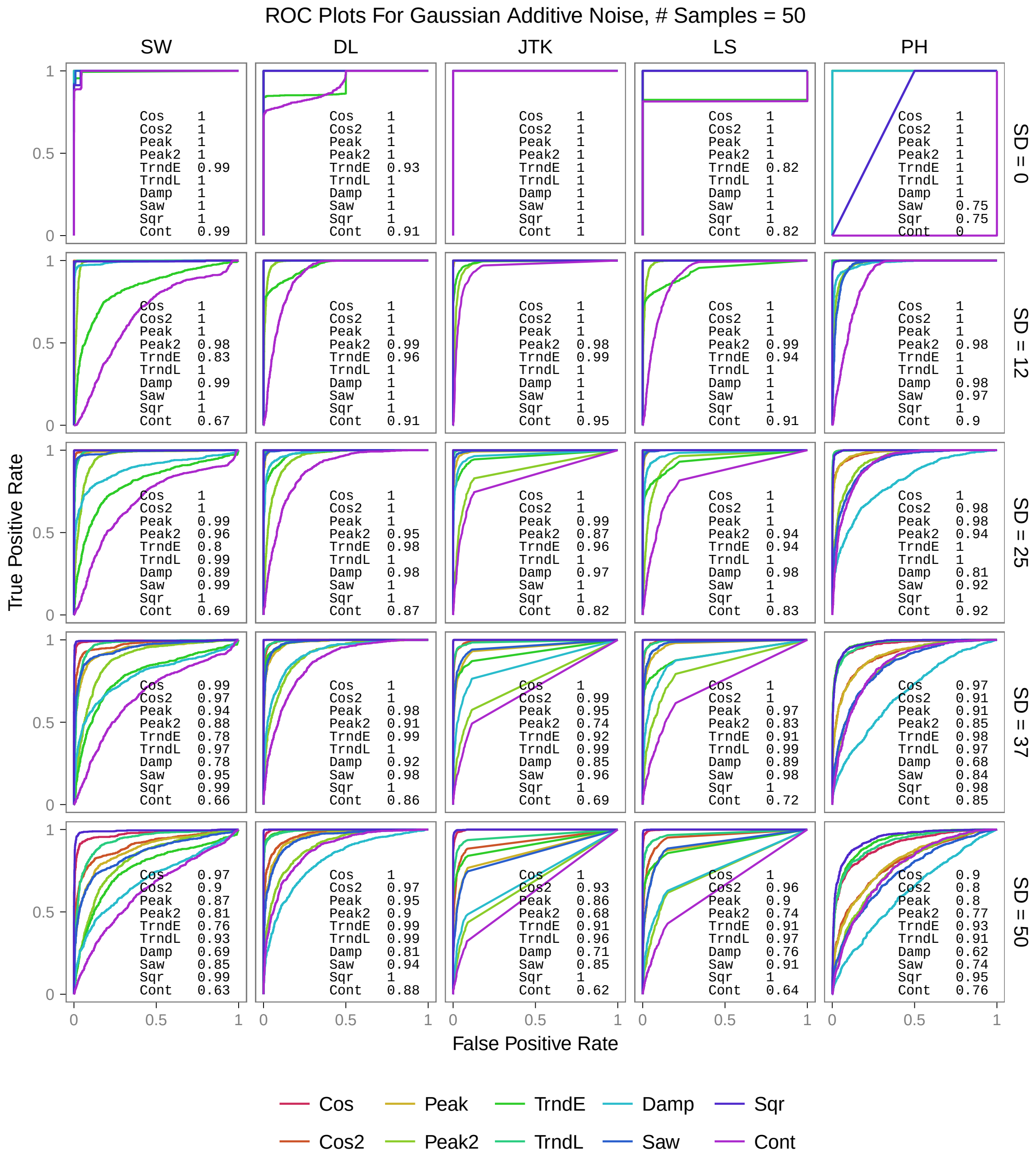

Figure S5: ROC plots of Performance on identifying periodic signals for different signal shapes and additive Gaussian noise levels on 50 samples.

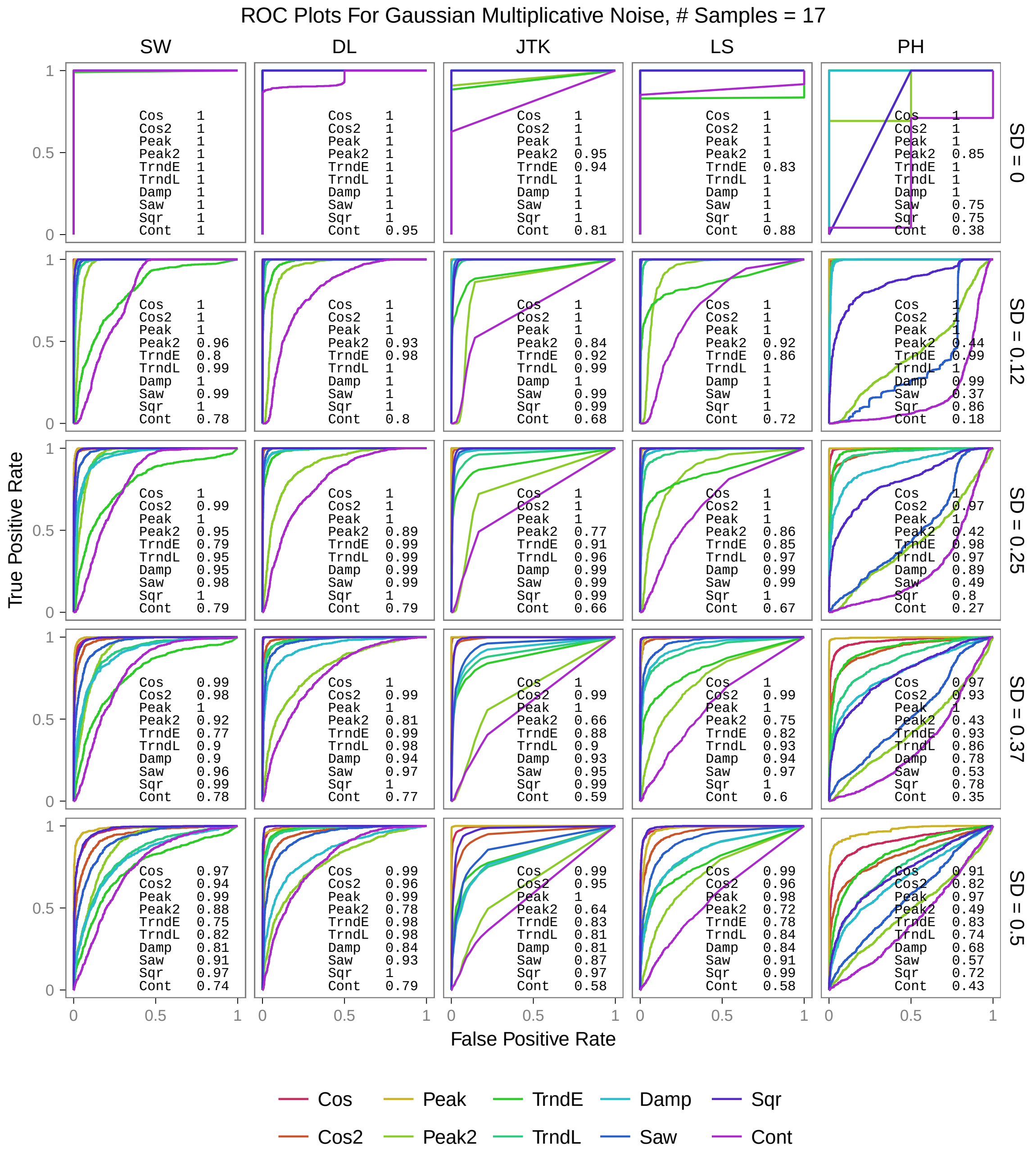

Figure S6: ROC plots of Performance on identifying periodic signals for different signal shapes and multiplicative Gaussian noise levels on 17 samples.

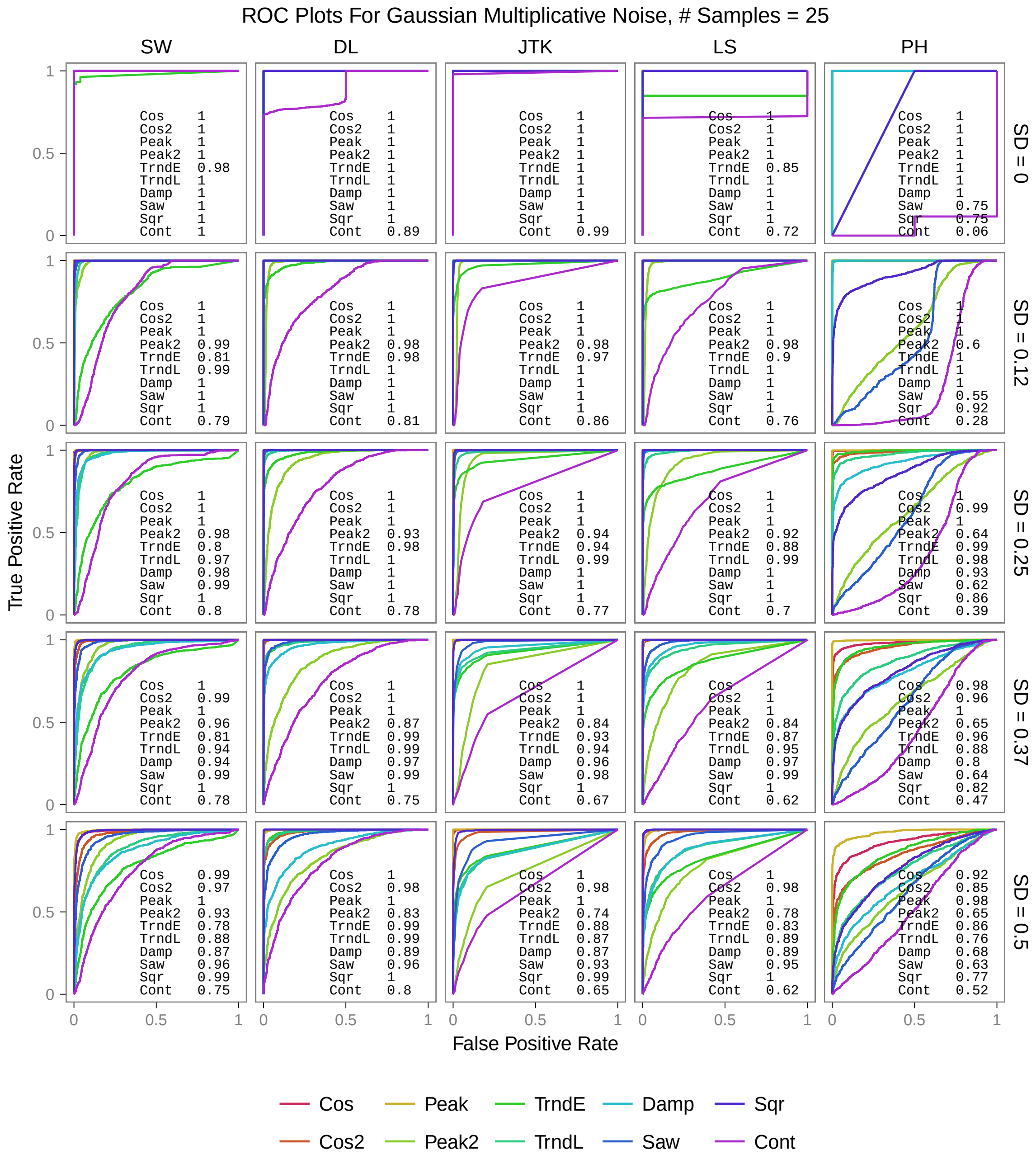

Figure S7: ROC plots of Performance on identifying periodic signals for different signal shapes and multiplicative Gaussian noise levels on 25 samples.

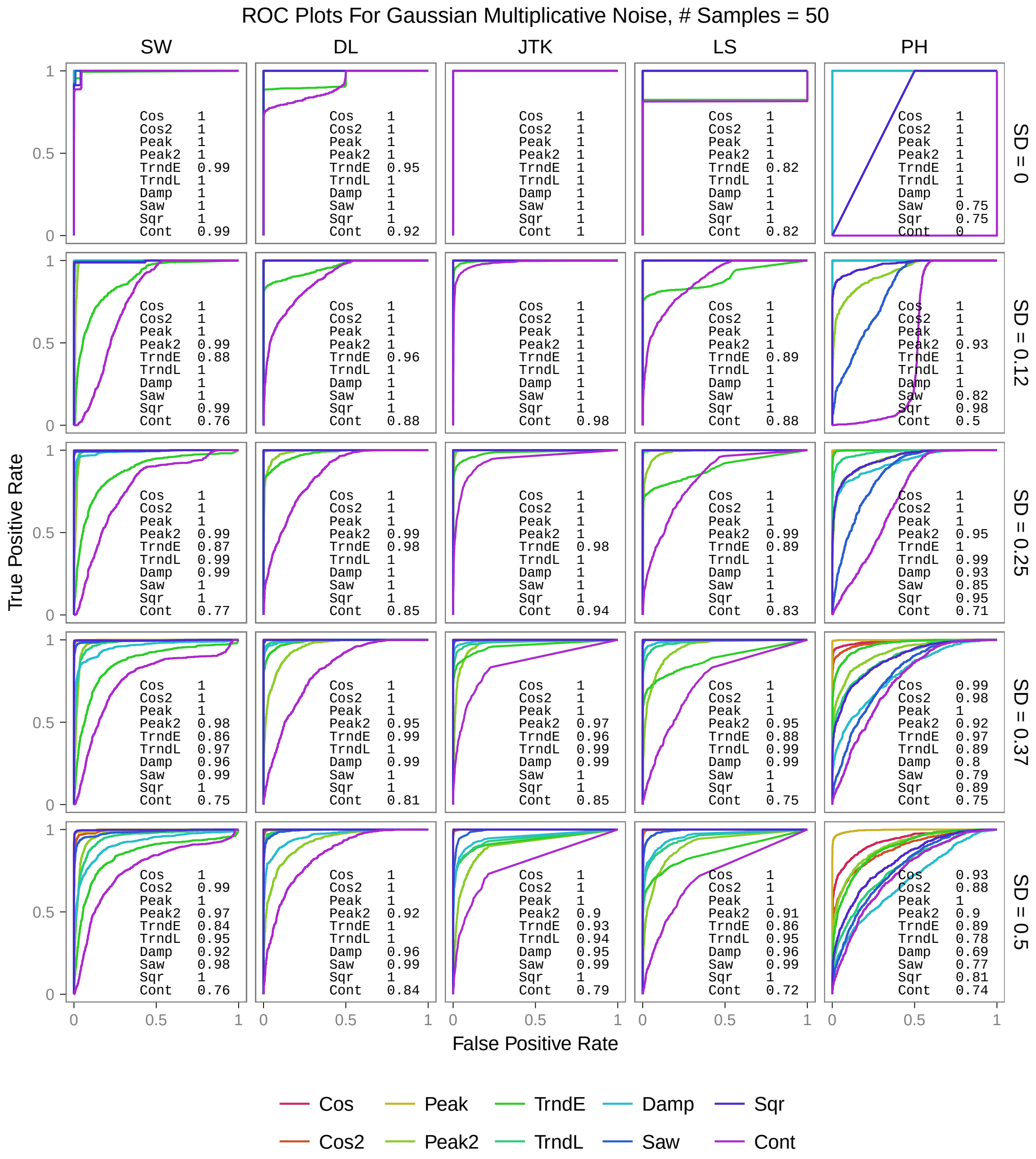

Figure S8: ROC plots of Performance on identifying periodic signals for different signal shapes and multiplicative Gaussian noise levels on 50 samples.

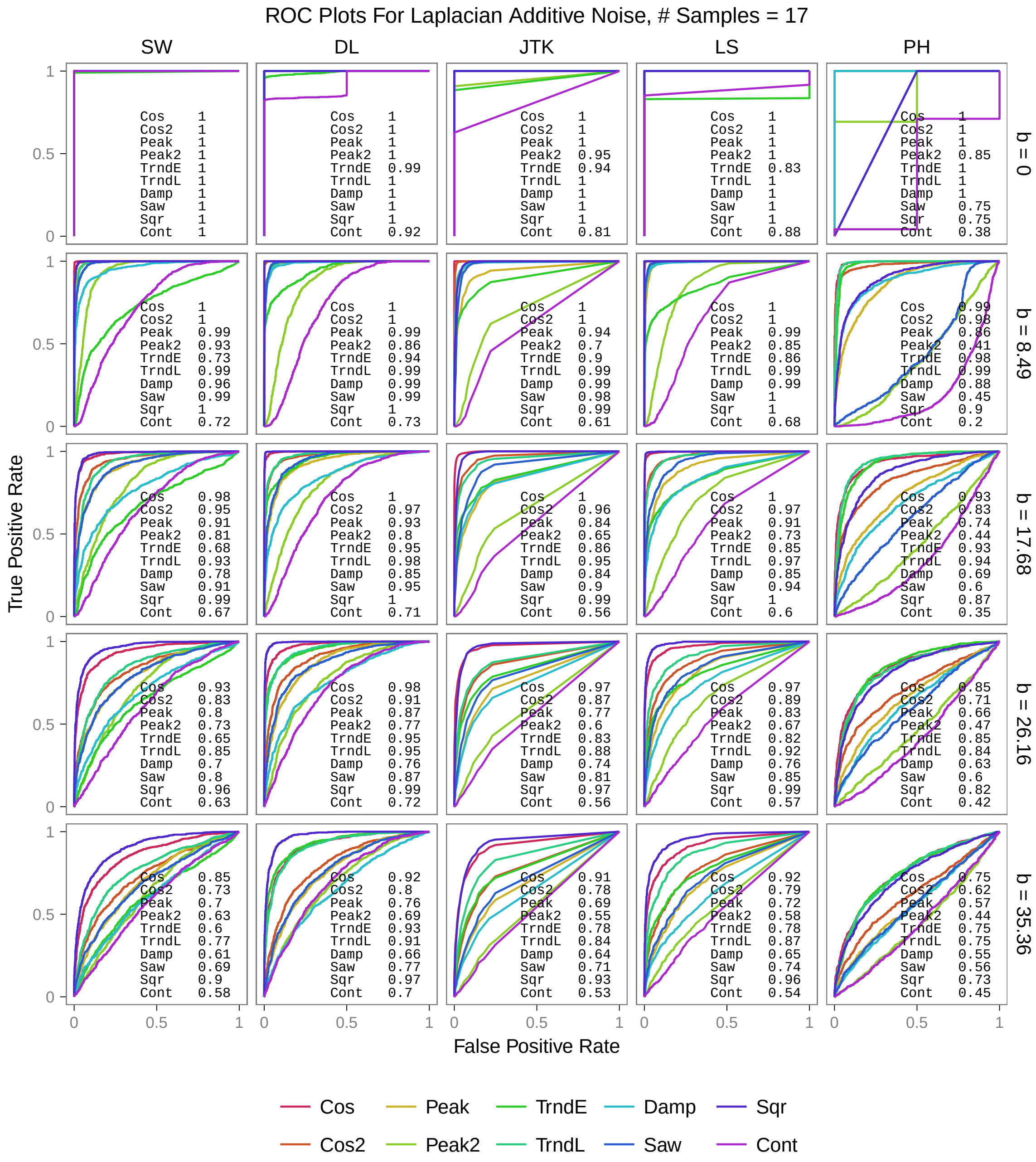

Figure S9: ROC plots of Performance on identifying periodic signals for different signal shapes and additive Laplacian noise levels on 17 samples.

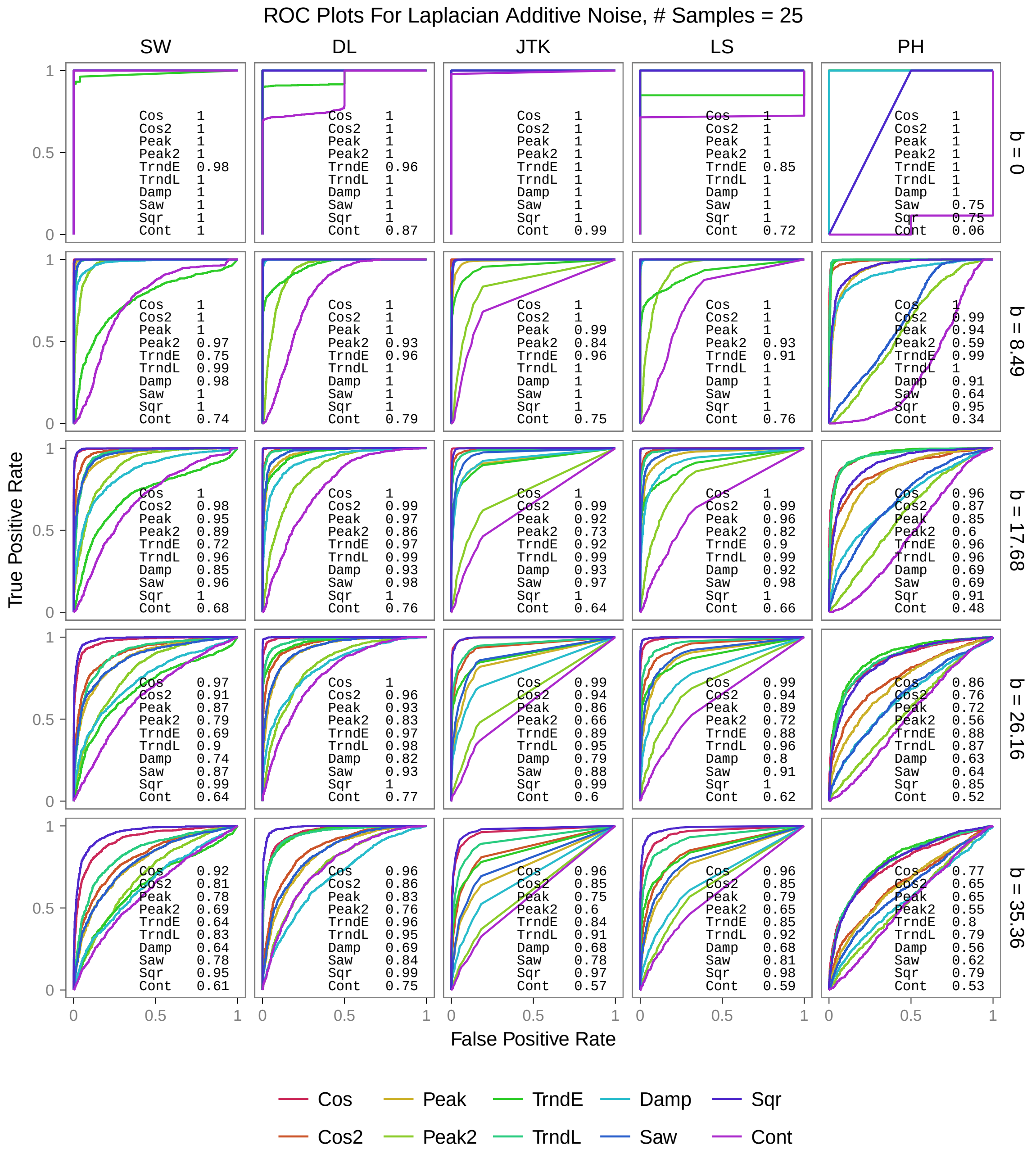

Figure S10: ROC plots of Performance on identifying periodic signals for different signal shapes and additive Laplacian noise levels on 25 samples.

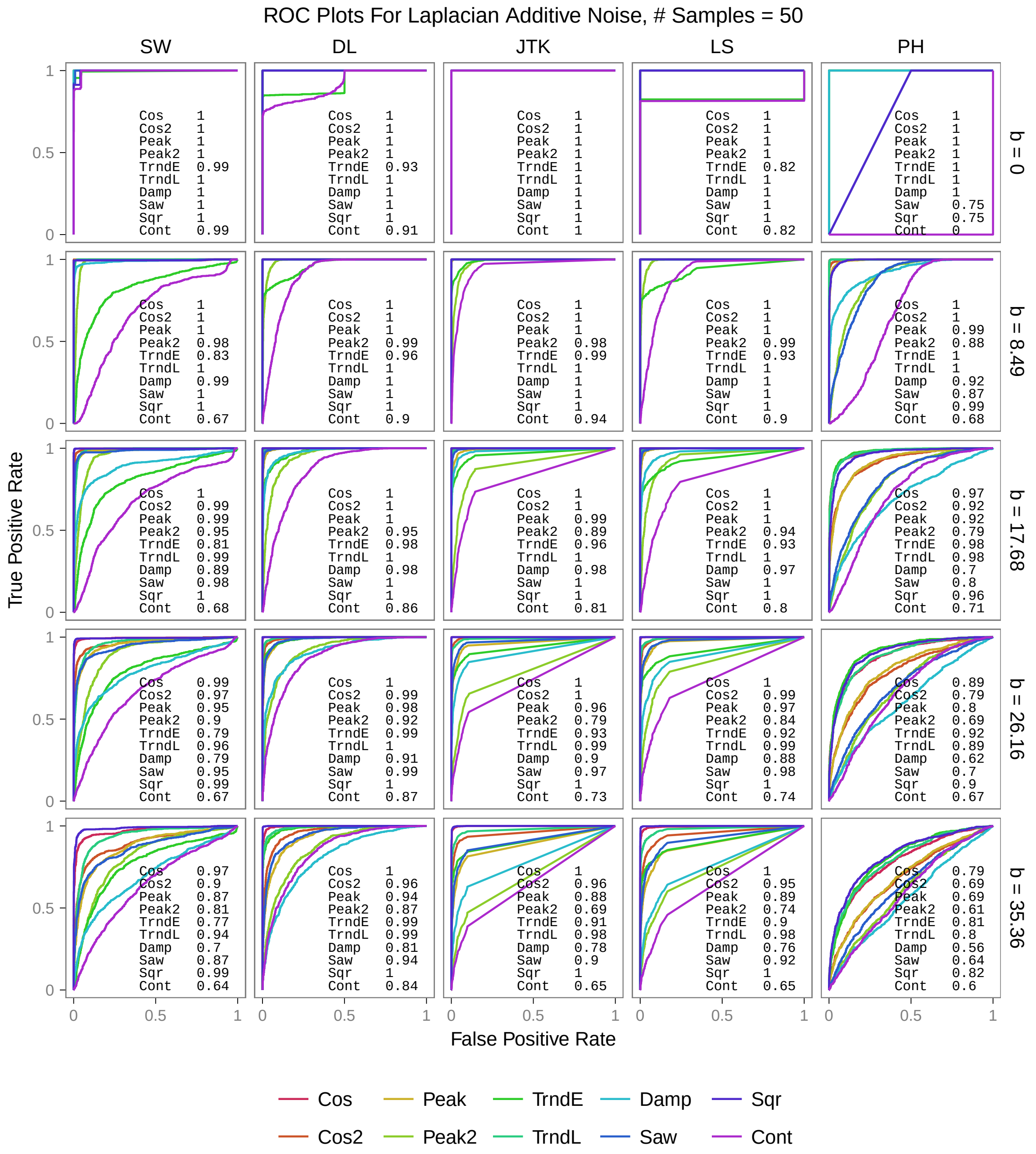

Figure S11: ROC plots of Performance on identifying periodic signals for different signal shapes and additive Laplacian noise levels on 50 samples.

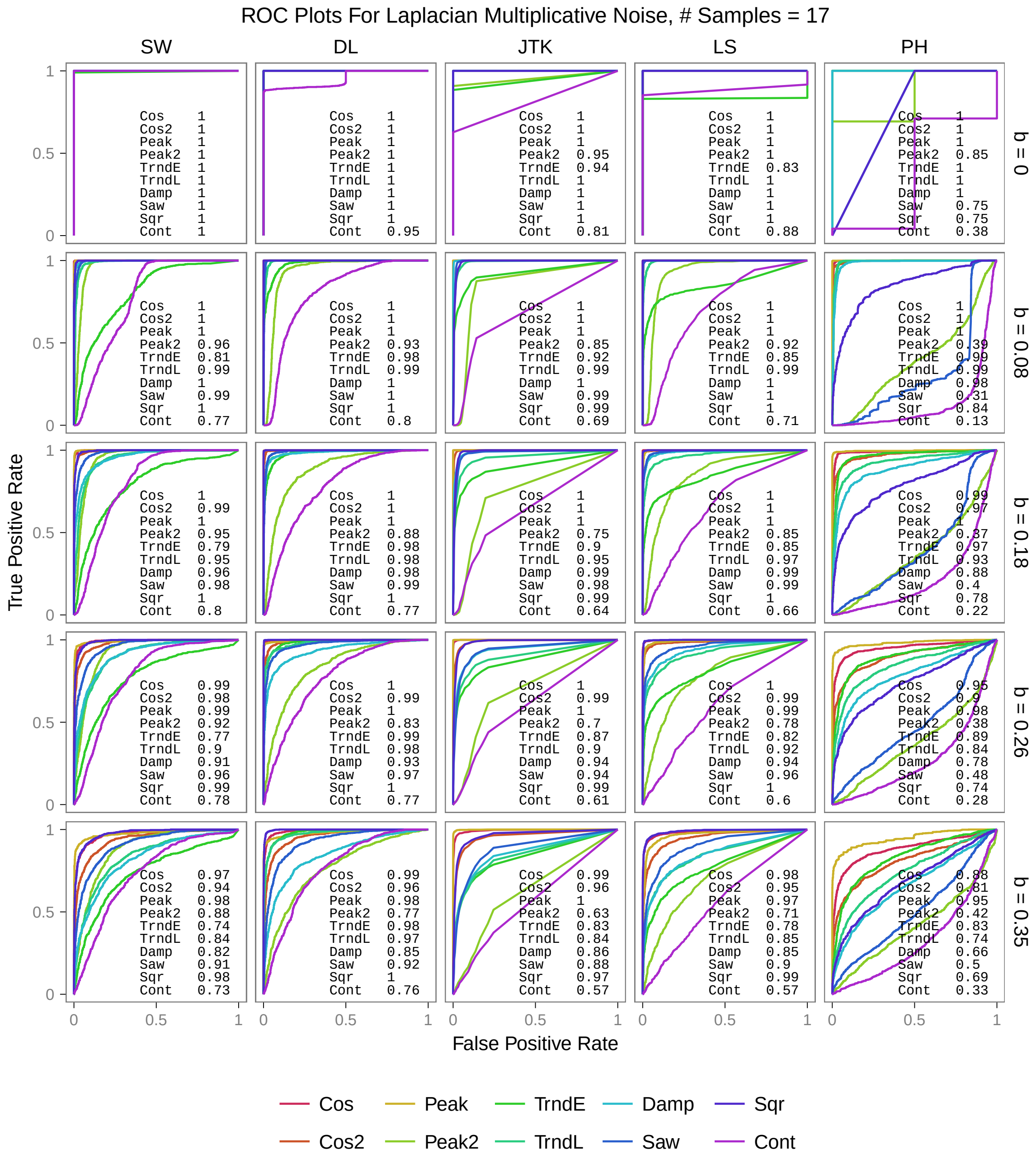

Figure S12: ROC plots of Performance on identifying periodic signals for different signal shapes and multiplicative Laplacian noise levels on 17 samples.

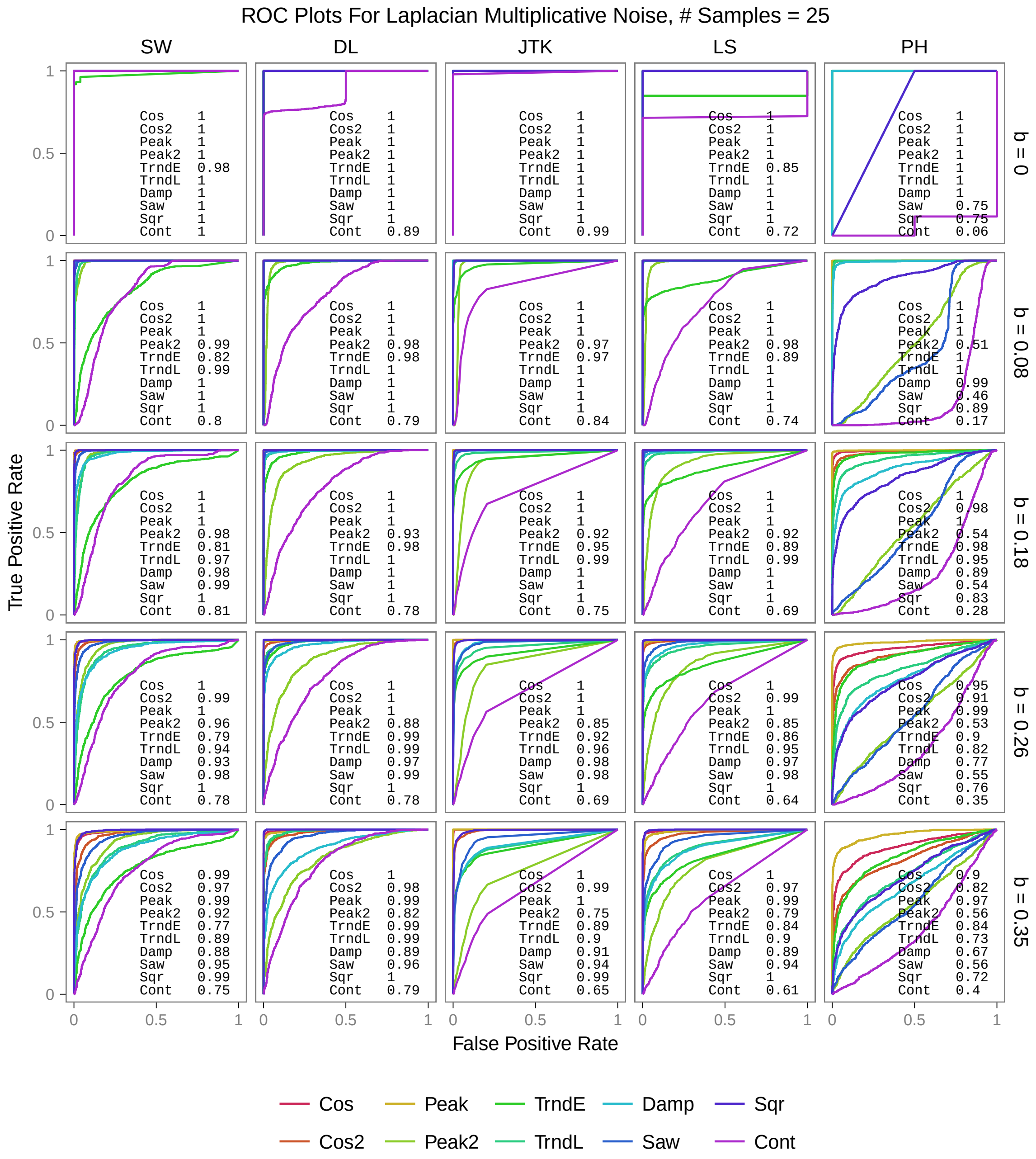

Figure S13: ROC plots of Performance on identifying periodic signals for different signal shapes and multiplicative Laplacian noise levels on 25 samples.

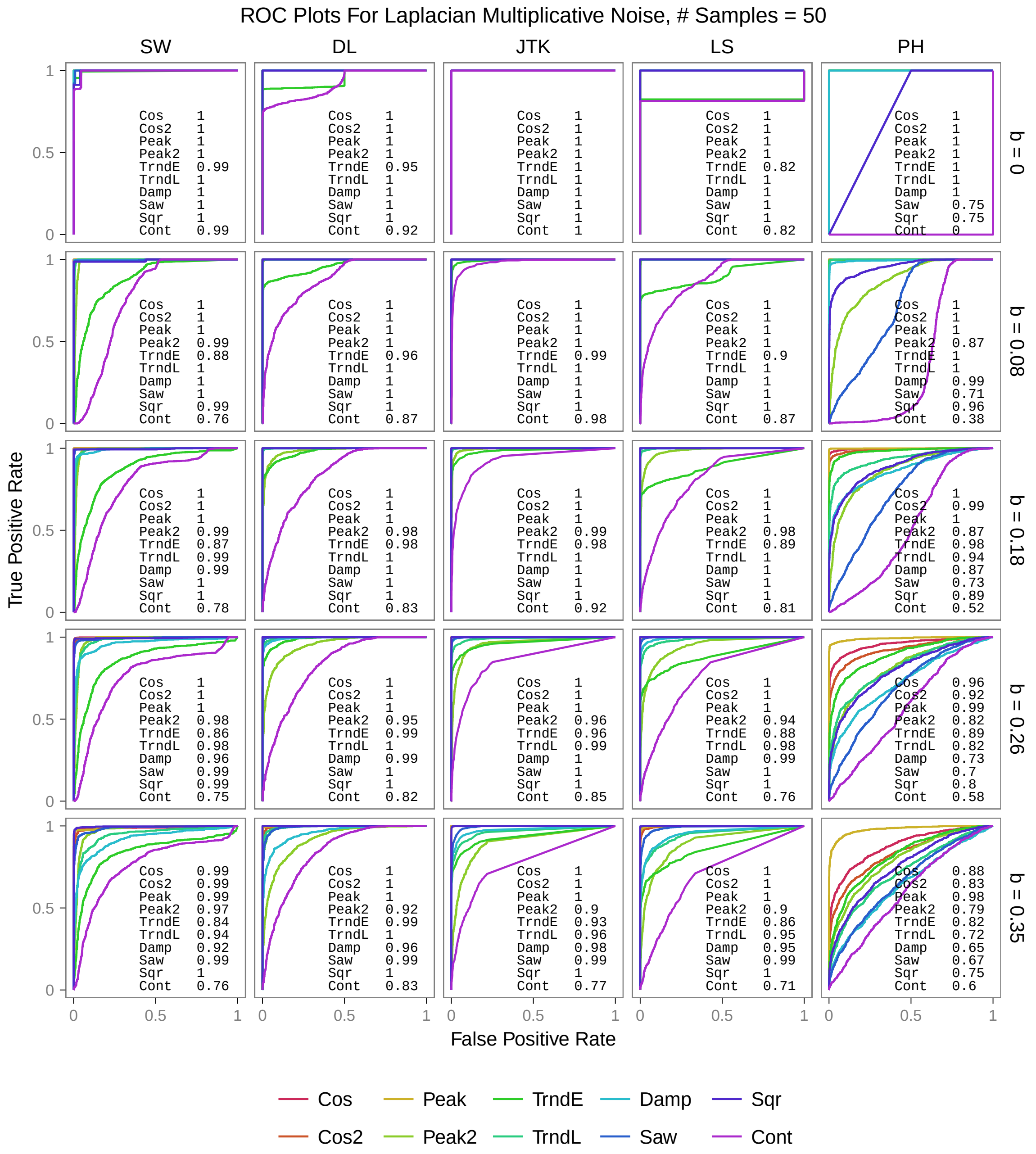

Figure S14: ROC plots of Performance on identifying periodic signals for different signal shapes and multiplicative Laplacian noise levels on 50 samples.

5 Synthetic Data: Score Distributions

Score distributions for each algorithm on synthetic data with different noise levels and sampling densities. These show algorithm biases for signal shapes. Number of samples = 50, 25, 17 and noise levels (Gaussian Noise SD = 0, 25, 50. The same data set used in the ROC analysis was used. The x-axis shows the scores, log transformed, ranging from the lowest (best score) to the highest (worst score) returned by the algorithm. The y-axis shows the number of profiles receiving the score. Shown for all algorithms: SW1perS (SW), JTK_CYCLE (JTK), Lomb-Scargle (LS), de Lichtenberg (DL), Persistent Homology (PH).

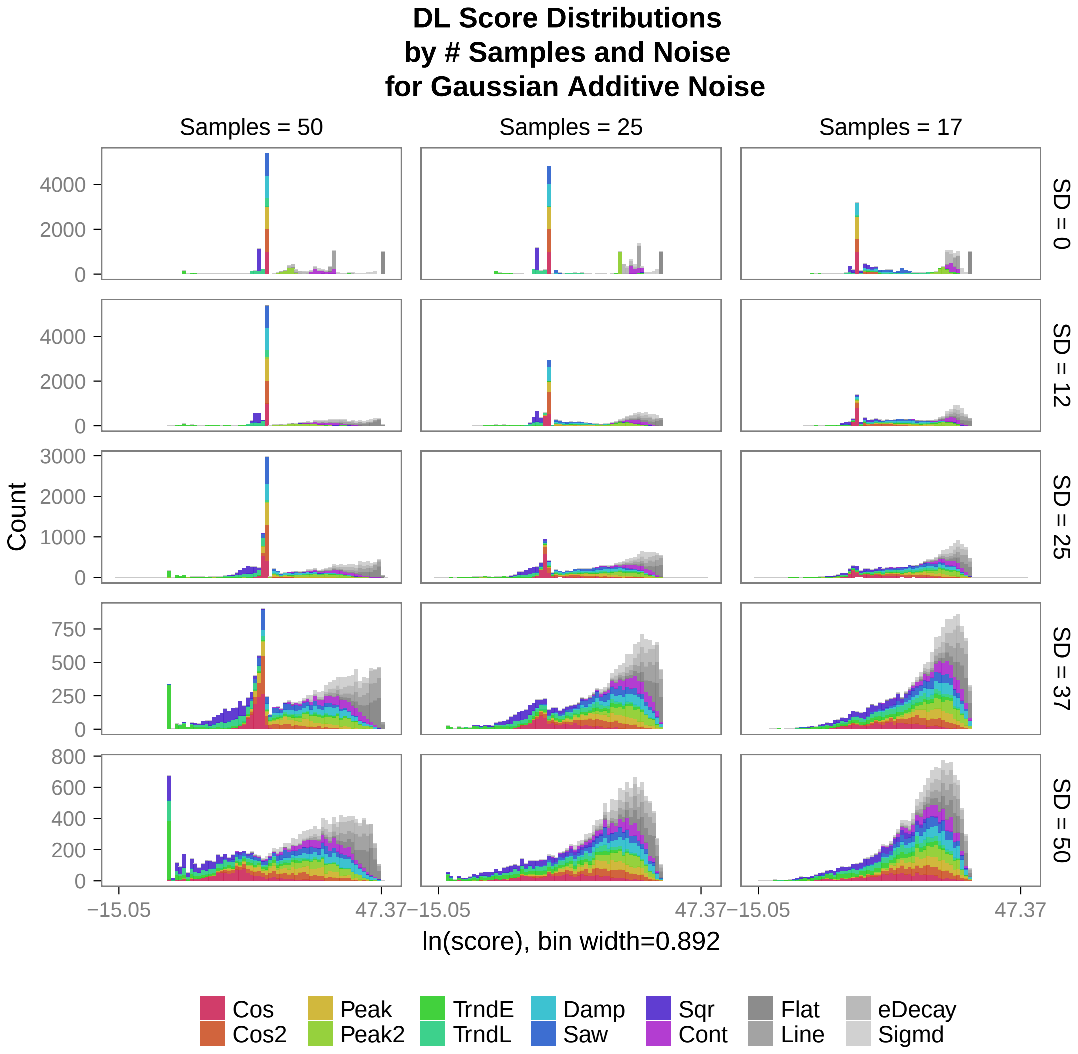

Figure S15: Scores distributions on synthetic data with additive Gaussian noise for de Lichtenberg.

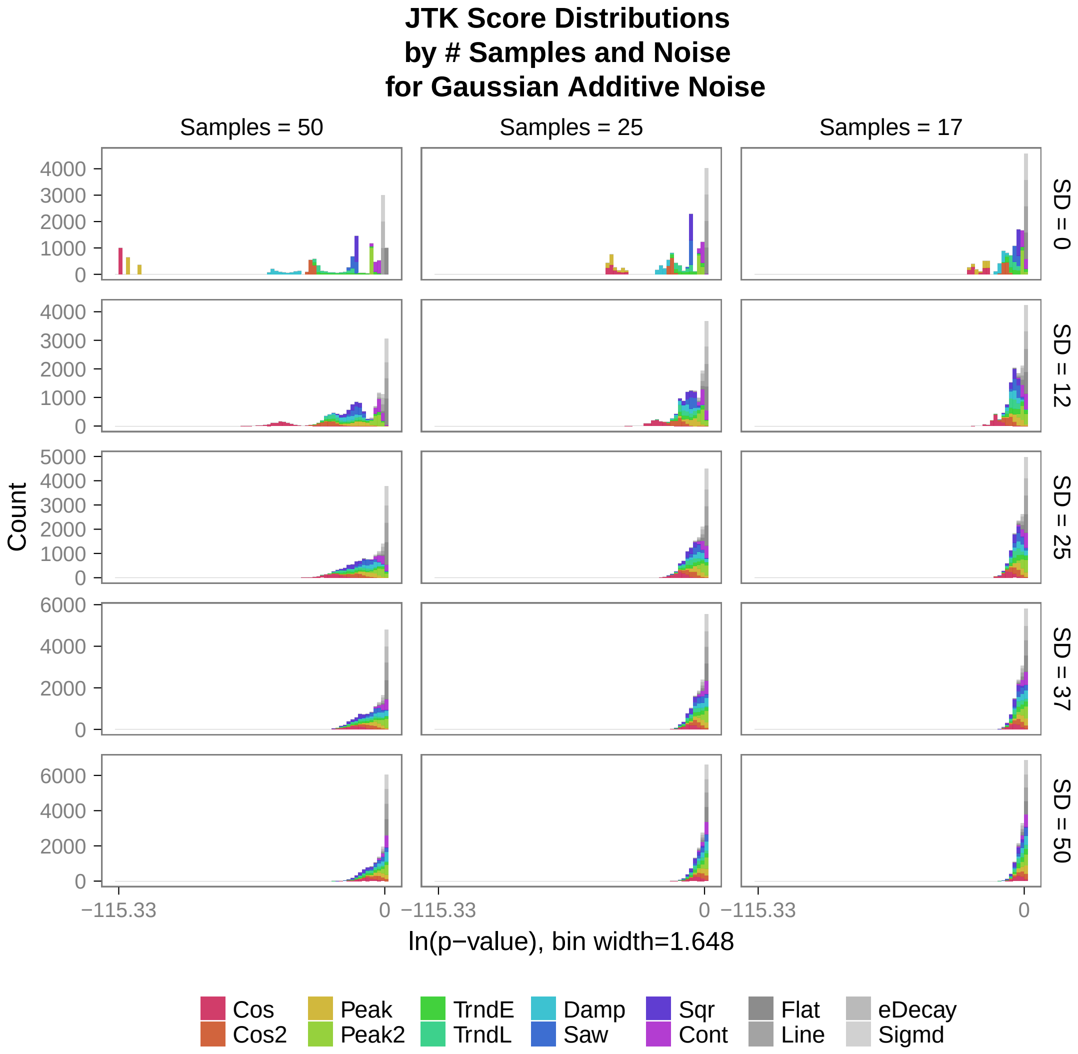

Figure S16: Scores distributions on synthetic data with additive Gaussian noise for JTK.

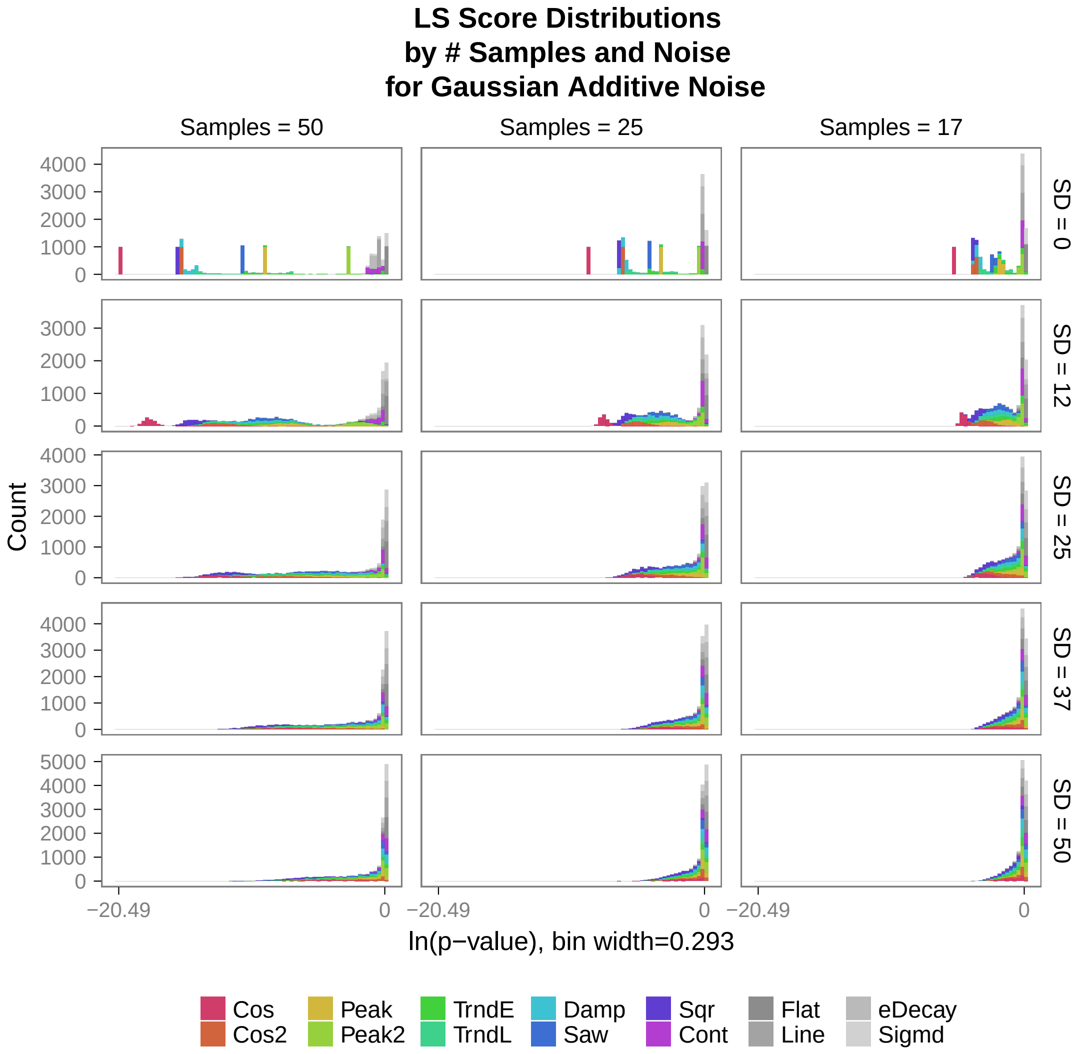

Figure S17: Scores distributions on synthetic data with additive Gaussian noise for Lomb-Scargle.

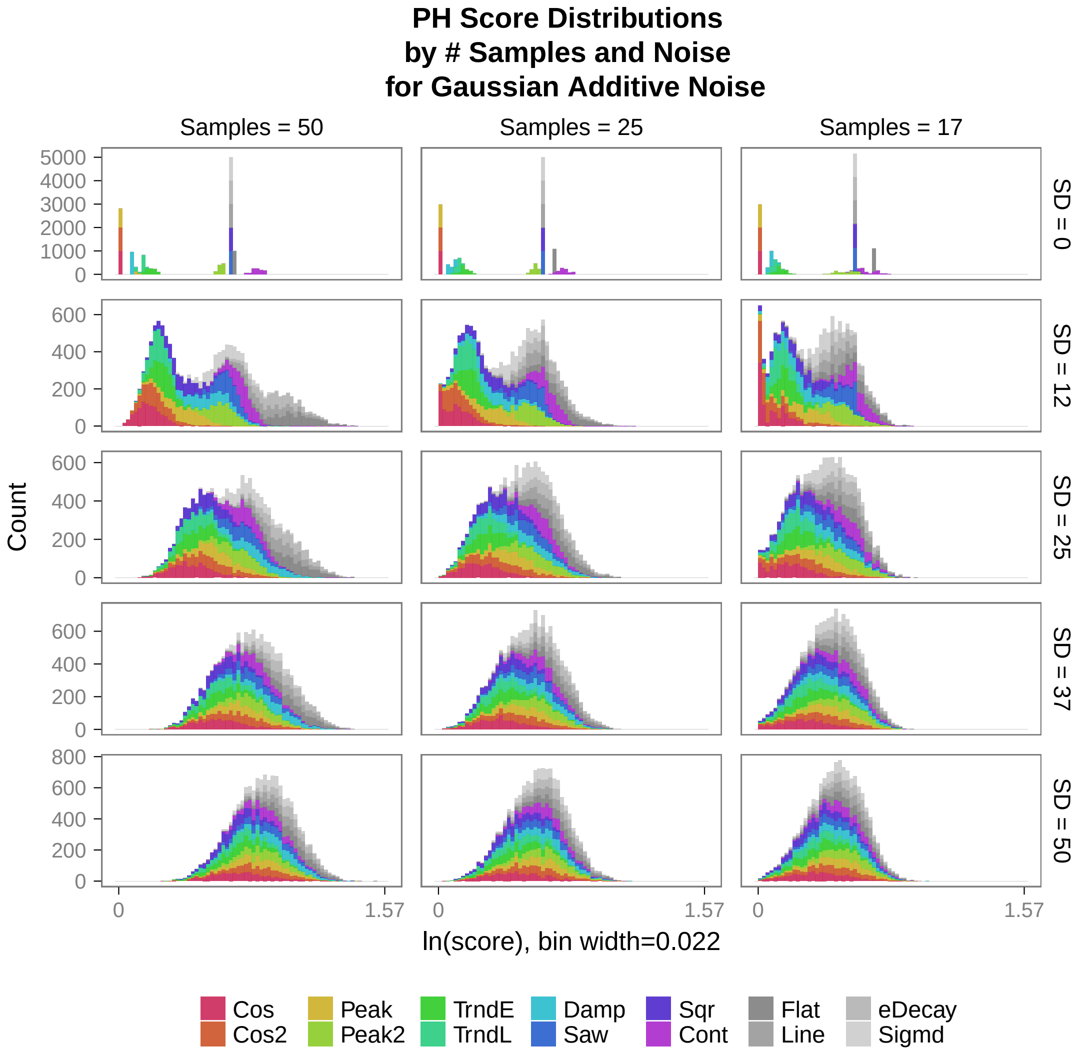

Figure S18: Scores distributions on synthetic data with additive Gaussian noise for PH.

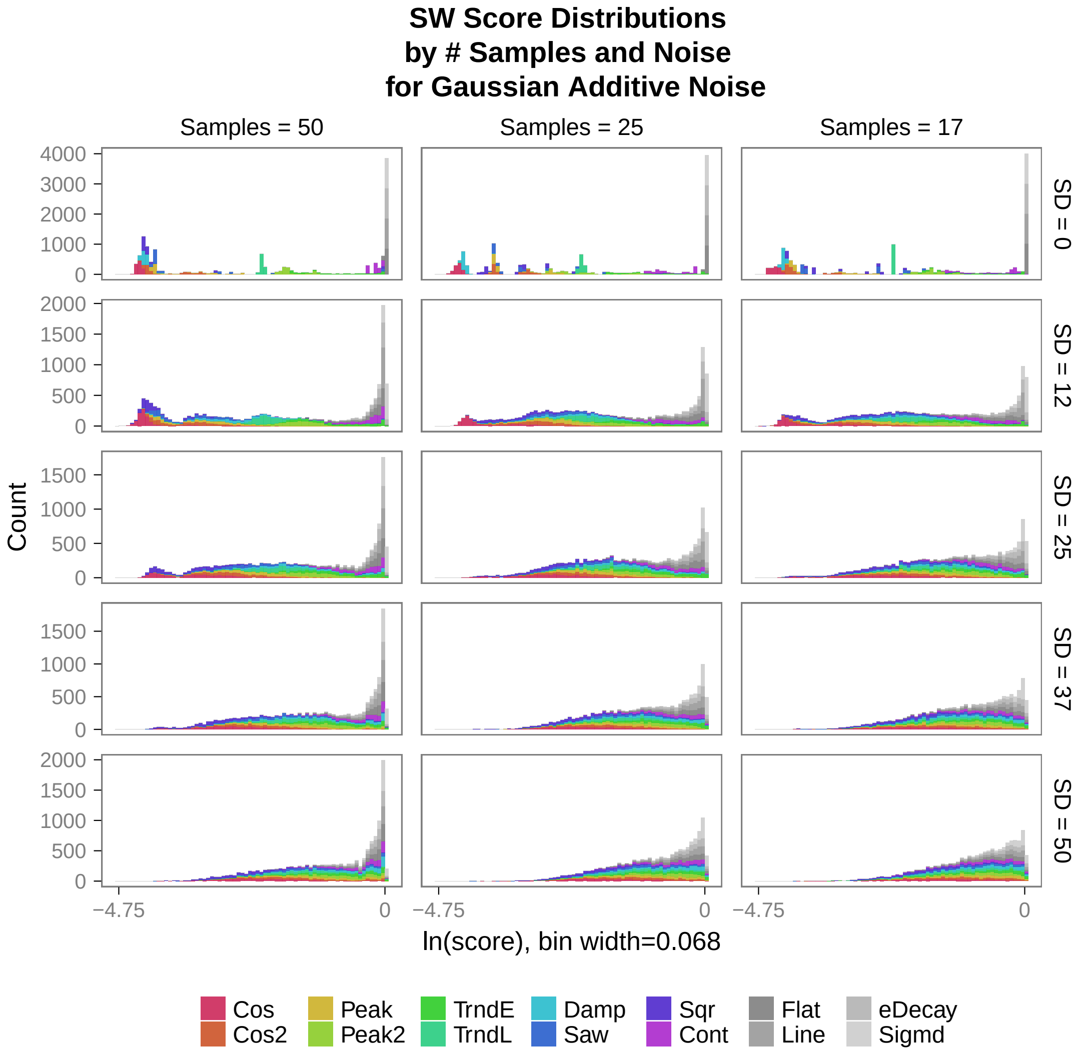

Figure S19: Scores distributions on synthetic data with additive Gaussian noise for SW1perS.

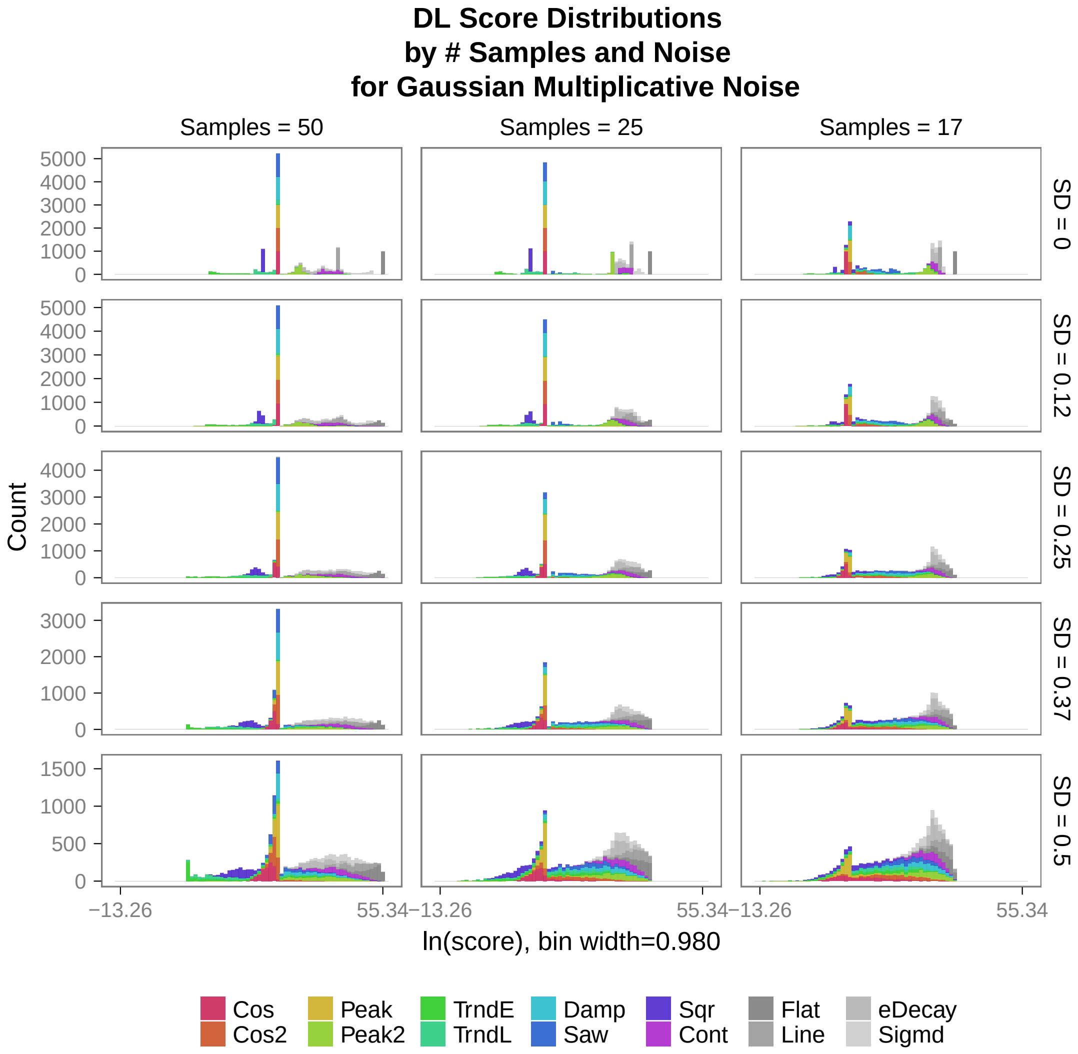

Figure S20: Scores distributions on synthetic data with multiplicative Gaussian noise for de Lichtenberg.

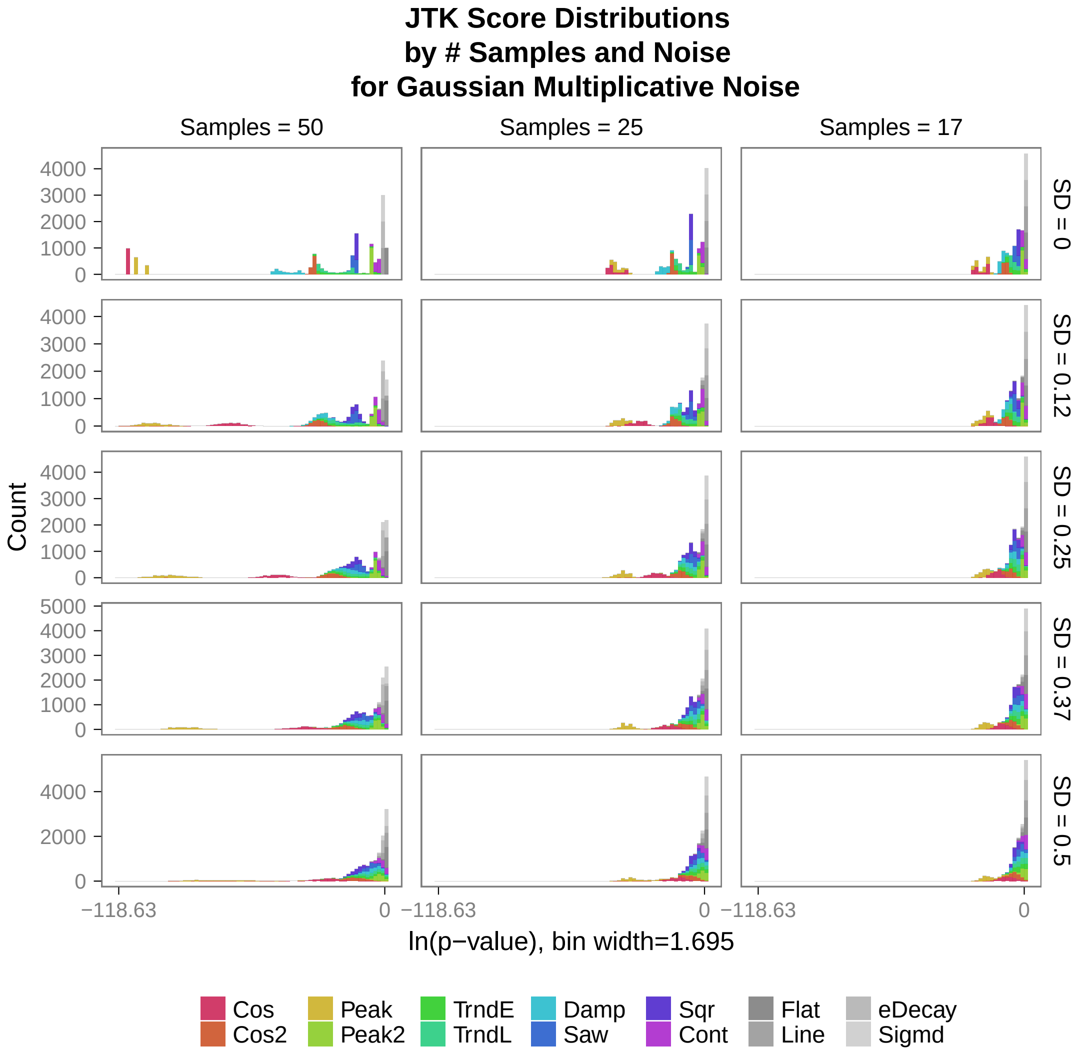

Figure S21: Scores distributions on synthetic data with multiplicative Gaussian noise for JTK.

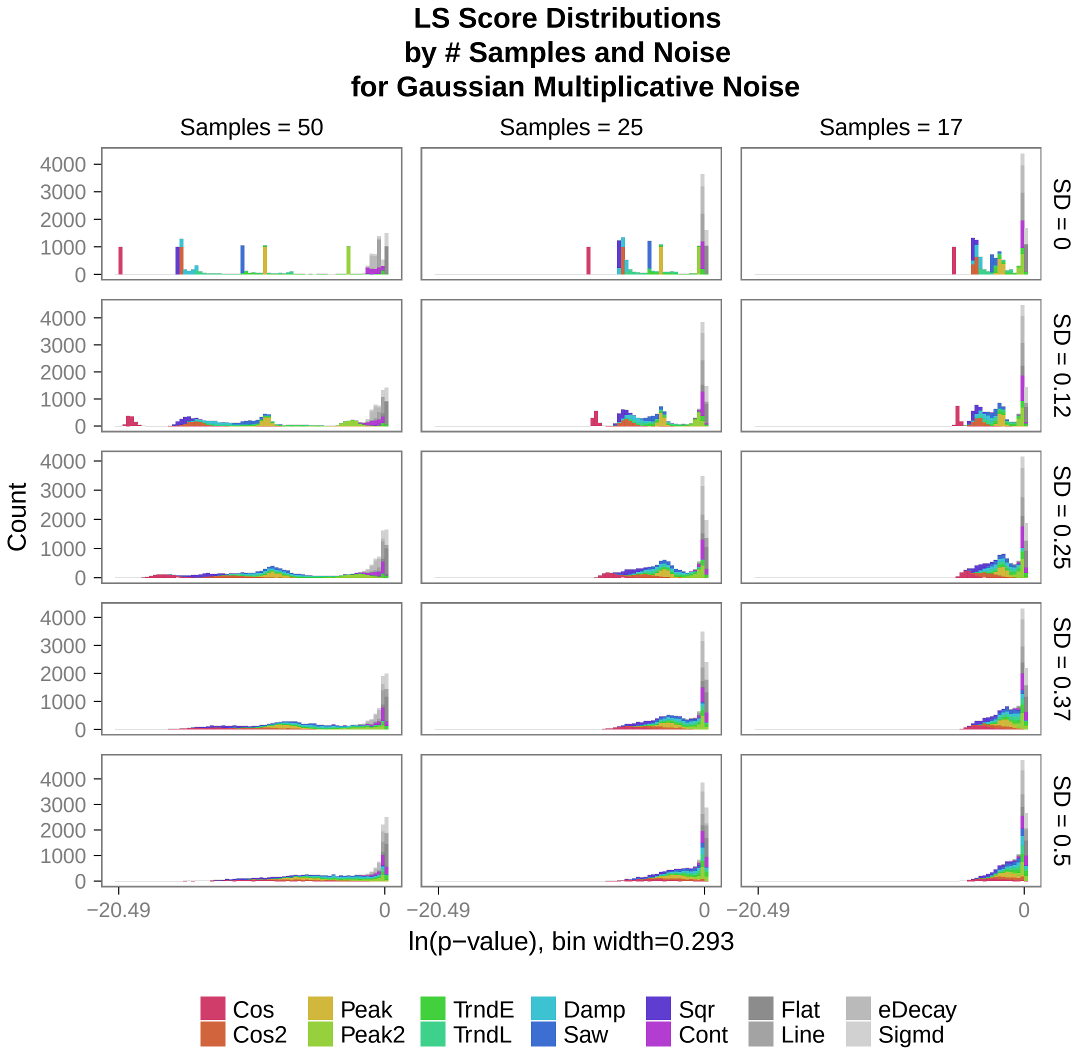

Figure S22: Scores distributions on synthetic data with multiplicative Gaussian noise for Lomb-Scargle.

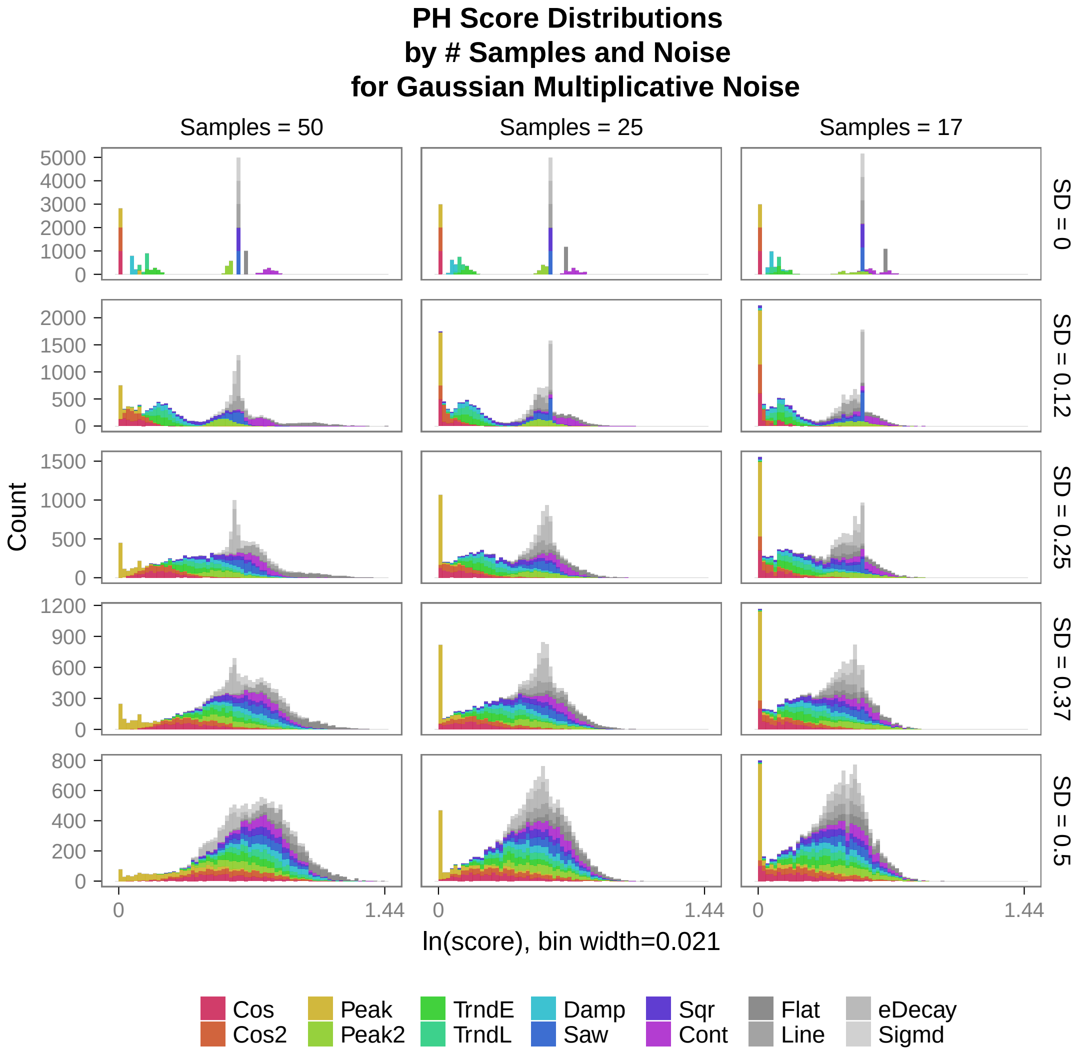

Figure S23: Scores distributions on synthetic data with multiplicative Gaussian noise for PH.

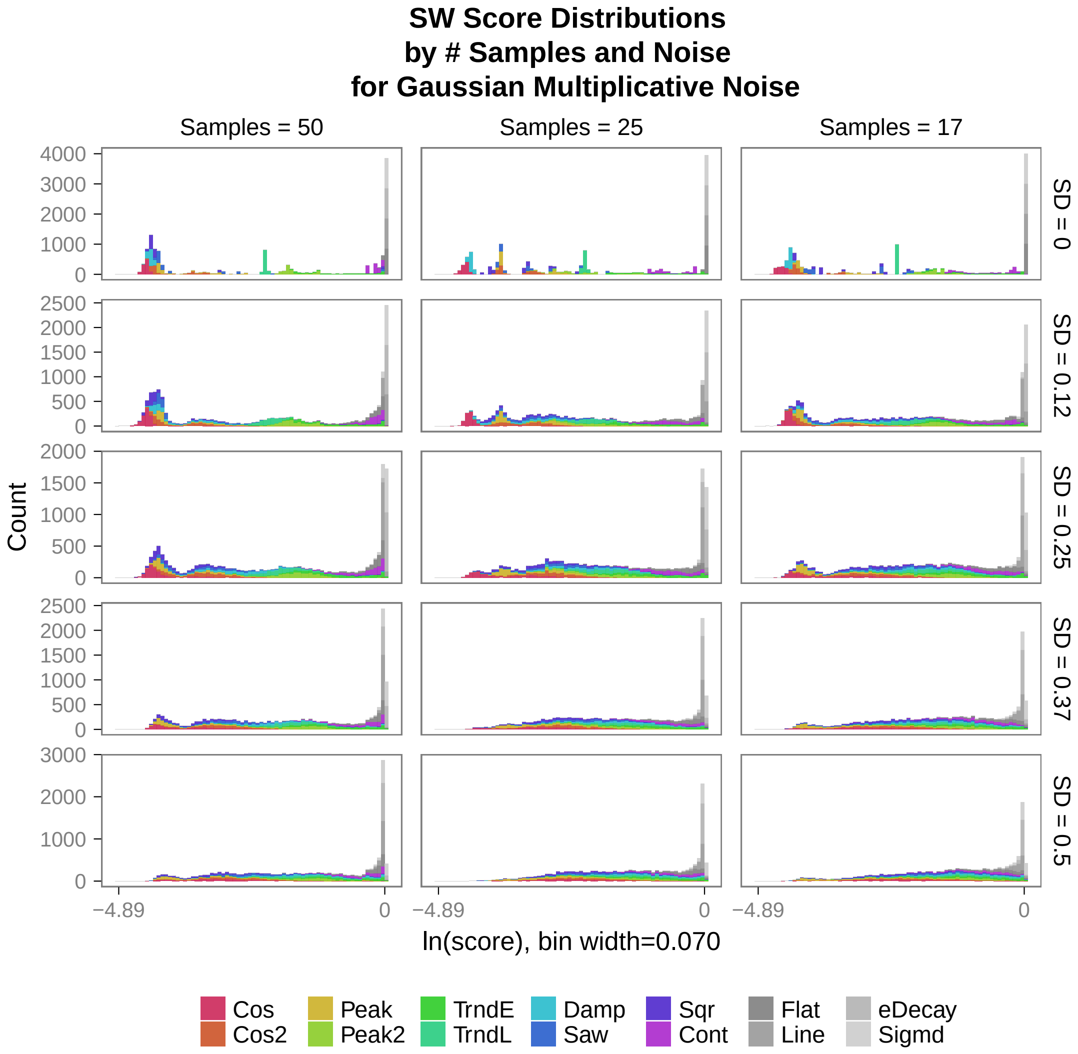

Figure S24: Scores distributions on synthetic data with multiplicative Gaussian noise for SW1perS.

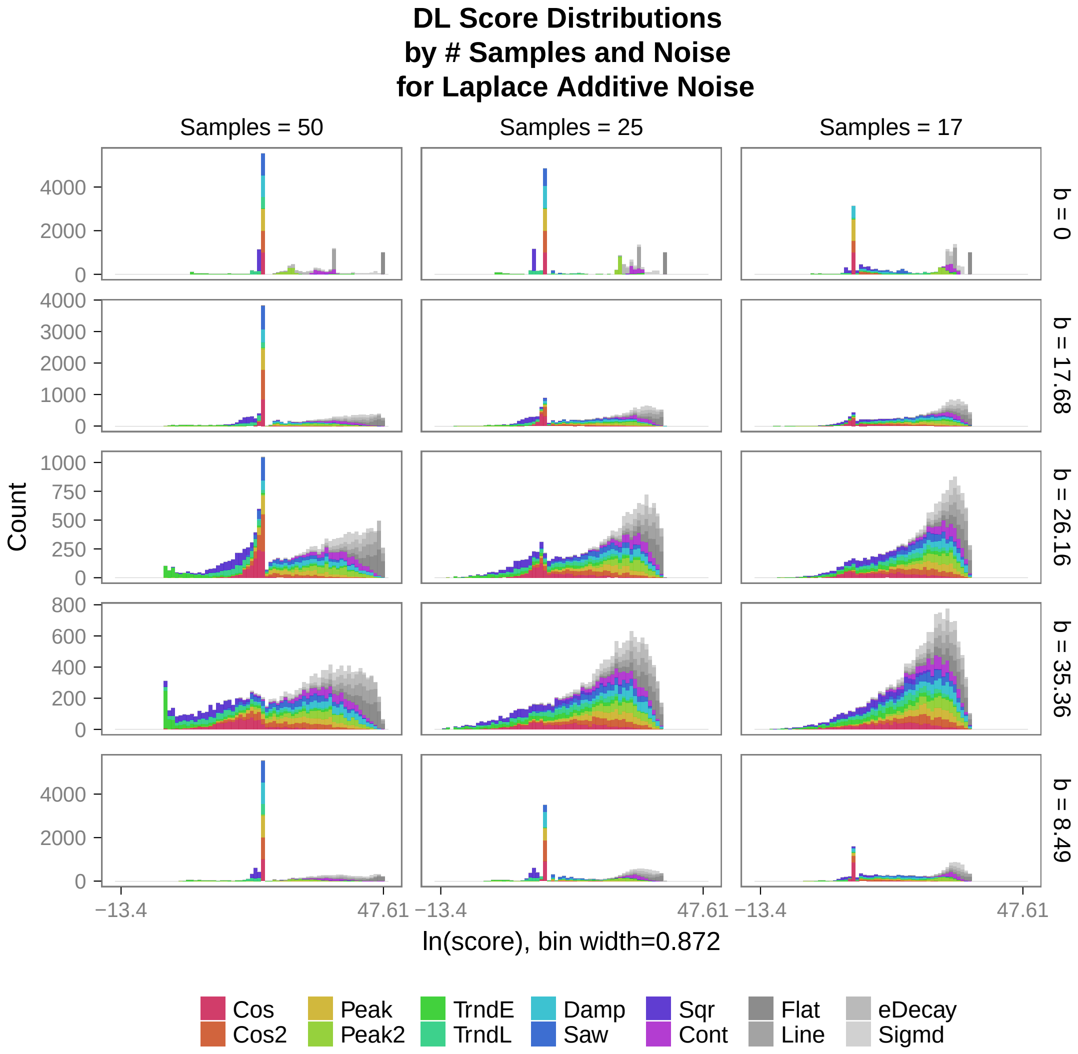

Figure S25: Scores distributions on synthetic data with additive Laplacian noise for de Lichtenberg.

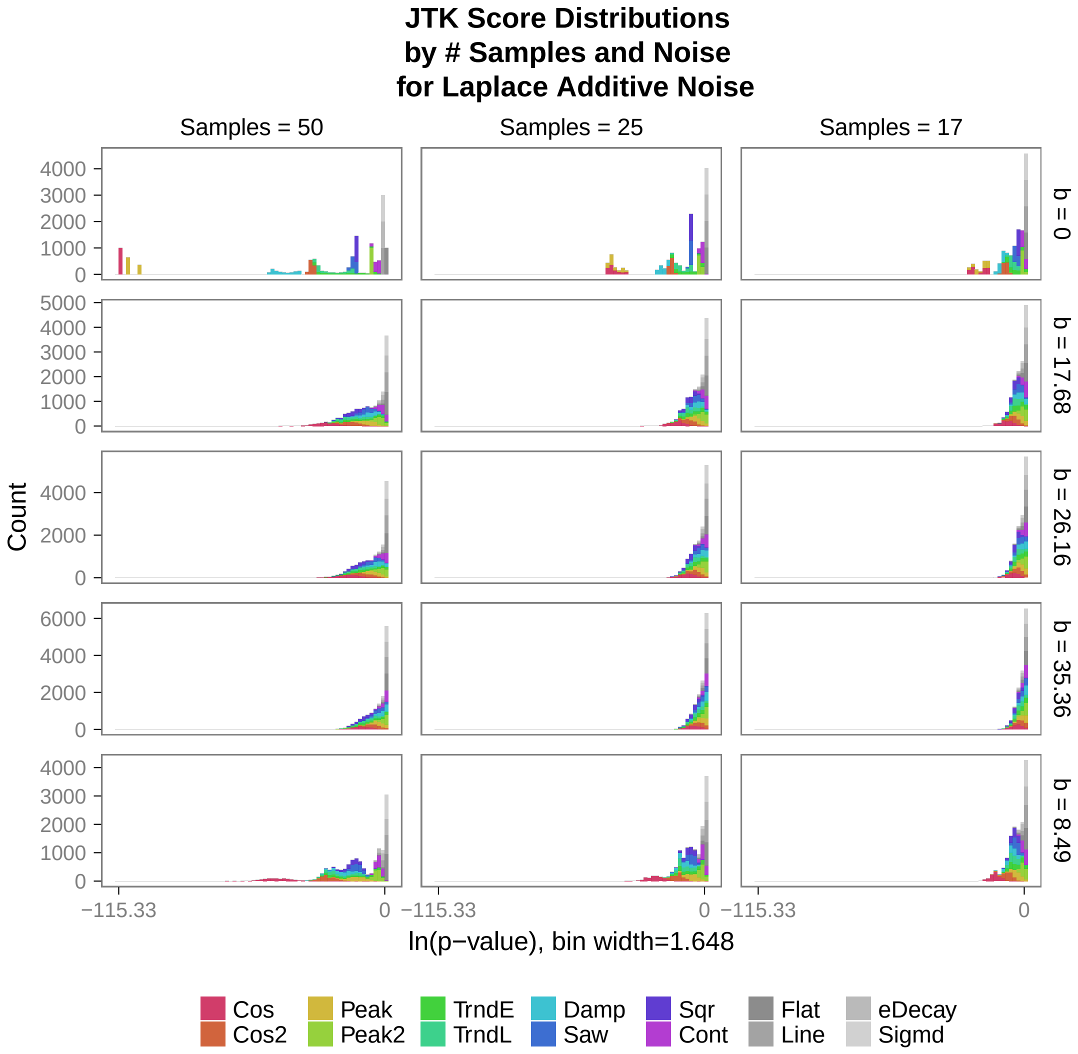

Figure S26: Scores distributions on synthetic data with additive Laplacian noise for JTK.

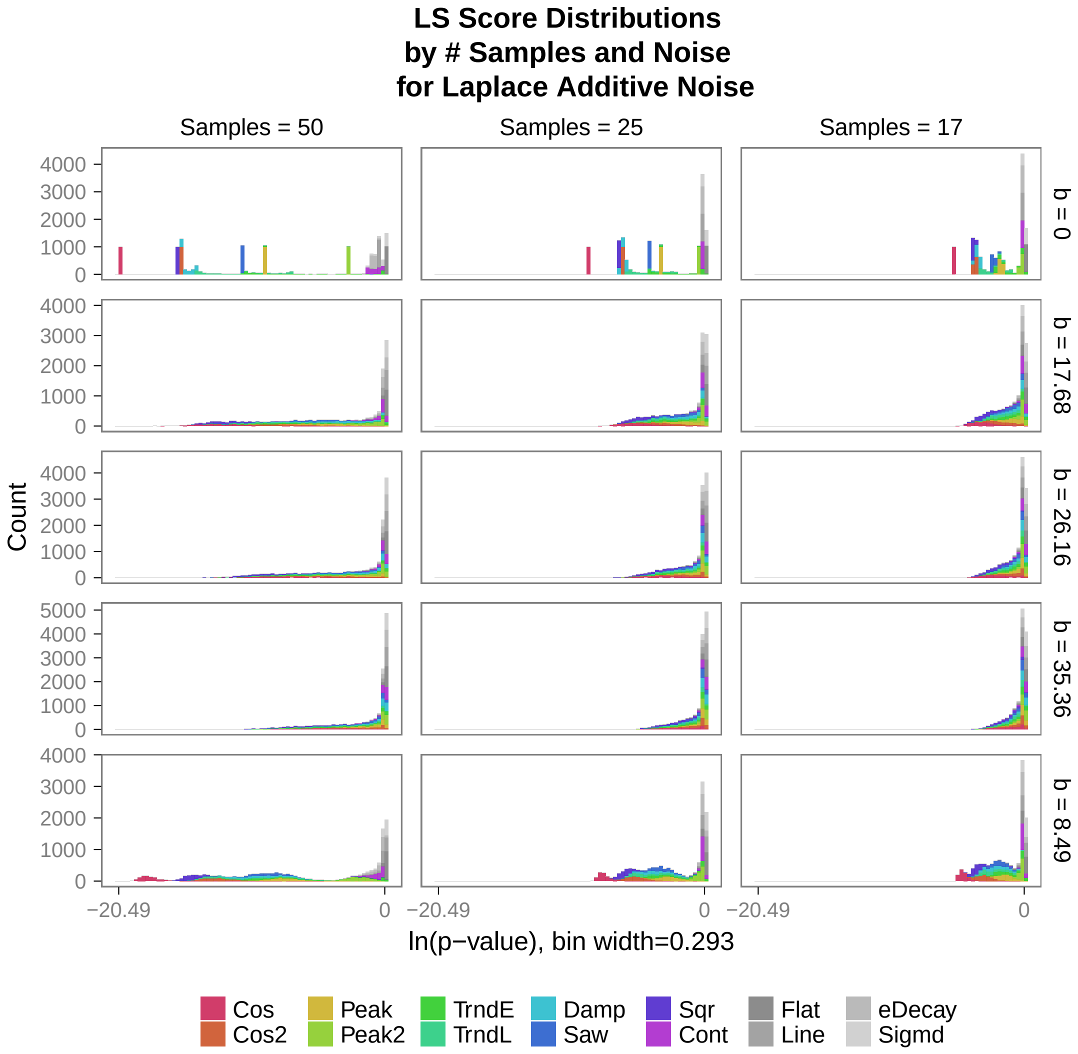

Figure S27: Scores distributions on synthetic data with additive Laplacian noise for Lomb-Scargle.

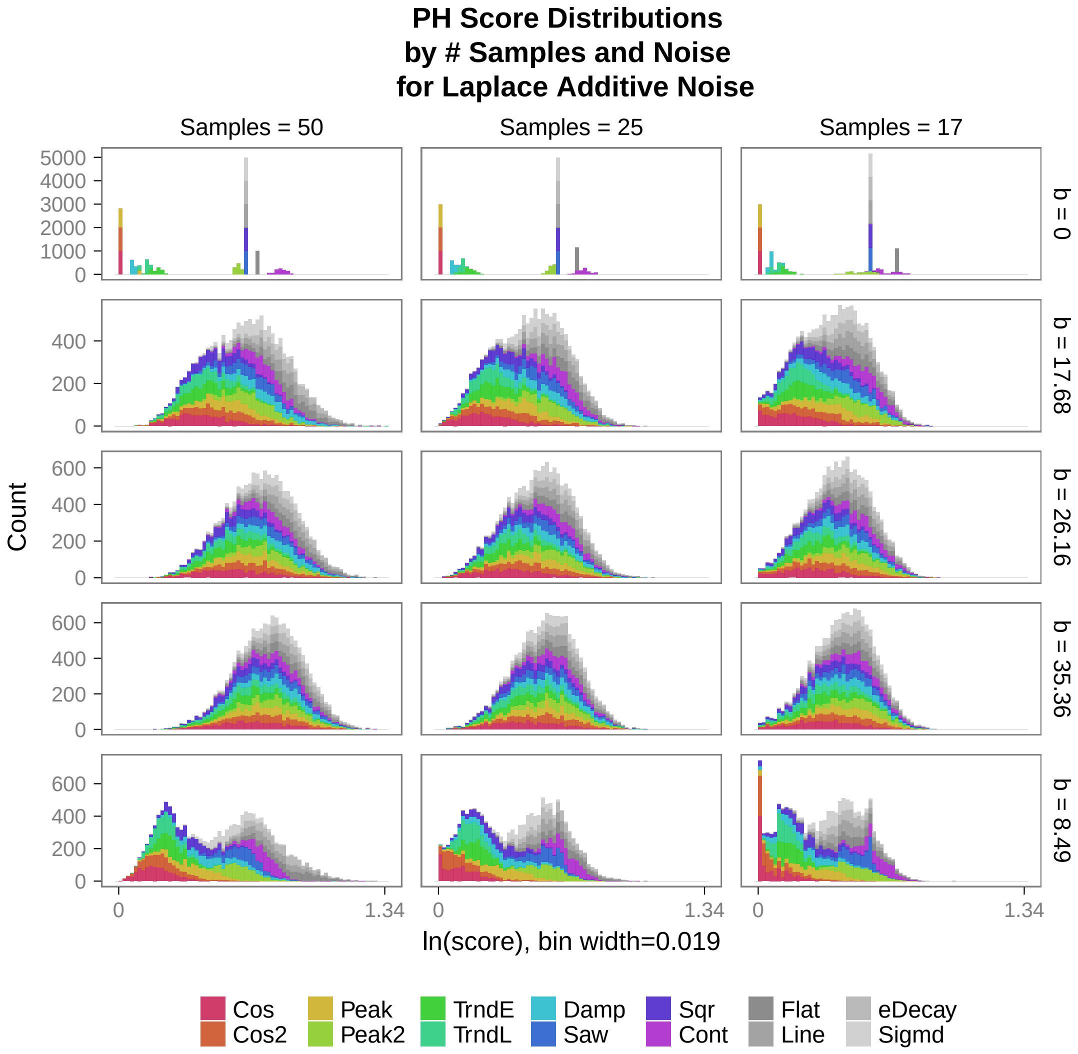

Figure S28: Scores distributions on synthetic data with additive Laplacian noise for PH.

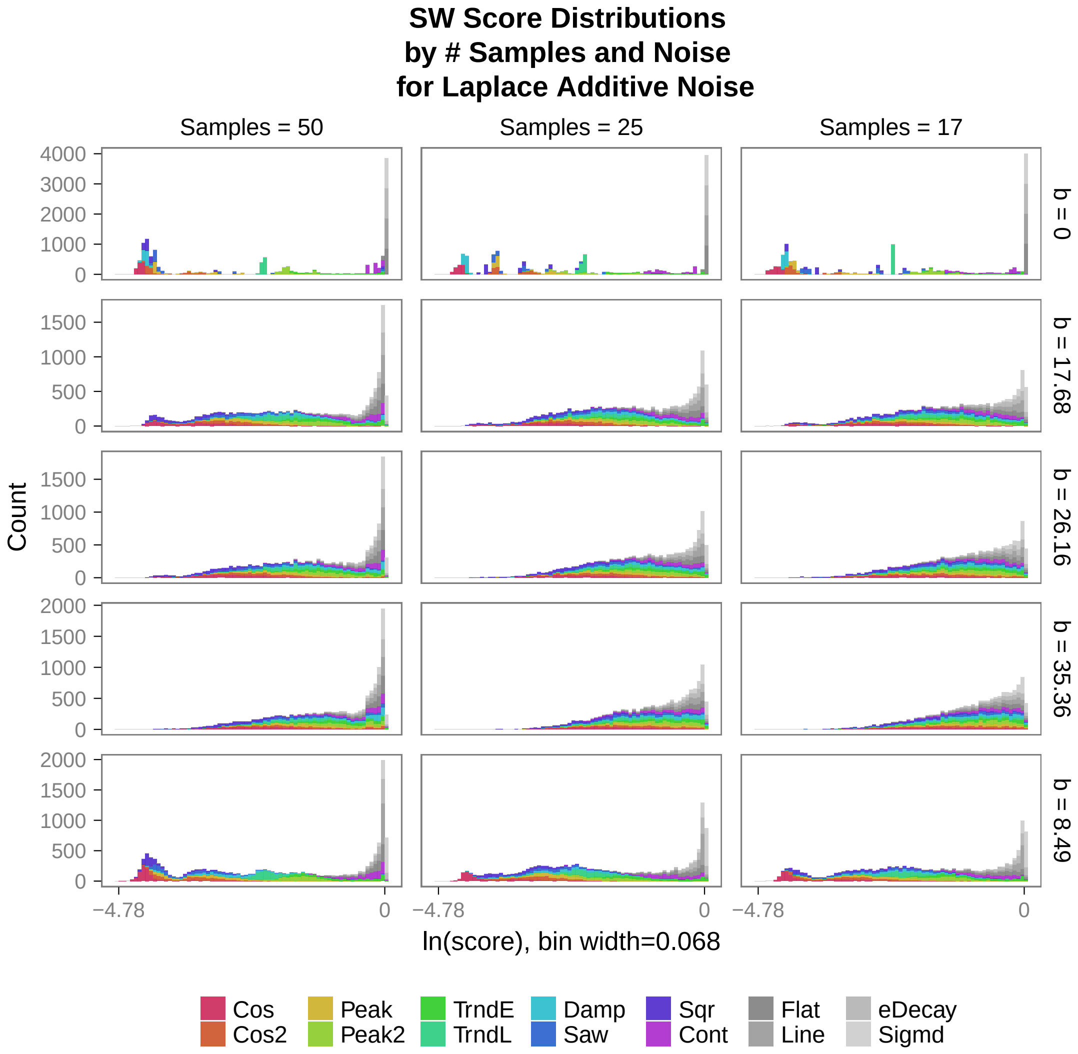

Figure S29: Scores distributions on synthetic data with additive Laplacian noise for SW1perS.

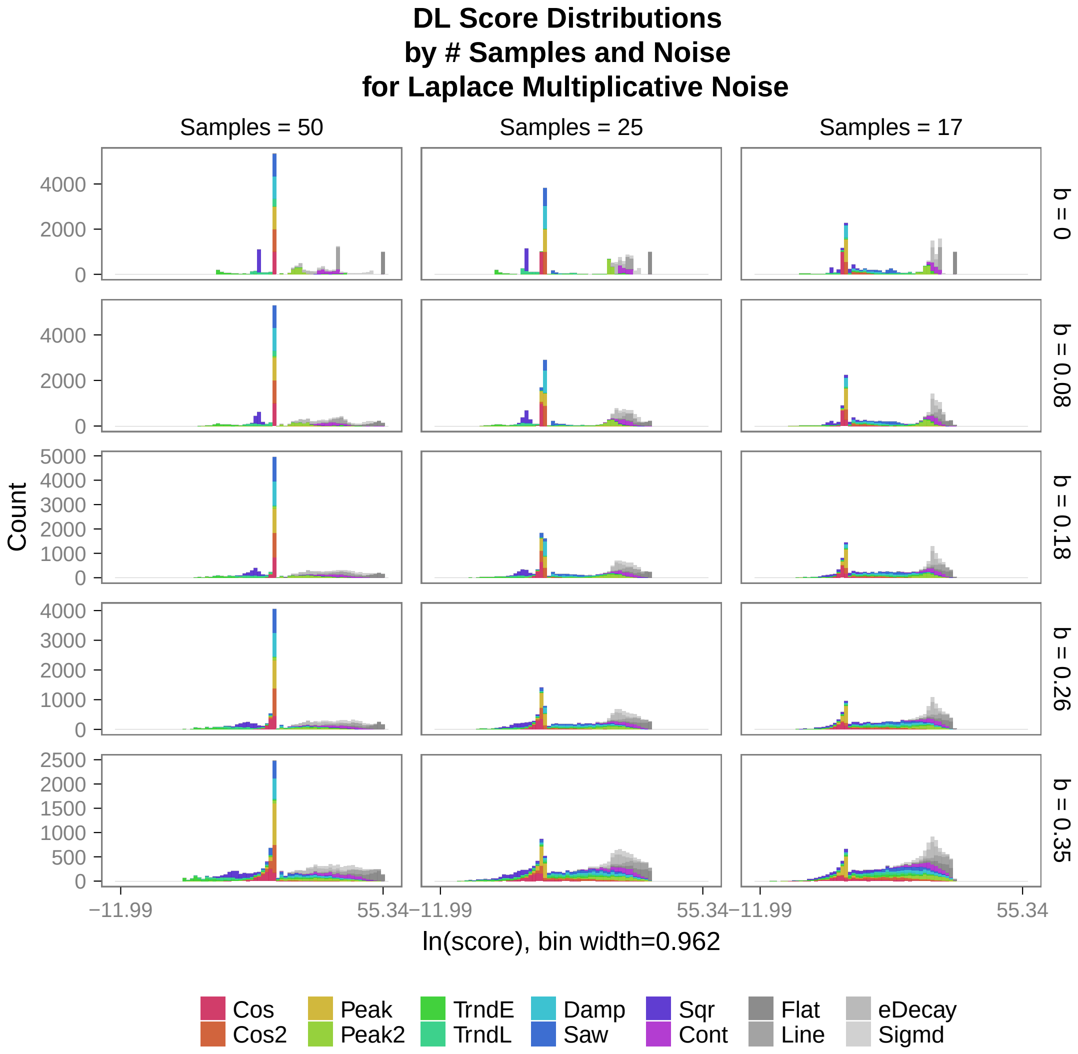

Figure S30: Scores distributions on synthetic data with multiplicative Laplacian noise for de Lichtenberg.

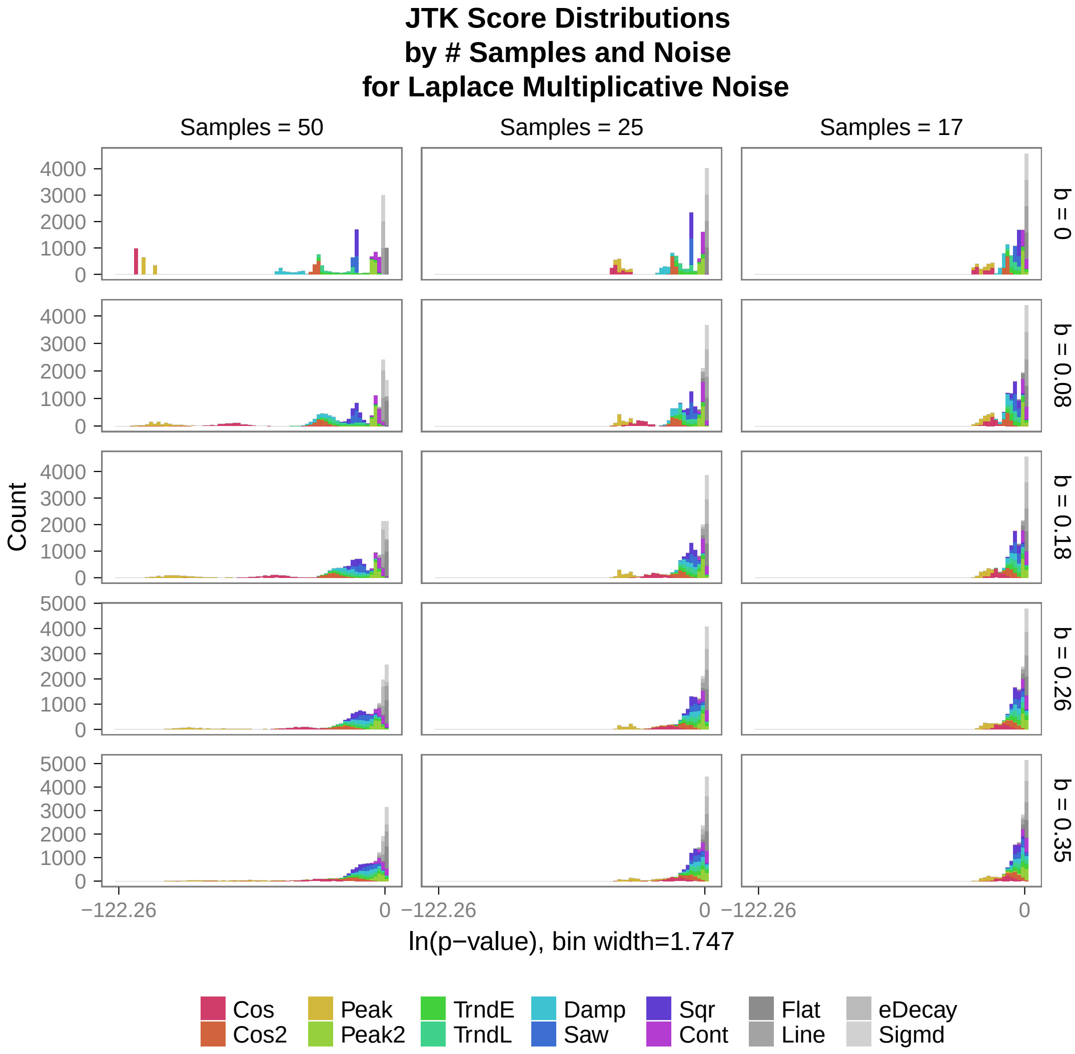

Figure S31: Scores distributions on synthetic data with multiplicative Laplacian noise for JTK.

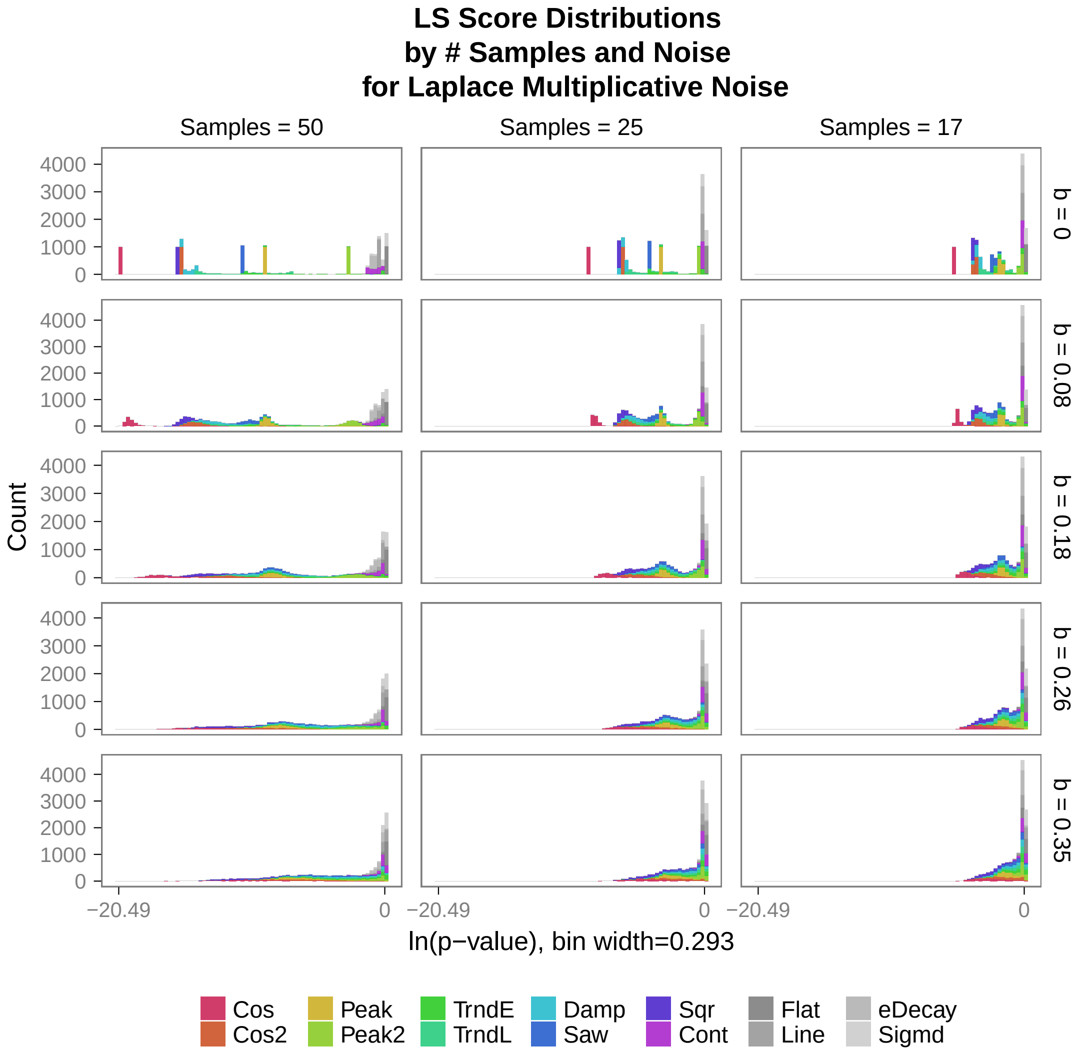

Figure S32: Scores distributions on synthetic data with multiplicative Laplacian noise for Lomb-Scargle.

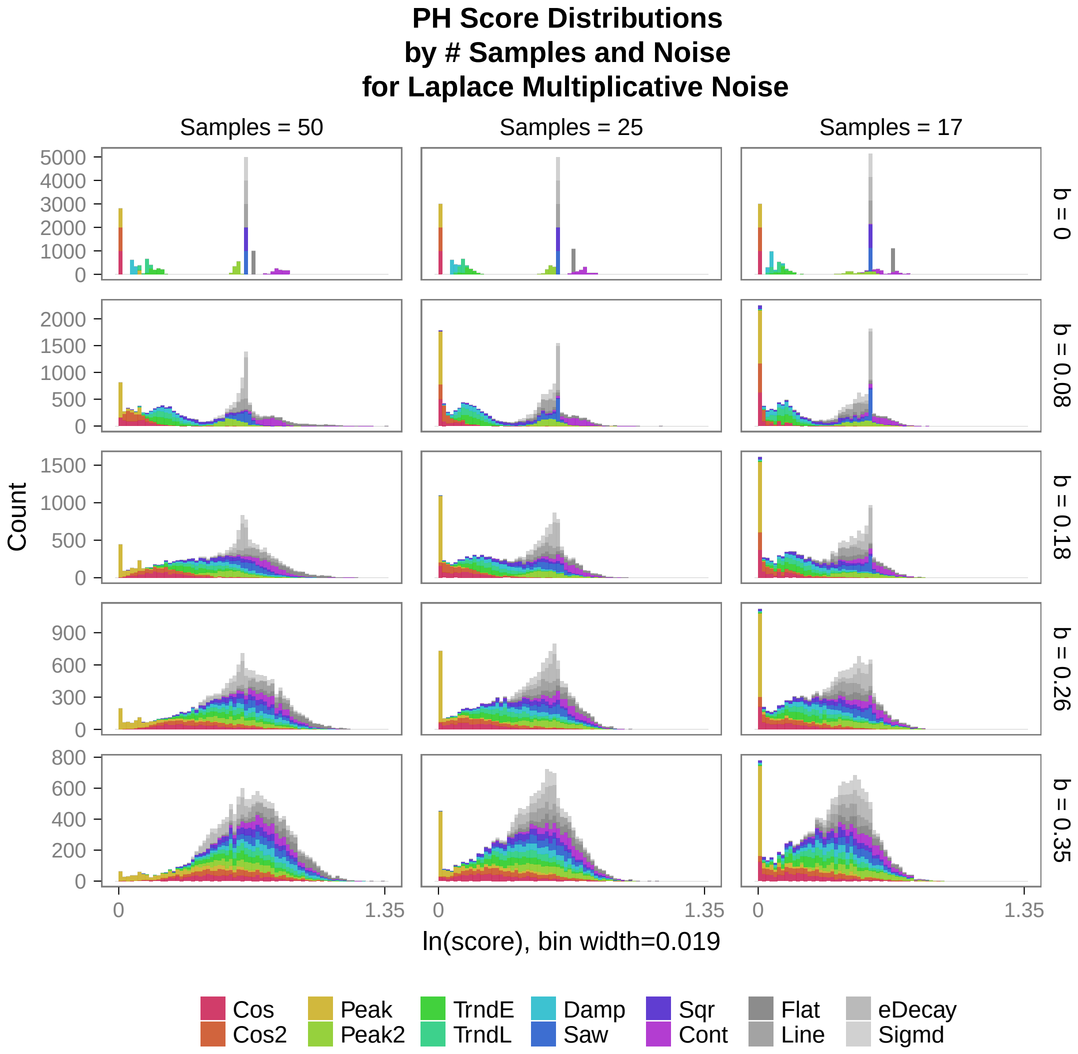

Figure S33: Scores distributions on synthetic data with multiplicative Laplacian noise for PH.

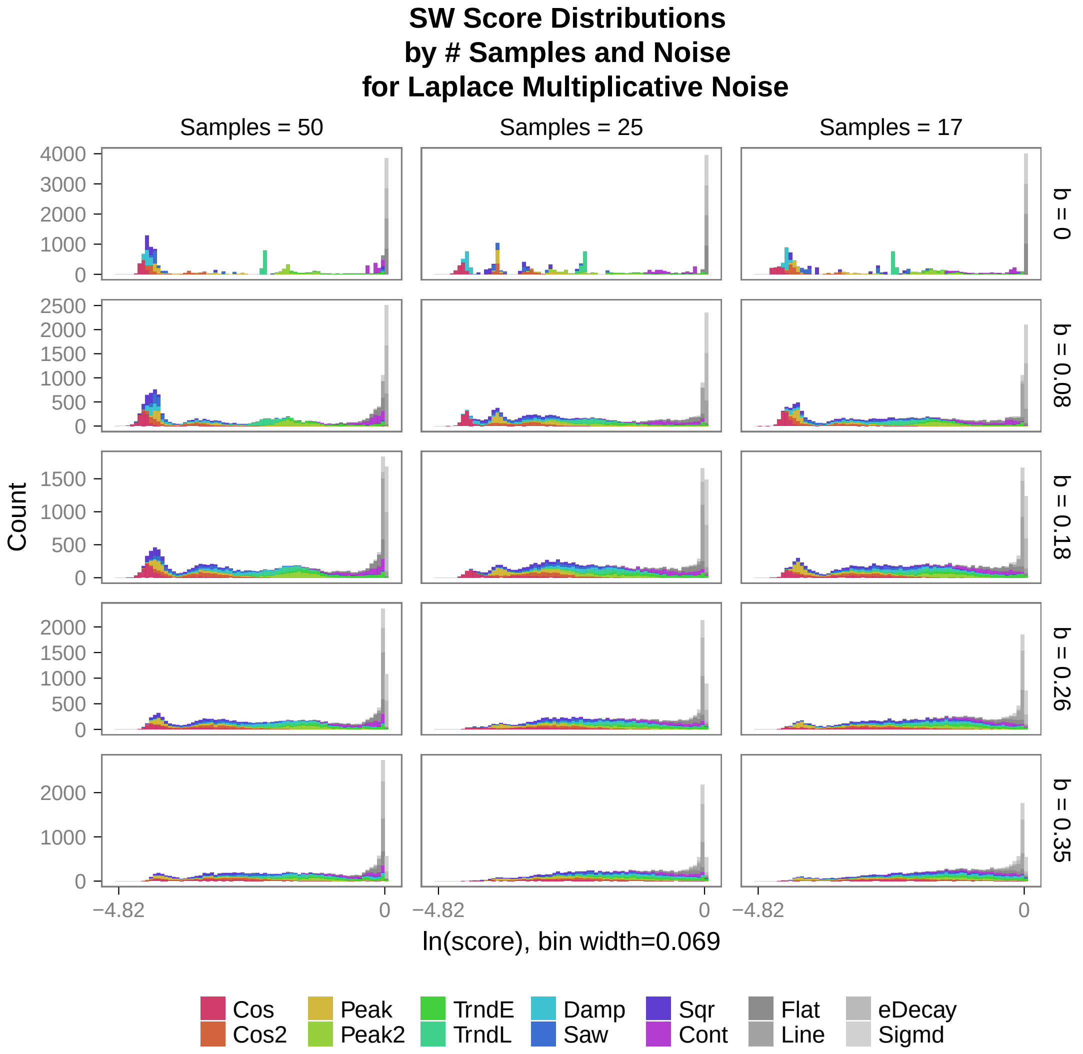

Figure S34: Scores distributions on synthetic data with multiplicative Laplacian noise for SW1PerS.

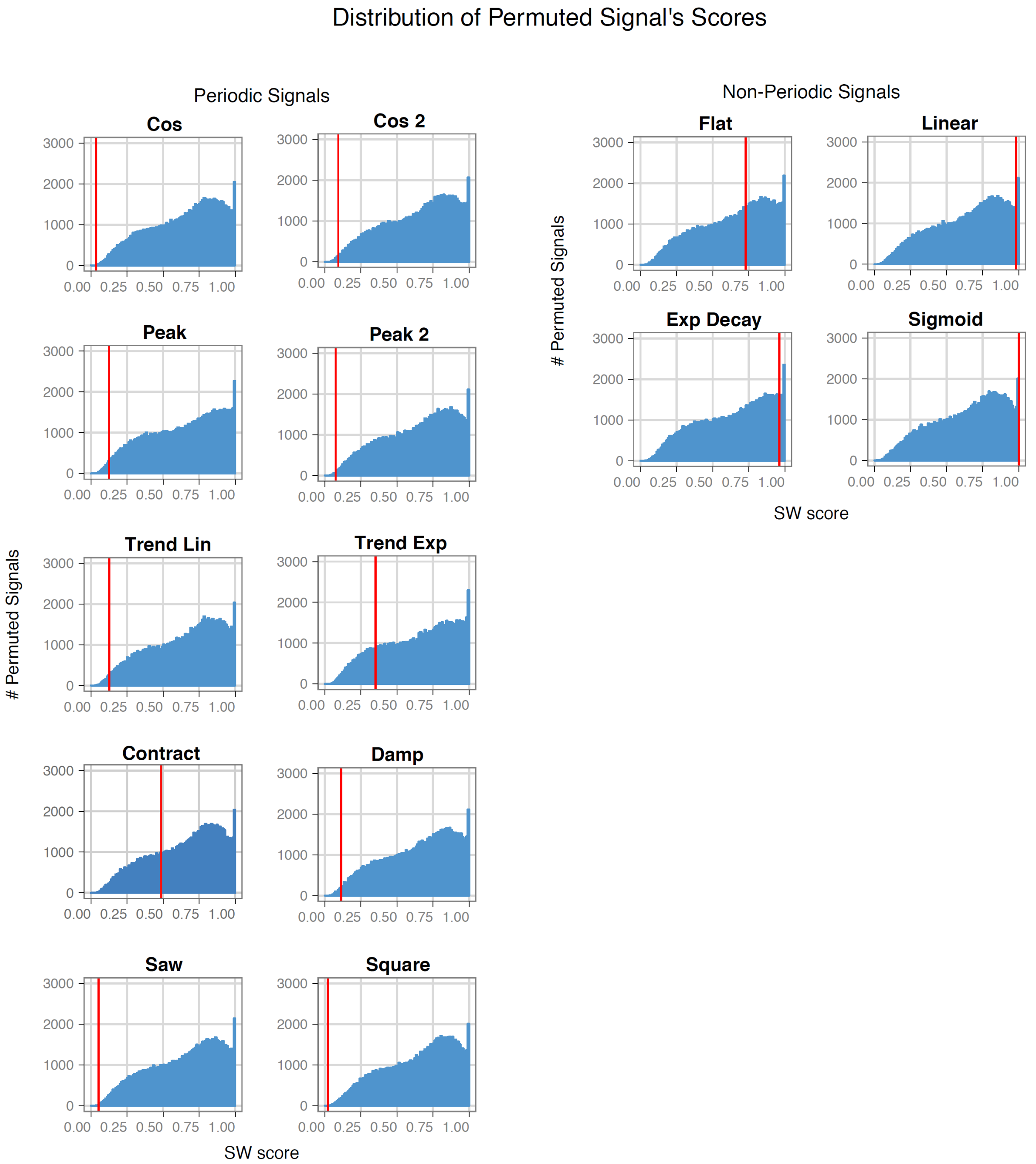

Figure S35: Distributions of scores for permuted signals. The red bar denotes the score for the original signal.

6 Biological Data

Note PH was omitted from further analysis on biological data.

The wild-type data (WT) from Orlando *et al.* (2008) shows periodic gene expression during the cell division cycle (CDC) in budding yeast, *S. cerevisiae*.

The yeast metabolic cycle (YMC) data of Tu *et al.* (2005) are from *S. cerevisiae*.

The mammal circadian rhythm data from Hughes *et al.* (2009) is from liver samples from wild-type mice.

Affymetrix probes were mapped to genes using the Affymetrix Annotations Release 32, June 2011.
YG_S98 Annotations, CSV format, Release 32 (3 MB 06/10/11):
http://www.affymetrix.com/Auth/analysis/downloads/na32/ivt/YG_S98.na32.annot.csv.zip
Yeast_2 Annotations, CSV format, Release 32 (3.6 MB, 6/10/11):
http://www.affymetrix.com/analysis/downloads/na32/ivt/Yeast_2.na32.annot.csv.zip
Mouse430_2 Annotations, CSV format, Release 32 (16 MB, 6/9/11):
http://www.affymetrix.com/analysis/downloads/na32/ivt/Mouse430_2.na32.annot.csv.zip

**Algorithm**	**Data Set**	**Parameters**
SW	Yeast Cell Cycle	feature.type: 3, nT: 200, allow.Trending: 1, ms.epsilon: 1 - cos(pi/16), ma.movingWindow: 3
LS	Yeast Cell Cycle	per_min: 64, per_max: 112, test_freq: 4
JTK	Yeast Cell Cycle	per_min: 64, per_max: 112, interval: 16
DL	Yeast Cell Cycle	num_permutations: 10000, period: 97.8
SW	Yeast Metabolic Cycle	feature.type: 3, nT: 200, allow.Trending: 1, ms.epsilon: 1 - cos(pi/16), ma.movingWindow: 7
LS	Yeast Metabolic Cycle	per_min: 264, per_max: 360, test_freq: 4
JTK	Yeast Metabolic Cycle	per_min: 264, per_max: 360, interval: 24
DL	Yeast Metabolic Cycle	period: 300, num_permutations: 10000
SW	Mammal Circadian	feature.type: 3, nT: 200, allow.Trending: 1, ms.epsilon: 1 - cos(pi/16), ma.movingWindow: 10
LS	Mammal Circadian	min_per: 20, max_per: 28, test_freq: 4
JTK	Mammal Circadian	per_min: 20, per_max: 28, interval: 1
DL	Mammal Circadian	num_permutations: 10000, period: 24

Table S3: Running the Algorithms on Biological Data. For each algorithm and data set, the parameters used to run the algorithm are listed. For SW1perS, we found these parameters to give the best average results on the synthetic data. For the Moving Average (ma), the size of the window is the number of samples divided by five.

symbol	SW_rank	DL_rank	LS_rank	JTK_rank	Max - Min	Norm Plot
SKI7	1	157	2	2.5	154.9018	
GGA2	2	638	505	940	987.3183	
CRH1	3	440	313.5	940	5796.019	
SBH1	4	142	41	36	2018.18	
WHI3	5	54	124	281	1640.201	
CSH1	6	543	694	1207	447.1636	
GDT1	7	212	165.5	1207	3376.449	
ZRG17	8	2516	441	281	300.9961	
AIM22	9	198	80.5	153	84.61007	
CDC11	10	31	283	681	963.0563	
PPH21	11	137	51	9	987.4555	
YRF1-(1-8)	13	185	1583	681	941.6383	
HEM1	14	561	650	940	974.6055	
HIS2	15	390	1482.5	681	521.3636	
MYO2	16	85	111	153	3584.211	
YOX1	17	76	1414	457.5	2123.593	
HAC1	18	1646	1472	2600	1845.381	
SLM5	19	235	84	153	172.0444	
PUS5	20	1582	694	153	106.7517	
ALR2	22	98	405	9	155.2843	
GIC2	23	338	1544	457.5	2361.804	
YRF1-(1-8)	24.5	330	1323	681	3202.917	
ETP1	26	1279	1614	2600	29.74337	
PHO87	27	657	306	681	587.7062	
NHP6A	28	666	558.5	457.5	2352.713	

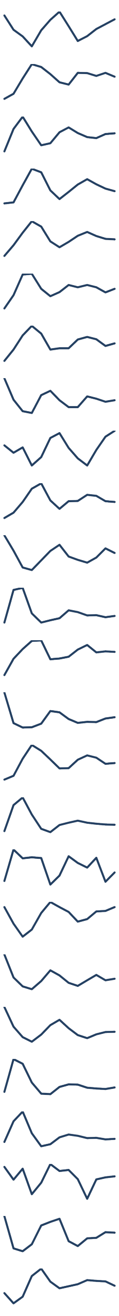

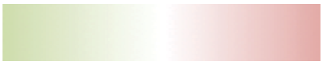

Figure S36: Top 25 results from SW1perS for yeast cell cycle data. Shown by rank against the rankings from DL, LS, and JTK. Any blank symbols were omitted.

symbol	SW_rank	DL_rank	LS_rank	JTK_rank	Max - Min	Norm Plot
YAH1	1	1112	86.5	93.5	14.12	
ADH1	2	1941	154.5	365	49.29	
MYO2	3	3282.5	252	365	5.89	
ALD3	4	125.5	20	53.5	3.40	
FTR1	5	1742	64	145.5	8.18	
GAS3	6	821	323	100.5	5.80	
SCW10	7	76.5	178.5	87.5	17.10	
HHT1	8	1342	119.5	763.5	40.54	
CST9	9	1705	293	763.5	1.16	
PMT4	10	868	29	138.5	7.07	
TOM22	11	2450.5	560	435.5	7.60	
YRO2	12.5	120	55	138.5	26.38	
SIL1	12.5	2236.5	114	435.5	1.25	
IRA2	14	2161.5	52	235.5	6.76	
COP1	15	3060.5	232	601.5	5.25	
AIM37	16	2171.5	292	34.5	8.55	
ACH1	17	776.5	415.5	343	39.87	
TIM23	18	2332.5	26	365	8.28	
DSF2	19	552	111.5	601.5	1.44	
RPS22B	20	177.5	697	632	43.47	
TOM20	21	1355.5	77.5	210.5	9.16	
PMT1	22	2233	645	1036.5	3.88	
MRM1	23	2421	14	182.5	3.19	
GAS1	24	1147	520.5	1085	39.94	
HTB2	25	52	46.5	6.5	20.65	

Best score — 50 percentile — Worst score

Largest amplitude — 50 percentile — Smallest amplitude

Figure S37: Top 25 results from SW1perS for yeast metabolic data. Shown by rank against the rankings from DL, LS, and JTK. Any blank symbols were omitted.

symbol	SW_rank	DL_rank	LS_rank	JTK_rank	Max - Min	Norm Plot
Fgf1	1	1451.5	39	116	10916.2	
Nr1d2	2	26.5	10	25.5	62708.9	
Nampt	3	242.5	48	5	17275.4	
Azin1	4	4053.5	134	121	8391.4	
Mreg	6.5	1534.5	38	34.5	30373.3	
Elovl3	6.5	26.5	1	1	149978.3	
Coq10b	8	62	120	100	14706.4	
Arntl	9	26.5	7	48.5	5154.5	
Kynu	10	2102	50	155.5	19870.7	
Insig2	11	201	18	67.5	52612	
Upp2	12	201	53	20	2706.5	
Gde1	13	1242	73	144.5	12204.3	
St3gal5	14	254	65	3	17587.6	
Grk5	15	56	177	181.5	1002	
Homer2	16	804.5	24	121	6225.1	
Rnf125	17	107	57	144.5	101512.1	
Slc30a10	18	212.5	69.5	78	5151.2	
Avpr1a	19	144.5	111	126	5018.8	
Abhd6	20	2289.5	49	64.5	15746.9	
Slc37a4	21	1114	9	24	30147.5	
C730049O14Rik	22	185	23	51.5	122859.7	
Abcg5	23	681.5	35	126	15615.6	
1110067D22Rik	24	56	11	32	8859.2	
Vps4b	25	4711	170	197.5	15520.2	
Gys2	27	532.5	3	6	30658.7	

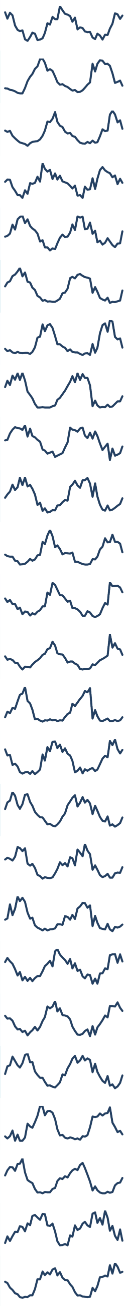

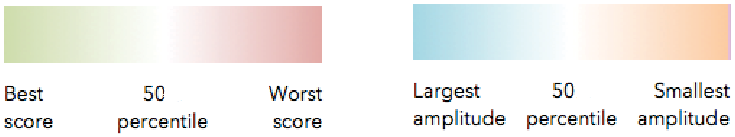

Figure S38: Top 25 results from SW1perS for mammal circadian data. Shown by rank against the rankings from DL, LS, and JTK. Any blank symbols were omitted.

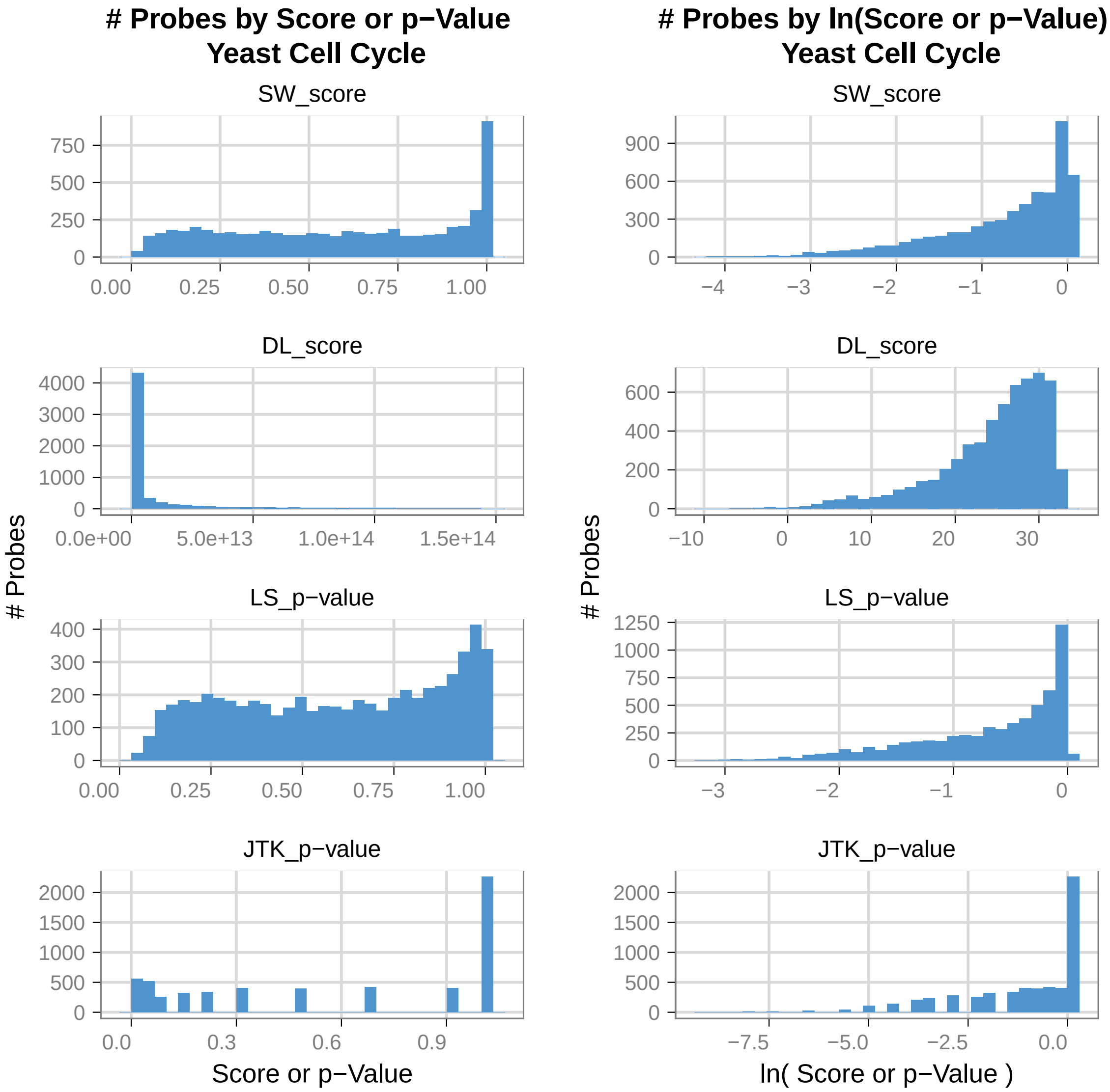

Figure S39: Histogram of number of probes by score or p-value for each algorithm on the Yeast Cell Cycle data set (Orlando *et al.*, 2008). Also shown with the ln(score or p-value).

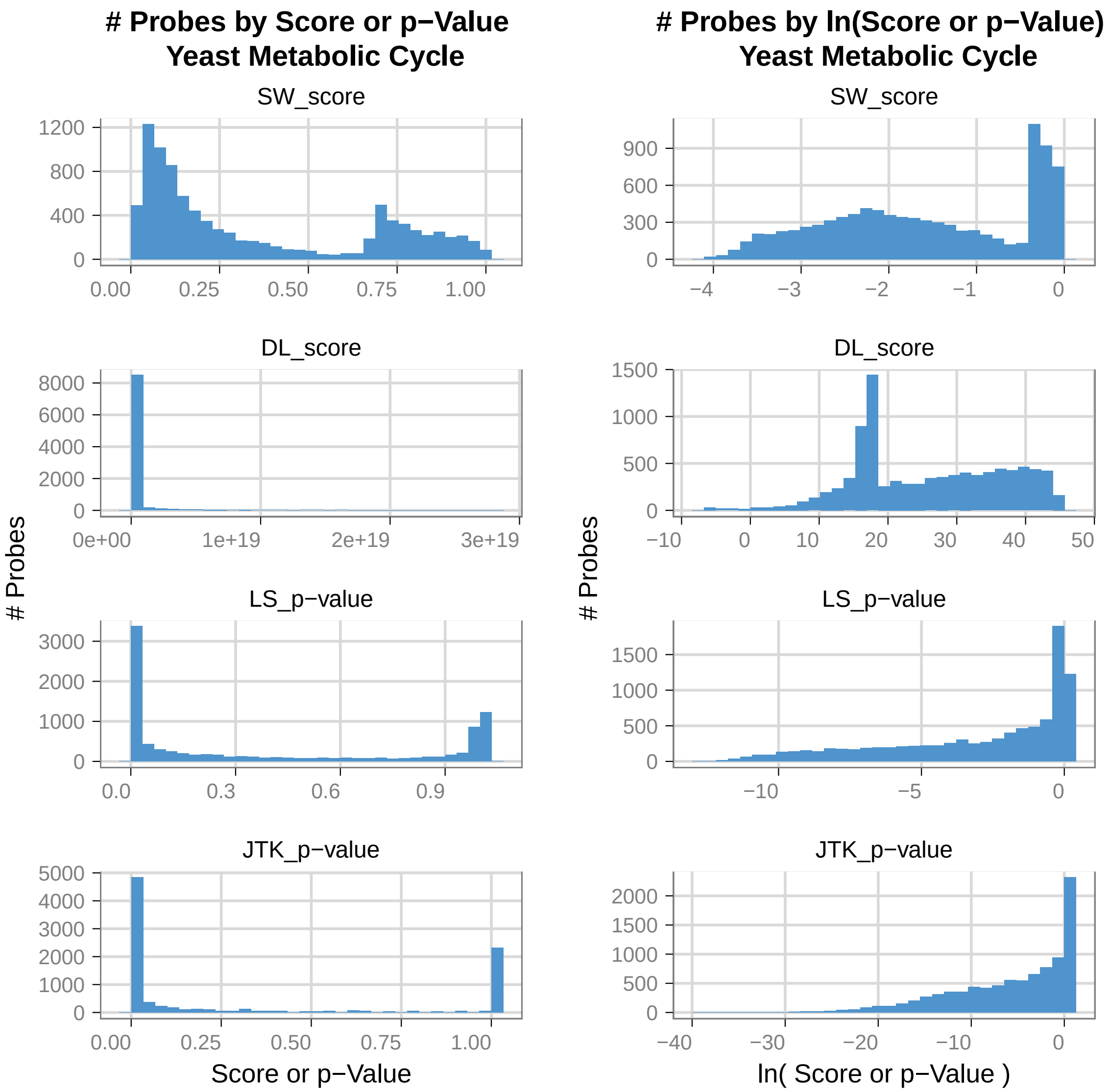

Figure S40: Histogram of number of probes by score or p-value for each algorithm on the Yeast Metabolic Cycle data set (Tu *et al.*, 2005). Also shown with the ln(score or p-value).

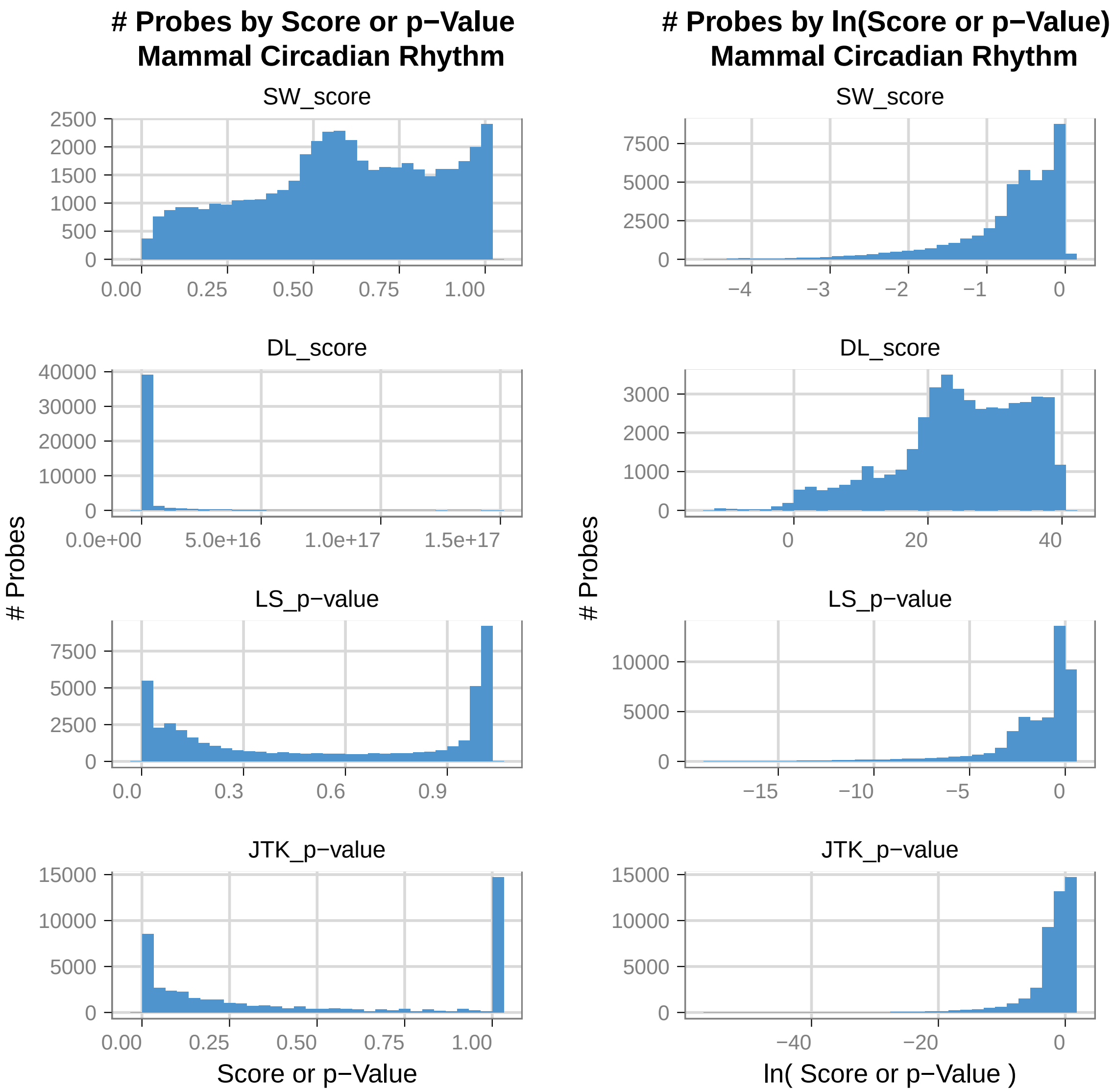

Figure S41: Histogram of number of probes by score or p-value for each algorithm on the Mammal Circadian Rhythm data set (Hughes *et al.*, 2009). Also shown with the ln(score or p-value).

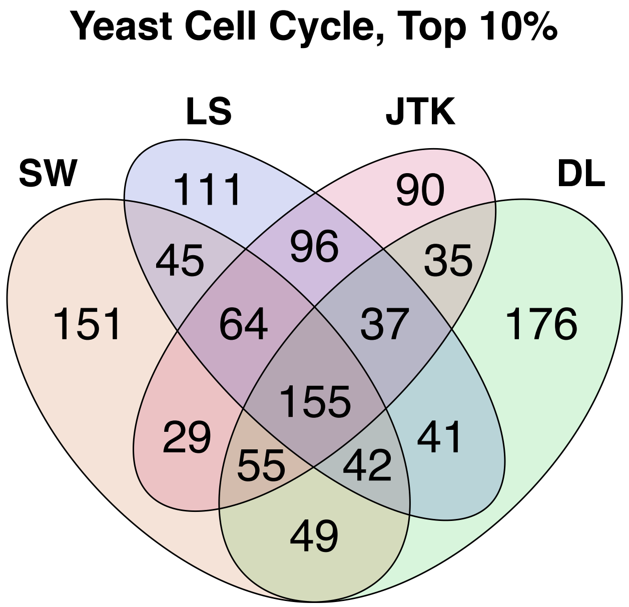

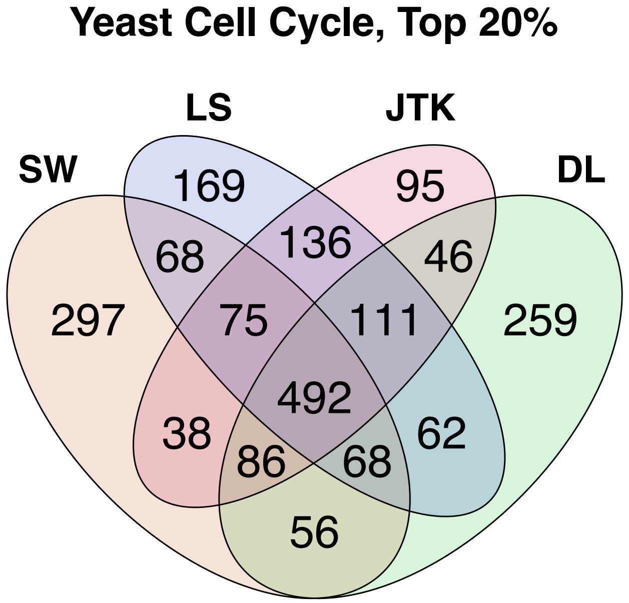

Figure S42: Yeast Cell Cycle overlap of probes in the top 10% and 20% (out of 5,900 probes) by each algorithm.

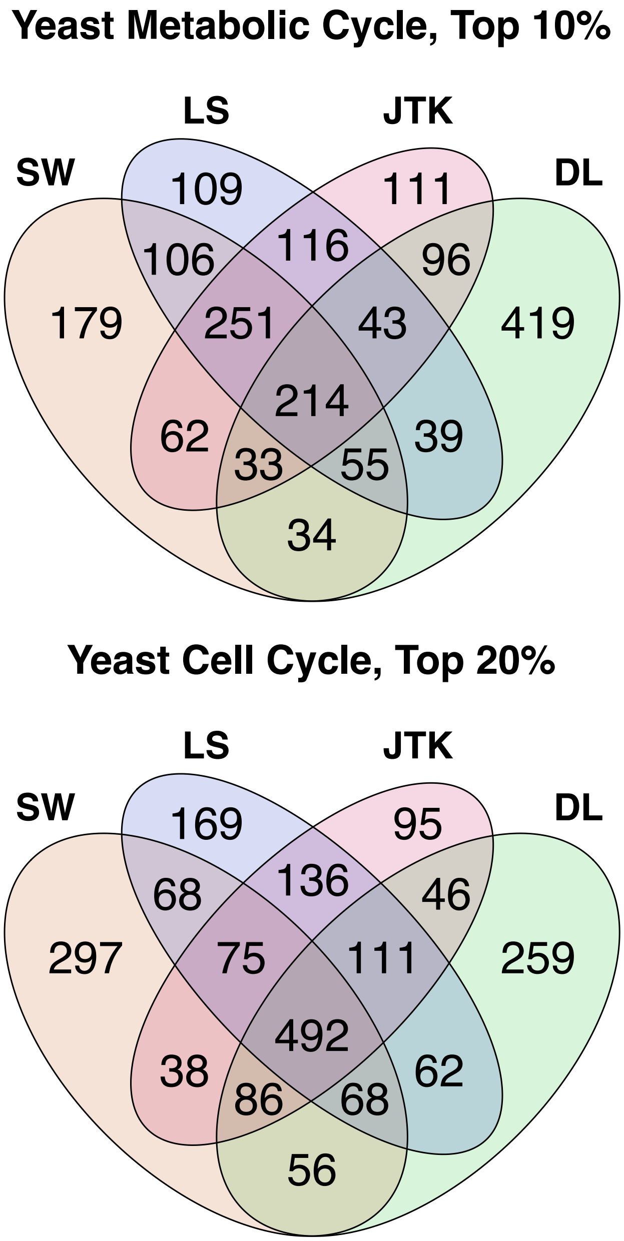

Figure S43: Yeast Metabolic Cycle overlap of probes in the top 10% and 20% (out of 9,335 probes) by each algorithm.

Mammal Circadian Rhythm, Top 5%

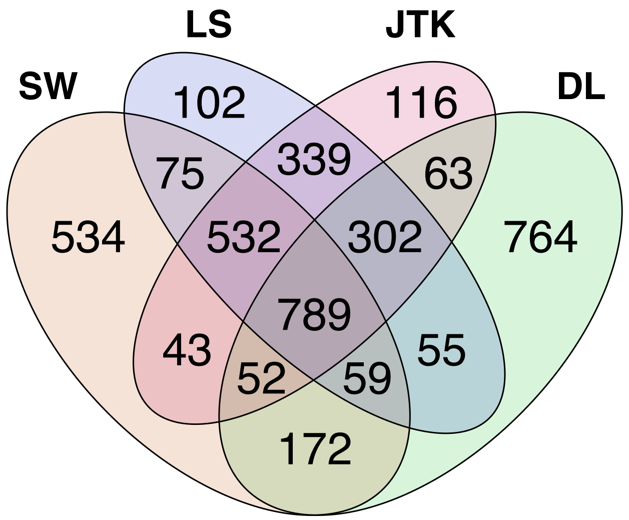

Mammal Circadian Rhythm, Top 10%

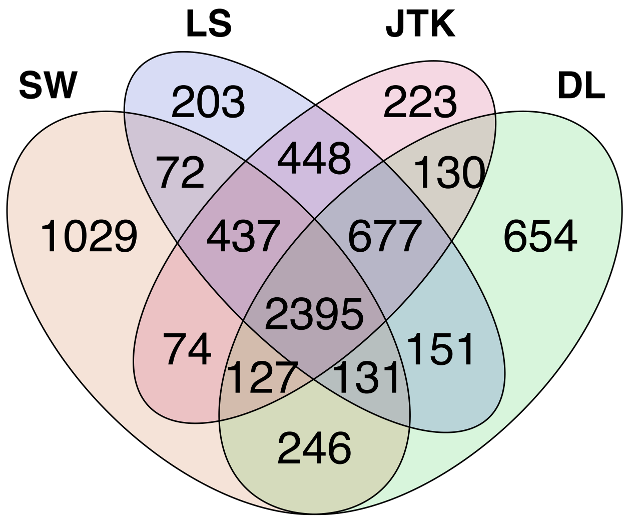

Figure S44: Mammal Circadian overlap of probes in the top 5% and 10% (out of 45,101 probes) by each algorithm.

Yeast Cell Cycle The number of genes / rank in different sets.

**Top #**	**590**	**1180**	**1770**	**2360**	**2950**	**3540**	**4130**	**4720**	**5310**	**5900**
**Top %**	**10**	**20**	**30**	**40**	**50**	**60**	**70**	**80**	**90**	**100**
SW and DL	0.51	0.59	0.66	0.7	0.73	0.76	0.79	0.85	0.91	100
SW and LS	0.52	0.60	0.68	0.73	0.77	0.81	0.85	0.89	0.92	100
SW and JTK	0.51	0.59	0.68	0.75	0.75	0.83	0.79	0.73	0.99	100
ALL	0.26	0.42	0.51	0.57	0.60	0.65	0.65	0.65	0.86	100
SW and (JTK or LS or DL)	0.74	0.75	0.82	0.86	0.89	0.92	0.94	0.96	0.99	100
JTK and (SW or LS or DL)	0.80	0.83	0.88	0.96	0.92	0.99	0.87	0.77	1.1	100
LS and (JTK or SW or DL)	0.81	0.86	0.89	0.94	0.95	0.97	0.97	0.98	100	100
DL and (JTK or LS or SW)	0.7	0.78	0.81	0.84	0.85	0.88	0.89	0.93	100	100

Table S4: Yeast Cell Cycle. Percent of overlap of the top percents of probes from the algorithms.

Yeast Metabolic Cycle The number of genes / rank in different sets.

**Top #**	**933**	**1866**	**2799**	**3732**	**4665**	**5598**	**6531**	**7464**	**8397**	**9330**
**Top %**	**0.1**	**0.2**	**0.3**	**0.4**	**0.5**	**0.6**	**0.7**	**0.8**	**0.9**	**1**
SW and DL	0.36	0.56	0.74	0.83	0.83	0.83	0.84	0.88	0.93	1
SW and LS	0.67	0.78	0.8	0.81	0.83	0.85	0.87	0.91	0.92	1
SW and JTK	0.6	0.73	0.8	0.83	0.86	0.85	0.87	0.87	1	1
ALL	0.23	0.42	0.6	0.71	0.76	0.78	0.8	0.83	0.89	1
SW and (JTK or LS or DL)	0.81	0.88	0.9	0.9	0.9	0.9	0.92	0.95	1	1
JTK and (SW or LS or DL)	0.87	0.96	0.98	0.98	0.99	0.98	0.98	0.93	1.08	1
LS and (JTK or SW or DL)	0.88	0.94	0.95	0.95	0.97	0.98	0.99	0.99	0.97	1
DL and (JTK or LS or SW)	0.55	0.76	0.9	0.96	0.94	0.93	0.93	0.95	1	1

Table S5: Yeast Metabolic Cycle. Percent of overlap of the top percents of probes from the algorithms.

Mammal Circadian Rhythm The number of genes / rank in different sets.

**Top #**	**4510**	**9020**	**13530**	**18040**	**22550**	**27060**	**31570**	**36080**	**40590**	**45100**
**Top %**	**0.1**	**0.2**	**0.3**	**0.4**	**0.5**	**0.6**	**0.7**	**0.8**	**0.9**	**1**
SW and DL	0.64	0.68	0.65	0.68	0.74	0.8	0.84	0.88	0.92	1
SW and LS	0.67	0.59	0.57	0.64	0.74	0.8	0.85	0.88	1	1
SW and JTK	0.67	0.66	0.65	0.68	0.73	0.78	0.8	0.76	1	1
ALL	0.53	0.55	0.51	0.56	0.63	0.7	0.73	0.72	0.92	1
SW and (JTK or LS or DL)	0.77	0.75	0.74	0.79	0.84	0.88	0.91	0.94	1	1
JTK and (SW or LS or DL)	0.95	0.9	0.89	0.91	0.92	0.92	0.91	0.82	1.11	1
LS and (JTK or SW or DL)	0.95	0.84	0.87	0.92	0.95	0.97	0.98	0.98	1.11	1
DL and (JTK or LS or SW)	0.85	0.88	0.91	0.94	0.95	0.96	0.97	0.97	1	1

Table S6: Mammal Circadian Rhythm. Percent of overlap of the top percents of probes from the algorithms.

7 Gene lists from ChIP-chip and ChIP-seq Data

For the yeast cell cycle, the ChIP-chip data of Simon *et al.* (2001) used nine known cell cycle transcription factors: Mbp1, Swi4, Swi6, Mcm1, Fkh1, Fkh2, Ndd1, Swi5, and Ace2. Their list of selected targets in table 1 was used.

For the mouse circadian rhythm, the Chip-seq data of Koike *et al.* (2012) used seven known circadian transcription factors: BMAL1, CLOCK, NPAS2, PER1, PER2, CRY1, and CRY2. Their study included measurements for six time points; we selected the genes that had the largest difference (greater than 50) in tag counts for each transcription factor/target pair.

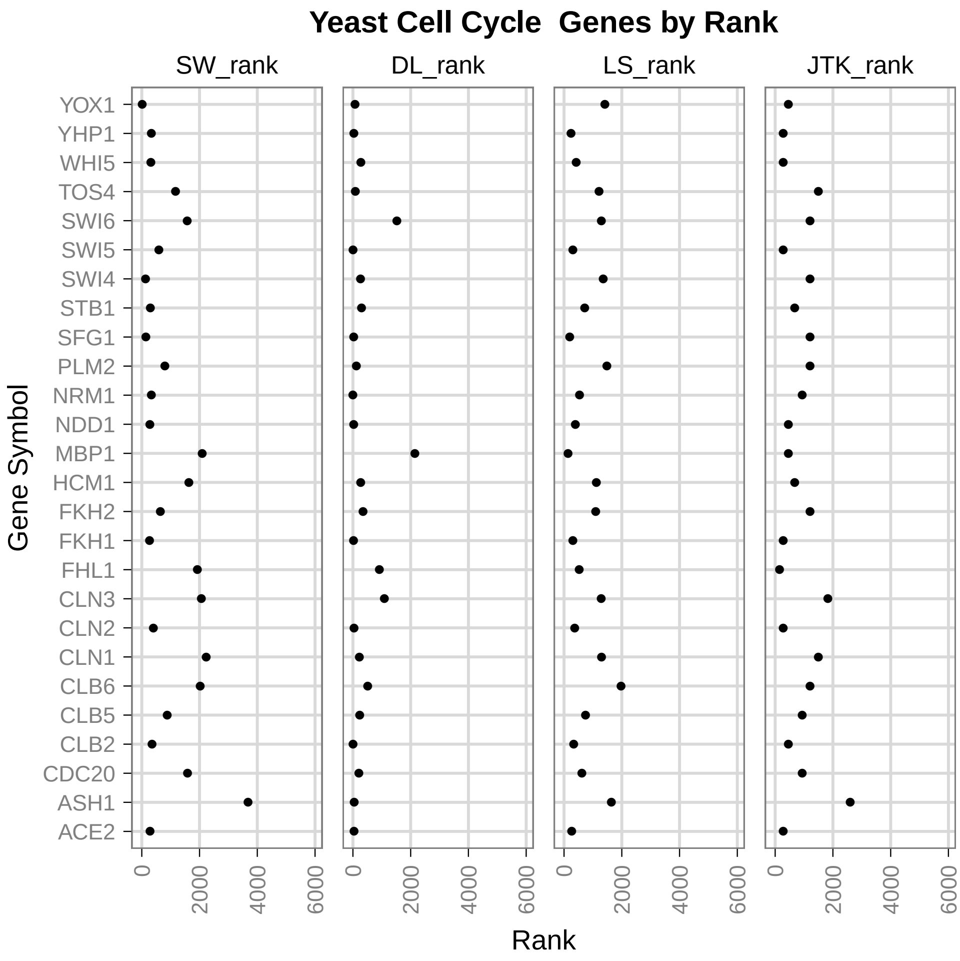

Figure S45: Compiled list of known genes for the yeast cell cycle, and their ranking by each algorithm.

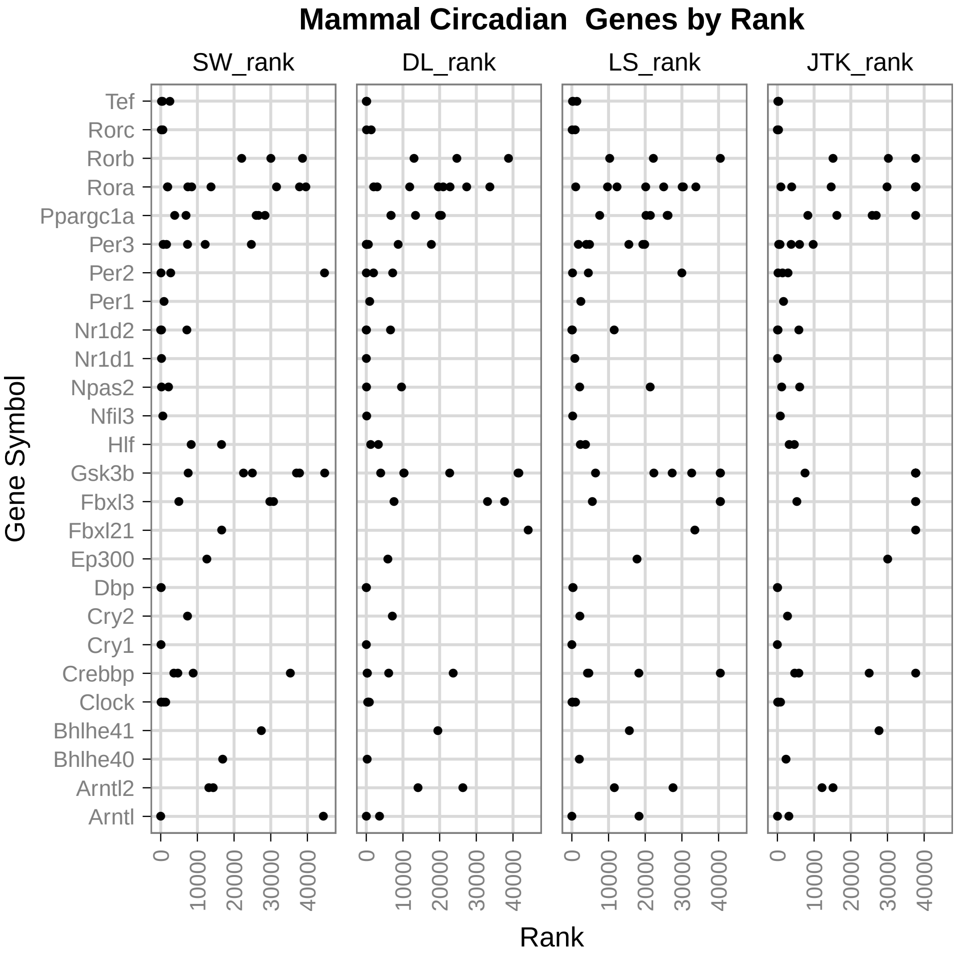

Figure S46: Compiled list of known genes for the mammal circadian rhythm, and their ranking by each algorithm. Some genes have multiple probes, and some probes appear less periodic.

8 Filtering Noise using Replicates

The combined score was the JTK score multiplied by the SW difference score. The SW difference score was the absolute difference between the SW score on replicate one and the SW score on replicate two. This assumes that two signals that are both considered very periodic are less likely to be noise. The JTK score was the p-value from a modified version of JTK that compared the two replicates. JTK was modified to use the signal from the first replicate as a reference, and the second replicate's signal was compared without applying different periods or phase shifts. This assumes that two signals that have highly correlated shapes are less likely to be noise. The correlation between the signals was computed as in the original version of JTK. The score cutoff of 0.05 was selected by plotting a histogram of the scores.

Yeast Cell Cycle, Top 10% SW, Not Top 10% Others,
Noise Filtered, AND Yeast Metabolic Cycle

sys_name	symbol	SW_rank	LS_rank	JTK_rank	DL_rank	rep_score	Spellman	Orlando	wt1	wt2
YNL042W	BOP3	76.0	657.5	681.0	644.0	1.42e−04				
YBL010C	---	105.0	1498.0	2199.5	1176.0	7.52e−05		x		
YBR046C	ZTA1	185.0	677.0	1207.0	629.0	1.48e−02		x		
YMR226C	---	192.0	2261.0	940.0	937.0	4.61e−02		x		
YOL105C	WSC3	219.0	823.5	1207.0	1168.0	3.52e−05		x		
YCR002C	CDC10	221.0	748.5	1827.0	1224.0	8.37e−03	x			
YDR135C	YCF1	223.0	1958.5	940.0	735.0	2.70e−02				
YLL058W	---	226.0	773.0	1495.0	974.0	1.55e−03		x		
YIL108W	---	242.0	2095.0	1207.0	1361.0	5.25e−05				
YJL197W	UBP12	244.0	992.5	940.0	829.0	2.50e−03				
YLR284C	ECI1	263.0	1213.0	2199.5	1239.0	1.94e−04		x		
YNL191W	DUG3	305.0	2177.0	2199.5	1628.0	6.09e−03				
YLR090W	XDJ1	306.0	1620.5	681.0	1061.0	6.95e−03				
YMR291W	---	312.0	1693.5	3010.5	1183.0	6.07e−03		x		
YGR234W	YHB1	315.0	1645.0	3010.5	1794.0	2.06e−05	x			
YDL102W	POL3	337.0	1990.0	940.0	647.0	1.08e−05	x	x		
YPR114W	---	344.0	1146.0	1207.0	1232.0	2.16e−02				
YNR027W	BUD17	345.5	873.5	940.0	915.0	7.69e−03				
YNL003C	PET8	353.0	1243.0	2199.5	1462.0	1.14e−04	x	x		
YFR028C	CDC14	382.0	1089.0	940.0	1025.0	1.03e−04		x		
YGL022W	STT3	420.0	1081.5	1827.0	692.0	1.92e−05				
YBL015W	ACH1	437.0	640.5	940.0	1926.0	1.96e−03		x		
YOR038C	HIR2	442.0	1419.5	940.0	990.0	9.85e−05				
YDR393W	SHE9	451.0	1656.0	940.0	922.0	3.56e−02				
YBR278W	DPB3	459.0	1759.0	940.0	672.0	1.51e−04		x		
YMR307W	GAS1	462.0	1018.5	940.0	601.0	2.09e−05	x			
YNL323W	LEM3	467.0	664.5	940.0	674.0	1.56e−02				
YMR106C	YKU80	489.0	1711.0	940.0	1542.0	2.20e−03				
YDL239C	ADY3	501.0	1836.0	2199.5	2218.0	3.55e−02				
YLR037C	PAU23	503.0	3117.0	4765.5	3803.0	2.87e−02		x		
YBL036C	---	516.0	1954.5	1495.0	1706.0	1.76e−05				
YDL086W	---	532.0	2060.0	940.0	963.0	1.79e−03				
YKL064W	MNR2	547.0	1267.5	940.0	1126.0	1.58e−02				
YOL058W	ARG1	551.0	645.5	940.0	610.0	9.64e−04	x	x		
YER034W	---	560.0	1880.5	1495.0	2026.0	1.26e−04				
YLR446W	---	578.0	1738.0	3426.0	1595.0	2.84e−03		x		

Figure S47: Yeast Cell Cycle, WT1. Top 10% of SW, not in top 10% of DL, LS, JTK. Filtered for noise using replicates (a combined score from a modified version of JTK and SW). Overlap with yeast metabolic cycle (YMC) from Tu *et al.* (2005). Genes that were in the periodic gene lists from Spellman *et al.* (1998) or Orlando *et al.* (2008) are marked.

Yeast Cell Cycle, Top 10% SW, Not Top 10% Others, Noise Filtered, AND GRR Negative

sys_name	symbol	SW_rank	LS_rank	JTK_rank	DL_rank	rep_score	Spellman	Orlando	wt1	wt2
YPR178W	PRP4	72.0	977.0	940.0	743.0	1.66e–03				
YBR046C	ZTA1	185.0	677.0	1207.0	629.0	1.48e–02		x		
YML020W	–––	209.0	1196.5	1495.0	877.0	4.71e–08	x	x		
YLL058W	–––	226.0	773.0	1495.0	974.0	1.55e–03		x		
YLR284C	ECI1	263.0	1213.0	2199.5	1239.0	1.94e–04		x		
YKR095W	MLP1	348.0	1534.5	1827.0	1954.0	7.00e–04				
YGR196C	FYV8	363.0	664.5	1207.0	1123.0	1.84e–02				
YOR290C	SNF2	391.0	1387.5	681.0	1468.0	4.09e–04				
YBR278W	DPB3	459.0	1759.0	940.0	672.0	1.51e–04		x		
YER007W	PAC2	494.0	1309.0	940.0	970.0	8.36e–04				
YDL239C	ADY3	501.0	1836.0	2199.5	2218.0	3.55e–02				
YKL064W	MNR2	547.0	1267.5	940.0	1126.0	1.58e–02				
YJL047C	RTT101	581.0	1693.5	1207.0	1103.0	4.61e–03				

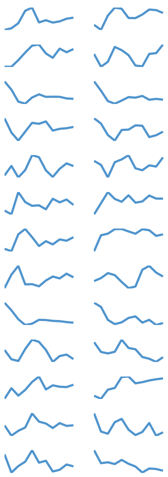

Figure S48: Yeast Cell Cycle, WT1. Top 10% of SW, not in top 10% of DL, LS, JTK. Filtered for noise using replicates (a combined score from a modified version of JTK and SW). Overlap with negative universal growth rate response (GRR) from Slavov and Botstein (2011). Genes that were in the periodic gene lists from Spellman *et al.* (1998) or Orlando *et al.* (2008) are marked.

Yeast Cell Cycle, Top 10% SW, Not Top 10% Others, Noise Filtered, AND GRR Positive

sys_name	symbol	SW_rank	LS_rank	JTK_rank	DL_rank	rep_score	Spellman	Orlando	wt1	wt2
YGR001C	---	259.0	1414.0	681.0	1167.0	7.69e−03			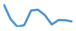	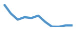
YGL256W	ADH4	350.0	1089.0	1207.0	685.0	1.32e−05			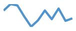	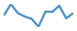
YGL099W	LSG1	542.0	1846.0	681.0	1136.0	4.61e−02		x	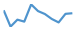	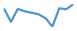

Figure S49: Yeast Cell Cycle, WT1. Top 10% of SW, not in top 10% of DL, LS, JTK. Filtered for noise using replicates (a combined score from a modified version of JTK and SW). Overlap with positive universal growth rate response (GRR) from Slavov and Botstein (2011). Genes that were in the periodic gene lists from Spellman *et al.* (1998) or Orlando *et al.* (2008) are marked.

9 GO Enrichment Analysis

9.1 SW1PerS

Term	Count	%	PValue
GO:0007049 cell cycle	94	15.64	7.33e-06
GO:0022402 cell cycle process	83	13.81	5.54e-06
GO:0033554 cellular response to stress	79	13.14	4.38e-04
GO:0045449 regulation of transcription	78	12.98	3.84e-02
GO:0051276 chromosome organization	68	11.31	8.67e-07
GO:0006259 DNA metabolic process	67	11.15	4.48e-04
GO:0022403 cell cycle phase	65	10.82	1.26e-04
GO:0051252 regulation of RNA metabolic process	59	9.82	2.84e-02
GO:0006355 regulation of transcription, DNA-dependent	57	9.48	4.05e-02
GO:0006974 response to DNA damage stimulus	53	8.82	5.86e-06
GO:0051301 cell division	53	8.82	7.56e-04
GO:0000278 mitotic cell cycle	51	8.49	1.49e-04

Table S7: Gene Ontology (GO) enrichment of biological processes categories. The top 10% of SW for Yeast Cell Cycle WT1. Gene list analyzed using DAVID, set contains 601 DAVID IDs, using GOTERM_BP_FAT. Top by percent, 50 or more in group, p-value < 0.05.

Term	Count	%	PValue
GO:0033554 cellular response to stress	25	16.78	3.61E-03
GO:0006974 response to DNA damage stimulus	15	10.07	9.79E-03
GO:0006281 DNA repair	14	9.40	6.34E-03
GO:0006302 double-strand break repair	7	4.70	4.25E-03
GO:0006298 mismatch repair	6	4.03	4.82E-04
GO:0000726 non-recombinational repair	5	3.36	1.17E-02

Table S8: Gene Ontology (GO) enrichment of biological processes categories. The top 10% of SW, not top 10% of DL, JTK, LS; for Yeast Cell Cycle WT1. Gene list analyzed using DAVID, set contains 149 DAVID IDs, using GOTERM_BP_FAT. Top by percent, 5 or more in group, p-value < 0.05.

9.2 DL

Term	Count	%	PValue
GO:0007049 cell cycle	159	26.37	2.87e-31
GO:0022402 cell cycle process	136	22.55	9.50e-27
GO:0022403 cell cycle phase	116	19.24	2.89e-25
GO:0000278 mitotic cell cycle	101	16.75	2.41e-28
GO:0051301 cell division	100	16.58	2.84e-23
GO:0033554 cellular response to stress	95	15.75	5.37e-07
GO:0000279 M phase	89	14.76	4.39e-18
GO:0006259 DNA metabolic process	83	13.76	1.75e-07
GO:0051276 chromosome organization	81	13.43	2.57e-10
GO:0048285 organelle fission	68	11.28	9.95e-21
GO:0000280 nuclear division	66	10.95	1.94e-20
GO:0007067 mitosis	61	10.12	2.58e-18
GO:0000087 M phase of mitotic cell cycle	61	10.12	4.82e-18

Table S9: Gene Ontology (GO) enrichment of biological processes categories. The top 10% of DL for Yeast Cell Cycle WT1. Gene list analyzed using DAVID, set contains 603 DAVID IDs, using GOTERM_BP_FAT. Top by percent, 60 or more in group, p-value < 0.05.

Term	Count	%	PValue
GO:0007049 cell cycle	61	32.62	9.83E-17
GO:0022402 cell cycle process	51	27.27	2.23E-13
GO:0022403 cell cycle phase	45	24.06	2.97E-13
GO:0051301 cell division	41	21.93	1.74E-13
GO:0000278 mitotic cell cycle	38	20.32	5.37E-13
GO:0000279 M phase	38	20.32	6.61E-12
GO:0033554 cellular response to stress	35	18.72	1.28E-04
GO:0006259 DNA metabolic process	33	17.65	1.23E-05
GO:0000280 nuclear division	27	14.44	8.20E-11
GO:0048285 organelle fission	27	14.44	2.14E-10
GO:0051276 chromosome organization	26	13.90	4.41E-04
GO:0007067 mitosis	24	12.83	4.42E-09
GO:0000087 M phase of mitotic cell cycle	24	12.83	5.53E-09
GO:0009628 response to abiotic stimulus	22	11.76	2.67E-03
GO:0007059 chromosome segregation	21	11.23	1.79E-07
GO:0051726 regulation of cell cycle	20	10.70	2.68E-06
GO:0006414 translational elongation	20	10.70	1.15E-02

Table S10: Gene Ontology (GO) enrichment of biological processes categories. The top 10% of DL, not top 10% of SW, JTK, LS; for Yeast Cell Cycle WT1. Gene list analyzed using DAVID, set contains 187 DAVID IDs, using GOTERM_BP_FAT. Top by percent, 20 or more in group, p-value < 0.05.

9.3 JTK

Term	Count	%	PValue
GO:0007049 cell cycle	90	15.99	4.60e-05
GO:0022402 cell cycle process	82	14.56	6.80e-06
GO:0045449 regulation of transcription	77	13.68	4.17e-02
GO:0006350 transcription	68	12.08	2.80e-02
GO:0033554 cellular response to stress	67	11.90	4.28e-02
GO:0051276 chromosome organization	66	11.72	2.67e-06
GO:0022403 cell cycle phase	66	11.72	4.98e-05
GO:0006259 DNA metabolic process	61	10.83	6.33e-03
GO:0000278 mitotic cell cycle	53	9.41	2.79e-05
GO:0051301 cell division	52	9.24	1.05e-03

Table S11: Gene Ontology (GO) enrichment of biological processes categories. The top 10% of JTK for Yeast Cell Cycle WT1. Gene list analyzed using DAVID, set contains 563 DAVID IDs, using GOTERM_BP_FAT. Top by percent, 50 or more in group, p-value < 0.05.

Term	Count	%	PValue
GO:0044265 cellular macromolecule catabolic process	13	14.77	3.64E-02
GO:0009057 macromolecule catabolic process	13	14.77	4.83E-02
GO:0044257 cellular protein catabolic process	12	13.64	1.07E-02
GO:0030163 protein catabolic process	12	13.64	1.38E-02
GO:0019941 modification-dependent protein catabolic process	9	10.23	1.99E-02
GO:0051603 proteolysis involved in cellular protein catabolic process	9	10.23	2.42E-02
GO:0043632 modification-dependent macromolecule catabolic process	9	10.23	2.93E-02
GO:0032543 mitochondrial translation	5	5.68	4.25E-02

Table S12: Gene Ontology (GO) enrichment of biological processes categories. The top 10% of JTK, not top 10% of SW, DL, LS; for Yeast Cell Cycle WT1. Gene list analyzed using DAVID, set contains 88 DAVID IDs, using GOTERM_BP_FAT. Top by percent, 5 or more in group, p-value < 0.05.

9.4 LS

Term	Count	%	PValue
GO:0007049 cell cycle	92	15.73	3.57e-05
GO:0022402 cell cycle process	84	14.36	4.50e-06
GO:0006350 transcription	69	11.79	3.07e-02
GO:0022403 cell cycle phase	66	11.28	9.63e-05
GO:0051301 cell division	58	9.91	4.39e-05
GO:0000278 mitotic cell cycle	56	9.57	5.47e-06
GO:0051276 chromosome organization	50	8.55	3.38e-02

Table S13: Gene Ontology (GO) enrichment of biological processes categories. The top 10% of LS for Yeast Cell Cycle WT1. Gene list analyzed using DAVID, set contains 585 DAVID IDs, using GOTERM_BP_FAT. Top by percent, 50 or more in group, p-value < 0.05.

Term	Count	%	PValue
GO:0006350 transcription	17	15.74	3.34E-02
GO:0006508 proteolysis	13	12.04	2.96E-02
GO:0051603 proteolysis involved in cellular protein catabolic process	10	9.26	2.91E-02
GO:0010033 response to organic substance	8	7.41	2.43E-02
GO:0015749 monosaccharide transport	5	4.63	8.64E-04
GO:0008645 hexose transport	5	4.63	8.64E-04
GO:0008643 carbohydrate transport	5	4.63	6.09E-03
GO:0030433 ER-associated protein catabolic process	5	4.63	7.19E-03

Table S14: Gene Ontology (GO) enrichment of biological processes categories. The top 10% of LS, not top 10% of SW, DL, JTK; for Yeast Cell Cycle WT1. Gene list analyzed using DAVID, set contains 108 DAVID IDs, using GOTERM_BP_FAT. Top by percent, 5 or more in group, p-value < 0.05.

References

Cohen-Steiner, D., Edelsbrunner, H., Harer, J., and Mileyko, Y. (2010). Lipschitz Functions Have L p -Stable Persistence. *Foundations of Computational Mathematics*, **10**(2), 127–139.

Comaniciu, D. and Meer, P. (2002). Mean shift: A robust approach toward feature space analysis. *Pattern Analysis and Machine Intelligence*, **24**(5), 603–619.

de Lichtenberg, U., Jensen, L. J., Fausbøll, A., Jensen, T. S., Bork, P., and Brunak, S. (2005). Comparison of computational methods for the identification of cell cycle-regulated genes. *Bioinformatics*, **21**(7), 1164–1171.

Edelsbrunner, H. and Harer, J. (2010). *Computational topology: an introduction*. American Mathematical Soc.

Forman, R. (1998). Morse theory for cell complexes. *Advances in Mathematics*, **134**(1), 90–145.

Glynn, E. F., Chen, J., and Mushegian, A. (2006). Detecting periodic patterns in unevenly spaced gene expression time series using Lomb–Scargle periodograms. *Bioinformatics*, **22**(3), 310–316.

Hughes, M., Hogenesch, J. B., and Kornacker, K. (2010). JTK_CYCLE: An Efficient Nonparametric Algorithm for Detecting Rhythmic Components in Genome-Scale Data Sets. *Journal of Biological Rhythms*, **25**(372), 372–380.

Hughes, M. E., DiTacchio, L., Hayes, K. R., Vollmers, C., Pulivarthy, S., Baggs, J. E., Panda, S., and Hogenesch, J. B. (2009). Harmonics of circadian gene transcription in mammals. *PLoS genetics*, **5**(4), e1000442.

Koike, N., Yoo, S.-H., Huang, H.-C., Kumar, V., Lee, C., Kim, T.-K., and Takahashi, J. S. (2012). Transcriptional architecture and chromatin landscape of the core circadian clock in mammals. *Science*, **338**(6105), 349–354.

Mischaikow, K. and Nanda, V. (2013). Morse theory for filtrations and efficient computation of persistent homology. *Discrete & Computational Geometry*, **50**(2), 330–353.

Orlando, D., Lin, C., Bernard, A., Wang, J., Socolar, J., Iversen, E., Hartemink, A., and Haase, S. (2008). Global control of cell-cycle transcription by coupled CDK and network oscillators. *Nature*, **453**(7197), 944–947.

Perea, J. A. and Harer, J. (2014). Sliding Windows and Persistence: An Application of Topological Methods to Signal Analysis. *Foundations of Computational Mathematics (to appear)*. Preprint available at arXiv:1307.6188v2 [math.AT].

Shannon, C. E. (1949). Communication in the presence of noise. *Proceedings of the IRE*, **37**(1), 10–21.

Simon, I., Barnett, J., Hannett, N., Harbison, C. T., Rinaldi, N. J., Volkert, T. L., Wyrick, J. J., Zeitlinger, J., Gifford, D. K., and Jaakkola, T. S. (2001). Serial Regulation of Transcriptional Regulators in the Yeast Cell Cycle. *Cell*, **106**(6), 697–708.

Sing, T., Sander, O., Beerenwinkel, N., and Lengauer, T. (2005). Rocr: visualizing classifier performance in r. *Bioinformatics*, **21**(20), 7881.

Slavov, N. and Botstein, D. (2011). Coupling among growth rate response, metabolic cycle, and cell division cycle in yeast. *Molecular Biology of the Cell*, **22**(12), 1997–2009.

Spellman, P. T. P., Sherlock, G. G., Zhang, M. Q. M., Iyer, V. R. V., Anders, K. K., Eisen, M. B. M., Brown, P. O. P., Botstein, D. D., and Futcher, B. B. (1998). Comprehensive identification of cell cycle-regulated genes of the yeast Saccharomyces cerevisiae by microarray hybridization. *Molecular Biology of the Cell*, **9**(12), 3273–3297.

Tarjan, R. E. (1975). Efficiency of a good but not linear set union algorithm. *Journal of the ACM (JACM)*, **22**(2), 215–225.

Tu, B., Kudlicki, A., Rowicka, M., and McKnight, S. (2005). Logic of the yeast metabolic cycle: temporal compartmentalization of cellular processes. *Science*, **310**(5751), 1152–1158.

Wickham, H. (2009). *ggplot2: elegant graphics for data analysis*. Springer New York.
