# Supplementary material for: SW1PerS: Sliding windows and 1-persistence scoring; discovering periodicity in gene expression time series data
Source: BMC Bioinformatics. 2015 Aug 16;16:257. doi: 10.1186/s12859-015-0645-6 (PMC4537550; doi:10.1186/s12859-015-0645-6)
Supplement: Additional file 3 — Top genes. This zip file contains three pdf files, associated to each one of the 3 biological data sets studied in this paper. Each file shows the full ordered list, sparkLines included, of genes in the top 10 % of rankings according to SW1PerS and that are not present in the top 10 % of the other algorithms. [file 12859_2015_645_MOESM3_ESM.zip › top_genes/hughes2009-liver_res__top10p-sw-oth.pdf]

| Probe | MGI_ID | Symbol | SW_rank | DL_rank | LS_rank | JTK_rank | Max-Min | Norm Plot |
|---|---|---|---|---|---|---|---|---|
| 1418616_at | MGI:99951 | Mafk | 513 | 5089 | 6409.5 | 5844.5 | 213 | 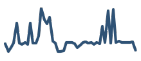 |
| 1445787_at | MGI:1923223 | Ccdc162 | 538 | 4932 | 8340.5 | 14192.5 | 54 | 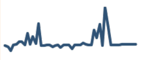 |
| 1422703_at | MGI:106594 | Gyk | 542 | 5224.5 | 6417 | 7066 | 3818.8 | 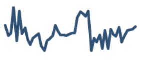 |
| 1421768_a_at | MGI:1347345 | Homer1 | 631 | 5153 | 5368.5 | 7210.5 | 107.4 | 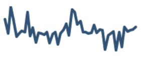 |
| 1453200_at | MGI:103291 | Rai1 | 714 | 4993 | 7609.5 | 11529.5 | 225.4 | 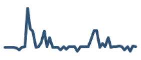 |
| 1451967_x_at | MGI:107532 | Kpnb1 | 826 | 8043 | 7293 | 6394 | 4967.8 | 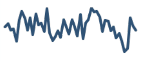 |
| 1424660_s_at | MGI:1921593 | Crtc2 | 830.5 | 11656 | 14651 | 11529.5 | 616.4 | 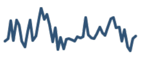 |
| 1460565_at | MGI:2444823 | Slc41a1 | 833 | 8942 | 13194 | 25253.5 | 93.3 | 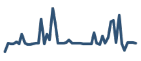 |
| 1455055_at | MGI:3041210 | Ccdc157 | 868.5 | 12068 | 14147.5 | 19316.5 | 739 | 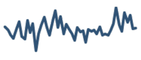 |
| 1448478_at | MGI:1929648 | Med20 | 883 | 10984 | 5716.5 | 7066 | 890.5 | 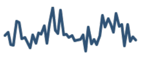 |
| 1452063_at | MGI:1920930 | Zbtb8a | 889 | 9960 | 23012.5 | 37728.5 | 108.3 | 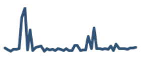 |
| 1428929_s_at | MGI:1914832 | Slc25a26 | 891 | 15162 | 16122.5 | 15975.5 | 112.3 | 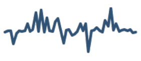 |
| 1420132_s_at | MGI:2652132 | Pttg1ip | 923 | 9677 | 20817.5 | 20671 | 1304.1 | 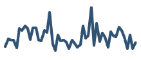 |
| 1419493_a_at | MGI:107749 | Tpd52 | 930 | 5916 | 4545 | 8420 | 1103 | 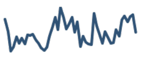 |
| 1452778_x_at | MGI:1855693 | Nap1l1 | 939 | 8586 | 6064 | 6224 | 2983 | 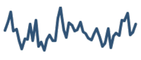 |
| 1451641_at | MGI:1931520 | Dbr1 | 953 | 5783 | 4908.5 | 6785.5 | 979 | 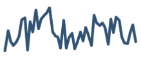 |
| 1430835_at | MGI:1923291 | Ccdc125 | 956 | 7045 | 16822.5 | 19590.5 | 51.6 | 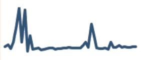 |
| 1423387_at | MGI:1914401 | Psmd9 | 966 | 7521 | 19572 | 18465.5 | 58.8 | 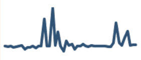 |
| 1456411_at | MGI:2444156 | Rccd1 | 969 | 25196 | 22406 | 13041 | 167 | 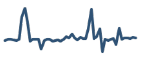 |
| 1418473_at | MGI:1913638 | Cutc | 970 | 5895 | 4565 | 7632 | 1267.7 | 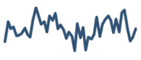 |
| 1434238_at | --- | | 995.5 | 5828 | 5892 | 8323 | 2619.7 | 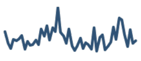 |
| 1430450_at | MGI:1913599 | Atp5sl | 1004 | 7151 | 15999.5 | 23873.5 | 201.1 | 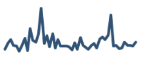 |
| 1423211_at | MGI:1913431 | Nop10 | 1019 | 4774 | 5519 | 6168.5 | 9732.8 | 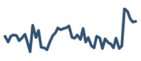 |
| 1435481_at | --- | | 1056 | 9961 | 21307 | 37728.5 | 89.4 | 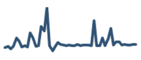 |
| 1424380_at | MGI:1916724 | Vps37b | 1063 | 10677 | 10078 | 5426.5 | 390.6 | 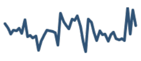 |
| 1437165_a_at | --- | | 1101 | 4984 | 6137.5 | 5475 | 1538.6 | 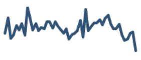 |
| 1446353_at | MGI:1915201 | Tubb6 | 1147 | 9472.5 | 20957.5 | 4947.5 | 25.7 | 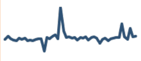 |

| Probe | MGI_ID | Symbol | SW_rank | DL_rank | LS_rank | JTK_rank | Max-Min | Norm Plot |
|---|---|---|---|---|---|---|---|---|
| 1452189_at | MGI:1924555 | Wdr82 | 1166 | 18875 | 15999.5 | 11946.5 | 2692.2 | 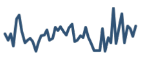 |
| 1447636_x_at | MGI:1924306 | Tmco4 | 1173 | 8125 | 6325.5 | 7377 | 721.8 | 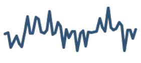 |
| 1430286_s_at | MGI:3705433 | Gm14057 | 1176 | 12034 | 17526 | 23641.5 | 195.9 | 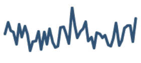 |
| 1438575_a_at | --- | | 1178 | 8051 | 17363 | 37728.5 | 262.6 | 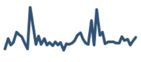 |
| 1438450_at | MGI:2135609 | Lin7a | 1180 | 6018 | 5008.5 | 6111.5 | 1574.3 | 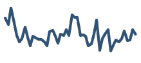 |
| 1434598_at | MGI:106330 | Larp4b | 1209 | 12346 | 9788 | 18465.5 | 13890.4 | 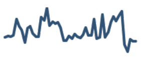 |
| 1417538_at | MGI:1345622 | Slc35a1 | 1235 | 10943 | 13835 | 6785.5 | 2149.3 | 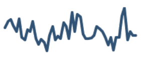 |
| 1428415_at | MGI:1913631 | Rnf113a2 | 1251 | 15787 | 20192.5 | 26273.5 | 1052.1 | 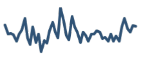 |
| 1452228_at | MGI:1914831 | Tbc1d23 | 1266 | 23911 | 17971.5 | 22916 | 458.9 | 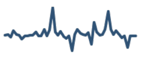 |
| 1421034_a_at | --- | | 1282 | 6667 | 8592 | 5475 | 539.7 | 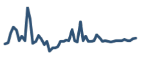 |
| 1415769_at | MGI:1202301 | Itch | 1290 | 8120 | 7403 | 8123.5 | 4371.3 | 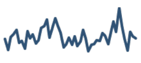 |
| 1451400_at | MGI:2384300 | Gemin8 | 1301.5 | 11306 | 16797 | 37728.5 | 58.6 | 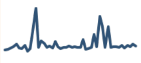 |
| 1429516_at | MGI:1861099 | Ubr2 | 1311 | 19984 | 12489 | 5228 | 126.3 | 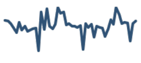 |
| 1439136_at | --- | | 1318 | 11372 | 20817.5 | 22916 | 94.3 | 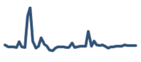 |
| 1446955_at | --- | | 1321 | 37215 | 19417 | 37728.5 | 64 | 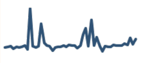 |
| 1439129_at | --- | | 1324.5 | 11989 | 8305 | 14440.5 | 58.4 | 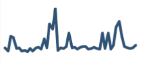 |
| 1452401_at | MGI:1926395 | Wtap | 1326 | 8014 | 10196 | 9771 | 5227.4 | 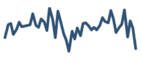 |
| 1440917_at | MGI:1915287 | 00093K20R | 1329 | 15834 | 25295 | 13264 | 116.9 | 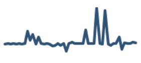 |
| 1420968_at | MGI:1914080 | Nacc1 | 1332 | 7671 | 15362 | 8727 | 203.2 | 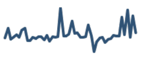 |
| 1429320_at | MGI:1918175 | Cdkn2aip | 1338 | 8018 | 7229.5 | 8420 | 588.4 | 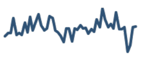 |
| 1417921_at | MGI:1914933 | 10029G23R | 1343.5 | 6151 | 9848 | 8727 | 2025 | 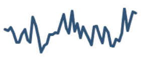 |
| 1443941_at | MGI:2685293 | Gm447 | 1352 | 23633.5 | 30534 | 37728.5 | 77.6 | 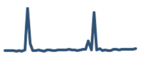 |
| 1436620_at | --- | | 1376 | 27303.5 | 27815 | 37728.5 | 60.3 | 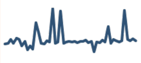 |
| 1429265_a_at | MGI:1891717 | Rnf130 | 1378 | 22840 | 11179.5 | 8323 | 4970.6 | 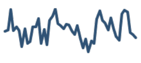 |
| 1454984_at | --- | | 1384 | 5170 | 8649.5 | 5942 | 20642.5 | 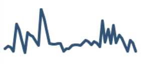 |
| 1424413_at | MGI:1917405 | Ogfrl1 | 1399 | 5720 | 5182.5 | 11529.5 | 404.6 | 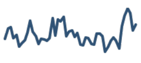 |
| 1424123_at | MGI:2384974 | Mfsd7c | 1407 | 8979 | 18991 | 24799.5 | 53.7 | 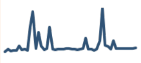 |

| Probe | MGI_ID | Symbol | SW_rank | DL_rank | LS_rank | JTK_rank | Max-Min | Norm Plot |
|---|---|---|---|---|---|---|---|---|
| 1448939_at | MGI:1353655 | Usp25 | 1408 | 26253 | 25705.5 | 37728.5 | 3453.5 | |
| 1459859_x_at | MGI:2135796 | Chrac1 | 1412 | 8135 | 10096.5 | 16234 | 2976.5 | |
| 1425012_at | MGI:109164 | Gng5 | 1414 | 22032 | 23215.5 | 21692.5 | 49.9 | |
| 1429859_a_at | MGI:1349429 | Arl2bp | 1434.5 | 7714 | 25720.5 | 28658 | 460.5 | |
| 1424519_at | MGI:2685015 | Mtg1 | 1448 | 6535 | 7731.5 | 8958.5 | 2062.7 | |
| 1459433_at | MGI:2443856 | 130051F05R | 1451 | 18994 | 19063.5 | 37728.5 | 89.4 | |
| 1451946_a_at | MGI:1918382 | Cabyr | 1453 | 4545 | 31813.5 | 4989 | 90.4 | |
| 1416843_at | MGI:1270843 | Pde6d | 1461.5 | 10910 | 14527 | 8123.5 | 710.2 | |
| 1416846_a_at | MGI:1933157 | Pdzrn3 | 1478 | 6509 | 8241 | 5555.5 | 1289.6 | |
| 1431337_a_at | MGI:1915144 | Fam45a | 1496 | 7138 | 5243.5 | 6915.5 | 2617.4 | |
| 1428777_at | MGI:2150016 | Spred1 | 1498 | 4897 | 23215.5 | 5228 | 128.5 | |
| 1435880_at | MGI:2139777 | Ankrd50 | 1504.5 | 4663 | 22111.5 | 6526 | 369.4 | |
| 1416861_at | MGI:1329014 | Stam | 1504.5 | 4813 | 5630 | 6050.5 | 755.8 | |
| 1437338_x_at | MGI:1921445 | Elp3 | 1511.5 | 26669.5 | 26398.5 | 37728.5 | 19.2 | |
| 1436906_at | MGI:1915968 | Rnf166 | 1530 | 23294 | 12038 | 9771 | 800.5 | |
| 1423998_at | MGI:1917489 | Gtf3c5 | 1535.5 | 22422.5 | 24685 | 24341 | 229.2 | |
| 1436760_a_at | MGI:98166 | Rps8 | 1548.5 | 7342 | 5310.5 | 6050.5 | 27529.7 | |
| 1423064_at | MGI:1261827 | Dnmt3a | 1557 | 31213.5 | 32815 | 37728.5 | 62 | |
| 1436867_at | --- | | 1562 | 21837 | 33162 | 37728.5 | 129.5 | |
| 1441098_at | MGI:2685159 | Pnldc1 | 1567 | 4728 | 14527 | 10419.5 | 36.6 | |
| 1416974_at | MGI:1929100 | Stam2 | 1574 | 10632 | 6240.5 | 5942 | 376.3 | |
| 1448689_at | MGI:1914172 | Rras2 | 1605 | 14030 | 13086 | 10419.5 | 1080.2 | |
| 1449324_at | MGI:1354385 | Ero1l | 1610 | 6400 | 6334 | 7925.5 | 2225 | |
| 1424373_at | MGI:1918953 | Armcx3 | 1614 | 4662 | 6623 | 8123.5 | 2243.4 | |
| 1418034_at | MGI:1916777 | Mrps9 | 1634 | 5296 | 7086 | 5228 | 762.3 | |
| 1453706_at | --- | | 1636.5 | 20653 | 20025.5 | 28851.5 | 55 | |
| 1453547_at | MGI:1917059 | 310046K07R | 1643 | 6606.5 | 12427 | 26491 | 383.1 | |

| Probe | MGI_ID | Symbol | SW_rank | DL_rank | LS_rank | JTK_rank | Max-Min | Norm Plot |
|---|---|---|---|---|---|---|---|---|
| 1419723_at | MGI:1097692 | Opn1mw | 1645 | 6687 | 26241.5 | 4777.5 | 27.8 | 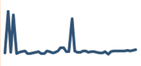 |
| 1423660_at | MGI:1098748 | Ctdsp2 | 1659 | 8870 | 6689.5 | 5645.5 | 1074.4 | 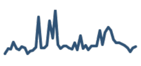 |
| 1438684_at | --- | | 1669 | 21332 | 25672 | 16234 | 67.2 | 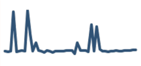 |
| 1434113_a_at | MGI:2684957 | Rexo4 | 1695 | 14202 | 18038.5 | 29027 | 3191.6 | 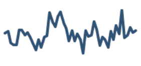 |
| 1416494_at | MGI:3612445 | BC002163 | 1703 | 6840.5 | 6396 | 4650 | 15689.2 | 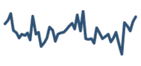 |
| 1421365_at | MGI:95586 | Fst | 1708 | 14506 | 18177.5 | 37728.5 | 374.9 | 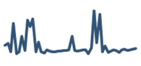 |
| 1417061_at | MGI:1315204 | Slc40a1 | 1709 | 11983 | 9375 | 8958.5 | 29455.7 | 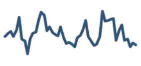 |
| 1433567_at | MGI:2448526 | Gmps | 1713 | 8150 | 5868 | 7925.5 | 9228.6 | 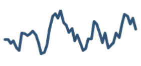 |
| 1423453_at | MGI:2146285 | Nol12 | 1718 | 7225 | 7110 | 4563.5 | 2710.4 | 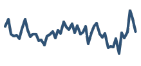 |
| 1448843_at | MGI:105082 | Ssr1 | 1722 | 6610 | 15015 | 15443.5 | 797.1 | 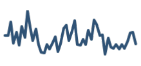 |
| 1460053_at | MGI:2442796 | Smyd4 | 1731 | 21001 | 23343.5 | 17046 | 58.6 | 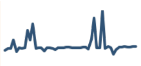 |
| 1448283_a_at | MGI:1858313 | Uba2 | 1736 | 6233 | 5537.5 | 6168.5 | 2646.5 | 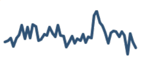 |
| 1451656_at | MGI:2136957 | Clmn | 1743 | 4999.5 | 5838 | 4733.5 | 52.3 | 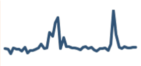 |
| 1453746_at | MGI:109606 | Fnbp1 | 1745 | 6628 | 16946.5 | 6455.5 | 49.4 | 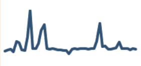 |
| 1437241_at | --- | | 1748 | 9194 | 19831 | 37728.5 | 282.5 | 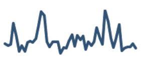 |
| 1430549_at | MGI:1913128 | Bet1l | 1749 | 4949 | 9051.5 | 27698 | 334.4 | 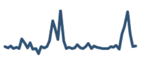 |
| 1423838_s_at | MGI:1919205 | 400003C14R | 1753 | 10957 | 6453.5 | 8958.5 | 3484.4 | 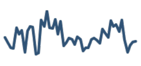 |
| 1428449_at | MGI:1919002 | Gtf3c2 | 1760.5 | 5066 | 13430.5 | 7463.5 | 1199.4 | 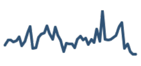 |
| 1427650_a_at | MGI:99852 | Runx1 | 1760.5 | 5529.5 | 14943 | 5389 | 15.5 | 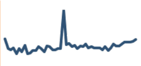 |
| 1425134_a_at | MGI:1919334 | Pigx | 1767 | 5431 | 5708.5 | 5745 | 8060 | 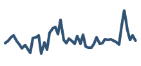 |
| 1433626_at | MGI:2143267 | Plscr4 | 1768 | 8822 | 15668 | 13958.5 | 92.4 | 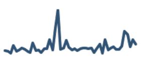 |
| 1422753_a_at | MGI:1914255 | Polr3k | 1773 | 5918 | 16771.5 | 10419.5 | 1491.3 | 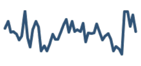 |
| 1428956_at | MGI:1913593 | Tmem177 | 1774 | 27218.5 | 31902 | 37728.5 | 193 | 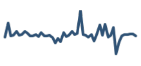 |
| 1443952_at | --- | | 1777.5 | 13129 | 4875.5 | 7210.5 | 547.3 | 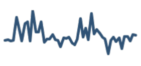 |
| 1426697_a_at | MGI:96829 | Lrpap1 | 1787 | 6726 | 6985.5 | 9456 | 776.6 | 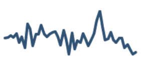 |
| 1448037_at | --- | | 1788.5 | 18093.5 | 16896 | 26721 | 877.4 | 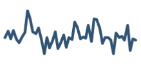 |
| 1429116_at | MGI:1924105 | Slc17a5 | 1802 | 7259 | 5712 | 6168.5 | 1557.7 | 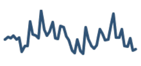 |

| Probe | MGI_ID | Symbol | SW_rank | DL_rank | LS_rank | JTK_rank | Max-Min | Norm Plot |
|---|---|---|---|---|---|---|---|---|
| 1458969_at | MGI:1352745 | Fscn1 | 1810 | 8950 | 27451 | 37728.5 | 216.8 | |
| 1436883_at | MGI:2444506 | Mbtps2 | 1811.5 | 6004 | 9002.5 | 7834 | 1115.4 | |
| 1421031_a_at | MGI:1916823 | 310016C08R | 1811.5 | 23908.5 | 20703.5 | 6338 | 115.5 | |
| 1437105_at | --- | | 1813 | 8552 | 12489 | 20130 | 2183.6 | |
| 1425742_a_at | MGI:109127 | Tsc22d1 | 1820 | 5026 | 10947.5 | 26273.5 | 44866.8 | |
| 1429034_at | MGI:1919889 | Eme2 | 1821 | 28027 | 19039.5 | 5745 | 259.4 | |
| 1435171_at | MGI:1914374 | 310416G20R | 1822 | 14449 | 18897 | 24565 | 80.5 | |
| 1424810_at | MGI:1923062 | Tasp1 | 1850 | 11674 | 22022.5 | 14192.5 | 1011 | |
| 1455597_at | MGI:1346873 | Map3k2 | 1857 | 12846 | 12319.5 | 11130.5 | 1244.8 | |
| 1427359_at | MGI:2443388 | Jhdm1d | 1860 | 9245 | 19270 | 16234 | 420.5 | |
| 1450318_a_at | MGI:105107 | P2ry2 | 1863.5 | 11673 | 17053 | 6590 | 207.8 | |
| 1444015_at | --- | | 1866.5 | 6546 | 8666 | 6394 | 691.4 | |
| 1415737_at | MGI:1914688 | Rfk | 1869 | 11731 | 12769.5 | 7734 | 2887.7 | |
| 1455268_at | MGI:1922658 | Dph3 | 1869 | 22528 | 23200 | 37728.5 | 261.5 | |
| 1453735_at | MGI:1917841 | 730455P16R | 1873.5 | 8408 | 10488.5 | 7544 | 917.6 | |
| 1427013_at | MGI:2447188 | Car9 | 1877 | 21605 | 22891.5 | 19869 | 158.8 | |
| 1417129_a_at | MGI:108564 | Meis2 | 1891.5 | 12917 | 20974 | 37728.5 | 202.8 | |
| 1430019_a_at | MGI:104820 | Hnrnpa1 | 1895 | 7135 | 12489 | 8727 | 5500.5 | |
| 1417261_at | MGI:2143977 | Mbtd1 | 1896 | 5640 | 14281 | 6718 | 593.9 | |
| 1441615_at | --- | | 1909.5 | 24418 | 23677.5 | 37728.5 | 151.3 | |
| 1452371_at | MGI:1916457 | Srsf11 | 1917 | 5615 | 5299.5 | 5844.5 | 6696.5 | |
| 1451558_at | MGI:1354695 | Fbxw7 | 1921 | 8107 | 15057.5 | 37728.5 | 764.8 | |
| 1418825_at | MGI:107567 | Irgm1 | 1931 | 5618 | 6388 | 5745 | 5526.9 | |
| 1431742_at | MGI:1917107 | 310053B23R | 1934.5 | 10356 | 29867.5 | 15443.5 | 117.3 | |
| 1434506_at | MGI:1924294 | Arid2 | 1941.5 | 6067 | 9357.5 | 5895.5 | 276.8 | |
| 1449406_at | MGI:1859320 | Cyhr1 | 1944 | 10620 | 13194 | 8224.5 | 1322.1 | |
| 1420089_at | MGI:104741 | Nfkbia | 1947 | 10317 | 18319 | 6050.5 | 491.5 | |

| Probe | MGI_ID | Symbol | SW_rank | DL_rank | LS_rank | JTK_rank | Max-Min | Norm Plot |
|---|---|---|---|---|---|---|---|---|
| 1451390_s_at | MGI:1916068 | Zfand2b | 1950 | 6146 | 4751 | 6282.5 | 660.2 | |
| 1418908_at | MGI:97475 | Pam | 1952 | 20427 | 21372.5 | 25466.5 | 52.3 | |
| 1421893_a_at | MGI:102724 | Tpp2 | 1960 | 8105 | 5008.5 | 4814.5 | 1227.1 | |
| 1431921_a_at | MGI:1098658 | Stag1 | 1961.5 | 6089 | 4935 | 5895.5 | 1130.9 | |
| 1415939_at | MGI:1328364 | Fmod | 1965.5 | 8500 | 18764.5 | 25466.5 | 82.3 | |
| 1440228_at | MGI:2683212 | Ranbp6 | 1968 | 7969 | 22780 | 37728.5 | 1286 | |
| 1434718_at | --- | | 1972.5 | 8905 | 15334 | 23400.5 | 187.2 | |
| 1418120_at | MGI:1913129 | Rbm8a | 1991 | 9288 | 14565.5 | 6224 | 178.9 | |
| 1433174_a_at | MGI:1918634 | 430440L12R | 1994 | 5131 | 21424 | 4563.5 | 34.3 | |
| 1419258_at | MGI:1196624 | Tcea1 | 1995 | 7150 | 7412 | 7066 | 13588 | |
| 1444054_at | --- | | 2000 | 6596 | 21508.5 | 19869 | 75.8 | |
| 1427112_at | MGI:1916987 | Ttl | 2003 | 5863 | 8608 | 5475 | 341.4 | |
| 1415911_at | MGI:1098233 | Impact | 2006 | 12840 | 16624 | 19590.5 | 954.4 | |
| 1417604_at | MGI:1098535 | Camk1 | 2007 | 14942 | 22300 | 27146 | 665.2 | |
| 1451978_at | MGI:106096 | Loxl1 | 2009 | 7597.5 | 19831 | 16775 | 80.3 | |
| 1445484_at | MGI:88578 | Cycs | 2015 | 5075 | 17836.5 | 9609.5 | 148.5 | |
| 1418607_at | MGI:1914485 | Zkscan14 | 2017 | 7188 | 9660.5 | 7544 | 548.1 | |
| 1434427_a_at | MGI:2442484 | Rnf157 | 2018.5 | 7240 | 12872 | 7066 | 100.4 | |
| 1435325_at | MGI:1916977 | Usp46 | 2021 | 7597.5 | 11101.5 | 7377 | 1436.6 | |
| 1425534_at | MGI:1352508 | Stau2 | 2023 | 7074 | 14798.5 | 17603 | 53.4 | |
| 1460699_at | MGI:1888676 | Rps27 | 2034 | 8619.5 | 13238.5 | 5793.5 | 57.4 | |
| 1424741_s_at | MGI:99946 | Creb3 | 2047 | 9910 | 9597 | 5309 | 3013.1 | |
| 1458057_at | --- | | 2049 | 5934 | 15901.5 | 21938.5 | 461 | |
| 1454794_at | MGI:1858896 | Spast | 2050.5 | 13946 | 13026 | 8123.5 | 1118.9 | |
| 1425488_at | MGI:2385598 | Slu7 | 2059 | 7250 | 5907.5 | 8727 | 1356.4 | |
| 1428840_s_at | MGI:1916230 | Wdr53 | 2061 | 6540 | 14392 | 26060 | 301 | |
| 1416910_at | MGI:1913398 | Dnajc15 | 2064 | 8073 | 10762.5 | 18465.5 | 11246.6 | |

| Probe | MGI_ID | Symbol | SW_rank | DL_rank | LS_rank | JTK_rank | Max-Min | Norm Plot |
|---|---|---|---|---|---|---|---|---|
| 1451622_at | MGI:1915671 | Lmbrd1 | 2069 | 24336.5 | 9616.5 | 6650.5 | 7198.8 | |
| 1433789_at | --- | | 2074.5 | 6197 | 17642.5 | 12382 | 409.7 | |
| 1417483_at | MGI:1931595 | Nfkbiz | 2078 | 12340 | 20416.5 | 14192.5 | 2325.9 | |
| 1427418_a_at | MGI:106918 | Hif1a | 2079 | 7292 | 5591 | 6168.5 | 4194.7 | |
| 1428099_a_at | MGI:98283 | Srsf1 | 2085 | 14474 | 5614 | 5645.5 | 16127.7 | |
| 1448964_at | MGI:104528 | S100g | 2089 | 5367 | 23012.5 | 10257.5 | 37.5 | |
| 1454949_at | MGI:2182061 | Usp7 | 2092 | 30092 | 29703.5 | 37728.5 | 33.9 | |
| 1420909_at | MGI:103178 | Vegfa | 2094 | 5250 | 4661.5 | 5475 | 4587.1 | |
| 1455003_at | --- | | 2104 | 5744 | 7278.5 | 5028.5 | 917.1 | |
| 1436133_at | MGI:1914683 | Ccdc127 | 2107 | 19581 | 17879 | 5272 | 84.2 | |
| 1459414_at | --- | | 2120 | 33345.5 | 31724.5 | 37728.5 | 50.6 | |
| 1455237_at | MGI:1919594 | Usp36 | 2121 | 23187 | 22891.5 | 37728.5 | 448.5 | |
| 1425200_at | MGI:2385186 | Clcc1 | 2122 | 30407.5 | 24036.5 | 14192.5 | 66.3 | |
| 1430293_a_at | MGI:1915415 | Fdx1l | 2124 | 9978 | 11568.5 | 14688 | 1835.4 | |
| 1421872_at | MGI:105065 | Rab24 | 2125 | 7092 | 4886.5 | 7210.5 | 2728.6 | |
| 1429081_at | MGI:1917547 | Gcc2 | 2131 | 7735 | 8763 | 8516.5 | 2557.3 | |
| 1442248_at | --- | | 2134 | 14459 | 22380 | 37728.5 | 107.6 | |
| 1459784_x_at | MGI:1914137 | Lonp2 | 2140 | 29834 | 11687.5 | 16234 | 409.7 | |
| 1416536_at | MGI:1915364 | Mum1 | 2146 | 8555 | 13026 | 17907.5 | 307 | |
| 1421036_at | MGI:109232 | Npas2 | 2148.5 | 9641 | 21356 | 6111.5 | 49.7 | |
| 1439336_at | --- | | 2148.5 | 25969.5 | 27081 | 37728.5 | 50.4 | |
| 1418285_at | MGI:102708 | Efnb1 | 2153 | 7326 | 5965.5 | 6282.5 | 346.7 | |
| 1422650_a_at | MGI:1914128 | Riok3 | 2155 | 5321.5 | 4875.5 | 4650 | 3428.4 | |
| 1423689_a_at | MGI:1915089 | Gpsm1 | 2166 | 12016 | 19700 | 16234 | 73.1 | |
| 1422173_at | MGI:102851 | Pdx1 | 2166 | 14947 | 21740 | 17046 | 114 | |
| 1448593_at | MGI:1197008 | Wisp1 | 2171 | 22817.5 | 22726 | 37728.5 | 29.6 | |
| 1421023_at | MGI:1203729 | Pik3c2a | 2173 | 5907 | 5478.5 | 8727 | 8730.1 | |

| Probe | MGI_ID | Symbol | SW_rank | DL_rank | LS_rank | JTK_rank | Max-Min | Norm Plot |
|---|---|---|---|---|---|---|---|---|
| 1452954_at | MGI:1915862 | Ube2c | 2174 | 11033 | 32982.5 | 37728.5 | 191.8 | 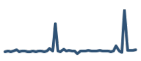 |
| 1436538_at | MGI:3603344 | Ankrd37 | 2175.5 | 8428 | 25185.5 | 7734 | 150.7 | 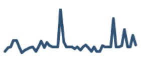 |
| 1455541_a_at | --- | | 2185 | 29505 | 20416.5 | 20389.5 | 47.8 | 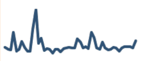 |
| 1419147_at | MGI:1929645 | Rec8 | 2189 | 7000 | 18529 | 11130.5 | 75.8 | 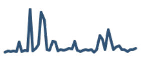 |
| 1429295_s_at | MGI:1916966 | Trip13 | 2193 | 8961 | 19816 | 23158.5 | 117.8 | 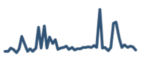 |
| 1420936_s_at | MGI:1861601 | Cpsf2 | 2224 | 7061 | 10562 | 6168.5 | 514.9 | 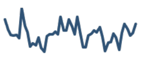 |
| 1444320_at | MGI:1919358 | Ddhd2 | 2229 | 5838 | 11302 | 11733 | 111.9 | 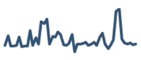 |
| 1428279_a_at | MGI:3584458 | Atxn7l1 | 2231 | 6440.5 | 8430 | 6785.5 | 481.3 | 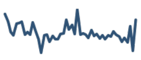 |
| 1448358_s_at | MGI:1915261 | Snrpg | 2237 | 18781 | 17177 | 23641.5 | 5070.8 | 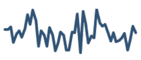 |
| 1447986_at | MGI:2143951 | D17892 | 2243 | 9204 | 10033.5 | 8123.5 | 300.5 | 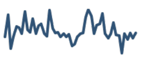 |
| 1438032_at | MGI:2443390 | Lrch1 | 2245 | 18223 | 19658.5 | 27146 | 136.4 | 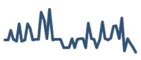 |
| 1437788_at | MGI:1932575 | Sp6 | 2247 | 7368.5 | 12319.5 | 14440.5 | 29.5 | 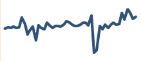 |
| 1439515_at | MGI:1920145 | Setd5 | 2256 | 5204 | 8064 | 6455.5 | 417 | 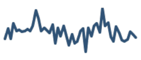 |
| 1419292_at | MGI:1925808 | Htra3 | 2258.5 | 6092 | 10488.5 | 5993.5 | 84.4 | 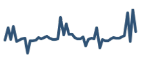 |
| 1428679_s_at | MGI:1915609 | 510010K14R | 2268.5 | 7555 | 6849 | 11946.5 | 990 | 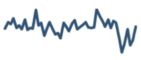 |
| 1424475_at | --- | | 2282 | 10970 | 18014.5 | 37728.5 | 192.3 | 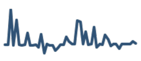 |
| 1426362_at | MGI:1917902 | Tmem144 | 2286 | 26572.5 | 28854 | 37728.5 | 424.2 | 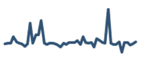 |
| 1424180_a_at | MGI:1344385 | Med24 | 2288.5 | 30511 | 23799.5 | 8224.5 | 396.2 | 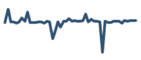 |
| 1429065_at | MGI:1914704 | Ikbip | 2293 | 7580 | 11835 | 23158.5 | 98.2 | 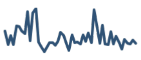 |
| 1448536_at | MGI:1914928 | Lsm3 | 2296 | 5729 | 10698.5 | 29027 | 2947.4 | 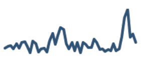 |
| 1456588_x_at | --- | | 2297 | 12924.5 | 14101.5 | 11946.5 | 58582.1 | 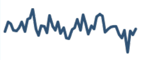 |
| 1420927_at | MGI:108470 | St6gal1 | 2298 | 8841 | 8681.5 | 17907.5 | 1823.9 | 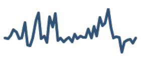 |
| 1452255_at | MGI:2444639 | Fbxo38 | 2299 | 22368.5 | 21828 | 23641.5 | 541.8 | 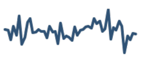 |
| 1435643_x_at | MGI:98888 | Ubb | 2304 | 8178 | 4764.5 | 4898 | 79202.3 | 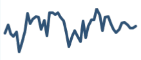 |
| 1440833_at | MGI:1916812 | Cdk13 | 2306 | 5711 | 12372.5 | 5389 | 271.1 | 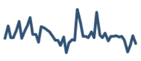 |
| 1452976_a_at | MGI:1890662 | Slc9a3r2 | 2307 | 26239 | 29703.5 | 28453 | 17.6 | 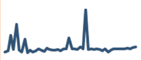 |
| 1447449_at | --- | | 2308 | 24347 | 23036.5 | 9929 | 227.5 | 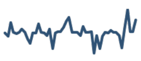 |

| Probe | MGI_ID | Symbol | SW_rank | DL_rank | LS_rank | JTK_rank | Max-Min | Norm Plot |
|---|---|---|---|---|---|---|---|---|
| 1446209_at | --- | | 2311 | 32548.5 | 40494 | 37728.5 | 84.8 | |
| 1425479_at | MGI:108048 | Smyd5 | 2318 | 32246.5 | 30880 | 37728.5 | 17.2 | |
| 1459661_at | MGI:2145433 | AW492955 | 2325 | 31764.5 | 29341.5 | 13264 | 127.2 | |
| 1428131_a_at | MGI:1889510 | Cdc42se1 | 2328 | 17126 | 20550.5 | 14688 | 670.8 | |
| 1431421_x_at | MGI:1913744 | Prelid1 | 2336 | 5529.5 | 4596 | 5745 | 91.6 | |
| 1426707_at | MGI:2183752 | Tubgcp3 | 2338 | 17391 | 23928 | 25253.5 | 584.5 | |
| 1451120_at | MGI:108403 | Polr1d | 2341 | 6430 | 7609.5 | 11130.5 | 7044.1 | |
| 1416411_at | MGI:95861 | Gstm2 | 2354 | 6166 | 8287.5 | 5895.5 | 10905.5 | |
| 1423026_at | MGI:2150020 | Rad51c | 2355 | 5297 | 18386 | 5745 | 52.6 | |
| 1418387_at | MGI:1922589 | Mphosph8 | 2359 | 7721 | 12721.5 | 28658 | 958.2 | |
| 1450958_at | MGI:104678 | Tm4sf1 | 2360 | 8006 | 12769.5 | 12382 | 3590.2 | |
| 1451499_at | MGI:2443963 | Cadps2 | 2363 | 21014 | 12550.5 | 13728 | 1918.8 | |
| 1437120_at | MGI:2443882 | Snx30 | 2364.5 | 24880.5 | 27775.5 | 37728.5 | 138.4 | |
| 1415744_at | MGI:95908 | H2-Ke2 | 2367.5 | 18773 | 16848 | 19033.5 | 4316.1 | |
| 1448948_at | MGI:107417 | Slc50a1 | 2379 | 17400 | 24241.5 | 27146 | 607.1 | |
| 1452428_a_at | MGI:88127 | B2m | 2380 | 15480 | 16822.5 | 13494 | 71053.1 | |
| 1451207_at | MGI:2384909 | Cbara1 | 2382.5 | 4960 | 4805 | 8516.5 | 1317 | |
| 1416313_at | MGI:1929671 | Mllt11 | 2384 | 23908.5 | 31724.5 | 10579.5 | 73.5 | |
| 1447522_s_at | MGI:1921743 | Tnks2 | 2391 | 6183 | 6072 | 7544 | 1990.3 | |
| 1419003_at | MGI:1346013 | Bves | 2395 | 39400 | 40494 | 37728.5 | 26.3 | |
| 1436887_x_at | MGI:2141989 | Grwd1 | 2397 | 16789 | 21760.5 | 6590 | 108.5 | |
| 1452361_at | MGI:1925927 | Rnf20 | 2405 | 23250 | 25767.5 | 37728.5 | 39.9 | |
| 1428050_a_at | MGI:1915462 | Tmbim4 | 2413.5 | 5610 | 5223 | 5745 | 6865.9 | |
| 1417571_at | MGI:97073 | Mpg | 2413.5 | 28664.5 | 25021.5 | 37728.5 | 779.7 | |
| 1440338_at | MGI:2443226 | Pde12 | 2416 | 11346 | 11493.5 | 6050.5 | 56.9 | |
| 1448430_a_at | MGI:106095 | Naca | 2420 | 10007 | 8491.5 | 6282.5 | 46904.3 | |
| 1425405_a_at | MGI:1889575 | Adar | 2429.5 | 6470 | 9261 | 8123.5 | 154.9 | |

| Probe | MGI_ID | Symbol | SW_rank | DL_rank | LS_rank | JTK_rank | Max-Min | Norm Plot |
|---|---|---|---|---|---|---|---|---|
| 1456830_at | MGI:1914099 | Ppp1r2 | 2432 | 26891 | 27061.5 | 28658 | 99.6 | 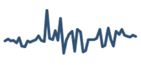 |
| 1442269_at | --- | | 2442.5 | 34965.5 | 23971 | 19590.5 | 26.8 | 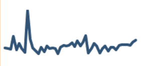 |
| 1429961_at | MGI:1924177 | 00021C14R | 2445 | 6526 | 15563.5 | 13494 | 1004.6 | 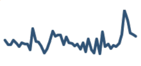 |
| 1439794_at | --- | | 2447 | 5387 | 7780.5 | 7925.5 | 514.3 | 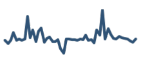 |
| 1429560_at | MGI:4834573 | Zfp955b | 2448 | 14468 | 9736.5 | 8323 | 1193.9 | 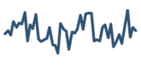 |
| 1437503_a_at | MGI:1915044 | Shisa5 | 2449 | 26669.5 | 12669 | 10090 | 6249.4 | 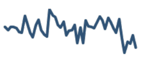 |
| 1427807_at | MGI:3642418 | Gm10083 | 2451 | 23621.5 | 32672 | 15443.5 | 13.5 | 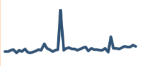 |
| 1460333_at | MGI:1915247 | Ddx59 | 2457 | 7632 | 9553.5 | 6050.5 | 642 | 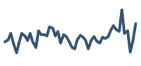 |
| 1434119_at | MGI:106544 | D2Wsu81e | 2459 | 11668 | 14978.5 | 22186.5 | 439.7 | 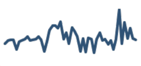 |
| 1425147_at | MGI:1925902 | 10075B13R | 2464 | 12611 | 14978.5 | 14440.5 | 59.4 | 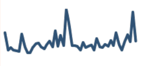 |
| 1419874_x_at | MGI:103222 | Zbtb16 | 2466 | 5802 | 22207.5 | 7734 | 752.9 | 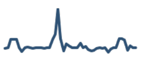 |
| 1433521_at | --- | | 2469.5 | 7371 | 7186 | 7834 | 2589.8 | 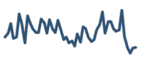 |
| 1454235_a_at | MGI:1922816 | Ing5 | 2471 | 19016 | 22986 | 37728.5 | 28.8 | 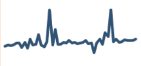 |
| 1428076_s_at | MGI:1915444 | Ndufb4 | 2475.5 | 5724 | 5525.5 | 5068.5 | 54858.8 | 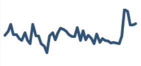 |
| 1457594_at | --- | | 2478 | 43596 | 31632 | 19869 | 19.3 | 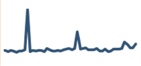 |
| 1436278_at | --- | | 2480 | 8413 | 19848 | 5228 | 128 | 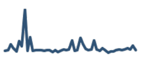 |
| 1444436_at | MGI:2441757 | 630030I15R | 2485.5 | 13964 | 17103 | 13041 | 278.4 | 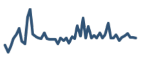 |
| 1425929_a_at | MGI:1929668 | Rnf14 | 2491 | 26856 | 6109 | 6168.5 | 2767.1 | 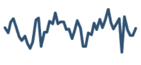 |
| 1431771_a_at | MGI:1929475 | Irak1bp1 | 2495 | 7303 | 19086.5 | 8619 | 94.8 | 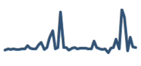 |
| 1428923_at | MGI:1923737 | Ppp1r3g | 2498.5 | 6513 | 26337 | 12382 | 121.5 | 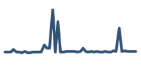 |
| 1418657_at | MGI:1917270 | Ino80b | 2508 | 29090.5 | 25628 | 37728.5 | 447.7 | 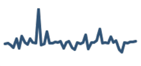 |
| 1454758_a_at | --- | | 2513 | 7312 | 17721.5 | 37728.5 | 43083.8 | 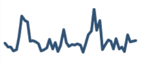 |
| 1452275_at | MGI:1096572 | Zfand3 | 2515.5 | 24332.5 | 33826 | 29027 | 116.2 | 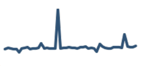 |
| 1437317_at | MGI:1349462 | Uba7 | 2520 | 23948 | 25360.5 | 37728.5 | 54.4 | 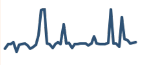 |
| 1444018_at | --- | | 2522 | 6001 | 6431 | 6915.5 | 458 | 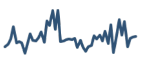 |
| 1440111_at | --- | | 2523 | 44615 | 40494 | 37728.5 | 74.4 | 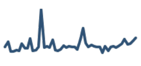 |
| 1437213_at | MGI:1915469 | Nudt21 | 2524.5 | 34711 | 14527 | 13264 | 1447.9 | 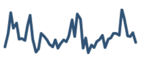 |

| Probe | MGI_ID | Symbol | SW_rank | DL_rank | LS_rank | JTK_rank | Max-Min | Norm Plot |
|---|---|---|---|---|---|---|---|---|
| 1426713_s_at | MGI:97838 | Eprs | 2526 | 7301 | 5434 | 8727 | 7931.5 | |
| 1435143_at | MGI:101762 | Elk3 | 2546 | 8548 | 11243.5 | 11529.5 | 302.3 | |
| 1429763_at | MGI:1925828 | Cnih4 | 2557 | 6098 | 6402 | 6526 | 2759.8 | |
| 1451524_at | MGI:1353435 | Fbxw2 | 2559 | 19858 | 27186.5 | 29548.5 | 268.8 | |
| 1454866_s_at | MGI:2146607 | Clic6 | 2566 | 6806 | 25566 | 4947.5 | 34.1 | |
| 1432805_at | MGI:1924451 | 30431A06R | 2569 | 7445.5 | 20482.5 | 12163.5 | 16.1 | |
| 1451379_at | MGI:105072 | Rab22a | 2572 | 15792 | 7984 | 7066 | 1247.3 | |
| 1455631_at | --- | | 2577 | 16730 | 22455.5 | 37728.5 | 147.7 | |
| 1449413_at | MGI:2135951 | Mpv17l | 2579 | 6237 | 4764.5 | 8022 | 1581.1 | |
| 1417816_s_at | MGI:1349457 | Serinc3 | 2583.5 | 5162 | 6876.5 | 6915.5 | 1872.6 | |
| 1434901_at | MGI:2685949 | Zbtb2 | 2583.5 | 24022.5 | 27232 | 37728.5 | 847.9 | |
| 1416350_at | MGI:2153049 | Klf16 | 2588 | 21681 | 28568 | 4650 | 125.1 | |
| 1424315_at | MGI:1915251 | 110004E09R | 2591 | 12305 | 14565.5 | 23641.5 | 854.8 | |
| 1448408_at | MGI:2177763 | Hps1 | 2596 | 8588 | 13430.5 | 11332.5 | 35.8 | |
| 1431726_a_at | MGI:1918698 | Tmem80 | 2596 | 10935 | 18780 | 17046 | 710.6 | |
| 1458082_at | --- | | 2596 | 14514 | 27815 | 7925.5 | 46.7 | |
| 1449013_at | MGI:1195261 | Eef2k | 2598 | 30565.5 | 31321 | 18744 | 28.5 | |
| 1453465_x_at | MGI:3705433 | Gm14057 | 2601 | 20861 | 22762.5 | 17603 | 424.9 | |
| 1437329_at | MGI:1918007 | Ptplb | 2603 | 10606 | 14056.5 | 12382 | 602.2 | |
| 1450852_s_at | MGI:101802 | F2r | 2604 | 6145 | 6870.5 | 7377 | 10072 | |
| 1455850_at | MGI:1919135 | 10003H01R | 2608.5 | 28863.5 | 25507.5 | 37728.5 | 29.9 | |
| 1449021_at | MGI:1914926 | Rpp21 | 2614 | 8953 | 13378.5 | 6850.5 | 2341.6 | |
| 1428750_at | MGI:1929744 | Cdc42ep2 | 2616 | 4704.5 | 5373.5 | 12163.5 | 147.6 | |
| 1455255_at | MGI:1918877 | 33420G11R | 2617 | 26713 | 25753.5 | 37728.5 | 866.2 | |
| 1426932_at | MGI:106253 | 19Bwg1357 | 2619 | 6492 | 9347.5 | 11946.5 | 312.1 | |
| 1452268_at | MGI:1920076 | Fam76b | 2626 | 13203 | 22873.5 | 12382 | 1565 | |
| 1431184_a_at | MGI:1922264 | 30503B20R | 2627 | 8136 | 13328.5 | 10931 | 225.3 | |

| Probe | MGI_ID | Symbol | SW_rank | DL_rank | LS_rank | JTK_rank | Max-Min | Norm Plot |
|---|---|---|---|---|---|---|---|---|
| 1430418_at | MGI:1913396 | Tmem57 | 2629.5 | 6718 | 16848 | 25466.5 | 503.9 | |
| 1445510_at | MGI:2146293 | C79601 | 2631 | 17632 | 21339 | 10257.5 | 21.2 | |
| 1455269_a_at | MGI:1345961 | Coro1a | 2636 | 24864.5 | 15964.5 | 17315 | 263.9 | |
| 1418863_at | MGI:95664 | Gata4 | 2637 | 5100 | 5661.5 | 4563.5 | 508.1 | |
| 1450432_s_at | MGI:1918961 | Mus81 | 2639 | 23514 | 24699 | 19590.5 | 59.5 | |
| 1448953_at | MGI:1328362 | Blm | 2641 | 21526 | 22010.5 | 37728.5 | 27.6 | |
| 1433631_at | MGI:95309 | Eif5 | 2648 | 9606 | 7385.5 | 7066 | 12185 | |
| 1456433_at | MGI:3037658 | H2afy2 | 2654.5 | 11706 | 8261.5 | 10090 | 3586.2 | |
| 1424923_at | MGI:105046 | Serpina3g | 2660 | 10687 | 17297 | 19590.5 | 1664.8 | |
| 1418115_s_at | MGI:3582695 | Tor1aip2 | 2662 | 6088 | 6775 | 7734 | 10315.5 | |
| 1443732_at | --- | | 2664.5 | 7335 | 28568 | 7377 | 52.5 | |
| 1434406_at | MGI:109605 | Srgap2 | 2668.5 | 14217 | 12924 | 19590.5 | 769 | |
| 1438062_at | MGI:2682305 | Rsf1 | 2670 | 22465.5 | 25203 | 37728.5 | 405 | |
| 1438125_at | MGI:2444267 | [illegible]30085N15F | 2683 | 5407 | 11493.5 | 5112 | 37 | |
| 1422547_at | MGI:96269 | Ranbp1 | 2698 | 7025 | 7504 | 5942 | 5344.6 | |
| 1446304_at | --- | | 2702 | 21881.5 | 19717.5 | 22426 | 41.8 | |
| 1420956_at | MGI:88039 | Apc | 2703 | 25687.5 | 21456 | 26273.5 | 1455.7 | |
| 1448590_at | MGI:88459 | Col6a1 | 2711.5 | 4518 | 13960 | 13041 | 638.6 | |
| 1448336_at | MGI:1343297 | Drg1 | 2719.5 | 19391 | 13789.5 | 20671 | 3515.2 | |
| 1422682_s_at | MGI:98839 | Prss1 | 2731 | 33641 | 35392.5 | 22671.5 | 185.3 | |
| 1438440_at | MGI:1261425 | Drosha | 2736.5 | 15852 | 23320.5 | 5350 | 18.8 | |
| 1436042_at | MGI:1099832 | Tln1 | 2742 | 22473 | 30330 | 29548.5 | 204.2 | |
| 1438061_at | MGI:1914897 | [illegible]30523C07F | 2748 | 9656 | 12971.5 | 22426 | 117.4 | |
| 1438037_at | --- | | 2758 | 23260.5 | 24376 | 37728.5 | 1589.9 | |
| 1425600_a_at | MGI:97613 | Plcb1 | 2760 | 22023 | 15228.5 | 5228 | 18.9 | |
| 1425115_at | MGI:894835 | Rbbp6 | 2764 | 4752 | 5130.5 | 5028.5 | 134.1 | |
| 1424166_at | MGI:109519 | Msh3 | 2766 | 6512 | 7857.5 | 5519 | 279.3 | |

| Probe | MGI_ID | Symbol | SW_rank | DL_rank | LS_rank | JTK_rank | Max-Min | Norm Plot |
|---|---|---|---|---|---|---|---|---|
| 1444645_at | --- | | 2769 | 11391 | 27507.5 | 20389.5 | 31.5 | |
| 1429720_at | MGI:1925939 | Naa35 | 2770.5 | 4790 | 5264 | 6650.5 | 2771.6 | |
| 1453077_a_at | MGI:1916338 | Snapc3 | 2773 | 7049 | 16547 | 23641.5 | 442.6 | |
| 1435810_at | MGI:1917817 | ’30455O13F | 2774 | 21609 | 21675 | 15195.5 | 1979.1 | |
| 1438899_at | MGI:2181068 | Klf17 | 2776 | 33486 | 27482.5 | 5350 | 51.4 | |
| 1453567_s_at | MGI:1920020 | Tmem216 | 2784 | 8025 | 8630 | 10749.5 | 1028.9 | |
| 1454742_at | MGI:2443755 | Rasgef1b | 2786 | 16165 | 20450 | 9316.5 | 1039 | |
| 1449289_a_at | MGI:88127 | B2m | 2789 | 13027 | 16674.5 | 25466.5 | 83523.3 | |
| 1416868_at | MGI:105388 | Cdkn2c | 2792 | 20798 | 22800 | 25466.5 | 772.4 | |
| 1432983_at | MGI:1917626 | ’00026H06F | 2794 | 10035.5 | 28699 | 10749.5 | 26 | |
| 1454065_at | MGI:1922574 | ?30562D21F | 2802.5 | 5529.5 | 6494 | 5389 | 20.9 | |
| 1423473_at | MGI:97298 | 02 Sep | 2808 | 36631 | 23320.5 | 26721 | 62.3 | |
| 1455705_at | MGI:2384577 | Tmem161a | 2812 | 24851.5 | 25060.5 | 37728.5 | 438.2 | |
| 1416210_at | MGI:1916119 | Imp3 | 2813 | 15826 | 6941 | 10749.5 | 3794.7 | |
| 1455827_at | MGI:2145597 | Mbnl2 | 2818 | 5645 | 7600 | 11130.5 | 6410.4 | |
| 1427170_at | --- | | 2819 | 18349 | 20111.5 | 25466.5 | 65 | |
| 1454971_x_at | --- | | 2825 | 7283 | 18038.5 | 37728.5 | 75511.1 | |
| 1416678_at | MGI:1349409 | Cops3 | 2828 | 6566 | 7104 | 5228 | 12281.6 | |
| 1428508_at | MGI:1914266 | Tbc1d2b | 2831 | 9354 | 13835 | 13264 | 1665.8 | |
| 1456872_a_at | --- | | 2832 | 7705 | 14689.5 | 6718 | 619.3 | |
| 1452534_a_at | MGI:96157 | Hmgb2 | 2835 | 19891 | 24415 | 22671.5 | 572.9 | |
| 1431934_at | MGI:1921959 | ?30505O20F | 2838 | 5404.5 | 29555 | 11529.5 | 34.9 | |
| 1436845_at | MGI:1270862 | Axin2 | 2840 | 5667 | 8359.5 | 5993.5 | 46.7 | |
| 1426377_at | MGI:3029290 | Zfp281 | 2840 | 20499 | 18014.5 | 27146 | 2052 | |
| 1436079_s_at | MGI:1928744 | Vapb | 2842 | 13992 | 22951.5 | 37728.5 | 355.1 | |
| 1418067_at | --- | | 2844 | 9332 | 6677 | 4814.5 | 10315.6 | |
| 1458381_at | MGI:1917912 | Clic5 | 2847 | 20229 | 21274.5 | 10257.5 | 42.9 | |

| Probe | MGI_ID | Symbol | SW_rank | DL_rank | LS_rank | JTK_rank | Max-Min | Norm Plot |
|---|---|---|---|---|---|---|---|---|
| 1444176_at | MGI:1924415 | Atp6v0d2 | 2851 | 23220 | 31321 | 18744 | 54.3 | 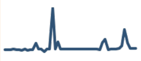 |
| 1430354_x_at | MGI:1931787 | Scyl1 | 2855 | 17331.5 | 22656 | 6282.5 | 19 | 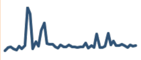 |
| 1429588_at | MGI:1914496 | 10474O19R | 2856 | 6674 | 14798.5 | 22186.5 | 3553.2 | 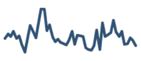 |
| 1415756_a_at | MGI:1333745 | Snapin | 2858 | 6774 | 8897.5 | 4814.5 | 1572 | 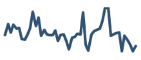 |
| 1451316_a_at | MGI:2385902 | Picalm | 2862 | 8187 | 8592 | 6915.5 | 9253.7 | 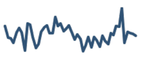 |
| 1434632_at | --- |  | 2866 | 4652 | 7379 | 9316.5 | 908.2 | 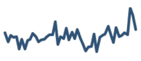 |
| 1418149_at | MGI:88394 | Chga | 2867 | 26545 | 34611 | 20951.5 | 29.8 | 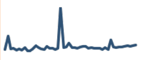 |
| 1420387_at | MGI:97138 | Mpv17 | 2879.5 | 7782 | 13378.5 | 8516.5 | 2193.2 | 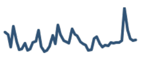 |
| 1454086_a_at | MGI:102811 | Lmo2 | 2879.5 | 22057.5 | 17244.5 | 23158.5 | 512.8 | 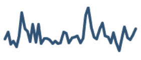 |
| 1448167_at | MGI:107655 | Ifngr1 | 2882 | 8810 | 9577.5 | 7734 | 943.9 | 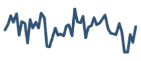 |
| 1418317_at | MGI:96785 | Lhx2 | 2884 | 5045 | 6589 | 11733 | 100.6 | 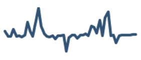 |
| 1448164_at | MGI:2651568 | Klhdc3 | 2888.5 | 20736.5 | 20056.5 | 26273.5 | 1655.4 | 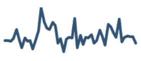 |
| 1416587_a_at | MGI:99137 | Xrcc1 | 2895.5 | 16921 | 22492 | 16503.5 | 773.6 | 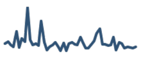 |
| 1458437_at | --- |  | 2895.5 | 36342 | 28194 | 23158.5 | 27.5 | 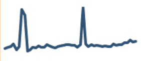 |
| 1449638_at | MGI:1923991 | 920021L13R | 2897 | 19874 | 23799.5 | 17603 | 46.6 | 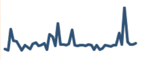 |
| 1438147_at | MGI:1924709 | Srcrb4d | 2900 | 9905 | 16187 | 16503.5 | 290.9 | 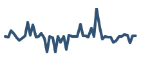 |
| 1453723_x_at | MGI:98888 | Ubb | 2909 | 10014 | 6831.5 | 4898 | 84190.8 | 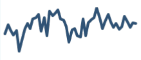 |
| 1430932_at | MGI:1924281 | Slc9a8 | 2915 | 6676 | 16746 | 37728.5 | 167.3 | 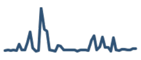 |
| 1448917_at | MGI:1917040 | Med30 | 2918 | 4798 | 5349.5 | 4898 | 2162 | 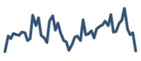 |
| 1434293_at | MGI:1923858 | Hectd3 | 2920 | 8166 | 17429 | 28851.5 | 97.6 | 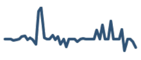 |
| 1441944_s_at | --- |  | 2927 | 13701 | 22696 | 4850.5 | 92.5 | 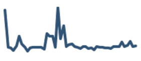 |
| 1425028_a_at | MGI:98810 | Tpm2 | 2928 | 7207 | 14442 | 14688 | 740.6 | 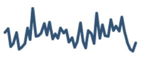 |
| 1430889_a_at | MGI:98812 | Tpmt | 2932 | 6231 | 9059.5 | 18465.5 | 15877.8 | 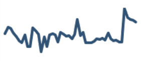 |
| 1452080_a_at | MGI:2150386 | Dcun1d1 | 2939 | 10938 | 15964.5 | 18465.5 | 5268.1 | 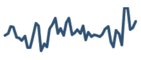 |
| 1419281_a_at | MGI:1330262 | Zfp259 | 2941 | 7576 | 7293 | 9198 | 3623.6 | 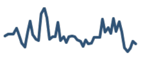 |
| 1424336_at | MGI:1914062 | Ppcdc | 2942 | 35696 | 40494 | 29548.5 | 58.8 | 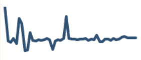 |
| 1427677_a_at | MGI:98368 | Sox6 | 2952 | 6424 | 15334 | 20671 | 1070.2 | 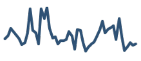 |

| Probe | MGI_ID | Symbol | SW_rank | DL_rank | LS_rank | JTK_rank | Max-Min | Norm Plot |
|---|---|---|---|---|---|---|---|---|
| 1444806_at | --- | | 2953 | 41766.5 | 35392.5 | 37728.5 | 30.9 | 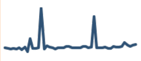 |
| 1423651_at | MGI:1916296 | Isca1 | 2959 | 12886 | 13567 | 9771 | 7754.3 | 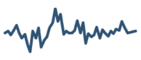 |
| 1449436_s_at | MGI:98888 | Ubb | 2979.5 | 12334 | 13430.5 | 10579.5 | 75946.9 | 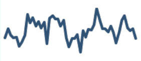 |
| 1427043_s_at | MGI:2384799 | Enox2 | 2981 | 15397 | 15964.5 | 25855 | 765.3 | 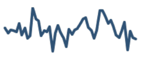 |
| 1429800_at | MGI:1924374 | I30221H12R | 2985 | 14237 | 22406 | 37728.5 | 442 | 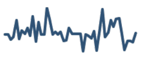 |
| 1447778_x_at | MGI:2389572 | Brcc3 | 2989 | 7732 | 9807.5 | 17907.5 | 869.4 | 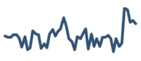 |
| 1417093_a_at | MGI:1338799 | Gtf2h4 | 2993 | 10277 | 10488.5 | 5844.5 | 971.8 | 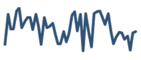 |
| 1454693_at | MGI:3036234 | Hdac4 | 2998 | 30548.5 | 31545 | 37728.5 | 27.5 | 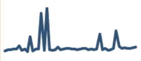 |
| 1430536_a_at | MGI:108089 | Erh | 2999 | 6033 | 4639.5 | 5555.5 | 8487.1 | 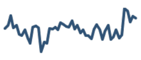 |
| 1454756_at | MGI:1917394 | Lrch3 | 3008 | 4874 | 13740 | 8323 | 569.2 | 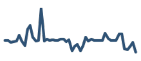 |
| 1435803_a_at | --- | | 3009 | 6591 | 4743.5 | 5745 | 4250.7 | 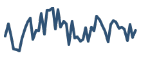 |
| 1460373_a_at | MGI:2136890 | Setd4 | 3013 | 6002 | 9347.5 | 16775 | 1001.3 | 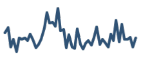 |
| 1446624_at | MGI:2179507 | Fktn | 3016.5 | 15142 | 14101.5 | 17046 | 130.9 | 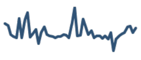 |
| 1433688_x_at | MGI:1914365 | Rpl14 | 3019.5 | 19596 | 16122.5 | 26060 | 35310.4 | 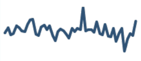 |
| 1428611_at | MGI:1921494 | Atg7 | 3021 | 28589.5 | 28479.5 | 37728.5 | 83.3 | 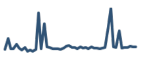 |
| 1441673_at | MGI:1915051 | Pllp | 3022 | 19081 | 40494 | 10257.5 | 94.2 | 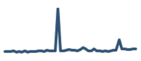 |
| 1417355_at | MGI:104748 | Peg3 | 3030 | 21411 | 26281.5 | 7377 | 389.2 | 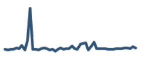 |
| 1455031_at | --- | | 3032 | 8959 | 18467.5 | 9609.5 | 515.8 | 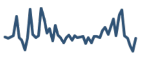 |
| 1429779_at | MGI:1924100 | Eif2c4 | 3035 | 6536 | 17320 | 7293.5 | 190 | 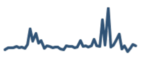 |
| 1448135_at | MGI:88096 | Atf4 | 3037 | 6761 | 5157.5 | 5475 | 5637.3 | 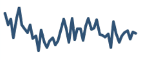 |
| 1459355_at | --- | | 3039 | 10477 | 18106 | 18465.5 | 13.2 | 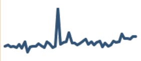 |
| 1446904_at | --- | | 3039 | 17106 | 14009 | 6718 | 59 | 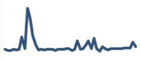 |
| 1418239_at | MGI:104539 | Apof | 3042 | 8317.5 | 9486 | 8516.5 | 51189.2 | 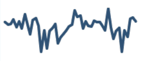 |
| 1422538_at | --- | | 3043 | 7164 | 6013 | 7377 | 1736.7 | 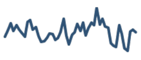 |
| 1423645_a_at | MGI:105037 | Ddx5 | 3046 | 8570 | 8359.5 | 7210.5 | 21796.4 | 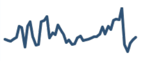 |
| 1443917_at | --- | | 3048.5 | 31173 | 32325 | 24799.5 | 19.8 | 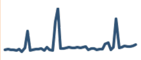 |
| 1428900_s_at | MGI:1924144 | Mettl15 | 3052.5 | 6611 | 5337 | 4989 | 1165.1 | 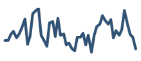 |

| Probe | MGI_ID | Symbol | SW_rank | DL_rank | LS_rank | JTK_rank | Max-Min | Norm Plot |
|---|---|---|---|---|---|---|---|---|
| 1436400_at | --- | | 3054 | 29963.5 | 29014.5 | 37728.5 | 158.6 | |
| 1435743_at | MGI:2683536 | Klhl23 | 3055 | 30885 | 29341.5 | 37728.5 | 56.3 | |
| 1448294_at | MGI:1929512 | Litaf | 3059 | 6126 | 11358 | 9085 | 154.2 | |
| 1448418_s_at | MGI:90168 | Dcaf11 | 3062 | 7769 | 6388 | 7377 | 24196.2 | |
| 1455607_at | MGI:1920030 | Rspo3 | 3064 | 7264 | 15057.5 | 20671 | 485.3 | |
| 1435330_at | MGI:2138243 | Pyhin1 | 3064 | 24427 | 32325 | 37728.5 | 307.3 | |
| 1432264_x_at | MGI:106015 | Cox7a2l | 3066 | 5156 | 4661.5 | 6224 | 9463 | |
| 1453433_at | MGI:1919588 | Wdr89 | 3072 | 9988 | 20666 | 7632 | 221.2 | |
| 1415863_at | MGI:109207 | Eif4g2 | 3075 | 32556.5 | 26503.5 | 29740 | 23322.7 | |
| 1454624_at | MGI:2444961 | Dennd2a | 3076 | 10805.5 | 31464 | 9771 | 18.6 | |
| 1433830_at | --- | | 3077 | 19496 | 21097 | 14192.5 | 10746.9 | |
| 1427334_s_at | MGI:1914496 | 310474O19R | 3079 | 7201 | 18847.5 | 20130 | 2401 | |
| 1443422_at | --- | | 3081 | 18314 | 30074 | 37728.5 | 191.5 | |
| 1452597_at | MGI:1913781 | 310061C15R | 3082.5 | 9219 | 7705.5 | 7377 | 2889.3 | |
| 1447181_s_at | MGI:1337120 | Slc7a7 | 3084 | 27073 | 27577 | 26491 | 19.9 | |
| 1430338_at | MGI:1921583 | 122401K19R | 3087 | 41631 | 40494 | 37728.5 | 114 | |
| 1435722_at | MGI:95811 | Gria4 | 3088 | 9802.5 | 19236 | 15195.5 | 14.8 | |
| 1456614_at | MGI:1913288 | Acn9 | 3094 | 6462 | 5076 | 7734 | 3415.8 | |
| 1453162_at | MGI:1914455 | Utp11l | 3097 | 8117 | 13876.5 | 11946.5 | 1478.1 | |
| 1454865_at | MGI:1924281 | Slc9a8 | 3098 | 9945 | 13567 | 6990.5 | 257.4 | |
| 1429658_a_at | MGI:106067 | Smc2 | 3104 | 17541 | 15461 | 6718 | 271.5 | |
| 1429698_at | MGI:3704243 | Gm9897 | 3107 | 6795 | 19188 | 21938.5 | 488.4 | |
| 1436393_a_at | MGI:2153072 | Trim37 | 3108 | 6432 | 10632 | 5993.5 | 205.8 | |
| 1434090_at | MGI:2442555 | Dis3l2 | 3111 | 27308 | 27254.5 | 37728.5 | 267.6 | |
| 1438007_at | MGI:2143691 | Fam19a2 | 3112 | 21323 | 30730.5 | 18192 | 95.3 | |
| 1418650_at | MGI:1915196 | Spata6 | 3113 | 9102.5 | 14147.5 | 9609.5 | 19.4 | |
| 1452992_at | MGI:1913690 | Cdc26 | 3114.5 | 6802 | 15668 | 5228 | 374.6 | |

| Probe | MGI_ID | Symbol | SW_rank | DL_rank | LS_rank | JTK_rank | Max-Min | Norm Plot |
|---|---|---|---|---|---|---|---|---|
| 1417245_at | MGI:1930949 | Gpr180 | 3116 | 9305 | 7511 | 5150 | 1475.8 | 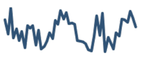 |
| 1424019_at | MGI:107891 | Nop2 | 3117 | 7538 | 7688 | 7544 | 1441.8 | 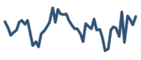 |
| 1455942_at | MGI:1354736 | Kdm2a | 3120 | 9569 | 15057.5 | 20951.5 | 385.5 | 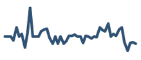 |
| 1454760_at | MGI:1919709 | Htatsf1 | 3125 | 12598 | 10762.5 | 6650.5 | 2560 | 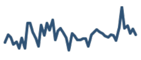 |
| 1423698_at | MGI:1289164 | Ncaph2 | 3126 | 12898 | 5056.5 | 6050.5 | 1634.8 | 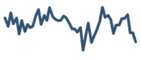 |
| 1430369_at | --- | | 3127.5 | 5968 | 14651 | 8022 | 131 | 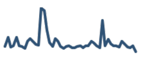 |
| 1457154_at | --- | | 3127.5 | 34767.5 | 34611 | 37728.5 | 73.3 | 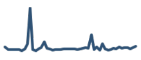 |
| 1423454_a_at | MGI:1338032 | Sema6c | 3133.5 | 22642 | 26398.5 | 8619 | 14.3 | 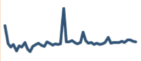 |
| 1433905_at | --- | | 3136 | 23499.5 | 21508.5 | 23873.5 | 167.3 | 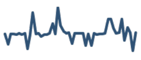 |
| 1431665_a_at | MGI:1353424 | Timm8b | 3138 | 7126 | 6677 | 6785.5 | 8768.8 | 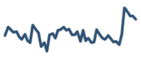 |
| 1452397_at | MGI:1914496 | [illegible]10474O19R[illegible] | 3139 | 9589 | 18764.5 | 37728.5 | 516.8 | 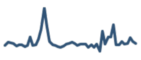 |
| 1443824_s_at | MGI:103100 | Car7 | 3141 | 41453.5 | 40494 | 37728.5 | 9.9 | 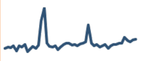 |
| 1453314_x_at | MGI:1913828 | [illegible]10039C10R[illegible] | 3144 | 19864 | 24561.5 | 29740 | 748.1 | 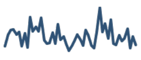 |
| 1435917_at | --- | | 3145 | 9215 | 6768 | 5309 | 2712.7 | 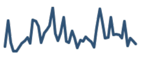 |
| 1449800_x_at | MGI:1919088 | Phf7 | 3148 | 7768 | 10262 | 12382 | 934.4 | 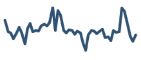 |
| 1452704_at | MGI:1915059 | Fam82a2 | 3151 | 6140 | 5044.5 | 5555.5 | 4623.5 | 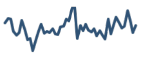 |
| 1451405_at | MGI:97499 | Pcca | 3155 | 6809 | 5860 | 6282.5 | 5708.9 | 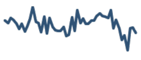 |
| 1451903_at | MGI:1918039 | Kynu | 3156 | 11016 | 13612.5 | 4611.5 | 132.3 | 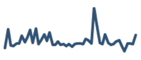 |
| 1434597_at | MGI:106330 | Larp4b | 3159 | 5759 | 5471.5 | 8958.5 | 6122.8 | 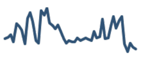 |
| 1426187_a_at | MGI:1346319 | Hax1 | 3159 | 9335 | 10411.5 | 12823.5 | 7556 | 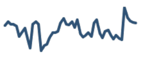 |
| 1443695_at | MGI:1196378 | Habp2 | 3161 | 10323 | 14651 | 15710 | 1569.6 | 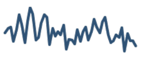 |
| 1455091_at | MGI:1925103 | Msl2 | 3162.5 | 5946 | 16122.5 | 17315 | 821.5 | 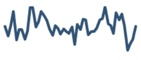 |
| 1432483_at | MGI:1924294 | Arid2 | 3167 | 8999.5 | 19616.5 | 5696 | 25.4 | 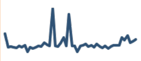 |
| 1434864_at | MGI:2442058 | Nipa1 | 3173.5 | 18120 | 19863.5 | 5993.5 | 33.8 | 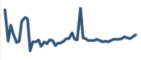 |
| 1422852_at | MGI:1929293 | Cib2 | 3176.5 | 16774 | 26059.5 | 37728.5 | 312.1 | 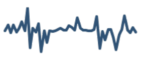 |
| 1438118_x_at | MGI:98932 | Vim | 3179 | 24855.5 | 11358 | 7210.5 | 3882.2 | 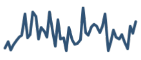 |
| 1419760_a_at | MGI:1858215 | Rwdd2b | 3180 | 8554 | 8947 | 5389 | 655.4 | 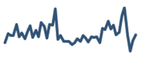 |

| Probe | MGI_ID | Symbol | SW_rank | DL_rank | LS_rank | JTK_rank | Max-Min | Norm Plot |
|---|---|---|---|---|---|---|---|---|
| 1438488_at | MGI:95421 | Esd | 3184 | 24052 | 10947.5 | 6526 | 136.6 | 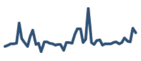 |
| 1433550_at | --- | | 3187 | 5226 | 6115.5 | 7131.5 | 1778.4 | 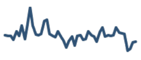 |
| 1426487_a_at | MGI:894835 | Rbbp6 | 3188 | 7972 | 16573 | 24106 | 1370.5 | 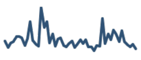 |
| 1428169_at | MGI:1924290 | Atg16l1 | 3189 | 15837 | 7314 | 9771 | 593.2 | 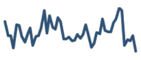 |
| 1428584_a_at | MGI:1919877 | Haghl | 3190.5 | 26310.5 | 28310 | 37728.5 | 161.4 | 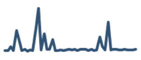 |
| 1424325_at | MGI:1925055 | Esco1 | 3192 | 5896.5 | 15362 | 11130.5 | 1689.5 | 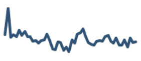 |
| 1415917_at | MGI:1342005 | Mthfd1 | 3195 | 5754 | 7137 | 8727 | 14016.3 | 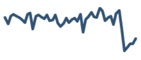 |
| 1418801_at | MGI:1921820 | Zkscan1 | 3196.5 | 13190 | 18728 | 12823.5 | 1412.8 | 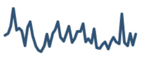 |
| 1438493_at | MGI:2687283 | Pld6 | 3200 | 12380 | 19309.5 | 9456 | 23.7 | 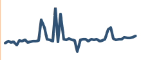 |
| 1451269_at | MGI:1919871 | Pdzd11 | 3208 | 5767 | 5981.5 | 6282.5 | 2005.8 | 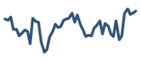 |
| 1419645_at | MGI:1343054 | Cstf2 | 3209.5 | 6138 | 5088 | 4947.5 | 1491.1 | 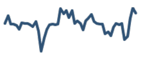 |
| 1430568_at | MGI:1914552 | Zc3h13 | 3213 | 12061 | 18214 | 9929 | 565.6 | 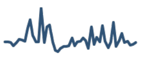 |
| 1445168_at | --- | | 3216 | 26638.5 | 13651 | 5895.5 | 80 | 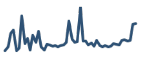 |
| 1435291_at | --- | | 3217 | 6594 | 5175.5 | 5844.5 | 1254.5 | 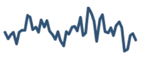 |
| 1433345_s_at | MGI:1921912 | ?30448K20R | 3218 | 5529.5 | 15015 | 7632 | 36.7 | 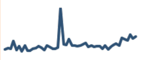 |
| 1442209_at | --- | | 3224.5 | 6278.5 | 31902 | 6990.5 | 17.8 | 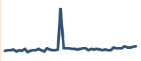 |
| 1457225_at | --- | | 3224.5 | 19515 | 23577.5 | 7463.5 | 23.6 | 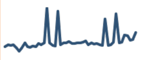 |
| 1434854_a_at | MGI:1914347 | Rps10 | 3228.5 | 18571 | 17076 | 12382 | 28936.2 | 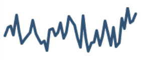 |
| 1420500_at | MGI:103268 | Dnajc1 | 3235 | 28861.5 | 25524.5 | 27146 | 4369.6 | 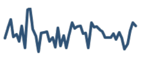 |
| 1448780_at | --- | | 3236 | 6223 | 9375 | 9771 | 1221.6 | 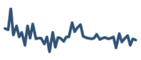 |
| 1417677_at | MGI:1338022 | Opn3 | 3246 | 36143.5 | 35392.5 | 37728.5 | 89.1 | 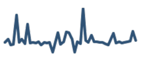 |
| 1448182_a_at | MGI:88323 | Cd24a | 3247.5 | 5948 | 21675 | 13264 | 4291.9 | 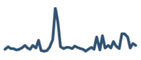 |
| 1459920_at | MGI:1918329 | ?33415E08R | 3247.5 | 7187 | 6467.5 | 15975.5 | 334.5 | 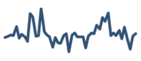 |
| 1442732_at | MGI:2136381 | Hadhb | 3249 | 7639 | 21928 | 37728.5 | 246.9 | 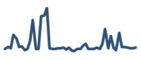 |
| 1447700_x_at | --- | | 3250 | 7618 | 20550.5 | 9456 | 807.6 | 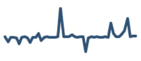 |
| 1451600_s_at | MGI:3644960 | Ces3b | 3251 | 8619.5 | 10271 | 9456 | 106483 | 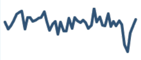 |
| 1444029_at | MGI:2141505 | Parp11 | 3254.5 | 40469.5 | 40494 | 37728.5 | 25.4 | 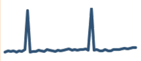 |

| Probe | MGI_ID | Symbol | SW_rank | DL_rank | LS_rank | JTK_rank | Max-Min | Norm Plot |
|---|---|---|---|---|---|---|---|---|
| 1451676_at | MGI:1913806 | Drap1 | 3265 | 12347 | 12140.5 | 6915.5 | 4512.5 | |
| 1419217_at | MGI:1351630 | Sergef | 3267 | 7696 | 15130.5 | 11332.5 | 180.4 | |
| 1454564_at | MGI:1918292 | 933401J01R | 3269 | 5367 | 7617.5 | 5185 | 34.4 | |
| 1436272_at | MGI:1919586 | Iars2 | 3278 | 12574 | 16746 | 7293.5 | 283.9 | |
| 1425474_a_at | MGI:2443189 | Vps39 | 3282 | 26262 | 28627 | 27146 | 132.9 | |
| 1424410_at | MGI:1923510 | Ttc8 | 3285 | 10616 | 13960 | 8123.5 | 552.4 | |
| 1448884_at | MGI:1915403 | Gtf2e2 | 3287.5 | 6199 | 7000 | 6282.5 | 2508.9 | |
| 1428873_a_at | MGI:1921276 | Msl1 | 3289 | 9226 | 22228.5 | 13728 | 842.5 | |
| 1421379_at | MGI:1351476 | Zfp354b | 3292 | 7288 | 15057.5 | 21448 | 260.2 | |
| 1444503_at | --- | | 3294 | 4572 | 16547 | 18744 | 275.5 | |
| 1424237_at | MGI:1915028 | Zfp639 | 3295 | 20086 | 21192 | 25035 | 1051.9 | |
| 1417313_at | MGI:1913344 | Lsm7 | 3297.5 | 17385 | 10632 | 10090 | 1423.4 | |
| 1435389_at | MGI:2663511 | Reps2 | 3300 | 19094.5 | 15927.5 | 26491 | 1257.6 | |
| 1427269_at | MGI:1916457 | Srsf11 | 3301 | 8078 | 10488.5 | 13728 | 3512.1 | |
| 1452612_at | MGI:1926163 | Ltn1 | 3304 | 28623.5 | 26419.5 | 37728.5 | 1695.3 | |
| 1427087_at | MGI:2183260 | Luc7l2 | 3306 | 4861 | 5463 | 6526 | 3071.5 | |
| 1453616_at | MGI:1917940 | 330408C21R | 3307 | 7736 | 10947.5 | 6526 | 43 | |
| 1454316_at | MGI:1926077 | 330426C09R | 3310 | 18419 | 22207.5 | 21692.5 | 29.6 | |
| 1455232_at | MGI:2136446 | Cml2 | 3316 | 15818 | 14943 | 10419.5 | 43262.3 | |
| 1451484_a_at | MGI:98460 | Syn1 | 3320 | 5529.5 | 18087 | 9316.5 | 6.7 | |
| 1455515_at | MGI:1919551 | 310041L15R | 3322 | 39351 | 31464 | 37728.5 | 21.6 | |
| 1460544_at | MGI:1925939 | Naa35 | 3333 | 8525 | 8592 | 6526 | 1098.4 | |
| 1423837_at | MGI:1919205 | 400003C14R | 3336 | 21881.5 | 11358 | 8516.5 | 685.6 | |
| 1435121_at | MGI:2664395 | Dio3os | 3339 | 32434 | 17614 | 37728.5 | 105.5 | |
| 1455637_x_at | MGI:3643396 | 230307C23R | 3340 | 4955.5 | 4661.5 | 4814.5 | 249.2 | |
| 1430438_at | --- | | 3342 | 40027.5 | 35392.5 | 37728.5 | 57.9 | |
| 1435075_at | MGI:1919150 | Tmem106b | 3348 | 19003 | 16489 | 9771 | 4992.9 | |

| Probe | MGI_ID | Symbol | SW_rank | DL_rank | LS_rank | JTK_rank | Max-Min | Norm Plot |
|---|---|---|---|---|---|---|---|---|
| 1422996_at | MGI:2159605 | Acot2 | 3350 | 14924 | 21290 | 9609.5 | 108.2 | 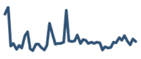 |
| 1448140_at | MGI:1922083 | Ciapin1 | 3357 | 21274 | 12198 | 11529.5 | 984.7 | 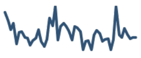 |
| 1427573_at | MGI:1344694 | Chic1 | 3358 | 10966 | 26166.5 | 6526 | 130.6 | 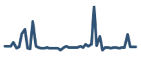 |
| 1452683_at | MGI:1915848 | Dnajc8 | 3363 | 16563 | 17429 | 14688 | 2253.5 | 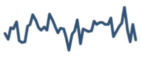 |
| 1453257_at | MGI:1196345 | Agpat5 | 3371 | 10930 | 18282 | 24106 | 1812.3 | 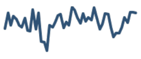 |
| 1417164_at | MGI:1927070 | Dusp10 | 3383.5 | 21814.5 | 33352 | 37728.5 | 241.8 | 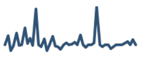 |
| 1450435_at | MGI:96721 | L1cam | 3386 | 9265 | 18749 | 29191.5 | 60.3 | 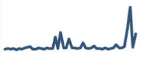 |
| 1454863_at | MGI:1924337 | Ankrd11 | 3387 | 10346 | 17905 | 7066 | 795.1 | 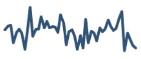 |
| 1434884_at | MGI:1914404 | Mtdh | 3388 | 7071 | 7720.5 | 7734 | 2887.5 | 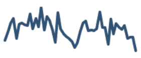 |
| 1416356_at | MGI:1917903 | Gmpr2 | 3397.5 | 6681 | 4848 | 5309 | 833.2 | 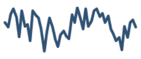 |
| 1443193_at | MGI:2443047 | Ttll1 | 3397.5 | 43970 | 32549.5 | 37728.5 | 32.9 | 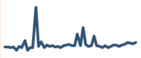 |
| FX-MURINE_B2_ | NA | NA | 3399 | 9472.5 | 5166.5 | 4814.5 | 100233 | 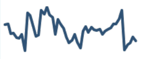 |
| 1443626_at | --- | | 3400 | 5734 | 21097 | 17046 | 497.2 | 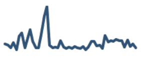 |
| 1442239_at | --- | | 3401 | 23376 | 7119 | 9085 | 61.1 | 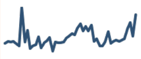 |
| 1435478_at | --- | | 3402.5 | 5633 | 4622.5 | 5228 | 6140.5 | 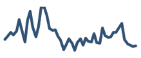 |
| 1449175_at | MGI:108031 | Gpr65 | 3404 | 6402 | 15999.5 | 21938.5 | 291.6 | 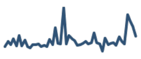 |
| 1426666_a_at | MGI:1924303 | Sun1 | 3405 | 16019 | 15595 | 5895.5 | 612.4 | 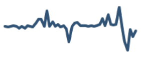 |
| 1428505_at | MGI:1913615 | Ccdc90b | 3408 | 10981 | 13835 | 7210.5 | 2534.9 | 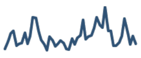 |
| 1442156_at | MGI:2442914 | 030030I06R | 3409 | 10295 | 15425 | 11529.5 | 54.8 | 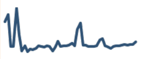 |
| 1453776_at | MGI:1917729 | Snx21 | 3410 | 5956 | 16489 | 26273.5 | 513.8 | 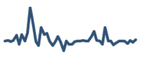 |
| 1434715_at | MGI:1919494 | 00014C10R | 3417 | 17223 | 18087 | 37728.5 | 189.7 | 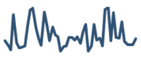 |
| 1457964_at | MGI:1917048 | 10044D09R | 3419 | 20787 | 25040 | 25035 | 497.6 | 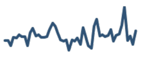 |
| 1423886_at | MGI:99914 | Lamc1 | 3422 | 25562 | 26601.5 | 28255 | 74.5 | 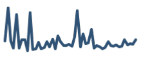 |
| 1452508_x_at | MGI:1916452 | Ptms | 3432.5 | 24427 | 26200.5 | 24341 | 889.1 | 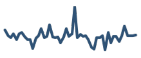 |
| 1424327_at | MGI:1922680 | 00002M19R | 3435 | 10656 | 11243.5 | 9771 | 1169.2 | 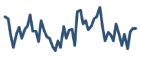 |
| 1448698_at | MGI:88313 | Ccnd1 | 3439 | 5753 | 13086 | 10090 | 1497.5 | 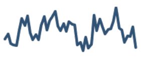 |
| 1446050_at | --- | | 3441.5 | 8975 | 29520.5 | 24799.5 | 221.1 | 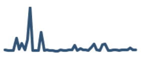 |

| Probe | MGI_ID | Symbol | SW_rank | DL_rank | LS_rank | JTK_rank | Max-Min | Norm Plot |
|---|---|---|---|---|---|---|---|---|
| 1452622_a_at | MGI:109200 | Tradd | 3443.5 | 6032 | 4914.5 | 6394 | 1302 | |
| 1421098_at | MGI:1926193 | Stap1 | 3447.5 | 5300.5 | 8511.5 | 6650.5 | 40.9 | |
| 1428618_at | MGI:1915183 | Hcfc2 | 3447.5 | 20486 | 25753.5 | 28255 | 452.8 | |
| 1439323_a_at | --- | | 3451 | 20802 | 27696 | 9198 | 32.3 | |
| 1425159_at | MGI:1915588 | Golt1a | 3452 | 11708 | 14527 | 20671 | 1018.9 | |
| 1419168_at | MGI:1354946 | Mapk6 | 3457 | 28796.5 | 29137.5 | 37728.5 | 247.1 | |
| 1452726_a_at | MGI:1916095 | Pih1d1 | 3466 | 6745 | 7868.5 | 6650.5 | 2045.7 | |
| 1438188_x_at | MGI:2444911 | Slc25a29 | 3467 | 6912.5 | 17026.5 | 7734 | 10.8 | |
| 1424819_a_at | MGI:2141866 | Ric8 | 3470 | 9974 | 5637 | 9771 | 492.2 | |
| 1460665_a_at | MGI:1298230 | Cnot7 | 3474 | 14501 | 7242.5 | 12163.5 | 647.8 | |
| 1448814_at | MGI:108088 | Gab1 | 3475 | 23343 | 10762.5 | 5645.5 | 119.8 | |
| 1435884_at | MGI:1338069 | Itsn1 | 3476 | 5755 | 6547.5 | 6718 | 1290.7 | |
| 1423700_at | MGI:1916513 | Rfc3 | 3479 | 5246 | 7024 | 9609.5 | 550.1 | |
| 1436625_at | MGI:95498 | Fcgr1 | 3480 | 35173 | 28506.5 | 13494 | 46 | |
| 1419549_at | MGI:88070 | Arg1 | 3484 | 8228.5 | 9337 | 7066 | 93920.3 | |
| 1415745_a_at | MGI:1206040 | Dscr3 | 3495 | 11626 | 11762 | 9771 | 2079.9 | |
| 1427568_a_at | MGI:1915509 | Ift80 | 3496 | 8558 | 17925.5 | 22426 | 202 | |
| 1442464_at | --- | | 3502 | 24431.5 | 31105.5 | 37728.5 | 75.6 | |
| 1426882_at | MGI:2140998 | Ube3c | 3503 | 8496 | 12550.5 | 8323 | 1099.6 | |
| 1424656_s_at | MGI:1918722 | Usp19 | 3506 | 7276 | 12924 | 5942 | 728.4 | |
| 1444766_at | MGI:3584458 | Atxn7l1 | 3509 | 30964 | 32982.5 | 37728.5 | 259.9 | |
| 1429937_at | MGI:1925831 | Utp23 | 3510 | 28800 | 31545 | 12823.5 | 40.2 | |
| 1435739_at | MGI:1333883 | Lats1 | 3511 | 4542 | 4951 | 7066 | 3539.2 | |
| 1455545_at | MGI:1916170 | 110065P20R | 3512 | 7081 | 8717.5 | 8323 | 3561.4 | |
| 1436475_at | --- | | 3516 | 7099 | 9486 | 8123.5 | 876.3 | |
| 1428694_at | MGI:1923207 | Mir17hg | 3517 | 7091 | 22818.5 | 17046 | 160.3 | |
| 1425684_at | MGI:1915111 | Akr1b10 | 3519 | 39528 | 26337 | 21938.5 | 50.4 | |

| Probe | MGI_ID | Symbol | SW_rank | DL_rank | LS_rank | JTK_rank | Max-Min | Norm Plot |
|---|---|---|---|---|---|---|---|---|
| 1438253_at | MGI:2686240 | Ssh1 | 3520.5 | 6238 | 13143.5 | 14939.5 | 87.4 | |
| 1430655_at | MGI:1918050 | 531405K08R | 3520.5 | 43875 | 40494 | 26491 | 30.4 | |
| 1426748_s_at | MGI:1351656 | Abcf3 | 3525 | 30012 | 32219.5 | 37728.5 | 271.5 | |
| 1428747_at | MGI:1919037 | Trnau1ap | 3527 | 5814 | 10488.5 | 5555.5 | 4086.5 | |
| 1435792_at | --- | | 3528 | 8463 | 9403.5 | 9198 | 78.9 | |
| 1418540_a_at | MGI:97813 | Ptpre | 3529 | 14891 | 24765.5 | 37728.5 | 83.3 | |
| 1426922_s_at | MGI:1333754 | Agfg1 | 3533 | 24180.5 | 8376 | 13958.5 | 294.3 | |
| 1447752_x_at | MGI:1343297 | Drg1 | 3536 | 6269 | 7907.5 | 8420 | 227.3 | |
| 1421286_a_at | MGI:88113 | Atp4a | 3537 | 18676.5 | 19831 | 29740 | 15 | |
| 1424500_at | MGI:2445193 | Utp6 | 3539 | 7145 | 11835 | 13264 | 2253.5 | |
| 1417660_s_at | MGI:1928344 | Vps29 | 3540.5 | 17698 | 18629 | 19590.5 | 25362.5 | |
| 1440914_s_at | --- | | 3540.5 | 28254.5 | 28149 | 9929 | 221.2 | |
| 1460737_at | MGI:1346500 | Igbp1 | 3542 | 9577 | 12872 | 7925.5 | 4884.1 | |
| 1419247_at | MGI:1098271 | Rgs2 | 3543 | 6429 | 7153.5 | 9609.5 | 126.6 | |
| 1455697_at | --- | | 3545 | 20305 | 17667 | 11130.5 | 549.9 | |
| 1449643_s_at | MGI:1202875 | Btf3 | 3549 | 12313 | 13238.5 | 16775 | 20221.9 | |
| 1419666_x_at | MGI:1891834 | Nupr1 | 3553.5 | 38907 | 40494 | 37728.5 | 99.6 | |
| 1431191_a_at | MGI:99667 | Syt1 | 3556 | 11707 | 20645 | 15710 | 114.5 | |
| 1441545_at | --- | | 3560 | 7698 | 26768.5 | 29364.5 | 187.1 | |
| 1417566_at | MGI:1914719 | Abhd5 | 3561 | 6440.5 | 7490 | 6394 | 3592 | |
| 1437265_at | MGI:3026931 | 830438D12R | 3564 | 35978 | 35392.5 | 37728.5 | 56.7 | |
| 1417539_at | MGI:1345622 | Slc35a1 | 3570 | 7243 | 8491.5 | 11946.5 | 3243.7 | |
| 1418967_a_at | MGI:1927450 | St7 | 3571 | 29424 | 35392.5 | 37728.5 | 377.5 | |
| 1443991_at | MGI:2429765 | Dock1 | 3575.5 | 9251 | 20722.5 | 5793.5 | 164.8 | |
| 1431692_a_at | MGI:1931457 | Cblc | 3575.5 | 24851.5 | 25801 | 27698 | 180.8 | |
| 1434170_at | MGI:2444462 | Dcaf12l1 | 3582 | 7542 | 11243.5 | 9085 | 122.1 | |
| 1435153_at | MGI:3026623 | Btbd6 | 3583 | 16016 | 9087 | 6850.5 | 512.1 | |

| Probe | MGI_ID | Symbol | SW_rank | DL_rank | LS_rank | JTK_rank | Max-Min | Norm Plot |
|---|---|---|---|---|---|---|---|---|
| 1445562_at | --- | | 3584.5 | 5643 | 19591 | 22671.5 | 192.2 | 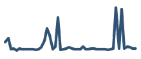 |
| 1428546_at | MGI:1891690 | Syncrip | 3587 | 7552 | 6768 | 8958.5 | 2547.5 | 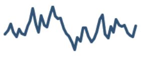 |
| 1421236_at | MGI:1891456 | Ripk2 | 3591 | 21342 | 20974 | 9609.5 | 32.8 | 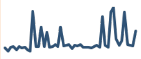 |
| 1424144_at | MGI:1914427 | Cdt1 | 3592 | 15590 | 25344 | 11733 | 95.7 | 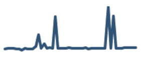 |
| 1429988_at | MGI:1929117 | Zfp235 | 3593 | 11034 | 17553.5 | 13494 | 233.9 | 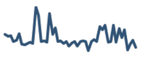 |
| 1419955_at | MGI:1096572 | Zfand3 | 3594 | 31451 | 33826 | 37728.5 | 347.9 | 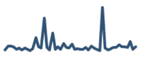 |
| 1443029_at | --- | | 3598 | 7017 | 22359.5 | 12606 | 199 | 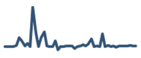 |
| 1438159_x_at | MGI:1920150 | Ndufv2 | 3600 | 7557 | 8445 | 8516.5 | 29793.3 | 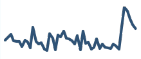 |
| 1455305_x_at | --- | | 3603 | 6561 | 7852 | 7734 | 3403 | 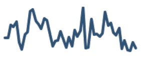 |
| 1443052_at | MGI:3780549 | Gm2381 | 3604 | 37126.5 | 31632 | 37728.5 | 59.4 | 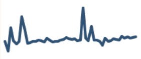 |
| 1453689_at | MGI:1920025 | Fance | 3608.5 | 9102.5 | 14565.5 | 16775 | 16.3 | 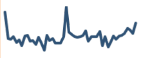 |
| 1419087_s_at | MGI:1914715 | Sf3a1 | 3610.5 | 17331.5 | 14442 | 4850.5 | 11.9 | 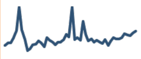 |
| 1448204_at | MGI:1927144 | Sav1 | 3610.5 | 27260.5 | 26891 | 37728.5 | 1375.9 | 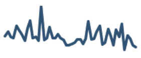 |
| 1428275_at | MGI:1916154 | Abhd13 | 3612 | 14207 | 22584 | 20671 | 1297.9 | 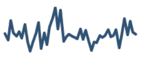 |
| 1422607_at | MGI:99254 | Etv1 | 3615.5 | 5529.5 | 26525 | 8123.5 | 15.1 | 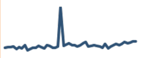 |
| 1452067_at | MGI:1914361 | Naaa | 3615.5 | 12194.5 | 24100.5 | 13958.5 | 12.4 | 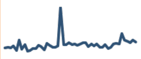 |
| 1432786_at | MGI:1921016 | 333420D23R | 3619 | 29652 | 31390.5 | 22916 | 59.1 | 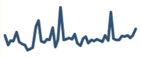 |
| 1437882_s_at | --- | | 3620 | 27746 | 29590 | 37728.5 | 113.3 | 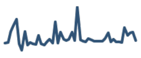 |
| 1447027_s_at | MGI:1934604 | Lias | 3622 | 5085 | 4530.5 | 5645.5 | 7073.5 | 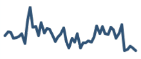 |
| 1429270_a_at | MGI:1919096 | Syce2 | 3624 | 9228 | 24750 | 37728.5 | 484.9 | 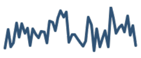 |
| 1416391_at | MGI:1919049 | Ptcd1 | 3629 | 16922 | 20865 | 29740 | 376.1 | 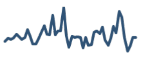 |
| 1448216_at | MGI:1341881 | Syngr3 | 3631 | 18167.5 | 21704 | 10257.5 | 13.3 | 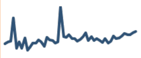 |
| 1437997_x_at | MGI:1289321 | Mrpl48 | 3632 | 6084 | 6486.5 | 7544 | 2197.2 | 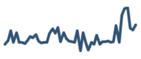 |
| 1452657_at | MGI:1889383 | Ap1s2 | 3634 | 6766 | 14763.5 | 5993.5 | 166.5 | 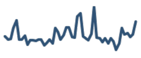 |
| 1447880_x_at | --- | | 3641 | 8510 | 19372.5 | 26721 | 101.1 | 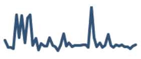 |
| 1432330_at | MGI:3647418 | Cfhr3 | 3642 | 7724 | 13789.5 | 7544 | 466.3 | 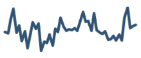 |
| 1423007_a_at | MGI:1195462 | Gfra2 | 3644 | 7790 | 28385.5 | 9771 | 46.4 | 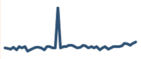 |

| Probe | MGI_ID | Symbol | SW_rank | DL_rank | LS_rank | JTK_rank | Max-Min | Norm Plot |
|---|---|---|---|---|---|---|---|---|
| 1431089_at | MGI:1861601 | Cpsf2 | 3646.5 | 20880 | 27343.5 | 16234 | 96.1 | 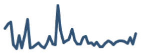 |
| 1440339_at | MGI:97370 | Enpp1 | 3648 | 6393 | 5088 | 5645.5 | 745.9 | 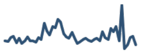 |
| 1431133_at | MGI:1921160 | Arhgap18 | 3649 | 9979 | 26955 | 28453 | 435 | 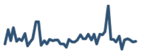 |
| 1428519_at | MGI:1913747 | 510528E23R | 3650 | 26442.5 | 29996 | 37728.5 | 458.7 | 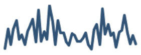 |
| 1452608_at | MGI:1891750 | Mycbp | 3651.5 | 5082 | 5188.5 | 7925.5 | 7141.7 | 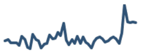 |
| 1427331_at | MGI:99401 | Adora1 | 3653.5 | 7647 | 15873 | 8224.5 | 57.2 | 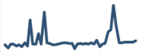 |
| 1439071_at | MGI:1918676 | 430416N02R | 3653.5 | 23833.5 | 24212 | 37728.5 | 431.2 | 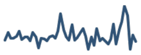 |
| 1425239_at | MGI:2136890 | Setd4 | 3657 | 8852 | 20550.5 | 28065.5 | 1138.9 | 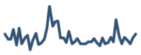 |
| 1417086_at | MGI:109520 | Pafah1b1 | 3659 | 8111 | 10562 | 17907.5 | 2212.9 | 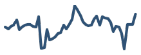 |
| 1434426_at | MGI:2142989 | Ncapd3 | 3660 | 11005 | 20090 | 19590.5 | 409.9 | 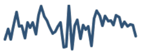 |
| 1424154_a_at | MGI:1921566 | Isca2 | 3661 | 7664 | 7609.5 | 4814.5 | 8915.6 | 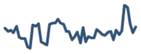 |
| 1435278_at | --- | | 3665.5 | 11625 | 20008 | 24799.5 | 666.2 | 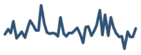 |
| 1423910_at | MGI:2183446 | Agap3 | 3668 | 7967 | 15362 | 17907.5 | 518 | 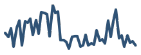 |
| 1419034_at | MGI:88543 | Csnk2a1 | 3671 | 6144 | 8620 | 7377 | 1276.8 | 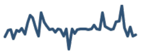 |
| 1455027_at | MGI:106484 | Rufy3 | 3678.5 | 29236 | 25653.5 | 37728.5 | 106.3 | 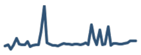 |
| 1428183_at | --- | | 3683 | 41410.5 | 35392.5 | 24565 | 306.3 | 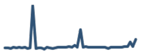 |
| 1431566_at | MGI:1918820 | 030622O22R | 3685.5 | 12854 | 20301 | 37728.5 | 402.6 | 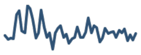 |
| 1441940_x_at | MGI:2442630 | Klhdc8a | 3685.5 | 14017 | 21003.5 | 9456 | 25.8 | 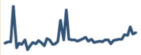 |
| 1433737_at | MGI:1341908 | Uhmk1 | 3688 | 26705 | 24486.5 | 18465.5 | 2414.5 | 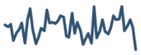 |
| 1427901_at | MGI:1915985 | Mrps18c | 3692 | 6117 | 5511 | 8958.5 | 18655.1 | 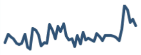 |
| 1450232_at | MGI:107572 | Xiap | 3692 | 10973.5 | 13194 | 12823.5 | 57.2 | 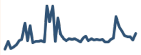 |
| 1460345_at | MGI:1919737 | Aida | 3692 | 32049.5 | 35392.5 | 37728.5 | 123.1 | 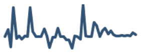 |
| 1428495_at | MGI:1922843 | 410003K15R | 3695 | 6530 | 5361.5 | 5555.5 | 5818.2 | 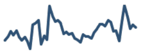 |
| 1434415_at | MGI:3654828 | Dact3 | 3696 | 5529.5 | 12198 | 9085 | 31.3 | 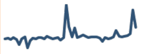 |
| 1428463_a_at | MGI:1349473 | Ppp2r5e | 3698 | 6045 | 9140.5 | 17907.5 | 17175.8 | 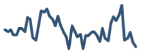 |
| 1419299_at | MGI:1913689 | 010012O05R | 3703 | 14697 | 13740 | 9771 | 1416 | 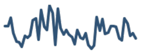 |
| 1449758_at | --- | | 3704 | 7783 | 9906 | 10257.5 | 53.8 | 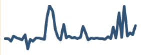 |

| Probe | MGI_ID | Symbol | SW_rank | DL_rank | LS_rank | JTK_rank | Max-Min | Norm Plot |
|---|---|---|---|---|---|---|---|---|
| 1434468_at | MGI:1098801 | Otud4 | 3705 | 7089 | 5849.5 | 6394 | 4609.4 | |
| 1450705_at | MGI:102744 | Rdbp | 3707 | 5112 | 5147 | 5475 | 1624.4 | |
| 1435021_at | --- | | 3709 | 5529.5 | 5251 | 9316.5 | 9.1 | |
| 1433144_at | MGI:1914731 | Alg2 | 3714 | 9802.5 | 26754 | 10579.5 | 23.4 | |
| 1456996_at | --- | | 3715 | 37126.5 | 24396.5 | 10257.5 | 74.1 | |
| 1448500_a_at | MGI:1919949 | Lime1 | 3716.5 | 25583 | 28063.5 | 37728.5 | 75 | |
| 1415686_at | MGI:1915615 | Rab14 | 3718 | 7136 | 6968.5 | 6394 | 7571.7 | |
| 1423224_at | MGI:1915228 | Tctn2 | 3719 | 21459 | 28568 | 7632 | 38.9 | |
| 1450845_a_at | MGI:1914132 | Bzw1 | 3721 | 8422 | 10234.5 | 12382 | 11470.3 | |
| 1440269_at | MGI:1289172 | Scyl2 | 3722 | 9102.5 | 14763.5 | 7377 | 9.8 | |
| 1435223_at | MGI:2387215 | Erlin2 | 3724.5 | 6115 | 9750 | 7066 | 1263.7 | |
| 1415910_s_at | MGI:1922083 | Ciapin1 | 3730 | 18450 | 17128.5 | 29027 | 2120.4 | |
| 1418685_at | MGI:2152213 | Tirap | 3732.5 | 10331 | 15785.5 | 9198 | 1494 | |
| 1439364_a_at | --- | | 3732.5 | 33538 | 28882.5 | 37728.5 | 44.4 | |
| 1434378_a_at | MGI:104991 | Mxd4 | 3739 | 13699 | 10632 | 6168.5 | 2044 | |
| 1444780_at | --- | | 3742 | 4621 | 28479.5 | 29548.5 | 214.4 | |
| 1425561_at | MGI:1917297 | Trnt1 | 3744 | 14925 | 20343.5 | 5555.5 | 727.2 | |
| 1422792_at | MGI:108415 | Pafah1b2 | 3746.5 | 13744 | 16155 | 17046 | 1229.3 | |
| 1459919_a_at | MGI:2140623 | Mobkl2c | 3749 | 21288 | 20783.5 | 23158.5 | 322.8 | |
| 1437617_x_at | MGI:1920997 | 10034G24R | 3751 | 8842 | 19145.5 | 15195.5 | 376.6 | |
| 1415794_a_at | MGI:109242 | Spin1 | 3756 | 11632 | 13612.5 | 12382 | 9345.5 | |
| 1417437_at | MGI:95606 | Xrcc6 | 3759 | 8590 | 10698.5 | 8516.5 | 1131.9 | |
| 1455138_x_at | --- | | 3762.5 | 5436 | 5201.5 | 5475 | 17619.6 | |
| 1456534_at | MGI:104676 | Man1a2 | 3764 | 5529.5 | 11021.5 | 7463.5 | 6.7 | |
| 1459162_at | --- | | 3767.5 | 5529.5 | 6531.5 | 4814.5 | 18.2 | |
| 1435467_at | MGI:1261419 | Fgd6 | 3769 | 5183 | 9375 | 11529.5 | 1453.3 | |
| 1421828_at | MGI:1100863 | Kpna3 | 3770 | 8054 | 11101.5 | 25035 | 1493.7 | |

| Probe | MGI_ID | Symbol | SW_rank | DL_rank | LS_rank | JTK_rank | Max-Min | Norm Plot |
|---|---|---|---|---|---|---|---|---|
| 1448026_at | MGI:2444748 | Chd7 | 3773 | 13143 | 22228.5 | 9085 | 52.4 | 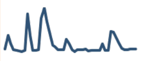 |
| 1442170_at | --- | | 3777 | 11176 | 18973.5 | 19869 | 22.1 | 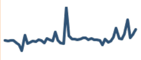 |
| 1428291_at | MGI:1916889 | Exosc8 | 3779 | 22984.5 | 21956.5 | 21202 | 1808.3 | 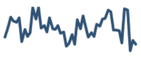 |
| 1429740_at | --- | | 3782 | 11521 | 24592.5 | 6990.5 | 15.4 | 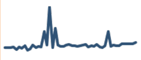 |
| 1417301_at | MGI:108474 | Fzd6 | 3784 | 8985 | 22037 | 22671.5 | 270.4 | 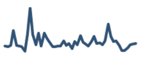 |
| 1454766_at | MGI:2442933 | Amn1 | 3787 | 7248 | 12769.5 | 10749.5 | 1956.9 | 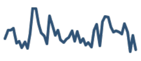 |
| 1430823_at | MGI:1924056 | 700029L08R | 3788 | 10805.5 | 20324 | 9929 | 16.7 | 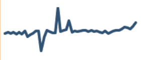 |
| 1459256_at | --- | | 3789 | 11176 | 28796 | 4947.5 | 21.3 | 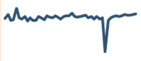 |
| 1428916_s_at | MGI:1915596 | Sirt5 | 3790 | 14680 | 17320 | 29027 | 686.7 | 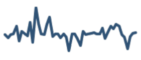 |
| 1425784_a_at | MGI:1860437 | Olfm1 | 3791 | 26065 | 25753.5 | 37728.5 | 111.7 | 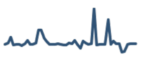 |
| 1429506_at | MGI:2135954 | Nkd1 | 3793 | 34520 | 27482.5 | 8123.5 | 25.4 | 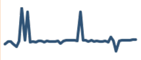 |
| 1460151_at | --- | | 3797 | 11652 | 26437.5 | 6718 | 661.3 | 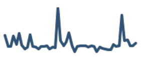 |
| 1435240_at | MGI:2442782 | Baz2b | 3800 | 5651 | 6003 | 7066 | 765.7 | 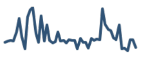 |
| 1417778_at | MGI:99179 | Zfp35 | 3803 | 7108 | 9314.5 | 7210.5 | 1359.3 | 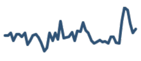 |
| 1440971_x_at | MGI:2442050 | Zfp771 | 3804.5 | 7955 | 10488.5 | 18744 | 251.6 | 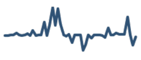 |
| 1422129_at | MGI:1346052 | Apc2 | 3804.5 | 39836.5 | 40494 | 37728.5 | 40.2 | 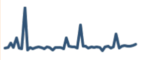 |
| 1424590_at | MGI:2148251 | Ddx19b | 3809 | 24039 | 25420.5 | 28658 | 355.2 | 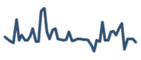 |
| 1438456_at | MGI:95886 | H13 | 3810 | 12354 | 19831 | 27698 | 710.2 | 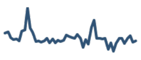 |
| 1417344_at | MGI:1920274 | 900064A13R | 3815 | 22545.5 | 19572 | 17315 | 7767.2 | 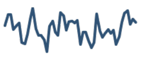 |
| 1455876_at | --- | | 3817 | 8041 | 8266 | 26491 | 220.3 | 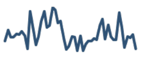 |
| 1433700_at | MGI:1914037 | 933433P14R | 3819 | 10313 | 13740 | 20671 | 2341 | 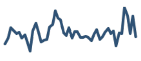 |
| 1450688_at | MGI:107483 | Rgl2 | 3821 | 6466 | 7803 | 9198 | 1211.7 | 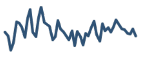 |
| 1453198_at | MGI:4834573 | Zfp955b | 3822 | 38313.5 | 23928 | 24106 | 581.4 | 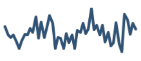 |
| 1443737_at | --- | | 3824.5 | 5401 | 22676.5 | 8836 | 40.9 | 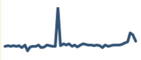 |
| 1443583_at | --- | | 3826 | 20721 | 22095 | 7066 | 141.4 | 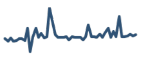 |
| 1435700_at | MGI:1917799 | Tln2 | 3828 | 35115 | 40494 | 29740 | 76.3 | 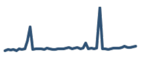 |
| 1434099_at | MGI:1342774 | Ppargc1a | 3830.5 | 6748 | 21440.5 | 25855 | 382.9 | 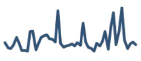 |

| Probe | MGI_ID | Symbol | SW_rank | DL_rank | LS_rank | JTK_rank | Max-Min | Norm Plot |
|---|---|---|---|---|---|---|---|---|
| 1441709_at | MGI:1345279 | Slc11a2 | 3830.5 | 7589 | 11993.5 | 7834 | 307.4 | 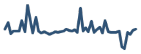 |
| 1420391_at | MGI:2135608 | Pard3 | 3834 | 32233.5 | 31724.5 | 37728.5 | 50.9 | 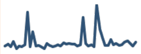 |
| 1453421_at | MGI:1351636 | Srr | 3836 | 10986 | 22187.5 | 17603 | 255.5 | 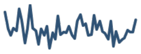 |
| 1457859_at | --- | | 3837 | 39966.5 | 23928 | 21202 | 24.1 | 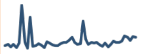 |
| 1428047_s_at | MGI:99211 | Zfx | 3838 | 8564 | 19252.5 | 6455.5 | 242.8 | 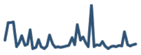 |
| 1417361_at | MGI:1929749 | Asb3 | 3840 | 12557 | 18236.5 | 10090 | 874.9 | 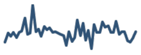 |
| 1417890_at | MGI:1919282 | Pdxp | 3841 | 15136 | 28084.5 | 6111.5 | 39.3 | 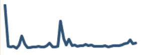 |
| 1437276_at | --- | | 3842 | 5115 | 7814.5 | 10419.5 | 610.6 | 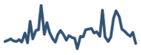 |
| 1457328_at | MGI:87936 | Adra2c | 3845 | 5300.5 | 16057.5 | 5185 | 33.4 | 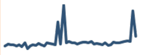 |
| 1456497_x_at | MGI:1914347 | Rps10 | 3846 | 23207.5 | 17667 | 10749.5 | 38618.6 | 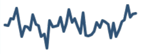 |
| 1460579_at | --- | | 3848 | 33497.5 | 34145 | 28255 | 15.9 | 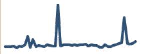 |
| 1439241_x_at | MGI:1930252 | Srd5a3 | 3850 | 18319 | 5397.5 | 12382 | 428.6 | 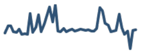 |
| 1425839_at | MGI:1913370 | Fkbp11 | 3851.5 | 5377 | 19357 | 6111.5 | 20.3 | 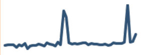 |
| 1447923_at | MGI:1916420 | 310026B05R | 3853 | 10272 | 10698.5 | 8123.5 | 2256.7 | 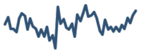 |
| 1423455_at | MGI:97803 | Ptma | 3854 | 7320 | 6085 | 5475 | 33623.4 | 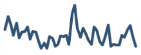 |
| 1448786_at | MGI:1914107 | Plbd1 | 3855 | 41302.5 | 22743.5 | 15710 | 3082.7 | 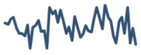 |
| 1427897_s_at | MGI:1919204 | Suds3 | 3856 | 5101 | 5914 | 4563.5 | 3766.3 | 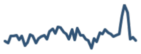 |
| 1460623_at | --- | | 3857.5 | 8152 | 6924 | 7544 | 1855 | 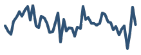 |
| 1423364_a_at | --- | | 3859 | 14968 | 18606 | 11946.5 | 970.8 | 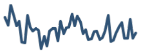 |
| 1446326_at | MGI:88468 | Col1a2 | 3861 | 44703 | 35392.5 | 37728.5 | 33.1 | 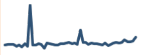 |
| 1434233_at | MGI:1914298 | Vma21 | 3863 | 25193.5 | 15362 | 9456 | 2646.3 | 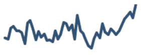 |
| 1455318_at | MGI:2445125 | Timd4 | 3865 | 29744.5 | 29915.5 | 25035 | 294 | 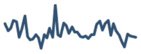 |
| 1417591_at | MGI:1917592 | Ptges2 | 3866 | 19430 | 7803 | 7544 | 1076.2 | 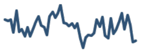 |
| 1430230_at | --- | | 3867 | 4806 | 4695.5 | 4777.5 | 74.5 | 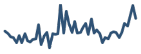 |
| 1444066_at | MGI:1913941 | Gapvd1 | 3869 | 25417 | 21482 | 11733 | 243.3 | 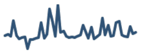 |
| 1429258_at | MGI:3696869 | 030001D20R | 3871 | 19933 | 21070 | 20130 | 16.6 | 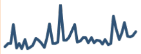 |
| 1424316_at | MGI:1914533 | Slc25a19 | 3874 | 23294 | 21019.5 | 14440.5 | 58.7 | 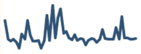 |

| Probe | MGI_ID | Symbol | SW_rank | DL_rank | LS_rank | JTK_rank | Max-Min | Norm Plot |
|---|---|---|---|---|---|---|---|---|
| 1426792_s_at | MGI:2140371 | Rusc2 | 3875 | 15367 | 20111.5 | 5745 | 226.9 | 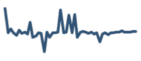 |
| 1426685_a_at | MGI:2144529 | Cnot6 | 3876 | 8033 | 8019.5 | 11946.5 | 2307.9 | 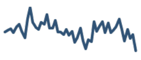 |
| 1426559_at | MGI:2384298 | Sbno1 | 3877 | 6403 | 11101.5 | 15710 | 6469.9 | 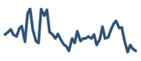 |
| 1452335_at | MGI:1919425 | Mfsd8 | 3878 | 22995 | 19086.5 | 16775 | 946.1 | 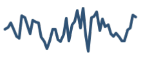 |
| 1428570_at | MGI:1858199 | Ccnc | 3879.5 | 7976 | 8230 | 5844.5 | 446.7 | 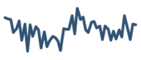 |
| 1418388_s_at | MGI:1922589 | Mphosph8 | 3879.5 | 8519 | 8497 | 9198 | 2586 | 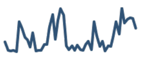 |
| 1449488_at | MGI:107374 | Pitx1 | 3886 | 7445.5 | 21387.5 | 7834 | 9.3 | 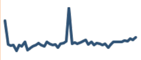 |
| 1423160_at | MGI:2150016 | Spred1 | 3887.5 | 4985.5 | 12769.5 | 14440.5 | 899.4 | 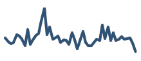 |
| 1422511_a_at | MGI:1919325 | Ogfr | 3887.5 | 21384 | 10287 | 14688 | 2165.7 | 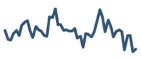 |
| 1445271_at | MGI:4821183 | Trim12c | 3889 | 10274 | 27988 | 37728.5 | 285.7 | 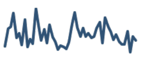 |
| 1426484_at | MGI:1915062 | Ubxn4 | 3892 | 13986 | 13194 | 8323 | 4460.5 | 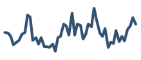 |
| 1447508_at | --- | | 3893 | 8149 | 20799 | 6394 | 62.1 | 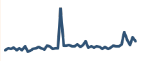 |
| 1457465_at | MGI:2685570 | Shroom4 | 3894 | 31441.5 | 31813.5 | 29740 | 20.2 | 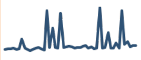 |
| 1448784_at | MGI:1346320 | Taf10 | 3897 | 15149 | 15015 | 15710 | 13586.7 | 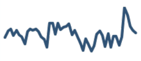 |
| 1434671_at | MGI:2138157 | 230337E12R | 3899 | 5862 | 7582.5 | 12382 | 963.6 | 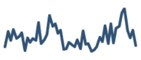 |
| 1419471_a_at | MGI:106014 | Nudc | 3904 | 7765.5 | 6388 | 4516 | 3250.3 | 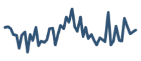 |
| 1457268_at | MGI:2143886 | Dot1l | 3907 | 21455 | 25720.5 | 14688 | 656.5 | 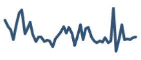 |
| 1423482_at | MGI:98917 | Uros | 3910 | 4779 | 6849 | 5068.5 | 2204.4 | 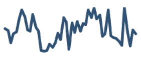 |
| 1420981_a_at | MGI:109360 | Lmo4 | 3914 | 27991.5 | 30117 | 37728.5 | 1064.7 | 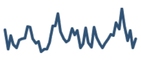 |
| 1450706_a_at | MGI:1929699 | Arl3 | 3915 | 13426 | 8758.5 | 7377 | 2462.5 | 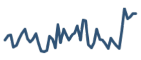 |
| 1456773_at | MGI:2387631 | Nupl2 | 3916 | 27453.5 | 28737 | 37728.5 | 156.4 | 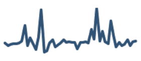 |
| 1415868_at | MGI:104689 | Cct4 | 3920 | 7005 | 14607 | 25855 | 274.2 | 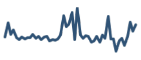 |
| 1433665_at | MGI:1929215 | Vps41 | 3922 | 16554 | 17553.5 | 6785.5 | 477.3 | 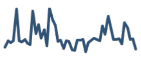 |
| 1449063_at | MGI:1338759 | Sec22b | 3923 | 6007 | 6453.5 | 5068.5 | 8834.2 | 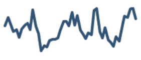 |
| 1433173_at | MGI:1918634 | 430440L12R | 3924 | 29326.5 | 30417 | 37728.5 | 173.2 | 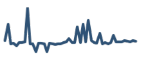 |
| 1426207_at | MGI:1338071 | Ikbkb | 3925 | 5652 | 9888.5 | 12382 | 6482 | 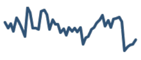 |
| 1424395_at | MGI:1913764 | Asrgl1 | 3926 | 8957 | 13194 | 7544 | 134.2 | 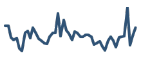 |

| Probe | MGI_ID | Symbol | SW_rank | DL_rank | LS_rank | JTK_rank | Max-Min | Norm Plot |
|---|---|---|---|---|---|---|---|---|
| 1438764_at | MGI:88031 | Anxa7 | 3927 | 5316 | 7284.5 | 5645.5 | 952 | 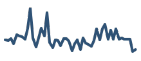 |
| 1446626_at | MGI:101825 | 16H22S680 | 3932 | 26997.5 | 32549.5 | 37728.5 | 104 | 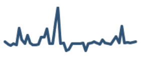 |
| 1452611_at | MGI:1926163 | Ltn1 | 3938 | 24632 | 25155 | 21202 | 362.8 | 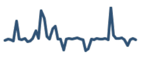 |
| 1453156_s_at | MGI:1916372 | Ptgr2 | 3939.5 | 26048.5 | 6566 | 5942 | 2395.7 | 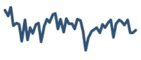 |
| 1432680_at | MGI:1918838 | 30009M17F | 3941 | 35025 | 40494 | 13264 | 92.2 | 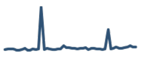 |
| 1424611_x_at | MGI:2442186 | Trub2 | 3942 | 12069 | 13284 | 21202 | 2042.6 | 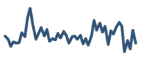 |
| 1441033_at | MGI:1914057 | Tmtc2 | 3944 | 16769 | 20277.5 | 19869 | 105 | 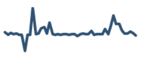 |
| 1430216_at | MGI:1353423 | Zfp292 | 3948 | 5611 | 19679 | 25466.5 | 463.5 | 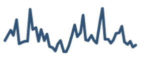 |
| 1435646_at | MGI:1338074 | Ikbkg | 3949 | 14899 | 11179.5 | 19033.5 | 2237.8 | 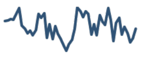 |
| 1451703_s_at | MGI:88061 | Aprt | 3951 | 7181 | 5243.5 | 5475 | 4217.5 | 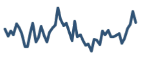 |
| 1459339_at | --- | | 3952 | 35217.5 | 34611 | 37728.5 | 28.1 | 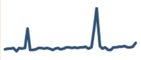 |
| 1428803_at | MGI:1921287 | Acot6 | 3953.5 | 10143.5 | 24194 | 17315 | 13.3 | 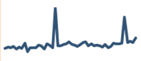 |
| 1418842_at | MGI:104568 | Hcls1 | 3956 | 26873 | 26754 | 23641.5 | 34.4 | 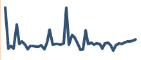 |
| 1458597_at | --- | | 3957 | 14959 | 13194 | 10419.5 | 81.3 | 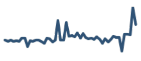 |
| 1428682_at | MGI:1926001 | Zc3h6 | 3958 | 10672 | 20550.5 | 37728.5 | 257 | 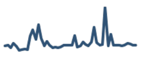 |
| 1452447_at | MGI:1923501 | 510007P08R | 3960 | 19394 | 27364.5 | 12382 | 53 | 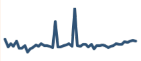 |
| 1425765_at | --- | | 3963 | 21921.5 | 20533 | 16234 | 100.9 | 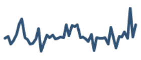 |
| 1417315_at | MGI:1859616 | Gripap1 | 3968 | 17087 | 21097 | 37728.5 | 1578.9 | 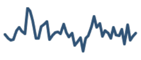 |
| 1460614_at | MGI:2442317 | Mier3 | 3971 | 10264 | 14651 | 9771 | 1554.7 | 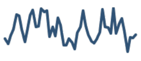 |
| 1451049_at | MGI:1350933 | Bcap31 | 3972 | 6797 | 5368.5 | 5942 | 9831.9 | 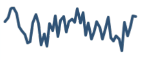 |
| 1426262_at | MGI:2448562 | Adnp2 | 3973 | 26226 | 26955 | 37728.5 | 123.8 | 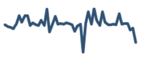 |
| 1449576_at | MGI:1913485 | Eif1ax | 3974 | 5089 | 4670 | 4650 | 1658.3 | 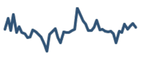 |
| 1430820_a_at | MGI:1917758 | Bbx | 3975.5 | 8028 | 13691 | 8516.5 | 1485.5 | 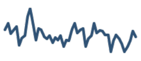 |
| 1425988_a_at | MGI:1314873 | Hipk1 | 3977.5 | 8579 | 18649 | 23873.5 | 265.6 | 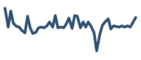 |
| 1415896_x_at | MGI:98347 | Snrpn | 3982 | 25871.5 | 26111 | 22916 | 972.4 | 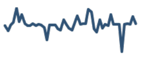 |
| 1452753_at | MGI:1916087 | Foxk2 | 3983.5 | 13161 | 13525 | 6050.5 | 786.4 | 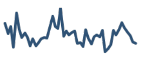 |
| 1426290_at | MGI:1913504 | Dimt1 | 3983.5 | 15124 | 18214 | 12823.5 | 497.2 | 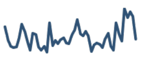 |

| Probe | MGI_ID | Symbol | SW_rank | DL_rank | LS_rank | JTK_rank | Max-Min | Norm Plot |
|---|---|---|---|---|---|---|---|---|
| 1450220_a_at | MGI:1353422 | Spdef | 3988 | 6335 | 11947 | 9085 | 16.9 | |
| 1417583_a_at | MGI:1919230 | Utp3 | 3990 | 7056 | 9113 | 10419.5 | 4582.2 | |
| 1442651_at | --- | | 3992 | 6147 | 25969 | 26949.5 | 300.9 | |
| 1422553_at | MGI:109583 | Pten | 3995 | 8514 | 12427 | 10090 | 16853.9 | |
| 1416751_a_at | MGI:1858415 | Ddx20 | 3995 | 11957 | 13525 | 13728 | 615.5 | |
| 1419648_at | MGI:106612 | Myo1c | 3995 | 24602 | 19765.5 | 15195.5 | 950.3 | |
| 1423572_at | MGI:108052 | Bcl2l2 | 3997.5 | 6443 | 6048 | 8516.5 | 945.6 | |
| 1428307_at | MGI:1919227 | Zdhhc13 | 3997.5 | 12322 | 12427 | 7066 | 933.6 | |
| 1438997_at | --- | | 3999.5 | 7760 | 28987 | 37728.5 | 133.8 | |
| 1420386_at | MGI:1919374 | Seh1l | 4003 | 8147 | 21943 | 9198 | 210.2 | |
| 1426858_at | MGI:96571 | Inhbb | 4004 | 17912.5 | 24896 | 18744 | 17 | |
| 1456288_at | MGI:1329004 | Slfn5 | 4006 | 20708 | 33162 | 37728.5 | 425.9 | |
| 1423040_at | MGI:1914132 | Bzw1 | 4007.5 | 5800 | 6704 | 5844.5 | 22281.5 | |
| 1422962_a_at | MGI:1346527 | Psmb8 | 4010 | 15153 | 17578 | 13728 | 3191.5 | |
| 1438747_at | --- | | 4011.5 | 32756.5 | 32434.5 | 37728.5 | 89.2 | |
| 1452893_s_at | MGI:1916888 | Enho | 4013 | 8826 | 19388.5 | 17907.5 | 978.7 | |
| 1455945_at | MGI:99205 | Zfp58 | 4015 | 12373 | 18405 | 20951.5 | 391 | |
| 1450819_at | MGI:1926483 | Pvrl1 | 4018 | 39713 | 35392.5 | 9609.5 | 48.7 | |
| 1438122_at | MGI:1920123 | [illegible]00006K08R | 4019.5 | 6820 | 17642.5 | 4814.5 | 65.6 | |
| 1446963_at | --- | | 4025 | 37344 | 35392.5 | 27146 | 34.3 | |
| 1416569_at | MGI:1861453 | Actl6a | 4027 | 41891.5 | 28479.5 | 37728.5 | 1761.8 | |
| 1424869_at | MGI:2384931 | Dhrs7b | 4030 | 6442 | 8542.5 | 6168.5 | 2787 | |
| 1456875_at | --- | | 4031 | 5926.5 | 6876.5 | 10419.5 | 732.9 | |
| 1418234_s_at | MGI:1915433 | Bcas2 | 4032 | 38289 | 15724 | 7632 | 2045.7 | |
| 1449889_a_at | MGI:1915345 | Ociad1 | 4035.5 | 5220 | 4897.5 | 8727 | 9265.6 | |
| 1427231_at | --- | | 4037 | 5049 | 16187 | 11529.5 | 1072.7 | |
| 1456249_x_at | MGI:98467 | Syp | 4038 | 32959.5 | 35392.5 | 23641.5 | 42.1 | |

| Probe | MGI_ID | Symbol | SW_rank | DL_rank | LS_rank | JTK_rank | Max-Min | Norm Plot |
|---|---|---|---|---|---|---|---|---|
| 1426946_at | MGI:1917822 | Ipo5 | 4046 | 13128 | 11493.5 | 10090 | 1442.3 | |
| 1425670_at | MGI:1333865 | Rfxank | 4047 | 14269 | 18236.5 | 11733 | 55.4 | |
| 1416691_at | MGI:1860138 | Gtpbp2 | 4048 | 9299 | 17905 | 20951.5 | 144.5 | |
| 1441365_at | MGI:2148705 | Foxp2 | 4049.5 | 7095 | 15724 | 5993.5 | 145.7 | |
| 1422869_at | MGI:96965 | Mertk | 4052 | 13970 | 18801.5 | 27880 | 1995.5 | |
| 1428861_at | MGI:1925999 | Filip1l | 4054.5 | 15574 | 22951.5 | 37728.5 | 497.3 | |
| 1453054_at | MGI:1349480 | Scamp1 | 4058 | 9259 | 5654.5 | 5309 | 2756 | |
| 1437658_a_at | --- | | 4059 | 8596 | 27081 | 23158.5 | 562.4 | |
| 1416254_a_at | MGI:2136772 | Vps16 | 4062 | 9628 | 5008.5 | 6111.5 | 1230.4 | |
| 1423649_at | MGI:1919348 | Tmem68 | 4063.5 | 8581 | 14353.5 | 14939.5 | 1841.2 | |
| 1415715_at | MGI:1915616 | Tmem129 | 4066 | 5771 | 12489 | 8516.5 | 2795.2 | |
| 1419688_at | MGI:1346322 | Gpc6 | 4068 | 13027 | 20932 | 19033.5 | 6.7 | |
| 1429707_at | MGI:104810 | Plaa | 4071 | 12563 | 13789.5 | 8123.5 | 1934.6 | |
| 1433873_s_at | MGI:102722 | Pcnt | 4072 | 8156 | 10947.5 | 16503.5 | 370.3 | |
| 1455675_a_at | --- | | 4074 | 16749 | 17614 | 17907.5 | 2453.7 | |
| 1423006_at | MGI:97584 | Pim1 | 4075 | 24644.5 | 29227.5 | 37728.5 | 187.7 | |
| 1419941_at | MGI:1924635 | 030018P15R | 4077 | 33058.5 | 40494 | 27880 | 43.7 | |
| 1423370_a_at | MGI:1920014 | Csnk1g2 | 4079 | 5272 | 9627 | 10749.5 | 2770.6 | |
| 1423789_at | MGI:2385132 | BC005624 | 4080 | 8414 | 7652.5 | 5942 | 1527.9 | |
| 1455781_at | MGI:2384836 | BC027231 | 4092 | 21608 | 22474 | 23873.5 | 234.4 | |
| 1449098_a_at | MGI:1347081 | Poli | 4093 | 18347 | 15845 | 6455.5 | 388.8 | |
| 1437163_x_at | MGI:1338799 | Gtf2h4 | 4094 | 6639 | 4545 | 6915.5 | 2135.9 | |
| 1423776_s_at | MGI:1289265 | Tbc1d22a | 4097 | 6175 | 8919.5 | 16234 | 1572.8 | |
| 1447597_at | MGI:1096397 | Polr1a | 4099 | 8952 | 24750 | 5597 | 100.8 | |
| 1427195_at | MGI:892977 | Slc26a2 | 4100 | 26038.5 | 30975 | 37728.5 | 98.2 | |
| 1435295_at | MGI:97566 | Pgm3 | 4101 | 9276 | 14147.5 | 8619 | 550.1 | |
| 1429046_at | MGI:1913563 | Smurf2 | 4104 | 5831 | 4684 | 6050.5 | 1003.8 | |

| Probe | MGI_ID | Symbol | SW_rank | DL_rank | LS_rank | JTK_rank | Max-Min | Norm Plot |
|---|---|---|---|---|---|---|---|---|
| 1422227_at | MGI:1333796 | Klf12 | 4108 | 15823.5 | 18341 | 20130 | 36.6 | 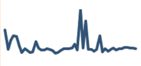 |
| 1427542_at | MGI:1924427 | Sobp | 4109 | 39536 | 31390.5 | 23400.5 | 13.4 | 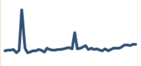 |
| 1442098_at | MGI:2146564 | AU022434 | 4110 | 5198 | 7770 | 7925.5 | 1320 | 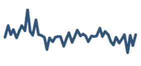 |
| 1447809_x_at | MGI:2444666 | Zfp420 | 4111.5 | 24229 | 31176.5 | 37728.5 | 22 | 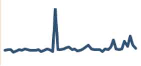 |
| 1442397_at | MGI:1921414 | Nfx1 | 4113.5 | 10637 | 14442 | 20389.5 | 94.3 | 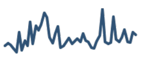 |
| 1417935_at | MGI:1914277 | Mkrn2 | 4113.5 | 23301.5 | 10411.5 | 5068.5 | 963 | 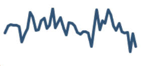 |
| 1440792_x_at | MGI:1195268 | Sigmar1 | 4115.5 | 6813 | 7128.5 | 6050.5 | 27.9 | 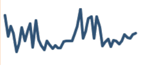 |
| 1428766_at | MGI:1914640 | Rnmtl1 | 4115.5 | 11299 | 13284 | 12823.5 | 868.5 | 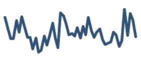 |
| 1422476_at | MGI:2137648 | Ifi30 | 4118 | 10016 | 10947.5 | 12823.5 | 3352.9 | 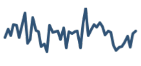 |
| 1421153_at | MGI:1914823 | Loxl4 | 4119.5 | 9926 | 24592.5 | 8619 | 113.8 | 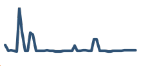 |
| 1450450_at | MGI:1858220 | Rcan3 | 4121 | 20453.5 | 21598 | 29191.5 | 17.9 | 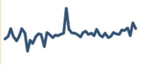 |
| 1454945_at | MGI:1921451 | Cep97 | 4123 | 5144 | 8193.5 | 5597 | 523.2 | 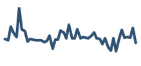 |
| 1438242_at | MGI:2443184 | Usp40 | 4125 | 19788 | 20499.5 | 6526 | 497 | 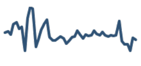 |
| 1416323_at | MGI:1914239 | Kctd20 | 4126 | 5184 | 11101.5 | 11529.5 | 1073.9 | 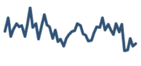 |
| 1415884_at | MGI:1915118 | Cela3b | 4127 | 7622 | 40494 | 37728.5 | 8781.8 | 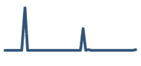 |
| 1437810_a_at | MGI:96024 | Hbb-bh1 | 4129 | 14462 | 24160 | 26060 | 260.3 | 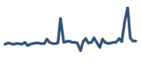 |
| 1436826_at | --- |  | 4130.5 | 8013 | 6666.5 | 8958.5 | 397.7 | 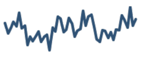 |
| 1418244_at | MGI:1915127 | Naa20 | 4135 | 7261 | 12971.5 | 26273.5 | 5284.8 | 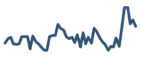 |
| 1418692_at | MGI:96960 | Rab8a | 4143 | 7586 | 5732.5 | 6282.5 | 4137.9 | 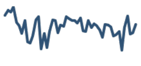 |
| 1447655_x_at | --- |  | 4146 | 13434 | 12427 | 9771 | 552.7 | 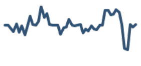 |
| 1454703_x_at | --- |  | 4148 | 6211 | 10044.5 | 4814.5 | 574.5 | 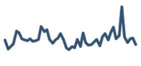 |
| 1424681_a_at | MGI:1347009 | Psma5 | 4150.5 | 12894 | 14194 | 7734 | 9404.5 | 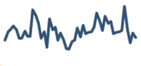 |
| 1454322_at | MGI:1921654 | [illegible]33404M09F | 4159 | 21959 | 26075.5 | 4516 | 29.2 | 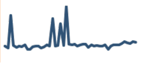 |
| 1444924_at | --- |  | 4160 | 27628.5 | 19658.5 | 12163.5 | 111.7 | 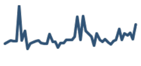 |
| 1428835_at | MGI:1919210 | Myh14 | 4165 | 8520 | 22187.5 | 7463.5 | 934.3 | 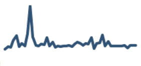 |
| 1440734_at | MGI:2155779 | Ttbk2 | 4167 | 33548 | 33352 | 37728.5 | 55.6 | 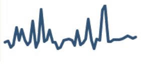 |
| 1450561_a_at | MGI:98443 | Surf1 | 4170 | 11713 | 7626 | 5696 | 4459.2 | 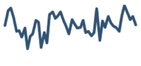 |

| Probe | MGI_ID | Symbol | SW_rank | DL_rank | LS_rank | JTK_rank | Max-Min | Norm Plot |
|---|---|---|---|---|---|---|---|---|
| 1429486_at | MGI:107815 | Pfkfb2 | 4178 | 13958 | 26549.5 | 12382 | 107.1 | 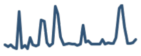 |
| 1433234_at | MGI:1921114 | ?30424E08R | 4181 | 9802.5 | 25882.5 | 9316.5 | 27 | 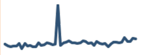 |
| 1417244_a_at | MGI:1859212 | Irf7 | 4183 | 18082 | 21849.5 | 28255 | 854.1 | 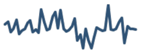 |
| 1428617_at | MGI:1915183 | Hcfc2 | 4184 | 8989 | 9166.5 | 19033.5 | 1595.5 | 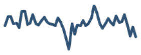 |
| 1454317_at | MGI:2429506 | Adam1b | 4186 | 5421 | 25452.5 | 5389 | 23 | 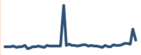 |
| 1448743_at | MGI:2139150 | Ssx2ip | 4187 | 15392 | 19063.5 | 29364.5 | 986.8 | 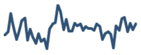 |
| 1429103_at | MGI:2450248 | Tomm22 | 4188 | 4999.5 | 5747.5 | 6785.5 | 4625.9 | 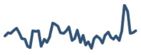 |
| 1419766_at | MGI:104754 | Sik1 | 4190.5 | 28417.5 | 30074 | 13264 | 91.5 | 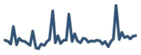 |
| 1451599_at | MGI:2651874 | Sesn2 | 4193 | 32747.5 | 32815 | 37728.5 | 323.6 | 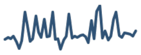 |
| 1437023_at | --- | | 4194 | 25518.5 | 28063.5 | 37728.5 | 60.6 | 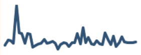 |
| 1444408_at | MGI:2445139 | 30040O20R | 4196 | 11636 | 20039.5 | 18465.5 | 135.9 | 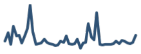 |
| 1424253_at | MGI:1917629 | Fam114a2 | 4200 | 5126 | 5024 | 4947.5 | 2074.9 | 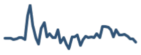 |
| 1448488_at | MGI:1924971 | Mrps5 | 4201 | 25026 | 23354.5 | 21692.5 | 1392.5 | 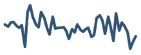 |
| 1454986_at | MGI:2442943 | Zfp668 | 4202 | 24621.5 | 19188 | 10749.5 | 34.8 | 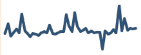 |
| 1424424_at | MGI:1353474 | Slc39a1 | 4203 | 9651 | 4943.5 | 5475 | 2606.2 | 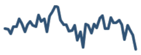 |
| 1451737_at | MGI:97583 | Pik3r1 | 4208.5 | 6413 | 11568.5 | 11529.5 | 1022 | 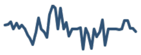 |
| 1437206_at | MGI:1920145 | Setd5 | 4210 | 7676 | 20398 | 18465.5 | 464 | 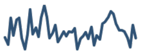 |
| 1453726_s_at | MGI:1916477 | 310407C02R | 4211 | 8143 | 10823.5 | 11946.5 | 16234.1 | 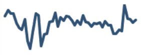 |
| 1419670_at | MGI:1339962 | Ftcd | 4213 | 7105 | 6540 | 6526 | 40668.1 | 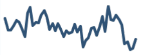 |
| 1427916_at | MGI:2386964 | St7l | 4214 | 7572 | 6219.5 | 7544 | 1561.3 | 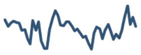 |
| 1450943_at | MGI:1913691 | Magohb | 4215 | 8031 | 21387.5 | 17046 | 373.7 | 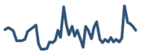 |
| 1422630_at | MGI:109292 | Rad50 | 4216 | 9210 | 16437 | 10931 | 423.9 | 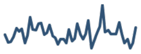 |
| 1453040_at | MGI:1914312 | Mcart6 | 4217 | 27588 | 25185.5 | 37728.5 | 85.7 | 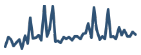 |
| 1425582_a_at | MGI:1891716 | Emcn | 4220.5 | 6448 | 15785.5 | 23641.5 | 748.4 | 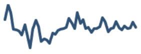 |
| 1449083_at | MGI:1914265 | Ccdc91 | 4223 | 7014 | 9177 | 15195.5 | 2185.7 | 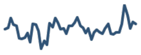 |
| 1416856_at | MGI:1913930 | 230401D17R | 4224 | 19108 | 14101.5 | 12382 | 2847.3 | 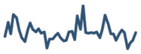 |
| 1431753_x_at | MGI:1915455 | Urm1 | 4227 | 26226 | 25720.5 | 37728.5 | 284.2 | 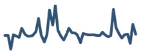 |

| Probe | MGI_ID | Symbol | SW_rank | DL_rank | LS_rank | JTK_rank | Max-Min | Norm Plot |
|---|---|---|---|---|---|---|---|---|
| 1435442_at | MGI:2684929 | Dcaf13 | 4232 | 4527 | 5323.5 | 4563.5 | 8151.8 | |
| 1418719_at | MGI:1923728 | Haus8 | 4234 | 21181 | 15057.5 | 8619 | 121.6 | |
| 1455955_s_at | --- | | 4236 | 7634 | 14907.5 | 20671 | 3140.8 | |
| 1446607_at | --- | | 4237 | 28205 | 24966.5 | 9198 | 23.8 | |
| 1452941_at | MGI:1913322 | Sdhaf2 | 4238.5 | 16023 | 12489 | 20130 | 1202.1 | |
| 1452233_at | MGI:102676 | Abcc1 | 4242 | 18909 | 19733 | 37728.5 | 56.2 | |
| 1441428_at | --- | | 4246 | 25098.5 | 27755.5 | 29364.5 | 140.7 | |
| 1439050_at | MGI:104995 | Gclm | 4247 | 16391 | 4670 | 4650 | 11023 | |
| 1439818_at | --- | | 4250 | 7817.5 | 14009 | 10090 | 29.1 | |
| 1451347_at | MGI:109446 | Doc2a | 4254 | 16168 | 21424 | 8619 | 40.6 | |
| 1427030_at | MGI:1196252 | Spice1 | 4257 | 30776 | 40494 | 37728.5 | 216.7 | |
| 1434992_at | MGI:2143792 | Zfr2 | 4259.5 | 11176 | 12872 | 7734 | 21.2 | |
| 1416737_at | MGI:101805 | Gys1 | 4259.5 | 19228.5 | 9269 | 6990.5 | 99.2 | |
| 1424983_a_at | MGI:1926082 | 700078E11R | 4261.5 | 12360 | 19968 | 12382 | 1660.5 | |
| 1422082_a_at | MGI:97316 | Nfya | 4263 | 6221 | 27482.5 | 5895.5 | 35.6 | |
| 1422581_at | MGI:1913125 | Pias1 | 4264.5 | 4967 | 7731.5 | 9198 | 1631.6 | |
| 1416012_at | MGI:1341878 | Ehd1 | 4266 | 12081 | 33352 | 7293.5 | 38.4 | |
| 1451292_at | MGI:2682609 | Zfp212 | 4267 | 7599 | 15130.5 | 21202 | 605.3 | |
| 1448542_at | MGI:1913415 | Bccip | 4269 | 9997 | 12319.5 | 6168.5 | 10992.6 | |
| 1448648_at | MGI:1915553 | Fam114a1 | 4272 | 9671 | 14527 | 17315 | 1430.8 | |
| 1436801_x_at | MGI:1929760 | Cdc42ep4 | 4274 | 9235 | 12971.5 | 5519 | 1966.4 | |
| 1450755_at | MGI:108415 | Pafah1b2 | 4275 | 21246 | 15285.5 | 16234 | 1265 | |
| 1436466_at | --- | | 4276 | 27716 | 24855.5 | 22671.5 | 146.5 | |
| 1424915_s_at | MGI:1919185 | 10044G17R | 4280 | 9642 | 13143.5 | 9771 | 5349.5 | |
| 1436688_x_at | MGI:1914365 | Rpl14 | 4280 | 25239 | 14056.5 | 16234 | 33987.2 | |
| 1438359_at | MGI:1922856 | 10003K15R | 4286 | 5977 | 6628.5 | 8516.5 | 8718.6 | |
| 1443444_at | --- | | 4287 | 15698 | 20900 | 8123.5 | 11.8 | |

| Probe | MGI_ID | Symbol | SW_rank | DL_rank | LS_rank | JTK_rank | Max-Min | Norm Plot |
|---|---|---|---|---|---|---|---|---|
| 1434149_at | MGI:98506 | Tcf4 | 4289 | 11326 | 18681 | 25466.5 | 549.4 | |
| 1439713_at | --- | | 4292.5 | 7334 | 14763.5 | 11946.5 | 335.6 | |
| 1423219_a_at | MGI:108180 | Mrpl49 | 4292.5 | 10959 | 9022 | 7925.5 | 3259.2 | |
| 1428642_at | MGI:1923407 | Slc35d3 | 4294 | 5421 | 6656 | 6455.5 | 28.5 | |
| 1443155_at | --- | | 4295 | 5529.5 | 7563 | 6718 | 13.5 | |
| 1426285_at | MGI:99912 | Lama2 | 4297 | 34494 | 35392.5 | 37728.5 | 30 | |
| 1423688_at | MGI:1920994 | Man2c1 | 4302 | 7663 | 11762 | 10419.5 | 720.3 | |
| 1446223_at | MGI:3649419 | Gm12289 | 4303 | 26253 | 40494 | 37728.5 | 123.9 | |
| 1431283_at | MGI:1923077 | 30542N06R | 4304 | 44923.5 | 40494 | 37728.5 | 13.3 | |
| 1434392_at | --- | | 4308 | 6024.5 | 7518 | 5793.5 | 2352.4 | |
| 1438031_at | MGI:3028579 | Rasgrp3 | 4309 | 21101 | 13789.5 | 10931 | 65.8 | |
| 1460467_at | MGI:1916957 | Iqcg | 4312 | 6259.5 | 19417 | 9316.5 | 57.5 | |
| 1428002_at | MGI:1926185 | Saal1 | 4313.5 | 5529.5 | 16997 | 4563.5 | 29.6 | |
| 1423421_at | MGI:1930842 | Ankrd49 | 4315 | 13722 | 13238.5 | 7210.5 | 1309 | |
| 1423120_at | MGI:96412 | Ide | 4316 | 13982 | 20301 | 22186.5 | 14608.6 | |
| 1436357_at | --- | | 4318.5 | 5629.5 | 17721.5 | 16234 | 1031.6 | |
| 1438956_x_at | --- | | 4318.5 | 6558 | 21986.5 | 6168.5 | 461.1 | |
| 1448603_at | MGI:1201408 | Srpk2 | 4320 | 8407 | 8837.5 | 16503.5 | 204.3 | |
| 1435237_at | --- | | 4323 | 19198 | 24577 | 26273.5 | 5314.3 | |
| 1437393_at | MGI:97595 | Prkca | 4326 | 17702 | 22891.5 | 20951.5 | 136.8 | |
| 1429489_at | MGI:1914182 | Rexo1 | 4332 | 18106 | 20162 | 27146 | 730.8 | |
| 1445358_at | MGI:1341087 | Tnks | 4334 | 14279 | 24345 | 6111.5 | 33.5 | |
| 1447109_at | --- | | 4336 | 8831 | 26768.5 | 37728.5 | 1055.9 | |
| 1432274_at | MGI:1922437 | 30543N07R | 4338 | 5529.5 | 12257.5 | 11733 | 8.2 | |
| 1416657_at | MGI:87986 | Akt1 | 4339 | 11666 | 7814.5 | 6394 | 1397 | |
| 1427276_at | MGI:1917349 | Smc4 | 4340 | 8832 | 19616.5 | 11529.5 | 646.3 | |
| 1423162_s_at | MGI:2150016 | Spred1 | 4341 | 8101 | 17205.5 | 30286 | 195.2 | |

| Probe | MGI_ID | Symbol | SW_rank | DL_rank | LS_rank | JTK_rank | Max-Min | Norm Plot |
|---|---|---|---|---|---|---|---|---|
| 1421641_at | MGI:1270850 | Slc6a2 | 4343 | 28052.5 | 28916.5 | 22671.5 | 42.1 | 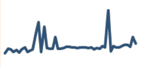 |
| 1455517_at | MGI:1100865 | Rbm4 | 4344 | 29917.5 | 20025.5 | 37728.5 | 35.1 | 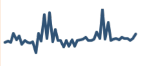 |
| 1427090_at | MGI:2682302 | Zbed4 | 4351 | 7060 | 15753 | 9456 | 354.7 | 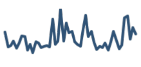 |
| 1457189_at | --- | | 4353 | 11748 | 26768.5 | 37728.5 | 213 | 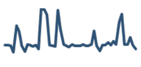 |
| 1435948_at | --- | | 4356 | 9958 | 10025 | 17315 | 297.4 | 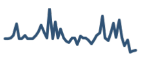 |
| 1435174_at | MGI:3698050 | 130049A11F | 4357 | 14245 | 23200 | 23641.5 | 1556 | 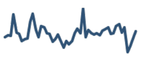 |
| 1436328_at | MGI:1915147 | Rnmt | 4359 | 23575 | 25566 | 37728.5 | 513.9 | 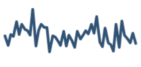 |
| 1460519_a_at | MGI:1922672 | Mettl5 | 4362 | 8085 | 5344.5 | 5475 | 7520.2 | 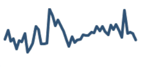 |
| 1424639_a_at | MGI:96158 | Hmgcl | 4363 | 30459 | 5182.5 | 4563.5 | 28905.4 | 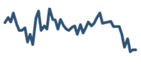 |
| 1436558_at | MGI:1922566 | Taf1d | 4364 | 29155 | 30034 | 37728.5 | 142.1 | 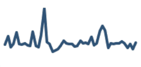 |
| 1452692_a_at | MGI:1920150 | Ndufv2 | 4365 | 10287 | 11419.5 | 7925.5 | 41794.1 | 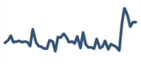 |
| 1427172_at | MGI:1350328 | Ofd1 | 4368 | 5821 | 10947.5 | 8836 | 222.7 | 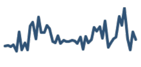 |
| 1416450_at | MGI:1926879 | Taf8 | 4369 | 25206.5 | 25882.5 | 37728.5 | 106 | 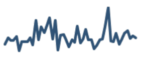 |
| 1420688_a_at | MGI:1329042 | Sgce | 4371 | 28334 | 29520.5 | 37728.5 | 1273.2 | 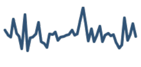 |
| 1417779_at | MGI:1913816 | Ntpcr | 4372.5 | 9618 | 11762 | 5475 | 344.3 | 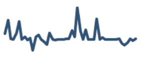 |
| 1441376_at | MGI:1890602 | Gabarapl2 | 4372.5 | 18882 | 19863.5 | 25253.5 | 515.8 | 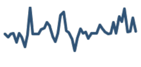 |
| 1426965_at | MGI:97855 | Rap2a | 4374 | 15569 | 11762 | 6050.5 | 1585.2 | 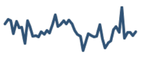 |
| 1421329_a_at | MGI:104790 | Smyd1 | 4375 | 5824 | 21019.5 | 5555.5 | 548.8 | 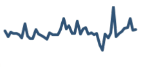 |
| 1459655_at | --- | | 4377 | 28801 | 26480 | 37728.5 | 70.1 | 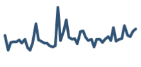 |
| 1447403_a_at | --- | | 4380 | 8986.5 | 15632 | 5112 | 88.1 | 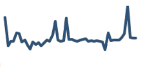 |
| 1447628_x_at | MGI:1924971 | Mrps5 | 4382 | 14253 | 20111.5 | 24106 | 49.7 | 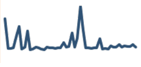 |
| 1422697_s_at | MGI:104813 | Jarid2 | 4385 | 6771 | 16352 | 27146 | 768.8 | 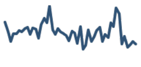 |
| 1433867_at | MGI:1916405 | 10030O07F | 4387 | 24707.5 | 25060.5 | 37728.5 | 505.7 | 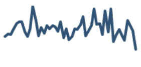 |
| 1418200_at | MGI:2140248 | Zbtb48 | 4388 | 13751 | 18586.5 | 8958.5 | 294.7 | 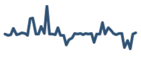 |
| 1434744_at | MGI:2387201 | Yrdc | 4391 | 9582 | 11101.5 | 28255 | 1328.9 | 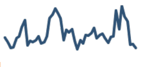 |
| 1445259_at | --- | | 4394.5 | 6335 | 7366 | 8420 | 8.6 | 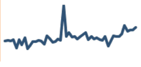 |
| 1451299_at | MGI:1309999 | Prkx | 4396 | 23903.5 | 24827.5 | 29364.5 | 522.5 | 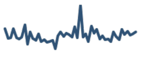 |

| Probe | MGI_ID | Symbol | SW_rank | DL_rank | LS_rank | JTK_rank | Max-Min | Norm Plot |
|---|---|---|---|---|---|---|---|---|
| 1438227_at | MGI:1099462 | She | 4397 | 17270 | 23850.5 | 21448 | 37.1 | 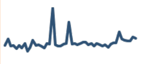 |
| 1427324_at | MGI:3603158 | Tmem120b | 4401 | 24160 | 30287 | 5309 | 155.8 | 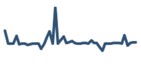 |
| 1453212_at | --- | | 4402 | 8916 | 16089 | 18744 | 523 | 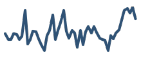 |
| 1452042_a_at | MGI:1917902 | Tmem144 | 4403.5 | 31850.5 | 34145 | 37728.5 | 45.1 | 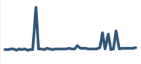 |
| 1460416_s_at | MGI:1891441 | Csprs | 4403.5 | 41792 | 40494 | 37728.5 | 36.6 | 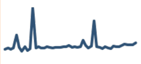 |
| 1449661_at | MGI:1261758 | Suz12 | 4406 | 21010 | 28334 | 37728.5 | 153.6 | 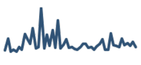 |
| 1430370_at | MGI:97838 | Eprs | 4408 | 9943 | 19877.5 | 37728.5 | 225.3 | 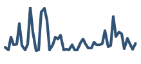 |
| 1417407_at | MGI:2141676 | Fbxl14 | 4409.5 | 19212 | 22555 | 27880 | 687.7 | 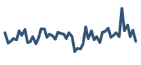 |
| 1455047_at | MGI:1929084 | Fbxo3 | 4413 | 24123 | 19128 | 22671.5 | 7152.2 | 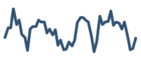 |
| 1428118_at | MGI:1915522 | Lingo1 | 4415.5 | 6840.5 | 15493.5 | 8123.5 | 39.7 | 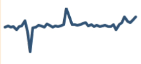 |
| 1451113_a_at | MGI:1345142 | Ik | 4418 | 11976 | 17526 | 19033.5 | 9188.9 | 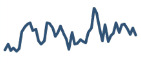 |
| 1445304_at | --- | | 4419 | 37518 | 33826 | 37728.5 | 102.3 | 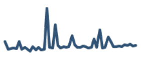 |
| 1429191_at | MGI:2445102 | Dhx33 | 4421 | 6054 | 8311 | 12382 | 853.6 | 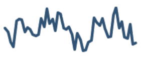 |
| 1445694_at | --- | | 4422 | 34878.5 | 28627 | 21692.5 | 121 | 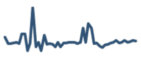 |
| 1455595_at | MGI:3576103 | Ugt2b36 | 4423.5 | 5410.5 | 6581.5 | 5745 | 77699.8 | 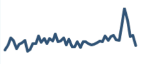 |
| 1445169_at | --- | | 4425 | 42448.5 | 33352 | 37728.5 | 87.4 | 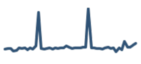 |
| 1445220_at | --- | | 4427 | 27904.5 | 34611 | 16234 | 80.5 | 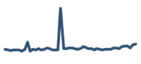 |
| 1416944_a_at | MGI:1346023 | Tlk2 | 4429 | 21186 | 22247.5 | 25035 | 1212 | 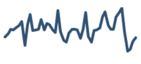 |
| 1452279_at | MGI:97545 | Cfp | 4430 | 8938 | 12489 | 14192.5 | 1648.9 | 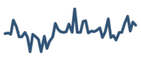 |
| 1424977_at | MGI:1921138 | Lrrc67 | 4431 | 12880 | 24258.5 | 10090 | 171.6 | 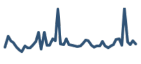 |
| 1439222_at | MGI:3045293 | Morc2b | 4438 | 7445.5 | 5625 | 9085 | 6.7 | 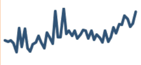 |
| 1438396_at | MGI:109589 | Ocrl | 4439 | 6485 | 6461 | 8727 | 126.3 | 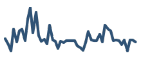 |
| 1421901_at | MGI:1353448 | Eif2ak1 | 4442 | 4690 | 4670 | 6394 | 2024.5 | 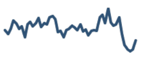 |
| 1444108_at | --- | | 4443.5 | 14038 | 16410 | 25855 | 836.8 | 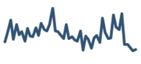 |
| 1423669_at | MGI:88467 | Col1a1 | 4443.5 | 20161 | 19499.5 | 37728.5 | 93.7 | 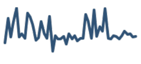 |
| 1427934_at | MGI:1917573 | Lyrm2 | 4446.5 | 21590 | 29658 | 37728.5 | 1024.1 | 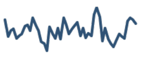 |
| 1427651_x_at | MGI:95896 | H2-D1 | 4449 | 38271 | 26847 | 37728.5 | 143.9 | 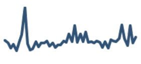 |

| Probe | MGI_ID | Symbol | SW_rank | DL_rank | LS_rank | JTK_rank | Max-Min | Norm Plot |
|---|---|---|---|---|---|---|---|---|
| 1420503_at | MGI:1890216 | Slc6a14 | 4451.5 | 25303.5 | 25672 | 14192.5 | 24.9 | |
| 1459671_at | --- | | 4458 | 7445.5 | 7441 | 5272 | 13.5 | |
| 1444805_at | --- | | 4459 | 38856.5 | 29958 | 37728.5 | 53.6 | |
| 1450570_a_at | MGI:88319 | Cd19 | 4461.5 | 39273.5 | 28699 | 15975.5 | 25.3 | |
| 1424132_at | MGI:96224 | Hras1 | 4463 | 17963 | 19733 | 7734 | 637.6 | |
| 1451311_a_at | MGI:1919924 | Adipor1 | 4465 | 9267 | 6684 | 6650.5 | 4149.8 | |
| 1427638_at | MGI:103222 | Zbtb16 | 4466 | 12052 | 24606 | 6111.5 | 407.5 | |
| 1439235_x_at | MGI:1916992 | Tm2d2 | 4468.5 | 10676 | 8571 | 6050.5 | 3657.3 | |
| 1440743_at | MGI:1921606 | 31428F04R | 4473 | 40934 | 40494 | 21938.5 | 62.1 | |
| 1437668_at | --- | | 4474 | 16157 | 23564 | 24799.5 | 229.4 | |
| 1448702_at | MGI:1913441 | Ier3ip1 | 4478 | 18667 | 20835.5 | 14688 | 5442.3 | |
| 1455530_at | --- | | 4480 | 23785.5 | 19357 | 19316.5 | 68.2 | |
| 1450807_at | MGI:1888501 | Ltb4r2 | 4481 | 25952 | 27507.5 | 16775 | 17.4 | |
| 1438531_at | --- | | 4483 | 6912.5 | 16029 | 7834 | 19.5 | |
| 1424851_at | MGI:1913420 | Chchd5 | 4484 | 39940.5 | 28916.5 | 37728.5 | 14.9 | |
| 1434476_at | MGI:2142523 | Crtc1 | 4485 | 36848 | 28194 | 24799.5 | 13.2 | |
| 1449216_at | MGI:1298377 | Itgae | 4486 | 9617 | 18703.5 | 5426.5 | 114 | |
| 1422434_a_at | MGI:1914623 | 10010C04R | 4487.5 | 5970 | 40494 | 37728.5 | 249266 | |
| 1420890_at | MGI:106911 | Hccs | 4489 | 9351 | 8974.5 | 7925.5 | 3219 | |
| 1431766_x_at | MGI:105110 | Rps2 | 4491 | 7887.5 | 5209.5 | 5844.5 | 16312.7 | |
| 1451668_at | MGI:2443990 | Fam20b | 4492 | 8981 | 18364.5 | 5228 | 1129.1 | |
| 1445420_at | --- | | 4496 | 8193 | 10882.5 | 7544 | 39.4 | |
| 1453188_at | MGI:1915036 | 30424C14R | 4498 | 8114 | 16713 | 6282.5 | 1131.7 | |
| 1445687_at | MGI:2685731 | Gm885 | 4499.5 | 20393 | 22641 | 22916 | 52.3 | |
| 1437499_at | --- | | 4501 | 33906 | 24882 | 19033.5 | 68.1 | |
| 1456597_at | MGI:2444491 | Heatr3 | 4504 | 23220 | 20626.5 | 15443.5 | 712.8 | |
| 1454692_x_at | --- | | 4505 | 5330.5 | 6635 | 19590.5 | 437.8 | |

| Probe | MGI_ID | Symbol | SW_rank | DL_rank | LS_rank | JTK_rank | Max-Min | Norm Plot |
|---|---|---|---|---|---|---|---|---|
| 1433899_x_at | --- | | 4507 | 9934 | 21812.5 | 37728.5 | 2452.1 | 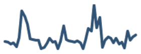 |
| 1442039_at | MGI:2181659 | Tox | 4509.5 | 6267 | 6716.5 | 5645.5 | 42 | 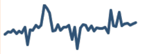 |
| 1431231_at | MGI:2448329 | Hist1h3f | 4511 | 6131 | 19765.5 | 9929 | 146.6 | 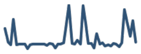 |
