# Supplementary material for: SW1PerS: Sliding windows and 1-persistence scoring; discovering periodicity in gene expression time series data
Source: BMC Bioinformatics. 2015 Aug 16;16:257. doi: 10.1186/s12859-015-0645-6 (PMC4537550; doi:10.1186/s12859-015-0645-6)
Supplement: Additional file 3 — Top genes. This zip file contains three pdf files, associated to each one of the 3 biological data sets studied in this paper. Each file shows the full ordered list, sparkLines included, of genes in the top 10 % of rankings according to SW1PerS and that are not present in the top 10 % of the other algorithms. [file 12859_2015_645_MOESM3_ESM.zip › top_genes/orlando2008-wt1tp13_res__top10p-sw-oth.pdf]

Probe	Sys_Name	Symbol	SW_rank	DL_rank	LS_rank	JTK_rank	Max-Min	Norm Plot
1777544_at	YFL031W	HAC1	18	1645	1472	2600	1845.38	
1769446_at	YHL010C	ETP1	26	1278	1614	2600	29.7434	
1769501_at	YLR266C	PDR8	44	1291	1231	1827	55.2746	
1772076_at	YGL090W	LIF1	69	1825	1159.5	2600	39.7952	
1772087_at	YBR255W	MTC4	70	1373	823.5	681	164.15	
1770289_at	YPR178W	PRP4	72	743	977	940	189.923	
1776356_at	YNL042W	BOP3	76	644	657.5	681	216.511	
1775005_at	YPL013C	MRPS16	98.5	1756	1794	1207	197.587	
1775170_at	YBL010C	---	105	1176	1498	2199.5	209.307	
1775457_at	YPL079W	RPL21B	108	4125	2443	2600	1002.25	
1770709_at	YER007C-A	TMA20	129	2534	2558	1207	1378.02	
1770885_at	YDR387C	---	140	1900	3083.5	2199.5	359.52	
1777548_at	YGL194C	HOS2	143	911	971.5	681	154.573	
1774651_at	YGL203C	KEX1	150	2703	2004.5	3010.5	410.993	
1779435_at	YIR024C	---	157	1018	1673	1827	194.275	
1771675_at	YFR002W	NIC96	165	1253	645.5	1207	396.922	
1778665_at	YJL029C	VPS53	174	3413	1583	2199.5	181.154	
1771967_at	YMR242W-A	---	179	3475	706.5	1495	23.5205	
1775610_s_at	// YCR039C ////	HMRA2 //	180	805	1267.5	681	516.37	
1769670_at	YKL208W	CBT1	184	1315	1414	1207	70.4109	
1780070_at	YBR046C	ZTA1	185	629	677	1207	265.551	
1779577_at	YBR196C-A	---	190	2422	1858	1827	58.3917	
1769806_at	YMR226C	---	192	937	2261	940	4766.1	
1773430_at	YDR376W	ARH1	204	1348	1174	1207	65.9037	
1776655_at	YML100W-A	---	206	3471	932.5	1495	28.4866	
1778520_at	YML020W	---	209	877	1196.5	1495	243.998	
1776520_at	YNL211C	---	214	1862	614	1207	201.798	

Probe	Sys_Name	Symbol	SW_rank	DL_rank	LS_rank	JTK_rank	Max-Min Norm	Plot
1769681_at	YOL105C	WSC3	219	1168	823.5	1207	311.299	
1776959_at	YCR002C	CDC10	221	1224	748.5	1827	770.273	
1779445_at	YDR135C	YCF1	223	735	1958.5	940	2104.74	
1770340_at	YLL058W	---	226	974	773	1495	205.485	
1772112_at	YGR028W	MSP1	227	1411	1034.5	1207	120.695	
1773219_at	YGL164C	YRB30	230.5	1102	1931.5	1495	452.264	
1773020_at	YIL108W	---	242	1361	2095	1207	554.592	
1772369_at	YJL197W	UBP12	244	829	992.5	940	492.041	
1770549_at	YDL057W	---	246	1368	1196.5	1495	75.9847	
1771830_at	YLR291C	GCD7	247	1658	963.5	1207	929.006	
1778418_at	YKL017C	HCS1	254	1990	1159.5	1495	60.7778	
1778574_at	YGL145W	TIP20	256	2171	1572.5	1495	114.195	
1773923_at	YOR192C	THI72	258	1556	867.5	940	33.6165	
1772581_at	YGR001C	---	259	1167	1414	681	1378.14	
1772167_at	YKL018C-A	---	260	755	1578	1207	499.332	
1780013_at	YLR284C	ECI1	263	1239	1213	2199.5	59.4026	
1775342_at	YJR070C	LIA1	270	2252	2672.5	1827	1149.8	
1777707_at	YBR047W	FMP23	271	1328	4179	1495	306.043	
1774438_at	YMR277W	FCP1	274	1661	1489	1495	277.387	
1769766_at	YBR296C	PHO89	276	4014	1387.5	2600	167.86	
1779963_at	YLR287C	---	280.5	2037	2079	1827	167.109	
1777758_at	YBR290W	BSD2	295	784	1167.5	1207	279.491	
1775039_at	YDR004W	RAD57	296	2515	1261.5	2199.5	199.375	
1778151_at	YNL191W	DUG3	305	1628	2177	2199.5	315.368	
1776248_at	YLR090W	XDJ1	306	1061	1620.5	681	301.355	
1774221_at	YNL125C	ESBP6	309	1812	594	1827	338.64	
1769626_at	YMR291W	---	312	1183	1693.5	3010.5	124.754	

Probe	Sys_Name	Symbol	SW_rank	DL_rank	LS_rank	JTK_rank	Max-Min	Norm Plot
1778103_at	YGR234W	YHB1	315	1794	1645	3010.5	2508.56	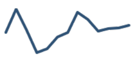
1779416_at	YNL067W-B	---	318	4824	896	2199.5	58.0273	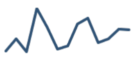
1778008_at	YPR078C	---	324	658	1303	2600	325.687	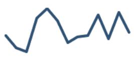
1773639_at	YDL099W	BUG1	326	1964	755	1495	276.041	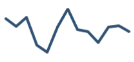
1779377_at	YER125W	RSP5	332	1934	2834	2199.5	1115.88	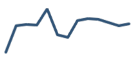
1779349_at	YDL102W	POL3	337	647	1990	940	934.036	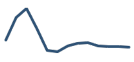
1776947_at	YIR009W	MSL1	339	883	1125.5	1207	181.071	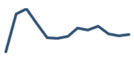
1776333_at	YPR114W	---	344	1232	1146	1207	1083.52	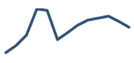
1770245_at	YNR027W	BUD17	345.5	915	873.5	940	172.935	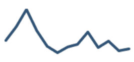
1778196_at	YKR095W	MLP1	348	1954	1534.5	1827	254.323	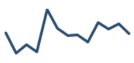
1778317_at	YGL256W	ADH4	350	685	1089	1207	844.036	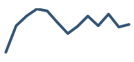
1779302_at	YIR001C	SGN1	352	1642	1640.5	1495	154.052	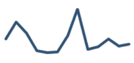
1775712_at	YNL003C	PET8	353	1462	1243	2199.5	319.583	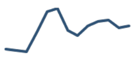
1771015_at	YPR179C	HDA3	358	731	2600	681	265.744	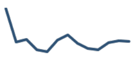
1778033_at	YKL040C	NFU1	361	1417	735	940	169.373	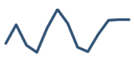
1778389_at	YGR196C	FYV8	363	1123	664.5	1207	99.6198	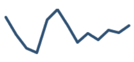
1779422_at	YJR062C	NTA1	366	2100	1507	1827	61.5622	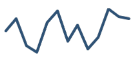
1774909_at	YDR125C	ECM18	370	1313	1318.5	1207	79.4283	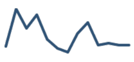
1772625_at	YEL046C	GLY1	375	1951	2593	1495	2961.29	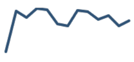
1779003_at	YFR028C	CDC14	382	1025	1089	940	541.568	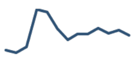
1776096_at	YER134C	---	383	2806	2144	1495	228.634	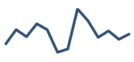
1774085_at	YJL178C	ATG27	384	1213	798.5	1495	523.081	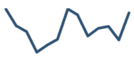
1777819_at	YJR043C	POL32	385	630	1813.5	681	771.301	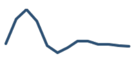
1773059_at	YGL124C	MON1	387	904	998	940	84.9236	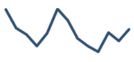
1769368_at	YOR290C	SNF2	391	1468	1387.5	681	637.728	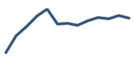
1779660_at	YLR174W	IDP2	397	875	2408.5	1827	215.211	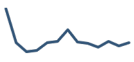
1770412_at	YML007C-A	---	400	1550	2153	1495	249.202	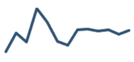

Probe	Sys_Name	Symbol	SW_rank	DL_rank	LS_rank	JTK_rank	Max-Min	Norm Plot
1778683_at	YNL042W-B	---	401	3861	706.5	1207	60.604	
1771091_at	YJR150C	DAN1	404	2525	2531	2600	31.0167	
1770766_at	YIL009C-A	EST3	408	1602	3078	940	442.003	
1778226_at	YKR020W	VPS51	410	2376	1449.5	2199.5	29.1047	
1778804_at	YER011W	TIR1	416	1667	2226.5	3010.5	1256.53	
1772876_at	YGL022W	STT3	420	692	1081.5	1827	2399.32	
1778279_at	YBR265W	TSC10	422	943	1095	1207	631.054	
1770108_at	YKL047W	---	424	2521	1940	1827	202.99	
1777465_at	YIL031W	ULP2	425	2358	2759.5	1827	154.114	
1773273_at	YBL015W	ACH1	437	1926	640.5	940	198.594	
X-Scv2-r2-Bs-dap	---	---	438	1200	1996	1495	2570.42	
1772288_at	YOR038C	HIR2	442	990	1419.5	940	443.692	
1779825_at	YNR065C	---	443	832	2973.5	1207	309.181	
1769781_at	YDR393W	SHE9	451	922	1656	940	54.2582	
1776496_at	YML111W	BUL2	452	2103	1907	3010.5	145.943	
1772310_at	YLR207W	HRD3	457	3802	3125	3010.5	124.852	
1779735_at	YBR278W	DPB3	459	672	1759	940	421.031	
1771981_at	YGL110C	CUE3	461	2273	2219.5	1495	217.387	
1774169_at	YMR307W	GAS1	462	601	1018.5	940	6496.56	
1776440_at	YNL323W	LEM3	467	674	664.5	940	488.307	
1779365_at	YML104C	MDM1	469	1005.5	1284	1495	462.572	
1777640_at	YLL054C	---	470	2558	735	1207	99.6131	
1777493_at	YNL127W	FAR11	472.5	2637	1333.5	2199.5	158.293	
1778010_at	YPL076W	GPI2	476	2084	2539	2199.5	174.636	
1774917_at	YPR023C	EAF3	477	1089	838	940	554.852	
1779583_at	YIL060W	---	480	1905	1907	1495	36.1079	
1770443_at	YJL085W	EXO70	485	1813	1267.5	1495	261.913	

Probe	Sys_Name	Symbol	SW_rank	DL_rank	LS_rank	JTK_rank	Max-Min	Norm Plot
1779272_at	YGL056C	SDS23	487	3808	4787	4765.5	147.286	
1773187_at	YMR106C	YKU80	489	1542	1711	940	124.271	
1770884_s_at	29W /// YNL2	SB1 /// SSB	492	2887.5	1628.5	1495	591.766	
1779317_at	YDR305C	HNT2	493	884	902.5	1207	286.483	
1774477_at	YER007W	PAC2	494	970	1309	940	219.274	
1775089_at	YNL204C	SPS18	495	2588	2698	3010.5	107.134	
1777092_at	YGR276C	RNH70	497	2032	855.5	1207	304.262	
1778824_at	YJL049W	---	499	1506	873.5	1495	117.508	
1772743_at	YDR369C	XRS2	500	2205	3156.5	2199.5	111.679	
1774087_at	YDL239C	ADY3	501	2218	1836	2199.5	74.7577	
1771211_at	YLR037C	PAU23	503	3803	3117	4765.5	2386.26	
1777088_at	YBR272C	HSM3	505	2289	2322.5	2199.5	107.985	
1778038_at	YDR091C	RLI1	507	1880	3225.5	1495	2730.41	
1773751_at	YDL233W	---	508	2114	2818.5	1207	98.4923	
1776484_at	YLR154W-F	---	509	3006	3117	2199.5	24.4441	
1777298_at	YIL152W	---	511	2419	1566	1495	106.697	
1778869_at	YOR256C	TRE2	512	1325	2346.5	1827	397.96	
1769694_at	YBL036C	---	516	1706	1954.5	1495	1027.92	
1773859_at	YBR132C	AGP2	519	2945	2969	4765.5	232.421	
1776036_at	YMR070W	MOT3	522	3234	2593	2199.5	546.207	
1779737_at	YDL165W	CDC36	528	2452	3750	4765.5	678.261	
1775529_at	YER060W-A	FCY22	531	2955	3548	4765.5	210.628	
1769516_at	YDL086W	---	532	963	2060	940	3072.94	
1775766_at	YOR113W	AZF1	536	3495	3202.5	4765.5	172.105	
1778190_at	YGL099W	LSG1	542	1136	1846	681	996.449	
1772774_at	YOL130W	ALR1	546	1285	2032.5	1495	462.807	
1778658_at	YKL064W	MNR2	547	1126	1267.5	940	270.503	

Probe	Sys_Name	Symbol	SW_rank	DL_rank	LS_rank	JTK_rank	Max-Min	Norm Plot
1774303_at	YDR303C	RSC3	550	1518	1369.5	1495	489.084	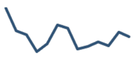
1779309_at	YOL058W	ARG1	551	610	645.5	940	1673.61	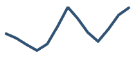
1776874_at	YMR167W	MLH1	552	1570	2089.5	1207	58.8366	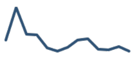
1776635_at	YGR146C-A	---	555	1452	1751.5	681	64.7761	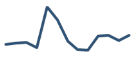
AFFX-LysX-3_at	---	---	559	1855	2600	3010.5	362.396	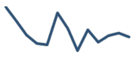
1779920_at	YER034W	---	560	2026	1880.5	1495	269.04	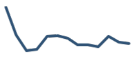
1770458_at	YOR032C	HMS1	561	2772	1444	2199.5	36.282	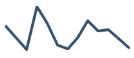
1771219_at	YFR019W	FAB1	562	1323	992.5	1495	165.218	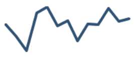
FX-Scv2-TrpnX-3	---	---	565	3707	3370.5	3010.5	35.1233	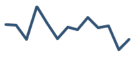
1775860_at	YPL022W	RAD1	567	1125	786	1207	152.919	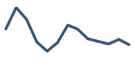
1776783_at	YDR177W	UBC1	568	1399	670	940	610.413	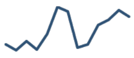
1773975_at	YJL095W	BCK1	571	1221	798.5	1495	142.296	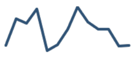
1770211_at	YEL062W	NPR2	574	2256	3282	940	237.542	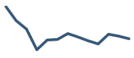
1773688_at	YLR446W	---	578	1595	1738	3426	72.3045	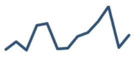
1774119_at	YJL047C	RTT101	581	1103	1693.5	1207	197.463	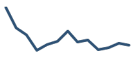
1772251_at	YJL189W	RPL39	588	2347	1419.5	1207	1732.36	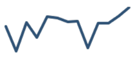
