# Supplementary material for: SW1PerS: Sliding windows and 1-persistence scoring; discovering periodicity in gene expression time series data
Source: BMC Bioinformatics. 2015 Aug 16;16:257. doi: 10.1186/s12859-015-0645-6 (PMC4537550; doi:10.1186/s12859-015-0645-6)
Supplement: Additional file 3 — Top genes. This zip file contains three pdf files, associated to each one of the 3 biological data sets studied in this paper. Each file shows the full ordered list, sparkLines included, of genes in the top 10 % of rankings according to SW1PerS and that are not present in the top 10 % of the other algorithms. [file 12859_2015_645_MOESM3_ESM.zip › top_genes/tu2005-evensamp_res__top10p-sw-oth.pdf]

Probe	Sys_Name	Symbol	SW_rank	DL_rank	LS_rank	JTK_rank	Max-Min	Norm Plot
5187_at	YGL192W	IME4	77	3142.5	1268	1230.5	0.65	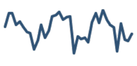
6335_at	YDR144C	MKC7	78	1179	1143	1284	1.03	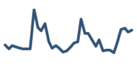
4834_at	YGR179C	OKP1	94	2812	1202	1541	1.54	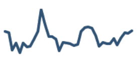
8855_at	YNR007C	ATG3	101	1996	1884.5	2332.5	2.33	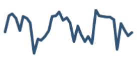
7301_at	YBR078W	ECM33	134	1355.5	1170.5	1444.5	35.02	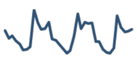
7302_at	YBR078W	ECM33	137	1552	1194.5	1492	35.86	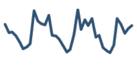
6450_at	YDR035W	ARO3	150.5	2508	1146.5	1541	2.65	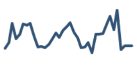
5936_at	YDR512C	EMI1	165	3049	1002	1085	6.54	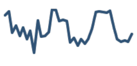
9918_at	YLR383W	SMC6	167	2604	1238.5	1597.5	2.02	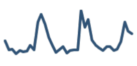
9823_at	---	---	201	1902	993.5	1952.5	12.93	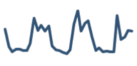
10902_at	YJR109C	CPA2	206.5	2436.5	1092	2714.5	11.42	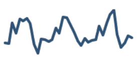
5685_at	YER030W	CHZ1	216	2858.5	1951	2656.5	7.96	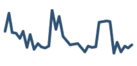
8344_at	YOR188W	MSB1	227	2747.5	1568.5	2768.5	1.66	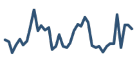
4943_at	YGR062C	COX18	235	2169	1526.5	1338	4.94	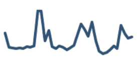
4244_at	YIL157C	COA1	237	1431	1131	1230.5	8.21	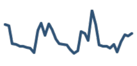
6806_at	YCR065W	HCM1	239	2140	1259	1180	2.33	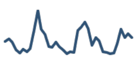
9149_at	YNL290W	RFC3	244.5	2515	998.5	1444.5	2.49	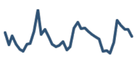
5602_at	YER118C	SHO1	255	1529.5	964	1952.5	1.85	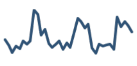
5719_at	YER019W	ISC1	262	2721	996	1131.5	1.77	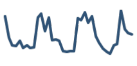
4479_at	YHR049C-A	---	265	1416	1616.5	1391	0.42	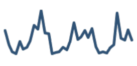
7763_at	YPR011C	---	269	2379	1045	1338	4.43	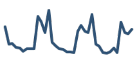
4847_at	YGR147C	NAT2	275	2985.5	1854	1702.5	3.86	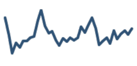
6656_at	YDL164C	CDC9	291	1494	1993.5	2714.5	2.81	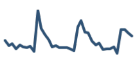
11078_at	YJL034W	KAR2	296	2391	1227	1230.5	24.50	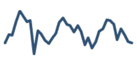
7409_at	YBL043W	ECM13	299	2127.5	1850	3440	7.78	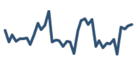
4312_at	YHR198C	AIM18	305.5	1596	1572.5	2120	3.38	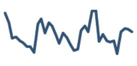
11044_at	YJL023C	PET130	319	2430	1203	1802	0.84	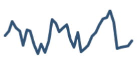

Probe	Sys_Name	Symbol	SW_rank	DL_rank	LS_rank	JTK_rank	Max-Min	Norm Plot
7901_at	YPL124W	SPC29	334	1042	962	1180	0.99	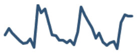
9638_at	YMR016C	SOK2	343	1021	1440.5	1597.5	3.00	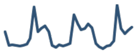
7707_i_at	YPR043W	RPL43A	347	3409.5	1808	1284	21.74	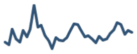
6639_at	YDL135C	RDI1	350	2824.5	1591	2010	5.00	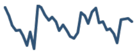
5035_at	YGL027C	CWH41	355.5	2393.5	2237.5	3289.5	3.83	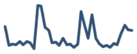
4824_at	YGR169C	PUS6	360	1278.5	1042.5	990	2.51	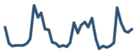
5349_at	YFR007W	YFH7	362	1485.5	1344	2596.5	2.46	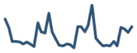
6902_at	YCL030C	HIS4	362	2537.5	1643.5	1753.5	15.85	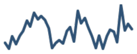
4814_at	YGR204W	ADE3	364	1824.5	1033	1753.5	16.70	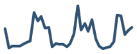
6727_at	YDL225W	SHS1	365.5	2776.5	1760	2391.5	1.81	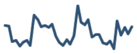
6042_at	YDR435C	PPM1	377.5	1777	1405	2391.5	2.96	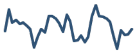
3804_f_at	G)D2 /// tQ(U	---	379.5	8408	6976.5	8173.5	0.82	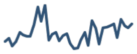
10607_at	YKL045W	PRI2	389	1233	1938	1541	4.30	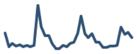
7191_f_at	YBR189W	RPS9B	392.5	2146	1555.5	1541	40.49	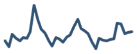
7824_at	YPL065W	VPS28	392.5	3409.5	1125.5	2120	1.14	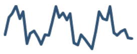
6585_at	YDL101C	DUN1	398.5	2521	1861	3614.5	1.09	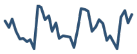
8836_at	YNR033W	ABZ1	409	3282.5	1936.5	2068	2.53	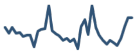
6199_at	YDR279W	RNH202	415	3233.5	2039	2332.5	0.94	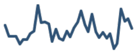
8508_at	YOR035C	SHE4	417	2463.5	1280.5	1899.5	2.13	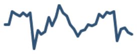
7417_at	YBL035C	POL12	421	954	1799	1391	5.94	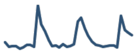
7681_at	YPR061C	JID1	426	2093	1579.5	2010	1.65	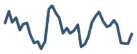
10984_at	YJR055W	HIT1	434.5	1782	3282	3890.5	0.42	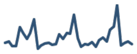
7190_i_at	YBR189W	RPS9B	436	2124	1447.5	1085	24.30	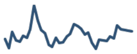
9592_at	YMR062C	ARG7	438	2910	1184.5	1131.5	6.22	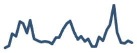
8578_at	YOL029C	---	443.5	2687	1561.5	1180	2.60	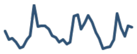
6797_at	YCR102C	---	445	1359.5	2436.5	1952.5	10.45	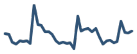
8305_at	YOR239W	ABP140	454	2307.5	1067	1131.5	1.59	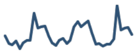

Probe	Sys_Name	Symbol	SW_rank	DL_rank	LS_rank	JTK_rank	Max-Min	Norm Plot
5395_at	YFL036W	RPO41	461	2910	1179	2391.5	4.45	
5636_at	YER065C	ICL1	466.5	2531.5	1325	1036.5	26.14	
4869_at	YGR124W	ASN2	473	2022	1282.5	1085	14.37	
9766_at	YML119W	---	480	2349	1718.5	1131.5	1.17	
4456_at	YHR075C	PPE1	482	1074	1063	2214	1.51	
8033_at	YPL262W	FUM1	483	2253	1213	2868	28.86	
6819_at	YCR079W	PTC6	484.5	1697	1007	1230.5	3.39	
5710_at	YER010C	---	490.5	3282.5	1334	2596.5	3.69	
11236_at	YJL190C	RPS22A	495.5	1405	1481	990	92.08	
6562_at	YDL080C	THI3	506	2756.5	1696.5	1085	3.86	
8600_at	YOL052C	SPE2	507	1078	954	990	7.05	
9094_s_at	29W /// YNL2	SSB1 /// SSB	510.5	1820	1991	1651	40.20	
7296_at	YBR073W	RDH54	514.5	2898	1129	1651	0.63	
11392_at	YAL056W	GPB2	528	1225.5	1515.5	1899.5	1.09	
10319_at	YLR023C	IZH3	533	1529.5	1330	2768.5	6.70	
8588_at	YOL019W	---	536	1308	2363	3341.5	2.56	
5183_at	YGL196W	DSD1	540	2038	1004.5	2391.5	5.02	
3791_s_at	L177C /// YJL	/// YRF1-5 //	542.5	1122	2250.5	1131.5	2.66	
6844_at	YCR059C	YIH1	544	2380.5	1461.5	1284	3.14	
8844_at	YNR041C	COQ2	545	2224.5	1747	1541	2.16	
4091_at	YIR010W	DSN1	546	3088	2452.5	3073	1.92	
9209_at	---	---	562.5	2135.5	1175	1230.5	24.01	
7206_at	YBR160W	CDC28	565	3409.5	1508	1651	1.58	
6468_at	YDR007W	TRP1	566	3233.5	1408	1492	4.27	
5433_at	YFL047W	RGD2	567.5	3282.5	1139.5	1597.5	1.49	
4864_at	YGR163W	GTR2	573	3409.5	2177	3125.5	1.19	
5954_at	YDR530C	APA2	575	3173	2021	3073	1.20	

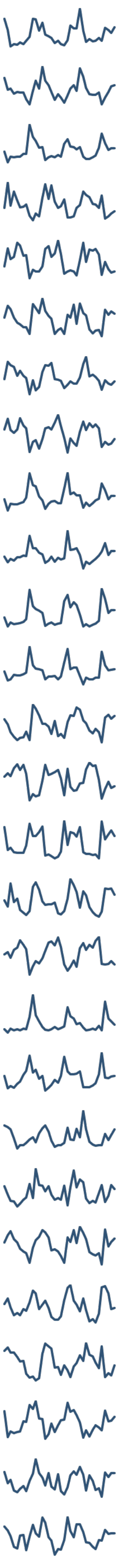

Probe	Sys_Name	Symbol	SW_rank	DL_rank	LS_rank	JTK_rank	Max-Min	Norm Plot
5645_at	YER072W	VTC1	583	2928.5	1088.5	2068	8.90	
10194_at	YLR121C	YPS3	586	3282.5	2569.5	3125.5	0.68	
5244_at	YGL226W	MTC3	588.5	1937.5	1104.5	1180	2.67	
10725_at	YKL148C	SDH1	599	1495	1473	2214	17.55	
9326_at	YMR277W	FCP1	601.5	2961.5	2622	2714.5	1.52	
4930_at	YGR094W	VAS1	603.5	2985.5	1346	2486	5.74	
4287_s_at	YHR209W	CRG1	608	1036	1778.5	3024	1.45	
10103_at	YLR211C	---	611.5	1630	1521.5	2656.5	2.21	
5728_i_at	YEL017C-A	PMP2	611.5	2919	1675.5	1036.5	30.20	
10161_g_at	502C /// YLR1	AM1 /// SAM	628	1133	1723	3289.5	60.82	
6685_at	YDL180W	---	629.5	3088	1508	1651	2.02	
9643_at	YML021C	UNG1	632.5	1582.5	1191	1131.5	2.04	
5100_at	YGL097W	SRM1	639	1469.5	2245	1284	7.73	
5582_at	YER142C	MAG1	646	2135.5	1502	1702.5	4.42	
9262_at	---	---	648	1217	1151	2486	0.47	
6472_at	YDR011W	SNQ2	652	2834	1188.5	990	8.29	
9736_at	YML106W	URA5	654	2332.5	2525	2540	20.31	
3770_i_at	RDN5-3	---	655.5	1771	1703.5	1597.5	18.84	
5318_s_at	YFL031W	HAC1	657	2706	1255	2068	18.59	
7209_at	YBR162W-A	YSY6	662	2864	1399	1651	4.72	
5952_at	YDR528W	HLR1	669	1505	2663	1802	0.98	
7367_at	YBR008C	FLR1	672	2340	2501	2010	4.08	
9047_at	YNL166C	BNI5	674	1570.5	952.5	1180	1.11	
5635_at	YER064C	---	675	964	1666	1036.5	3.04	
7881_at	YPL144W	POC4	682	2884.5	1677	1851.5	2.97	
6497_at	YDL010W	GRX6	689	2232	1057	1597.5	3.06	
6039_g_at	32W /// YDR4	NPL3	691	1943	1988.5	2010	23.98	

Probe	Sys_Name	Symbol	SW_rank	DL_rank	LS_rank	JTK_rank	Max-Min	Norm Plot
10156_at	YLR176C	RFX1	692	3105.5	1764	1541	1.39	
11021_f_at	YJL001W	PRE3	693	2115	2030	1180	10.83	
5659_at	YER048C	CAJ1	697	3409.5	1444.5	1036.5	4.24	
7127_at	YBR217W	ATG12	700	2249	1568.5	2596.5	0.71	
5328_at	YFR031C	SMC2	711	2919	1047.5	1036.5	2.61	
6377_at	YDR097C	MSH6	715	3569	2381	3341.5	5.06	
7435_at	YBL061C	SKT5	722	1288	1740	1899.5	4.12	
10478_at	YKR097W	PCK1	726	963	1184.5	1180	15.95	
4956_at	YGR028W	MSP1	728	1635	1696.5	2540	4.08	
8535_at	YOR017W	PET127	730	2775	1490	2391.5	1.42	
5468_at	---	---	731	1062	1826.5	2768.5	4.09	
5993_at	YDR479C	PEX29	734	2537.5	1555.5	948	3.37	
7325_at	YBR057C	MUM2	735	2630	1301	1651	2.81	
6671_at	YDL192W	ARF1	736.5	2935.5	1551	1651	34.67	
3939_at	YBR006W	UGA2	741	1964	2508	2656.5	2.59	
6885_at	YCR008W	SAT4	743	2073	2257	1284	4.76	
4527_at	YHR009C	---	745	3088	2289.5	3672.5	3.91	
6070_s_at	[illegible]418W /// YEL[illegible]12A /// RPL[illegible]		761	3233.5	1907.5	1492	20.15	
7378_s_at	[illegible]27W /// YBR0[illegible]19A /// RPL[illegible]		766	2714	1425	2164.5	56.06	
9080_at	YNL223W	ATG4	775	1768	1647	2486	0.99	
7797_at	YPL046C	ELC1	780	3409.5	1791.5	1338	1.62	
4491_at	YHR063C	PAN5	785.5	1250.5	998.5	3341.5	5.80	
4247_at	YIL154C	IMP2'	785.5	1592	1842.5	2120	2.34	
9765_at	YML120C	NDI1	794	1247.5	1277	1492	24.50	
10957_at	YJR073C	OPI3	795	1338	1255	1338	23.55	
10843_at	---	---	796	1694.5	1919	2540	0.37	
4832_at	YGR177C	ATF2	801	2103	1912	3183.5	0.39	

Probe	Sys_Name	Symbol	SW_rank	DL_rank	LS_rank	JTK_rank	Max-Min	Norm Plot
4668_at	---	---	802	1551	1301	1597.5	0.63	
10738_i_at	YKL180W	RPL17A	811	2387	1121	948	44.74	
8320_at	YOR209C	NPT1	812	2773	1196	1541	6.25	
7994_at	YPL255W	BBP1	818.5	1123.5	1192.5	948	0.62	
4889_at	YGR099W	TEL2	826	3409.5	2320	4054	0.76	
6202_at	YDR282C	---	831	3409.5	1371	1541	1.04	
6357_at	YDR122W	KIN1	832	1442	1181	2010	3.02	
10097_at	YLR249W	YEF3	834	1896.5	1250	1230.5	65.16	
7214_at	YBR167C	POP7	834	2725	1372.5	1284	1.98	
4462_at	YHR080C	---	837	1840	1121	1444.5	2.98	
5716_at	YER016W	BIM1	843	3233.5	1342	1541	1.01	
8590_at	YOL017W	ESC8	845	955.5	2970	3492.5	0.89	
8509_at	YOR036W	PEP12	848	1900.5	1069.5	1541	2.11	
4584_at	YHL024W	RIM4	850	1493	1436.5	1492	13.66	
9141_at	YNL298W	CLA4	854	1322	1800.5	1541	3.98	
8491_at	YOR063W	RPL3	855.5	2658	1326	948	27.63	
7549_g_at	97W /// YPR1	MAL33	858	3105.5	1951	3341.5	6.47	
6519_at	YDL033C	SLM3	860	3409.5	1177	2768.5	0.80	
7067_at	YBR294W	SUL1	861.5	2985.5	1596.5	1284	14.17	
9304_at	YMR298W	LIP1	863	3409.5	1811.5	3125.5	5.19	
10385_i_at	033C /// YLL0[illegible]L8A /// RPL		865	2559	2582.5	2214	31.39	
5979_at	YDR510W	SMT3	866	3409.5	1896.5	2164.5	3.08	
4759_at	YGR239C	PEX21	871	1514	2262.5	2391.5	2.83	
7295_at	YBR072W	HSP26	872	1121	1030	3024	51.65	
5263_at	YGL250W	RMR1	877	1480.5	1383.5	2270	1.14	
10301_at	YLR048W	RPS0B	877	1874	1904.5	1085	55.46	
8566_at	YOR005C	DNL4	884	3409.5	1942	2068	0.56	

Probe	Sys_Name	Symbol	SW_rank	DL_rank	LS_rank	JTK_rank	Max-Min	Norm Plot
9501_at	YMR146C	TIF34	887.5	2525	2735.5	2486	14.44	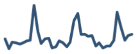
10620_at	YKL032C	IXR1	889	2368.5	1262	1541	7.34	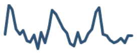
5058_at	YGL049C	TIF4632	891	2434	2322.5	2656.5	1.16	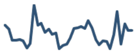
10753_at	YKL166C	TPK3	894	1461	2081.5	1338	1.59	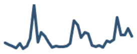
4935_at	YGR054W	---	895	1709	1831.5	1492	3.14	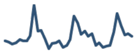
9237_at	---	---	900	1507	1047.5	1338	1.61	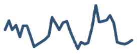
7374_at	YBL031W	SHE1	903	1731	1073.5	1284	0.49	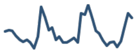
9923_f_at	YLR388W	RPS29A	906	2439.5	2267	1085	43.62	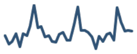
8874_at	YNL021W	HDA1	910	2057.5	1456	1802	0.61	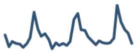
5048_at	YGL059W	PKP2	911	1187	2018.5	3289.5	1.67	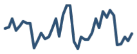
7105_at	YBR240C	THI2	916.5	2325	2240	1230.5	9.19	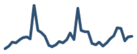
6539_at	YDL059C	RAD59	916.5	2475	1495	2120	1.43	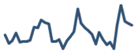
5755_at	YEL035C	UTR5	920.5	1475.5	1123.5	1036.5	0.57	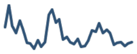
7245_at	YBR109C	CMD1	920.5	3173	1627	2010	14.26	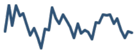
6073_at	YDR376W	ARH1	922	2368.5	1114.5	1753.5	2.80	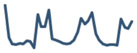
10180_at	YLR150W	STM1	932	3122	1461.5	1541	25.01	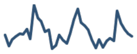
8312_at	YOR246C	---	932	3409.5	1703.5	2214	3.05	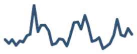
